# Total Synthesis of (+)‐Melonine and (+)‐*N_4_*‐Oxy Melonine Enabled by an Intramolecular Alkene Diamination Reaction

**DOI:** 10.1002/anie.8101956

**Published:** 2026-01-09

**Authors:** Vincent Goëlo, Qian Wang, Jieping Zhu

**Affiliations:** ^1^ Laboratory of Synthesis and Natural Products (LSPN) Institute of Chemical Sciences and Engineering Ecole Polytechnique Fédérale de Lausanne EPFL‐SB‐ISIC‐LSPN, BCH 5304 Lausanne 1015 Switzerland

**Keywords:** Alkene diamination, Asymmetric synthesis, Indole alkaloids, Melonine, Total synthesis

## Abstract

Among more than four thousand monoterpene indole alkaloids (MIAs) isolated to date, only a few feature a 2,2,3‐trisubstituted indoline moiety. (+)‐Melonine and (+)‐*N_4_*‐oxy melonine possess a highly rearranged carbon framework, presumably arising from cyclization of a rearranged iminium ion of quebrachamine precursor. We report herein the first enantioselective total synthesis of (+)‐melonine and (+)‐*N_4_*‐oxy melonine featuring: a) a highly enantioselective CBS reduction followed by a stereospecific Johnson‐Claisen rearrangement for the synthesis of enantioenriched *β*‐substituted *γ*,*δ*‐unsaturated ester; b) Bower's bis‐cyclizative diamination of alkene, enabling the conversion of a functionalized cycloheptene to the tetracyclic core of the natural products; and c) an AlMe_3_‐mediated lactamization that concurrently achieves desymmetrization at the C20 prochiral center.

## Graphical abstract

A complexity‐generating intramolecular alkene diamination constitutes the pivotal step in the enantioselective synthesis of the title natural products featuring a strained, cage‐like pentacyclic architecture.

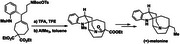

Melonine was first isolated in 1978 by Rabaron et al.^[^ ^1^ ^]^ from the stems of *Melodinus philliraeoides* (*Apocynaceae*)^[^ ^2^ ^]^ collected in New Caledonia. Its structure was initially assigned by Baassou et al. in 1983 as a pentacyclic compound featuring an unusual C2‐C6 bond (Scheme 1a).^[^ ^3^, ^4^, ^5^ ^]^ In 2021, Beniddir, Le Pogam and co‐workers re‐isolated (+)‐melonine (**1**) and revised its structure through detailed spectroscopic analysis and DFT calculations.^[^ ^6^ ^]^ The absolute configuration, (2*R*,3*S*,7*S*,20*R*), was established by comparison between the TDDFT‐calculated and experimental electronic circular dichroism (ECD) spectra. The structure of (+)‐*N*_4_‐oxy melonine (**2**) was revised accordingly.

**Scheme 1:**
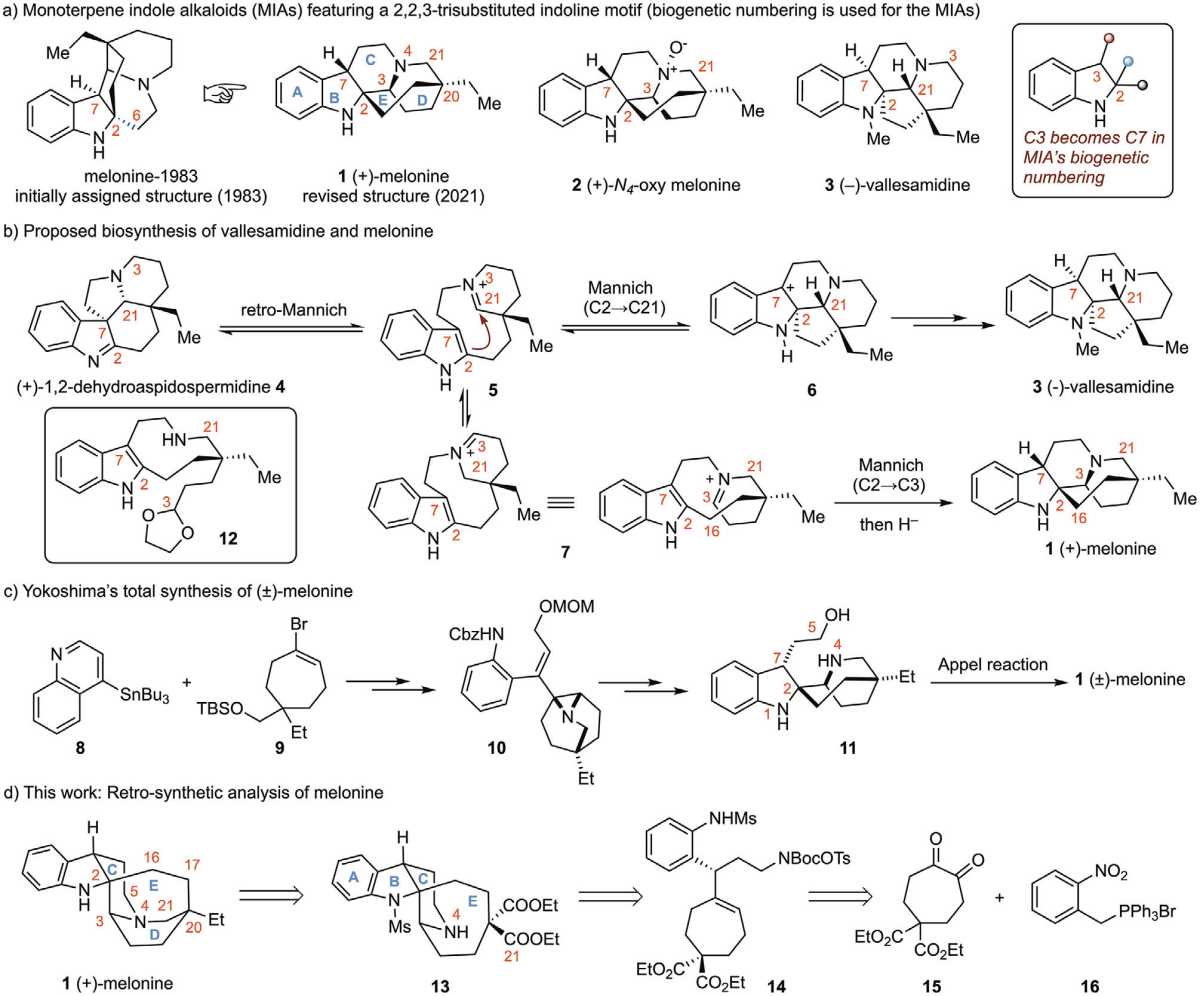
(+)‐Melonine and (+)‐*N_4_*‐oxy melonine: structure and synthesis.

Melonine (**1**) belongs to a small subfamily of monoterpene indole alkaloids (MIAs), known as the schizozygane alkaloids.^[^ ^7^ ^]^ A defining structural feature of this class, exemplified by (–)‐vallesamidine (**3**),^[^ ^8^ ^]^ is the presence of a 2,2,3‐trisubstituted indoline motif.^[^ ^9^ ^]^ Possible biosynthetic pathways leading from dehydroaspidospermidine (**4**) to both melonine (**1**) and vallesamidine (**3**) are depicted in Scheme 1b. A retro‐Mannich reaction of **4** would generate the bridged iminium ion **5**, a precursor of quebrachamine. Intramolecular nucleophilic addition of C2 to the iminium moiety would furnish intermediate **6**, which upon reduction and *N*‐methylation, would afford vallesamidine (**3**).^[^ ^10^ ^]^ Alternatively, iminium **5** could undergo 1,3‐prototropy to the isomeric iminium ion **7**.^[^ ^11^ ^]^ Subsequent transannular cyclization via C2─C3 bond formation followed by reduction of the resulting carbocation would yield melonine (**1**).^[^ ^6^ ^]^ Although rare, a related 1,3‐prototropy has been observed and explored in the efficient conversion of condyfoline to tubifoline.^[^ ^12^ ^]^

In 2025, Yokoshima and co‐workers achieved the first total synthesis of (±)‐melonine (**1**) (Scheme 1c), thereby confirming the revised structural assignment.^[^ ^13^ ^]^ Starting from the Stille cross coupling between 4‐(tributylstannyl)quinoline (**8**) and vinyl bromide **9**, (±)‐melonine (**1**) was synthesized in 23 steps in the longest linear sequence. The synthesis features a key intramolecular aziridine ring‐opening of intermediate **10** followed by construction of the piperidine ring via intramolecular Appel reaction of amino alcohol **11**, culminating in an elegant synthesis of the target molecule.

Our group has long been interested in the synthesis of alkaloids bearing a 2,2‐disubstituted indoline^[^ ^14^, ^15^, ^16^, ^17^ ^]^ unit as well as schizozygane type natural products.^[^ ^18^ ^]^ In approaching the synthesis of melonine (**1**), we initially investigated the cyclization of iminium salt **7** (See Supporting Information), inspired by the proposed biosynthetic pathway. The iminium ion **7** was generated by acidic treatment of compound **12**, which was readily prepared from easily accessible starting materials.^[^ ^19^, ^20^ ^]^ However, all attempts to achieve the conversion of **7** to **1**–or directly from **12** ^[^ ^21^, ^22^, ^23^, ^24^ ^]^ were unsuccessful, likely due to the unfavorable conformational characteristics of the iminium ion **7**.

A three‐dimensional structure of melonine (**1**), constructed using a Dreiding stereomodel, is depicted in Scheme 1d. In this structure, the quinolizidine unit (C─D ring) features a *cis*‐fused ring junction, with a D ring adopting a boat conformation to accommodate the C16─C17 bridge spanning the C2 and C20 carbons. This conformational arrangement necessitates distortion from the preferred geometry of the indolo[2,3‐*a*]quinolizidine skeleton,^[^ ^25^, ^26^, ^27^ ^]^ thereby increasing the ring strain. Yokoshima previously accomplished the total synthesis of melonine (**1**) through a strategically designed sequence involving construction of the C ring onto a preassembled D‐E ring system. Guided by this conformational analysis, we envisioned accessing the pentacyclic framework via formation of the N4─C21 bond from fused tetracyclic compound. Our retro‐synthetic analysis is outlined in Scheme 1d. Specifically, we anticipated that the N4─C21 bond could be forged through lactamization of amino diester **13**, which in turn could arise from a bis‐cyclizative diamination of cycloheptene **14**. The readily accessible cycloheptanedione derivative **15** and Wittig reagent **16** were identified as suitable starting materials for the synthesis of enantioenriched compound **14**. Herein, we report the successful realization of this strategy, culminating in a concise enantioselective synthesis of (+)‐melonine (**1**) in 15 steps in the longest linear sequence from ethyl malonate. Oxidation of **1** with *m*‐CPBA (*meta*‐chloroperoxybenzoic acid) affords the corresponding (+)‐*N_4_*‐oxy melonine (**2**) in quantitative yield.

The synthesis of the intermediate **14** is depicted in Scheme 2. Double alkylation of ethyl malonate (**17**) with 4‐bromobut‐1‐ene (**18**) afforded **19**, which underwent Ru‐catalyzed one‐pot intramolecular ring closing metathesis/*α*‐ketohydroxylation sequence to afford the desired cycloheptanone derivative **20** in 50% yield (5 mol% of Grubbs II catalyst, then Oxone).^[^ ^28^ ^]^ Subsequent DMP (Dess‐Martin Periodinane) oxidation of the *α*‐hydroxyketone in **20** furnished the corresponding cycloheptanedione **15**, which, upon Wittig olefination with the phosphonium salt **16** (toluene, Et_3_N) delivered a mixture of (*E*)‐**21** and (*Z*)‐**21** in a ratio of 10:1. Notably, the *E*/*Z* selectivity decreased to 4:1 when the olefination reaction was conducted in CH_2_Cl_2_. The observed high *E*‐selectivity is consistent with the fact that the ylide generated from phosphonium salt **16** is a stabilized one.

**Scheme 2:**
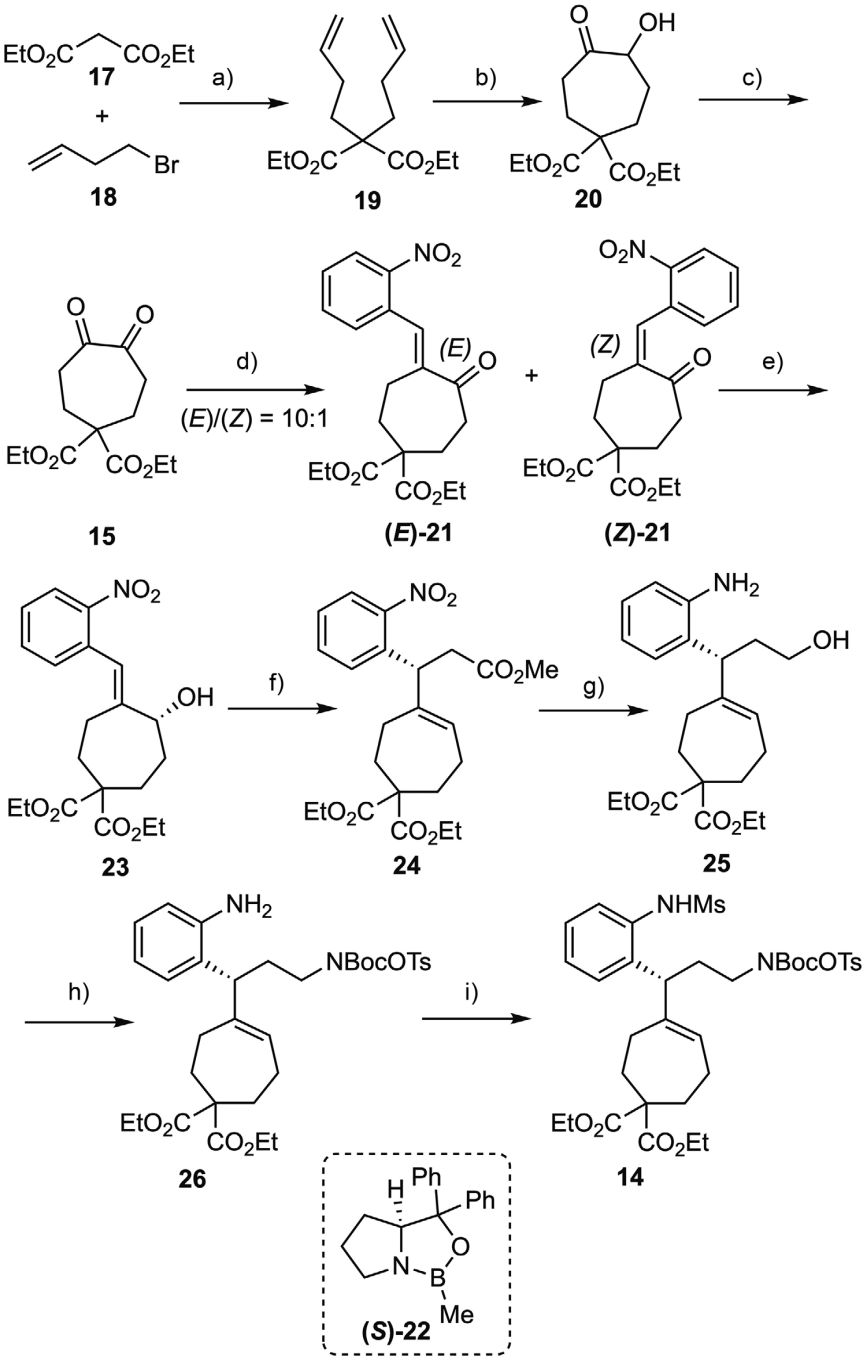
Synthesis of enantioenriched cycloheptene derivative. Reagents and conditions: a) **17** (1.0 equiv.), NaH (2.4 equiv.), **18** (3.0 equiv.), DMF, RT, 48 h, 97%; b) Grubbs II (5.0 mol%), EtOAc, RT, 12 h, then Oxone (5.0 equiv.), NaHCO_3_ (2.5 equiv.), EtOAc/MeCN/H_2_O (v/v/v = 6:6:1), 0 °C, 30 min, 50%; c) DMP (1.1 equiv.), DCM, RT, 2 h; d) **16** (1.6 equiv.), NEt_3_ (1.5 equiv.), toluene, 0 °C to RT, 18 h, 41% over 2 steps, (*E*):(*Z*) = 10:1; e) **(*S*)‐22** (50 mol%), BH_3_·THF (1.1 equiv.), THF, ‐20 °C, 1 h, 99%, 97% *ee*; f) PivOH (20 mol%), MeC(OMe)_3_, 80 °C, 3 h, then 120 °C, 4 h, 61%, 97% *ee*; g) DIBAL‐H (2.0 equiv.), THF, ‐10 °C, 1 h, RT, 1 h, then Zn (14.0 equiv.), NH_4_Cl (14.0 equiv.), MeOH, RT, 2 h, 78%; h) BocNHOTs (1.5 equiv), DIAD (1.2 equiv.), PPh_3_ (1.5 equiv.), THF, 0 °C, 1 h, RT, 2 h, 62%; i) MsCl (3.0 equiv.), pyridine (4.0 equiv.), DCM, RT, 3 h, 97%. DIBAL‐H = diisobutylaluminium hydride, DIAD = diisopropyl azodicarboxylate, MsCl = methanesulfonyl chloride.

Enantioselective reduction of the ketone was then carried out separately on the isomerically pure olefins (*E*)‐**21** and (*Z*)‐**21**. In the presence of Corey‐Bakshi‐Shibata (CBS) catalyst (*S*)‐**22** (50 mol%) and BH_3_·THF (1.1 equiv) at ‐20 °C, reduction of (*E*)‐**21** furnished (*R*)‐**23** in 99% yield with 97% *ee*.^[^ ^29^, ^30^, ^31^ ^]^ The absolute configuration was assigned based on the established stereochemical model of the CBS reduction and later confirmed by X‐ray crystallographic analysis of an advanced intermediate. The *ee* of **23** reduced to 55% when 10 mol% of the (*S*)‐**22** was used, likely due to the competitive background reduction of (*E*)‐**21** by BH_3_·THF. Interestingly, reduction of (*Z*)‐**21** proceeded much more slowly under the same conditions affording racemic allylic alcohol, presumably due to steric hindrance around the carbonyl oxygen in (*Z*)‐**21**. This observation prompted us to examine the enantioselective reduction of the *E*/*Z* mixture of **21**. Remarkably, (*R*)‐**23** was isolated in 97% *ee*, along with recovered (*Z*)‐**21**. The facile separation of (*R*)‐**23** from (*Z*)‐**21** made this approach particularly attractive, as it obviates the need for tedious chromatographic separation of the individual *E*/*Z* isomers. Overall, (*R*)‐**23** was isolated in 37% yield from *α*‐hydroxyketone **20**.

Treatment of (*R*)‐**23** with a catalytic amount of pivalic acid in trimethyl orthoacetate (80 °C, 3 h, then 120 °C, 4 h) afforded, in a stereospecific manner, the *β*‐substituted *γ*,*δ*‐unsaturated ester (*R*)‐**24** (61%, 97% *ee*) via Johnson‐Claisen [3,3]‐sigmatropic rearrangement of the corresponding ketene acetal intermediate.^[^ ^32^, ^33^ ^]^ One‐pot selective reduction of methyl ester in the presence of the diethylmalonate unit with DIBAL‐H (diisobutylaluminium hydride, 2.0 equiv., ‐10 °C), followed by reduction of nitro to amino group (Zn powder, NH_4_Cl, MeOH) converted **24** into amino alcohol **25** in 78% yield. The Mitsunobu reaction of **25** with commercially available aminating reagent NHBocOTs under standard conditions afforded **26**, whose amino group was subsequently sulfonylated to give compound **14**. It is noteworthy that all those transformations were performed on gram‐scale.

Compound **14** was designed as a precursor for the subsequent bis‐cyclizative diamination of alkene, leveraging the elegant method recently developed by Bower and co‐workers.^[^ ^34^, ^35^, ^36^, ^37^ ^]^ Gratifyingly, stirring a trifluoroethanol solution of **14** (*c* 0.1 M) in the presence of 4 equivalents of trifluoroacetic acid at 75°C afforded two products **13** and **29** in yields of 48% and 52%, respectively (Scheme 3). The desired tetracyclic compound **13** arose from a regioselective 5‐*exo*‐tet ring opening of aziridinium intermediate **27** by the tethered sulfonamide. In contrast, for the diastereomeric aziridinium **28**, the same cyclization was geometrically impossible due to the *cis* relative stereochemistry between the C─Ar and C─N bonds. Instead, one of the two ethoxycarbonyl groups, positioned *trans* to the C─N bond, opened the aziridinium via an S_N_2 process to afford the bridged lactone **29**, whose structure was confirmed by X‐ray crystallographic analysis.^[^ ^38^ ^]^ This structural elucidation also corroborated the absolute configuration of the allylic alcohol **23** (*cf* Scheme 2), initially assigned based on the empirical CBS reduction model. Treatment of a solution of **13** with AlMe_3_ then triggered an intramolecular amidation reaction to deliver the bridged lactam **30**, accompanied by desymmetrization at the C20 prochiral center. This transformation completed the construction of the cage‐like pentacyclic skeleton of melonine (**1**).

**Scheme 3:**
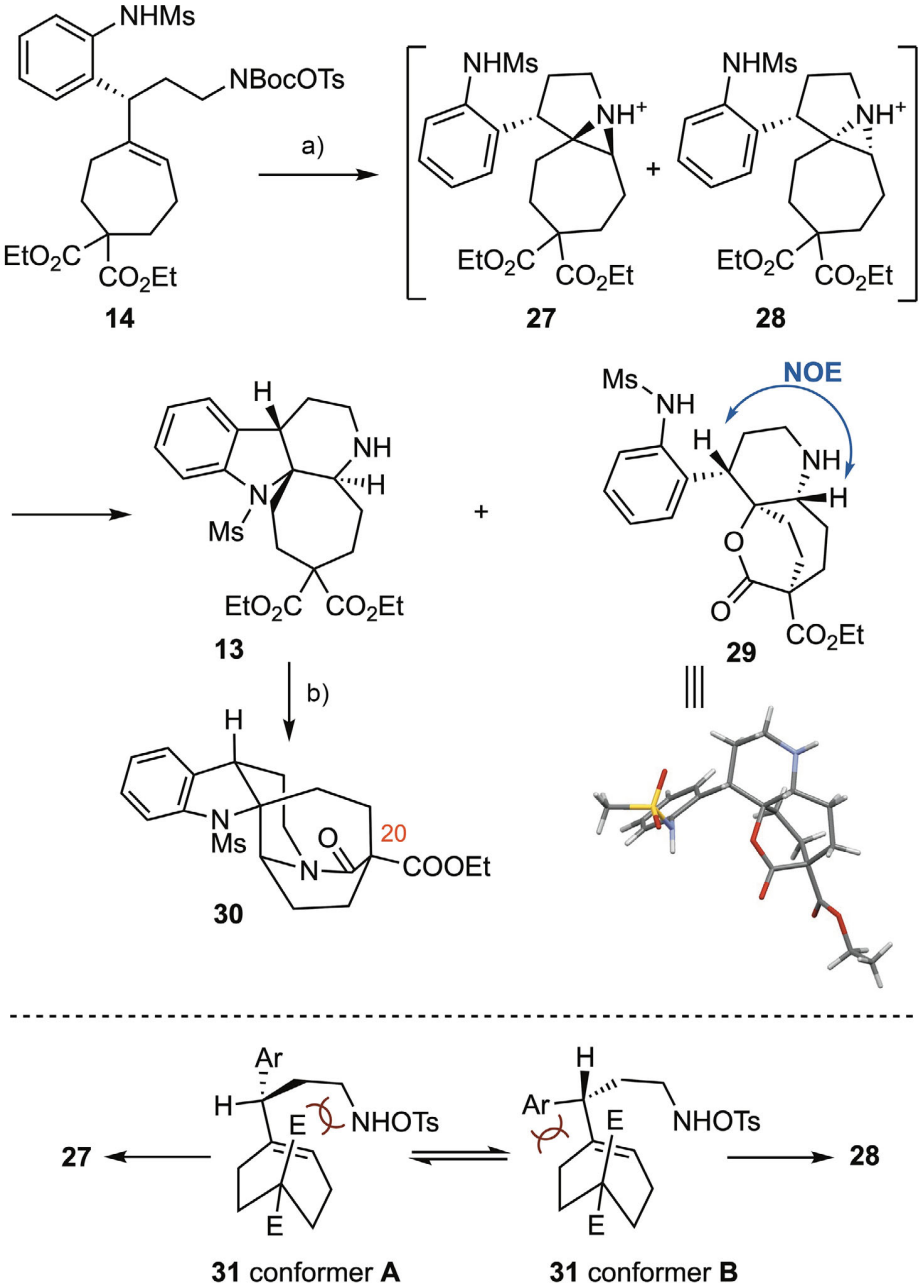
Bis‐cyclizative diamination of cycloheptene. Reagents and conditions: a) TFA (4.0 equiv.), TFE, 75 °C, 18 h, **13**, 48% and **29**, 52%; b) AlMe_3_ (1.03 equiv.), toluene, RT, 1 h, 40 °C, 3 h, 74%. TFA = trifluoroacetic acid, TFE = trifluoroethanol.

Bower has demonstrated that aziridination of alkene bearing an allylic stereocenter proceeds with high diastereoselectivity.^[^ ^34^, ^35^, ^36^, ^37^ ^]^ The lack of diastereoselectivity in our case likely stems from the conformational properties of compound **31**. Two conformers arising from rotation of the Csp^2^─Csp^3^ bond in **31** could account for the formation of two diastereomeric aziridines. Conformer **A**, which minimizes the A^1,2^‐strain, suffers from steric repulsion between the axial ethoxycarbonyl group and the incoming 2‐aminoethyl group. Conformer **B**, although experiencing greater A^1,2^‐strain, avoids this clash during aziridination from the *re*‐face of the double bond. The absence of a strong conformational preference thus resulted in the observed lack of diastereoselectivity, which was nonetheless offset by the excellent yield of this transformation.

The completion of the total synthesis of (+)‐melonine (**1**) and its *N_4_*‐oxide **2** is summarized in Scheme 4. Simultaneous reduction of the ester, amide and the sulfonamide functionalities in **30** with LiAlH_4_ afforded **32** in 79% yield.^[^ ^38^ ^]^ However, all attempts to homologate the C20‐hydroxymethyl substituent were unsuccessful, presumably due to interferences from the two amino groups. The sequence was therefore slightly modified. Chemoselective reduction of the ester in **30** with LiBH_4_, leaving the amide and sulfonamide groups intact, furnished alcohol **33**. Oxidation of **33** under Ley's conditions followed by a one‐pot methylenation of the aldehyde with Matsubara's reagent^[^ ^39^, ^40^, ^41^ ^]^ and hydrogenation of the resulting alkene using Wilkinson's catalyst^[^ ^42^, ^43^ ^]^ under a hydrogen atmosphere provided **34** in 72% yield over two steps.^[^ ^44^ ^]^ Finally, one‐pot reduction of the amide and *N*‐mesyl groups with Red‐Al in toluene afforded (+)‐melonine (**1**) in 95% yield. Oxidation of **1** with *m*‐CPBA in CHCl_3_ proceeded smoothly to afford the corresponding (+)‐*N_4_*‐oxy melonine (**2**) in quantitative yield (30 mg scale). A single batch execution of the entire synthetic sequence delivered 165 mg of the natural product **1**, underscoring the overall efficiency of our approach.

**Scheme 4:**
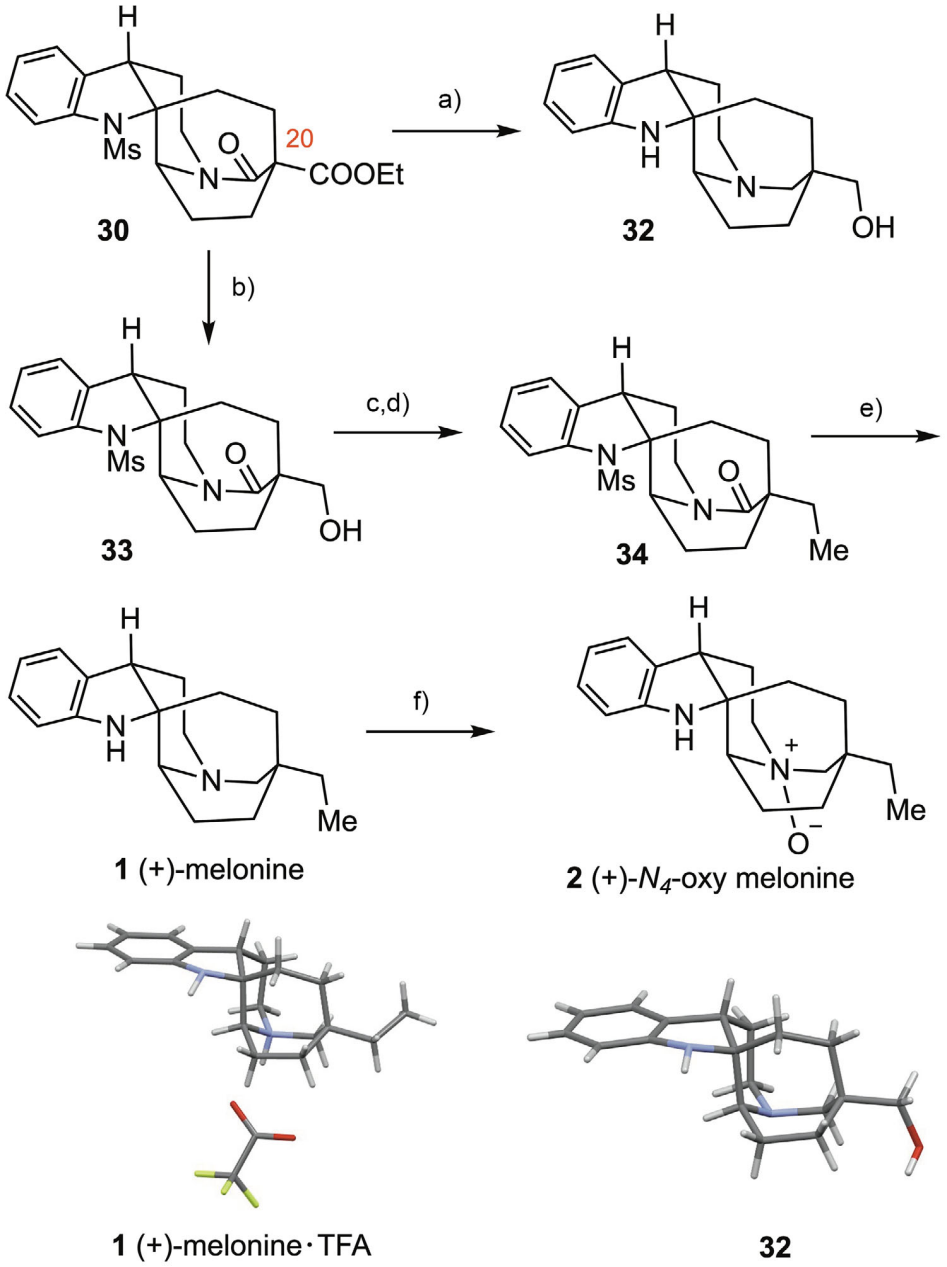
Endgame: Synthesis of melonine and its *N_4_*‐oxide. Reagents and conditions: a) LAH (5.0 equiv.), THF, RT, 1 h, then reflux, 5 h, 79%; b) LiBH_4_ (8.0 equiv.), MeOH, Et_2_O, RT, 2 h, 92%; c) TPAP (20 mol%), NMO (3.0 equiv.), 4 Å MS (1.0 g/mmol), DCM, RT, 2 h; d) CH_2_(ZnI)_2_ (4.0 equiv.), THF, RT, 4 h, then RhCl(PPh_3_)_3_ (10 mol%), H_2_ (balloon), THF, RT, 16 h, 72% over 2 steps; e) Red‐Al (10.0 equiv.), toluene, RT, 1 h, then 95 °C, 3 h, 95%; f) *m‐*CPBA (1.1 equiv.), CHCl_3_, 0 °C, 15 min, 99%. TPAP = tetrapropylammonium perruthenate, NMO = *N*‐methylmorpholine *N*‐oxide, Red‐Al = sodium bis(2‐methoxyethoxy)aluminium hydride, *m‐*CPBA =* m*‐chloroperoxybenzoic acid.

The spectroscopic data of both **1** and **2** match well with those described in the literature. Finally, the X‐ray crystallographic analysis of the TFA salt of **1** unambiguously confirmed not only the structure of (+)‐melonine (**1**), but also its absolute configuration.^[^ ^38^ ^]^

In summary, we accomplished the first enantioselective total synthesis of (+)‐melonine (**1**) in 15 steps in the longest linear sequence from ethyl malonate. The synthesis featured a) a highly enantioselective CBS reduction followed by stereospecific Johnson–Claisen rearrangement to construct an enantioenriched *β*‐substituted *γ*,*δ*‐unsaturated ester; b) Bower's bis‐cyclizative diamination of alkene, enabling the transformation of a functionalized cycloheptene into the tetracyclic motif of the natural products; and c) an AlMe_3_‐mediated lactamization accompanied by desymmetrization of the C20 prochiral center.

## Supporting Information

The authors have cited additional references within the Supporting Information.^[^ ^45^, ^46^, ^47^, ^48^, ^49^, ^50^, ^51^, ^52^, ^53^ ^]^

## Conflict of Interests

The authors declare no conflict of interest.

## Supporting information



Supporting Information

## Data Availability

The data that support the findings of this study are available in the Supporting Information of this article.
