# Supplementary material for: Total Synthesis of (+)‐Melonine and (+)‐N4 ‐Oxy Melonine Enabled by an Intramolecular Alkene Diamination Reaction
Source: Angew Chem Int Ed Engl. 2026 Jan 9;65(7):e8101956. doi: 10.1002/anie.8101956 (PMC12887629; doi:10.1002/anie.8101956)
Supplement: Supplementary file 1 — Supporting Information [file ANIE-65-e8101956-s001.pdf]

Supporting information

Total Synthesis of (+)-Melonine and (+)-N_4-Oxy Melonine Enabled by an Intramolecular Alkene Diamination Reaction

Vincent Goëlo, Qian Wang and Jieping Zhu*

Laboratory of Synthesis and Natural Products (LSPN), Institute of Chemical Sciences and Engineering, Ecole Polytechnique Fédérale de Lausanne, EPFL-SB-ISIC-LSPN, BCH 5304, 1015 Lausanne (Switzerland)

*Correspondence to: jieping.zhu@epfl.ch

Contents

1) General information 2

2) Initial strategy attempted: access to the biosynthetic iminium ion 3

A) Synthetic access to the biosynthetic iminium and transannular cyclization attempts 3

B) Experimental procedures and characterization data of key advanced intermediates 7

3) Experimental procedures and characterization data 9

A) Starting material preparation 9

B) Selective formation and enantioselective reduction of enone (*E*)-21 15

C) Stereospecific Johnson-Claisen rearrangement and key intermediate synthesis 24

D) Two-step key sequence towards melonine core 32

E) End-game of the total synthesis of (+)-melonine 36

4) References 50

5) Copies of NMR spectra 51

6) X-Ray structural analysis 114

1) General information

Reagents and solvents were purchased from commercial sources and preserved under argon. More sensitive compounds were stored in a desiccator or in the glovebox if required. Reagents were used as received without further purification unless otherwise noted. All reactions were performed under nitrogen (or argon) and stirring unless otherwise noted. When needed, glassware was dried overnight in an oven (150 °C). Solvents indicated as dried were either purchased as such, distilled prior to use, or dried by a passage through a column of anhydrous alumina or copper using a Puresolv MD 5 from Innovative Technology Inc., based on the Grubbs' design. Flash column chromatography was performed using Silicycle SiliaFlash® P60 230-400 mesh. Reactions were monitored using Merck Kieselgel 60F254 aluminum. TLC's were revealed by UV fluorescence (254 nm) then with either $KMnO_4$ or phosphomolybdic acid. NMR spectra were recorded on AV2 400 MHz, AV2 500 MHz, AV2 600 MHz or AV2 800 MHz Brüker spectrometers at room temperature. ^{1}H frequencies are at 400 MHz, 500 MHz, 600 MHz and 800 MHz, ^{13}C frequencies are at 101 MHz, 126 MHz, 151 MHz, and 201 MHz. Chemical shifts (δ) were reported in parts per million (ppm) relative to residual solvent peaks rounded to the nearest 0.01 for proton and 0.1 for carbon (ref: $CDCl_3$ [^{1}H: 7.26, ^{13}C: 77.16 ppm]; CD_3OD [^{1}H: 3.31, ^{13}C: 49.00 ppm]; CD_3CN [^{1}H: 1.94, ^{13}C: 1.32 and 118.26 ppm]). Coupling constants (J) were reported in Hz to the nearest 0.1 Hz. Peak multiplicity was indicated as follows: s (singlet), d (doublet), t (triplet), q (quartet), p (quintet), m (multiplet) and br (broad). Attribution of peaks was done using the multiplicities and integrals of the peaks. COSY, HSQC, HMBC and 1D NOE experiments were used when needed to confirm the assignments. IR spectra were recorded in a Jasco FT/IR-4X spectrometer outfitted with a PIKE technology MIRacleTM ATR accessory as neat films compressed onto a Zinc Selenide window. The spectra are reported in cm^{-1}. Abbreviations used are: w (weak), m (medium), s (strong) and br (broad). The accurate masses were measured by the mass spectrometry service of the EPFL by ESI-TOF using a QTOF Ultima from Waters. Melting points were determined using a Stuart SMP30. Specific optical rotations $[\alpha]_D$ were measured with a Jasco P-2000 polarimeter (589 nm). Enantiomeric excesses were determined with a 1260 Infinity II SFC System from Agilent using chiral stationary phase columns by comparing the samples with the corresponding racemic samples, column and elution details specified in each entry.

List of Abbreviations:

TLC – thin-layer chromatography; FCC – flash column chromatography.

2) Initial strategy attempted: access to the biosynthetic iminium ion

A) Synthetic access to the biosynthetic iminium and transannular cyclization attempts

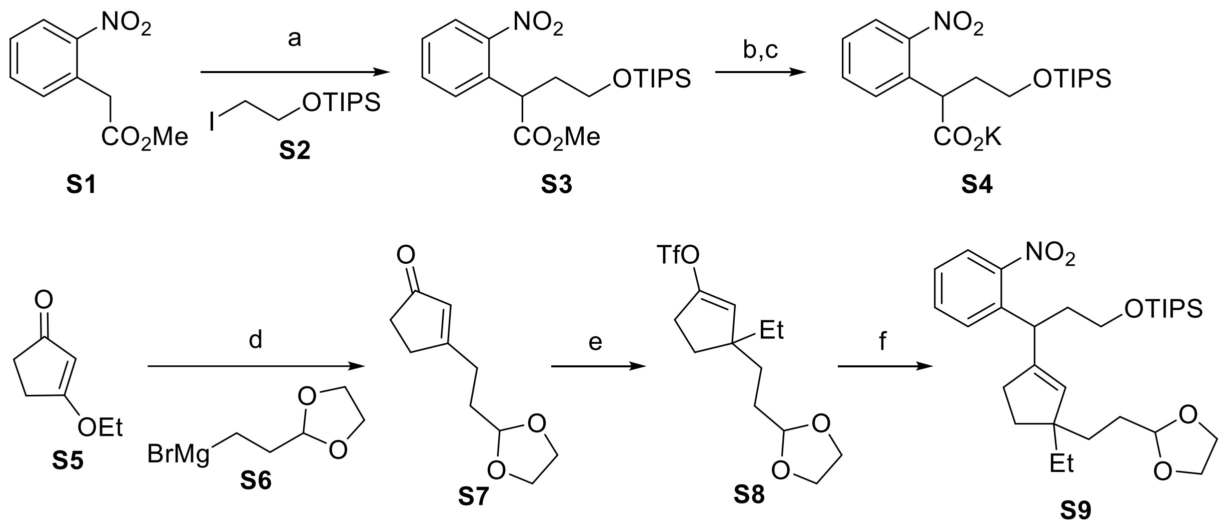

Scheme S1. Preparation of the starting materials and fragments cross coupling.

Reagents and conditions. a) **S2** (1.2 equiv), Cs_2CO_3 (1.5 equiv), DMF, 60 °C, 16 h, 94%; b) KOH (5.0 equiv), H_2O/MeOH/THF (1:1:6), rt, 6 h, 92%; c) *t*-BuOK (1.0 equiv), EtOH, rt, 1 h, quant.; d) **S6** (1.2 equiv), THF, 60 °C, 3 h, then acidic work up, 81%; e) EtMgBr (4.0 equiv), CuBr·DMS (2.0 equiv), THF, -40 °C, 3 h, then Comins' reagent (2.0 equiv), rt, 16 h, 70%; f) **S8** (1.0 equiv), **S4** (1.7 equiv), $[Pd(allyl)Cl]_2$ (5 mol%), XPhos (15 mol%), DMF/diglyme (1:10), 100 °C, 3 h, 86%, dr 1:1.

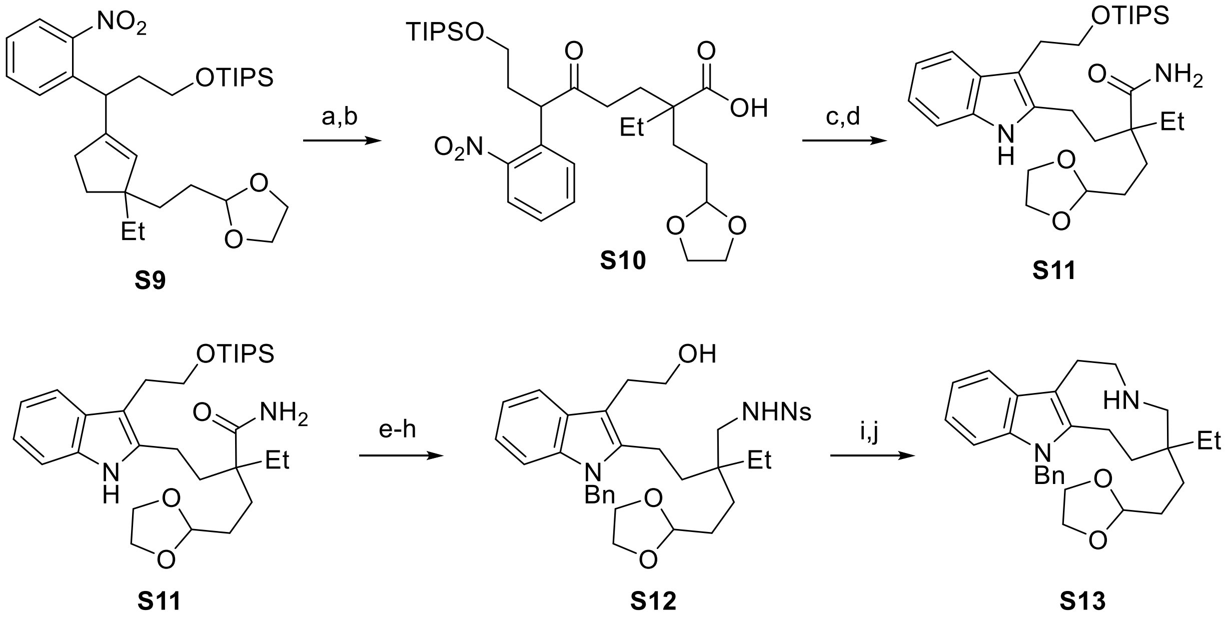

Scheme S2. Formation of the 9-membered ing precursor of the target iminium.

Reagents and conditions. a) O_3 bubbling, Sudan III dye (traces), DCM, -78 °C, then PPh_3 (2.0 equiv), rt, 2 h; b) 2-methylbut-2-ene (20.0 equiv), NaH_2PO_4 (10.0 equiv), $NaClO_2$ (3.0 equiv), *t*-BuOH/H_2O (3:2), rt, 1.5 h, 73% over 2 steps; c) Zn (70.0 equiv), $CaCl_2$ (7.0 equiv), MeOH, reflux, 2 h, quant.; d) HATU (2.0 equiv), DIPEA (30.0 equiv), NH_4Cl (28.0 equiv), DMF, rt, 1 h, 70%; e) NaH (3.0 equiv), BnBr (1.0 equiv), DMF, rt, 4 h, 67%; f) $LiAlH_4$ (6.0 equiv), THF, reflux, 16 h; g) *o*-NsCl (1.2 equiv), NEt_3 (2.0 equiv), DCM, rt, 4 h, 60% over 2 steps; h) TBAF (1.2 equiv), THF, rt, 3 h, 81%; i) PPh_3 (2.0 equiv), DIAD (syringe pump addition, 1.5 equiv), neopentyl alcohol (40 mol%), toluene, rt, 4 h, 98%; j) PhSH (2.0 equiv), Cs_2CO_3 (2.0 equiv), MeCN, rt, 2 h, 76%.

Our first synthetic strategy targeting the proposed biosynthetic iminium started with the preparation of the cyclopentene **S9** *via* a Pd-catalyzed decarboxylative cross coupling between the two fragments **S4** and **S8** (Scheme S1). This cyclopentene **S9** was successfully opened *via* ozonolysis in the presence of Sudan III dye as color-indicator to monitor the reaction and avoid acetal deprotection (Scheme S2). Pinnick oxidation of the resulting aldehyde provided **S10**, which was subsequently converted to the indole amide **S11** and further to compound **S12** after functional group interconversions. Fukuyama-Mitsunobu cyclization of precursor **S12** afforded the desired 9-membered ring compound **S13** after removal of the nosyl group.

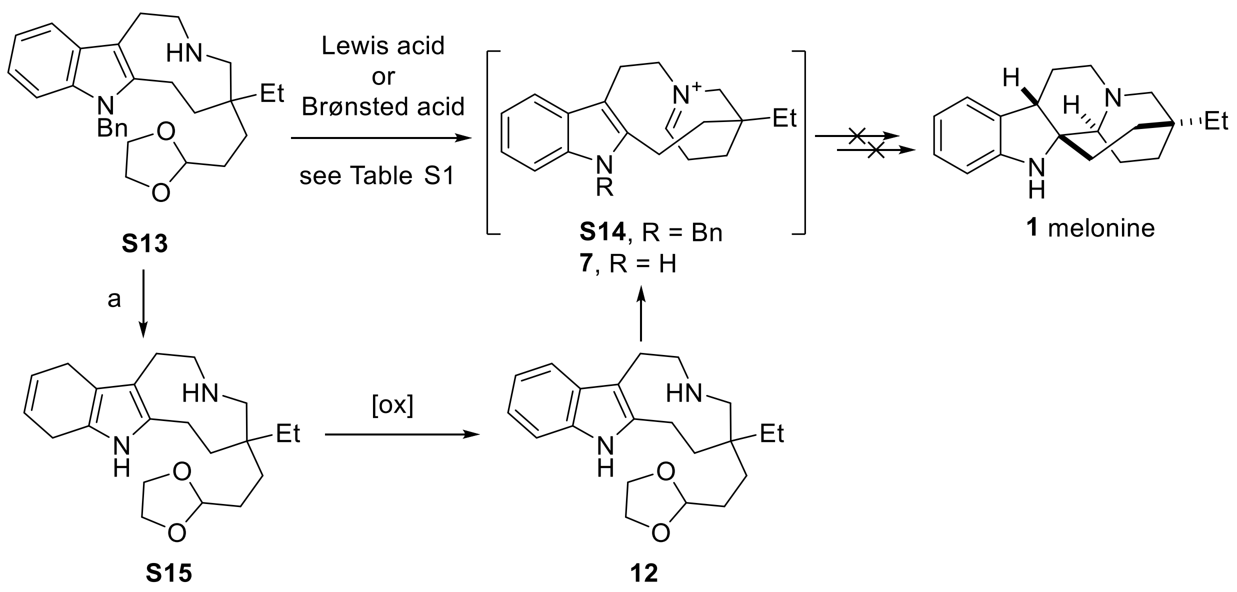

Scheme S3. End-game investigation: iminium formation and failed cyclization towards melonine. Reagents and conditions. a) Li, NH_3, THF, -78 °C, 1 h, then MeOH, -78 °C to rt.

The endgame involving iminium formation and biomimetic cyclization towards melonine was investigated starting with the *N*-benzyl protected indole **S13** (Scheme S3). Upon treatment of **S13** with HCl, formation of iminium **S14** was cleanly observed after solvent evaporation and analysis of the crude mixture (Table S1, entry 1). Nevertheless, no cyclized product was observed. The *N*-benzyl protecting group could be successfully removed under Birch reduction conditions (Li, NH_3, THF, -78 °C). Upon addition of methanol at the end of the deprotection, further reduction

of the indole moiety provided the corresponding 4,7-dihydroindole product **S15**.[1,2] Attempts to use this reduced indole intermediate, leveraging its pyrrole-like C2 nucleophilicity as well as its oxidation state that is like the one in melonine, all failed in producing the desired cyclization. Notably, the rearomatization process of **S15** to **12**, which already appeared highly favorable under air and neutral conditions, was accelerated under acidic conditions. Representative conditions attempted for the one-pot conversion of **S13**, **S15** or **12** into the desired melonine skeleton are depicted in Table S1.

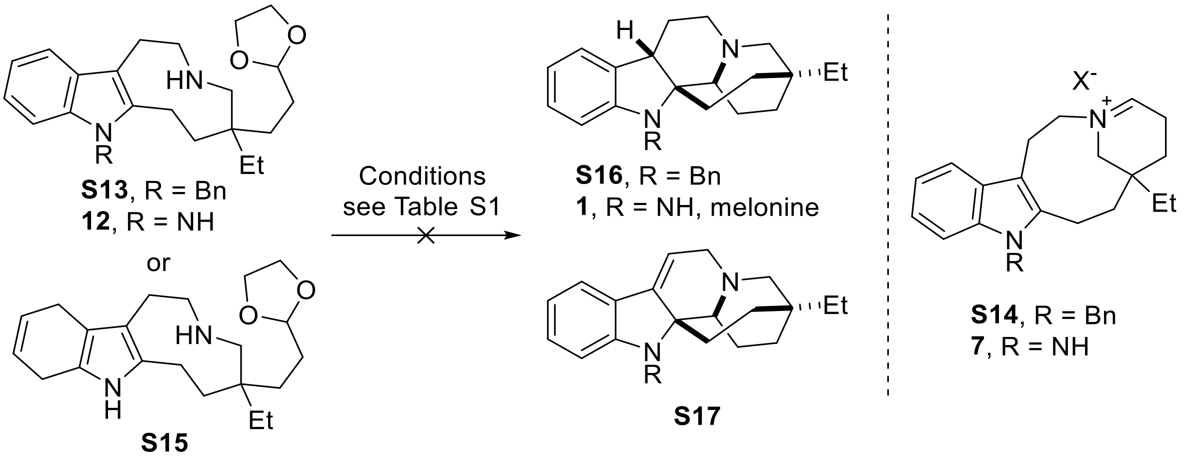

Entry	Conditions	Results
**1**	1 N HCl, H_2O, MeOH, rt	**S14 or 7**
**2**	Zn, $CuSO_4$, AcOH, reflux	Decomp.
**3**	SmI_2, $BF_3 \cdot Et_2O$, MeCN, rt	**No acetal deprotection**
**4**	$Ce(OTf)_3$, MeCN, 85 °C	**S14 or 7**
**5**	$In(OTf)_3$, glovebox, dry MeCN, 85 °C	**S14 or 7**
**6**	$Bi(NO_3)_3 \cdot 5H_2O$, MeCN, 85 °C	Decomp.
**7**	TMSOTf, 2,6-lutidine, DCM, 0 °C, then H_2O	Decomp.
**8**	$BF_3 \cdot Et_2O$, DCM, -78 °C to rt	Decomp.
**9**	TFA, toluene, 5 Å MS	Decomp.
**10**	TFA, *n*-butanol, 130 °C	Decomp.
**11**	$PTSA \cdot H_2O$, NMP, 130 °C	Decomp.
**12**	AcOH, reflux	Decomp.
**13**	$HBF_4 \cdot Et_2O$, MeCN, 0 °C to rt	Decomp.

Table S1. Representative conditions attempted for the one pot iminium formation/transannular cyclization from S13, S15 and 12.

Applying the reductive conditions developed by Le Men and co-workers in their rearrangement of indolenine using Zn, $CuSO_4 \cdot 5H_2O$ in refluxing AcOH (entry 2),[3] similarly to

Boger *et al.*[4] with their related system, no desired cyclization occurred, leading to complex mixtures only. Also, inspired by Boger's strategy (entry 3), we attempted a reductive protocol combining $BF_3 \cdot Et_2O/SmI_2$ which led only to unknown byproduct mixtures, without acetal deprotection. Then a variety of Lewis acids (entries 4-8) and Brønsted acids (entries 9-13) were investigated in diverse types of solvent and temperatures, but without success in accessing cyclized products **S16**, **S17** or **1**. While these conditions mainly le to complex mixtures and decomposition of the substrates, iminiums **S14** or **7** could be observed when employing Lewis acidic $Ce(OTf)_3$ or $In(OTf)_3$ (entries 4-5).

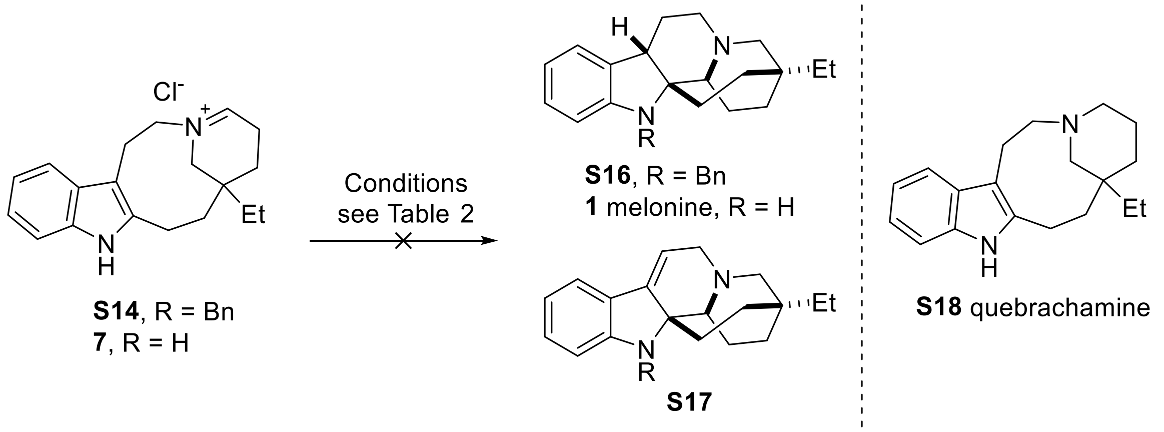

Entry	Conditions	Results
**1**	Zn, $CuSO_4$, AcOH, reflux	Decomp.
**2**	SmI_2, THF, rt	Decomp.
**3**	SmI_2, $BF_3 \cdot Et_2O$, MeCN, rt	Decomp.
**4**	K_2CO_3, MeOH, 65 °C	Decomp.
**5**	$NaBH_4$, MeOH, rt	**S18 observed**

Table S2. Attempts to promote transannular cyclization from the iminium intermediate.

Still encouraged by the formation of the biosynthetic iminium, further investigations to promote the transannular cyclization were explored (Table S2). From iminiums **S14** and **7**, once again Le Men's reductive conditions were applied without success (entry 1). Next, SmI_2 was used to generate the corresponding α-amino radical, aiming for a transannular radical cyclization on the favored C2-position of the indole. However, with or without addition $BF_3 \cdot Et2O$, only complex mixtures were observed, without any trace of desired cyclized products (entries 2-3). In presence of weak base, the crude iminium was completely decomposed (entry 4). As expected, reduction of the iminium **S7**, led to the observation of quebrachamine **(S18)** (entry 5), thus confirming the generation and structural integrity of the expected iminium intermediate. It was noteworthy that in 2024, Wang's group reported the total synthesis of quebrachamine **(S18)** employing a similar 9-

membered ring formation through a Fukuyama-Mitsunobu cyclization.[5] The observed lack of productive cyclization suggested that this biosynthetic iminium **7**, in our hands, might stem from conformational strain preventing proper spatial alignment of the iminium with the C2 position of the indole. Indeed, the 9,6-fused ring system, with a one-carbon bridge may introduce significant strain to reach the desired conformation, requiring the three-carbon unit bearing the iminium group to fold inside the medium-sized ring, a geometry that may be energetically inaccessible under laboratory conditions.

B) Experimental procedures and characterization data of key advanced intermediates

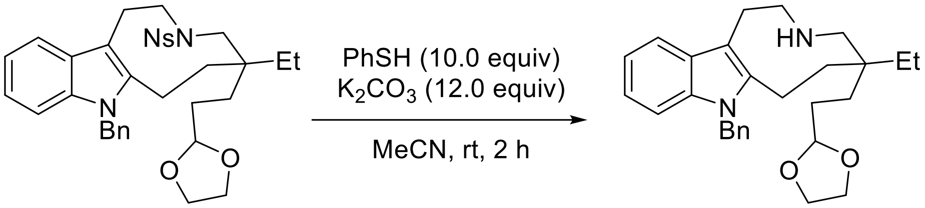

S19 **S13**

K_2CO_3 (322 mg, 2.33 mmol, 12.0 equiv) was added at rt to a solution of cyclic nosylamine **S19** (120 mg, 194 µmol, 1.0 equiv) and thiophenol (198 µL, 1.94 mmol, 10.0 equiv) in dry MeCN (1.9 mL) and the mixture was stirred at rt for 2 h. Water was added and it was extracted three times with EtOAc. The combined organic layers were washed with brine, dried over $MgSO_4$, filtered and concentrated under vacuum. FCC purification (SiO_2, 0/1 to 1/4 MeOH/EtOAc) afforded the corresponding secondary amine **S13** (64 mg, 148 µmol, 76% yield) as a colorless oil.

5-(2-(1,3-dioxolan-2-yl)ethyl)-8-benzyl-5-ethyl-1,2,3,4,5,6,7,8-octahydroazonino[5,4-b]indole (S13)

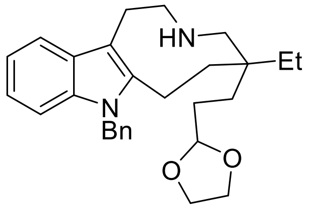

S13

Colorless oil.

^{1}H NMR (400 MHz, $CDCl_3$) δ 7.50 (d, J = 8.0 Hz, 1H), 7.32 – 7.20 (m, 4H), 7.17 – 7.08 (m, 2H), 6.97 (d, J = 7.3 Hz, 2H), 5.32 (s, 2H), 4.80 (t, J = 4.5 Hz, 1H), 3.99 – 3.90 (m, 2H), 3.88 – 3.79 (m, 2H), 3.09 (s, 2H), 2.90 (t, J = 5.8 Hz, 2H), 2.75 (s, 2H), 2.63 (t, J = 5.5 Hz, 2H), 1.69 – 1.15 (m, 8H), 0.79 (t, J = 7.4 Hz, 3H).

^{13}C NMR (101 MHz, $CDCl_3$) δ 140.7, 138.0, 136.9, 128.9, 127.5, 127.3, 126.0, 121.6, 119.6, 117.8, 109.5, 106.7, 104.8, 65.0, 65.0, 48.3, 46.7, 45.7, 39.7, 35.1, 28.6, 27.9, 27.8, 20.6, 17.2, 7.7.

HRMS (ESI/QTOF) m/z: $[M + H]^+$ Calcd for $C_{28}H_{37}N_2O_2^+$ 433.2850; Found 433.2856.

IR (ν_{max}, cm^{-1}) 2930 (m), 2876 (m), 1466 (m), 1357 (m), 1130 (m), 1031 (m), 737 (s).

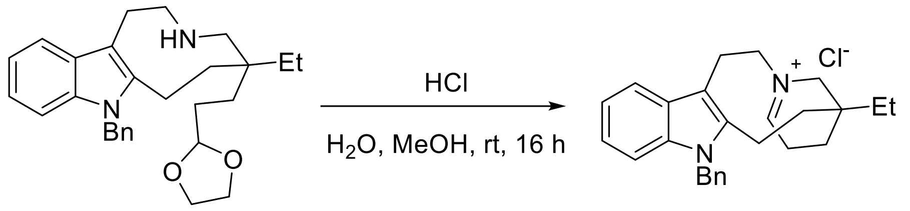

S13 **S14**

A solution of compound **S13** (5.0 mg, 11.6 μmol, 1.0 equiv) in a 1:1 mixture of 1 N aqueous HCl (1 mL) and MeOH (1 mL), was stirred at rt for 16 h. The mixture was then concentrated under reduced pressure to afford crude iminium **S14** as a white solid.

10-benzyl-7-ethyl-1,2,5,6,7,8,9,10-octahydro-3,7-methano[1]azacycloundecino[5,4-b]indol-3-ium chloride (S14)

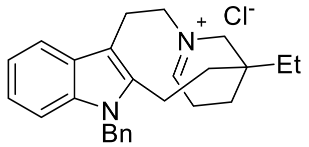

S14

White solid.

^{1}H NMR (800 MHz, CD_3OD) δ 8.14 (s, 1H), 7.63 – 7.58 (m, 1H), 7.32 – 7.19 (m, 4H), 7.17 – 7.08 (m, 2H), 6.94 – 6.90 (m, 2H), 5.45 (d, *J* = 17.3 Hz, 1H), 5.36 (d, *J* = 17.3 Hz, 1H), 4.37 (dd, *J* = 15.5, 2.9 Hz, 1H), 4.20 (d, *J* = 12.8 Hz, 1H), 3.98 (td, *J* = 12.3, 4.0 Hz, 1H), 3.67 – 3.58 (m, 1H), 3.60 (s, 2H), 3.41 – 3.32 (m, 2H), 2.90 – 2.72 (m, 2H), 1.88 (ddd, *J* = 15.7, 10.3, 2.0 Hz, 1H), 1.64 (dt, *J* = 13.9, 7.9 Hz, 1H), 1.53 – 1.41 (m, 2H), 1.41 – 1.30 (m, 1H), 1.05 (dd, *J* = 15.7, 7.7 Hz, 1H), 0.95 (t, *J* = 7.5 Hz, 3H).

^{13}C NMR (201 MHz, CD_3OD) δ 181.4, 141.9, 139.7, 138.6, 129.8, 128.4, 127.9, 127.1, 123.1, 120.9, 118.7, 110.9, 105.7, 64.3, 61.9, 54.3, 47.3, 37.0, 34.1, 33.0, 28.5, 22.1, 18.9, 7.8.

HRMS (ESI/QTOF) m/z: $[M]^+$ Calcd for $C_{26}H_{31}N_2^+$ 371.2482; Found 371.2486.

3) Experimental procedures and characterization data

A) Starting material preparation

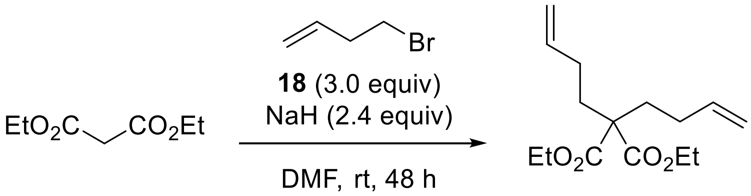

17 19

At 0 °C, diethyl malonate (**17**) (30.5 mL, 200 mmol, 1.0 equiv) was slowly added to a suspension of NaH (60% wt in mineral oil, 1.2 equiv) in dry DMF (300 mL) and the mixture was stirred at rt for 45 minutes. 4-Bromobut-1-ene (**18**) (24.4 mL, 240 mmol, 1.2 equiv) was then slowly added and the reaction mixture was stirred at rt for 18 h. The mixture was subsequently cooled to 0 °C followed by the addition of NaH (60% wt in mineral oil, 1.2 equiv). After stirring at rt for 45 min, 4-bromobut-1-ene (30.4 mL, 300 mmol, 1.5 equiv) was added, and the mixture was stirred at rt for an additional 18 h. The reaction mixture was then poured into saturated NH_4Cl solution, diluted with water and extracted three times with Et_2O. The combined organic layers were washed with brine, dried over Na_2SO_4, filtered and concentrated under reduced pressure. The crude product was purified by FCC (SiO_2, 1/6 Et_2O/Hexane) to give dialkylated malonate **19** (52.1 g, 194 mmol, 97%) as a colorless oil.

All characterization data were in full agreement with those reported in the literature.[6]

diethyl 2,2-di(but-3-en-1-yl)malonate (19)

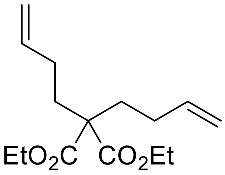

19

Colorless oil.

Rf = 0.42 (1/8 Et_2O/Hexane).

^{1}H NMR (400 MHz, $CDCl_3$) δ 5.84 – 5.72 (m, 2H), 5.03 (dq, J = 17.3, 1.6 Hz, 2H), 4.98-4.94 (m, 2H), 4.18 (q, J = 7.1 Hz, 4H), 2.03 – 1.90 (m, 8H), 1.25 (t, J = 7.1 Hz, 6H).

^{13}C NMR (101 MHz, $CDCl_3$) δ 171.6, 137.7, 115.2, 61.3, 57.2, 31.7, 28.5, 14.2.

HRMS (nanochip-ESI/LTQ-Orbitrap) m/z: $[M + Na]^+$ Calcd for $C_{15}H_{24}NaO_4^+$ 291.1567; Found 291.1562.

IR (ν_{max}, cm^{-1}) 2961 (w), 2927 (w), 2856 (w), 1730 (s), 1454 (w), 1260 (m), 1201 (m), 1142 (m), 1095 (m), 1027 (m), 913 (m), 862 (w), 803 (m), 735 (m), 675 (w), 650 (w).

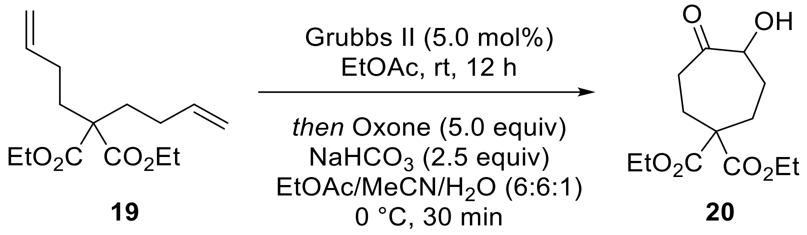

<u>One-pot RCM/oxidation:</u> To a degassed solution of diene **19** (10.7 g, 40.0 mmol, 1.0 equiv) in dry EtOAc (185 mL) was added Grubbs II catalyst (1.7 g, 2.0 mmol, 5.0 mol%). The flask was subjected to three cycles of evacuation and argon backfilling before being stirred at rt for 12 h. Upon completion of the reaction, MeCN (185 mL), H_2O (31 mL) and $NaHCO_3$ (8.4 g, 100 mmol, 2.5 equiv) were added sequentially. Using a cryostat set at -10 °C, the reaction mixture was cooled to an internal temperature of 0 °C. Oxone (123 g, 200 mmol, 5.0 equiv) was added in one portion under vigorous stirring and the reaction mixture was stirred at this temperature for 30 min (careful monitoring of the reaction). The mixture was then diluted with EtOAc (200 mL), and the resulting solids were removed by filtration and washed with EtOAc. The filtrate was then treated with saturated aqueous solutions of $Na_2S_2O_3$ and $NaHCO_3$ (v/v = 1:1, 500 mL) and the aqueous layer was extracted twice more with EtOAc (200 mL). The combined organic layers were washed with brine, dried over Na_2SO_4, filtered and concentrated under reduced pressure. The crude product was purified by FCC (3/2 EtOAc/Hexane) to give α-hydroxyketone **20** (6.5 g, mmol, 50%) with minor α-diketone **15**, as a colorless oil.

All characterization data were in full agreement with those reported in the literature.[7]

diethyl 4-hydroxy-5-oxocycloheptane-1,1-dicarboxylate (20)

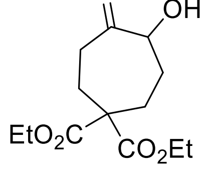

20

Colorless oil.

Rf = 0.53 (2/3 EtOAc/Hexane).

^{1}H NMR (400 MHz, $CDCl_3$) δ 4.27 (dd, *J* = 8.8, 4.1 Hz, 1H), 4.25 – 4.11 (m, 4H), 3.74 (s, 1H), 2.77 (ddd, *J* = 16.9, 8.4, 2.6 Hz, 1H), 2.53 (ddd, *J* = 16.9, 10.9, 2.7 Hz, 1H), 2.39 – 2.29 (m, 2H), 2.18 (ddd, *J* = 15.3, 8.4, 2.7 Hz, 1H), 2.11 – 2.01 (m, 2H), 1.96 – 1.83 (m, 1H), 1.26 (t, *J* = 7.3 Hz, 3H), 1.24 (t, *J* = 7.3 Hz, 3H).

^{13}C NMR (101 MHz, $CDCl_3$) δ 211.9, 171.6, 170.5, 76.6, 61.9, 61.8, 57.1, 35.4, 30.8, 29.0, 28.3, 14.2.

HRMS (nanochip-ESI/LTQ-Orbitrap) m/z: $[M + Na]^+$ Calcd for $C_{13}H_{20}NaO_6^+$ 295.1152; Found 295.1153.

IR (ν_{max}, cm^{-1}) 3455 (w), 2979 (w), 2939 (w), 2903 (w), 1724 (s), 1451 (w), 1391 (w), 1368 (w), 1296 (m), 1236 (s), 1181 (m), 1146 (m), 1094 (m), 1063 (m), 1040 (m), 859 (w).

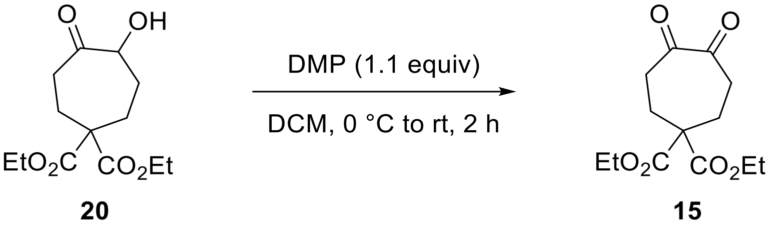

α-Hydroxyketone **20** (7.0 g, 25.7 mmol, 1.0 equiv, containing some *α*-diketone **15**) was dissolved in dry DCM (260 mL) and cooled to 0 °C. DMP (12.0 g, 28.3 mmol, 1.1 equiv) was added portionwise and the reaction mixture was stirred at 0 °C for 15 minutes, then at rt for 2 h. Upon completion, the reaction was quenched by the addition of saturated aqueous solutions of $Na_2S_2O_3$ and $NaHCO_3$ (v/v = 1:1, 400 mL) and stirred at rt for 5 min. The mixture was extracted three times with DCM. The combined organic layers were washed with brine, dried over Na_2SO_4, filtered and concentrated under reduced pressure to give crude *α*-diketone **15** as a deep-yellow oil. The crude product was directly used in the next step without further purification.

diethyl 4,5-dioxocycloheptane-1,1-dicarboxylate (15)

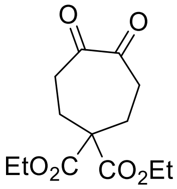

5

Deep-yellow oil.

Rf = 0.35 (2/3 EtOAc/Hexane).

^{1}H NMR (400 MHz, $CDCl_3$) δ 4.24 (q, *J* = 7.0 Hz, 4H), 2.69 – 2.66 (m, 4H), 2.43 – 2.40 (m, 4H), 1.27 (t, *J* = 7.2 Hz, 6H).

^{13}C NMR (101 MHz, $CDCl_3$) δ 203.9, 170.4, 62.2, 56.6, 35.5, 27.0, 14.1.

HRMS (nanochip-ESI/LTQ-Orbitrap) m/z: $[M + H]^+$ Calcd for $C_{13}H_{19}O_6^+$ 271.1176; Found 271.1180.

IR (ν_{max}, cm^{-1}) 2979 (w), 2930 (w), 2857 (w), 1728 (s), 1456 (m), 1294 (m), 1241 (m), 1207 (m), 1186 (m), 1060 (w), 1024 (m), 860 (w), 677 (m), 654 (m).

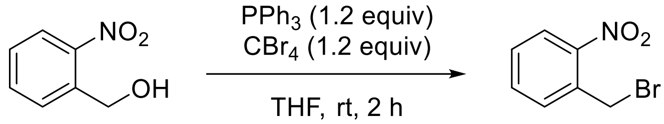

S20 **S21**

2-Nitrobenzyl alcohol (**S20)** (15.3 g, 100 mmol, 1.0 equiv) and PPh_3 (31.6 g, 120 mmol, 1.2 equiv) were dissolved in dry THF (500 mL) and cooled to 0 °C. CBr_4 (39.8 g, 120 mmol, 1.2 equiv) was added portionwise to the solution and the reaction mixture was stirred at rt for 2 h. The solvent was removed under reduced pressure, and the crude product was purified directly by FCC (SiO_2, 1/3 EtOAc/Hexane) to give compound **S21** (21.1 g, 97.7 mmol, 98%) as light-yellow crystals.

All characterization data were in full agreement with those reported in the literature.[8]

1-(bromomethyl)-2-nitrobenzene (S21)

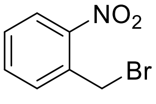

S21

Light-yellow crystals.

Rf = 0.50 (1/6 EtOAc/Hexane).

^{1}H NMR (400 MHz, $CDCl_3$) δ 8.04 (dd, *J* = 8.2, 1.3 Hz, 1H), 7.65 – 7.54 (m, 2H), 7.49 (ddd, *J* = 8.8, 7.1, 1.8 Hz, 1H), 4.83 (s, 2H).

^{13}C NMR (101 MHz, $CDCl_3$) δ 148.1, 133.8, 132.9, 132.7, 129.7, 125.6, 29.0.

HRMS (Sicrit plasma/LTQ-Orbitrap) m/z: $[M + H]^+$ Calcd for $C_7H_7BrNO_2^+$ 215.9655; Found 215.9653.

IR (ν_{max}, cm^{-1}) 3111 (w), 3059 (w), 2855 (w), 1607 (w), 1576 (w), 1519 (s), 1430 (m), 1338 (s), 1311 (m), 1226 (m), 1203 (m), 1116 (w), 858 (m), 792 (m), 749 (m), 696 (m), 663 (m).

Mp = 43 – 45 °C.

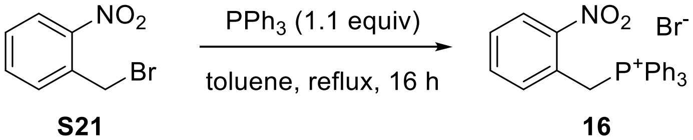

S21 **16**

2-Nitrobenzylbromide (**S21)** (21.1 g, 97.7 mmol, 1.0 equiv) and PPh_3 (28.2 g, 107.5 mmol, 1.1 equiv) were dissolved in dry toluene (500 mL) and heated under reflux for 16 h. The mixture was cooled to rt, and the resulting precipitate was collected by filtration on a Büchner funnel. The solid was washed successively with toluene (x3) and Et_2O (x3). After drying under reduced pressure for 1 h, the pure phosphonium salt **16** (45.7 g, 95.5 mmol, 98%) was obtained as a beige powder and used without further purification.

All characterization data were in full agreement with those reported in the literature.[9]

(2-nitrobenzyl)triphenylphosphonium bromide (16)

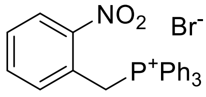

16

Beige powder.

^{1}H NMR (400 MHz, $CDCl_3$) δ 8.11 (ddd, *J* = 7.8, 2.8, 1.5 Hz, 1H), 7.93 (dd, *J* = 8.3, 1.4 Hz, 1H), 7.81 – 7.75 (m, 3H), 7.73 – 7.66 (m, 6H), 7.66 – 7.58 (m, 7H), 7.48 (tt, *J* = 7.8, 1.9 Hz, 1H), 6.12 (d, *J* = 14.9 Hz, 2H).

^{13}C NMR (101 MHz, $CDCl_3$) δ 148.5 (d, *J* = 5.6 Hz), 135.5 (d, *J* = 5.5 Hz), 135.4 (d, *J* = 3.0 Hz), 135.1 (d, *J* = 3.4 Hz), 134.5 (d, *J* = 9.9 Hz), 130.4 (d, *J* = 12.6 Hz), 130.0 (d, *J* = 3.6 Hz), 125.8 (d, *J* = 2.9 Hz), 124.7 (d, *J* = 9.1 Hz), 117.4 (d, *J* = 86.1 Hz), 28.7 (d, *J* = 48.8 Hz).

^{31}P NMR (162 MHz, $CDCl_3$) δ 24.69.

HRMS (nanochip-ESI/LTQ-Orbitrap) m/z: $[M]^+$ Calcd for $C_{25}H_{21}NO_2P^+$ 398.1304; Found 398.1310.

IR (ν_{max}, cm^{-1}) 3050 (w), 3011 (w), 2970 (w), 2831 (w), 2775 (w), 1742 (m), 1515 (s), 1438 (m), 1337 (s), 1198 (w), 1107 (s), 994 (w), 867 (w), 832 (m), 790 (m), 761 (m), 745 (s), 714 (m), 686 (s).

Mp = 236 – 238 °C.

B) Selective formation and enantioselective reduction of enone (*E*)-21

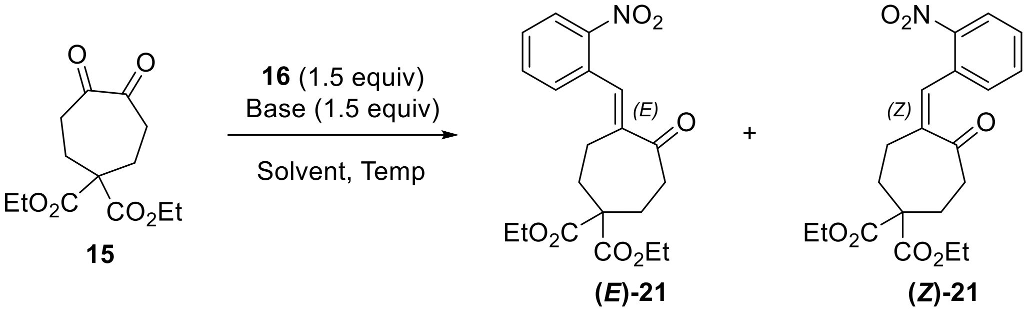

Entry	Base[a]	Solvent	Temp (°C)	Time/Conv.	(*E*)-21:(*Z*)-21[b]
1	NEt_3	DCM	20	24 h / 100%	3.8:1
2	NEt_3	DCM	30	24 h / 100%	3.3:1
3	NEt_3	THF	20	24 h / 100%	6:1
4	NEt_3	1,4-dioxane	20	24 h / 60%	5.4:1
5	NEt_3	*p*-xylene	20	24 h / 70%	9.4:1
6	NEt_3	DME	20	24 h / 100%	7.8:1
**7**	**NEt_3**	**toluene**	**20**	**24 h / 100%**	**9.5:1**
8	$NaHCO_3$	toluene	20	24 h / 0%	-
9	KOH	toluene	20	24 h / 100%	8:1
10	*t*-BuOK	toluene	20	24 h / 100%	Decomp.
11	DIPEA	toluene	20	24 h / 50%	8.3:1
12	Phosphorane[c]	toluene	20	24 h / 0%	-
13	K_2CO_3	toluene	20	24 h / 100%	10:1
**14**	**NEt_3**	**toluene**	**5**	**72 h / 100%**	**12.8:1**
15	NEt_3	toluene	80	24 h / 100%	5:1

Table S3. Condition screening for the Wittig reaction between 15 and 16: effect of base, solvent and temperature on the conversion and the (*E*)-21:(*Z*)-21 ratio.

[a] To a solution of **15** (6.8 mg, 25.2 µmol, 1.0 equiv) and **16** (17.9 mg, 37.7 µmol, 1.5 equiv) in solvent (250 µL) was added base (37.7 µmol, 1.5 equiv) and the reaction mixture was stirred at the indicated temperature and for the specified time. [b] The **(*E*)-21:(*Z*)-21** ratio was determined by crude NMR analysis after filtration of the solids, NH_4Cl/EtOAc work up and subsequent passage through a silica gel pad to remove triphenylphosphine derivatives. The yield is in most cases around 50% over 2 steps. [c] **16** (200 mg, 418 µmol, 1.0 equiv) was dissolved in DCM/H_2O (1/1, 4.0 mL) and deprotonated by the addition of NaOH (3.5 N in H_2O, 239 µL, 836 µmol, 2.0 equiv). After stirring at rt for 1 h, the resulting phosphorane was extracted three times with DCM. The combined organic layers were washed with brine, dried over Na_2SO_4, filtered and concentrated under reduced pressure to give crude phosphorane (152 mg, 382 µmol, 92%) as deep purple crystals.

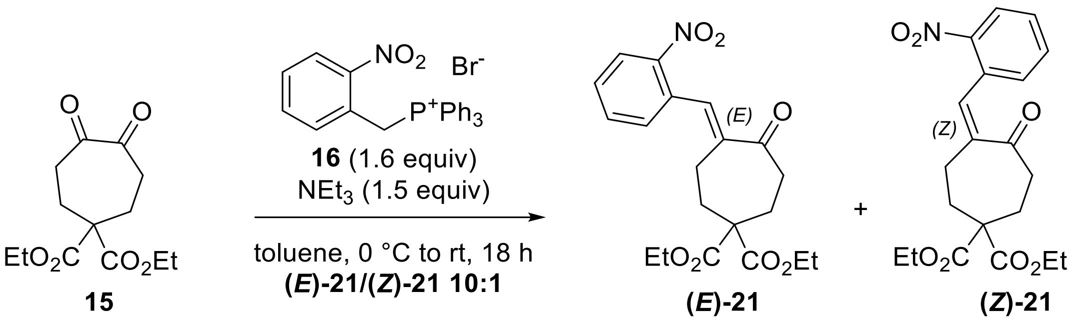

To a solution of crude α-diketone **15** (8.6 g, 31.8 mmol, 1.0 equiv) and phosphonium salt **16** (24.3 g, 50.9 mmol, 1.6 equiv) in dry toluene (320 mL), cooled at 0 °C, was slowly added NEt_3 (6.6 mL, 47.7 mmol, 1.5 equiv), after 30 min, the reaction mixture was slowly warmed to rt and stirred at rt for 18 h. The resulting solids were removed by filtration (washed with toluene), and the filtrate was then poured into saturated NH_4Cl solution and extracted three times with EtOAc. The combined organic layers were washed with brine, dried over Na_2SO_4, filtered and concentrated under reduced pressure. The crude product was purified by FCC (SiO_2, 1/4 EtOAc/Hexane) to give pure enone **(*E*)-21** (4.6 g, 11.6 mmol, 37% over 2 steps) as a beige solid and **(*Z*)-21** (463 mg, 1.2 mmol, 4% over 2 steps) as a pale-yellow oil.

diethyl (*E*)-4-(2-nitrobenzylidene)-5-oxocycloheptane-1,1-dicarboxylate ((*E*)-21)

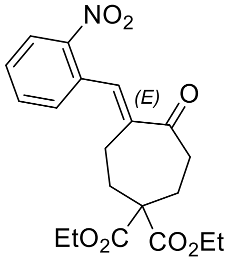

(*E*)-21

Beige solid.

Rf = 0.31 (1/3 EtOAc/Hexane).

^{1}H NMR (400 MHz, $CDCl_3$) δ 8.15 (dd, *J* = 8.2, 1.3 Hz, 1H), 7.76 (s, 1H), 7.65 (td, *J* = 7.5, 1.3 Hz, 1H), 7.51 (dddd, *J* = 8.2, 7.4, 1.5, 0.7 Hz, 1H), 7.26 (dt, *J* = 7.7, 1.2 Hz, 1H), 4.20 (q, *J* = 7.1 Hz, 4H), 2.80 – 2.75 (m, 2H), 2.54 – 2.49 (m, 2H), 2.36 – 2.31 (m, 2H), 2.20 – 2.15 (m, 2H), 1.25 (t, *J* = 7.1 Hz, 6H).

^{13}C NMR (101 MHz, $CDCl_3$) δ 201.8, 171.0, 148.1, 139.9, 134.2, 133.6, 132.3, 131.0, 129.2, 125.2, 61.9, 58.0, 38.8, 34.0, 29.3, 23.7, 14.2.

HRMS (nanochip-ESI/LTQ-Orbitrap) m/z: $[M + Na]^+$ Calcd for $C_{20}H_{23}NNaO_7^+$ 412.1367; Found 412.1376.

IR (ν_{max}, cm^{-1}) 2980 (w), 2939 (w), 2907 (w), 2874 (w), 1725 (s), 1525 (m), 1450 (w), 1345 (w), 1296 (m), 1236 (s), 1179 (m), 1155 (m), 1064 (m), 1023 (m), 903 (w), 857 (w), 790 (w), 732 (w), 707 (w), 673 (w).

Mp = 88 – 90 °C.

diethyl (*Z*)-4-(2-nitrobenzylidene)-5-oxocycloheptane-1,1-dicarboxylate ((*Z*)-21)

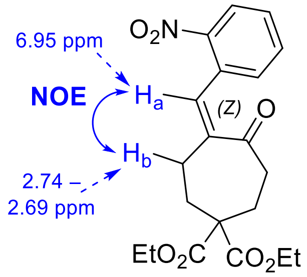

(*Z*)-21

Pale-yellow oil.

Rf = 0.36 (1/3 EtOAc/Hexane).

^{1}H NMR (400 MHz, $CDCl_3$) δ 8.08 (dd, *J* = 8.3, 1.3 Hz, 1H), 7.53 (td, *J* = 7.6, 1.3 Hz, 1H), 7.41 (td, *J* = 7.9, 1.5 Hz, 1H), 7.25 (d, *J* = 7.1 Hz, 1H), 6.95 (s, 1H), 4.23 (q, *J* = 7.2 Hz, 4H), 2.74 – 2.69 (m, 2H), 2.55 – 2.50 (m, 2H), 2.38 – 2.34 (m, 2H), 2.33 – 2.29 (m, 2H), 1.27 (t, *J* = 7.1 Hz, 6H).

^{13}C NMR (101 MHz, $CDCl_3$) δ 203.7, 171.1, 147.5, 141.3, 133.4, 133.3, 133.2, 131.1, 128.4, 124.6, 61.8, 57.8, 39.1, 34.6, 30.5, 28.8, 14.2.

HRMS (nanochip-ESI/LTQ-Orbitrap) m/z: $[M + Na]^+$ Calcd for $C_{20}H_{23}NNaO_7^+$ 412.1367; Found 412.1376.

IR (ν_{max}, cm^{-1}) 2981 (w), 2938 (w), 2907 (w), 1725 (s), 1524 (w), 1448 (w), 1368 (w), 1346 (w), 1294 (m), 1231 (s), 1184 (m), 1097 (m), 1060 (m), 1023 (m), 913 (m), 859 (w), 730 (s).

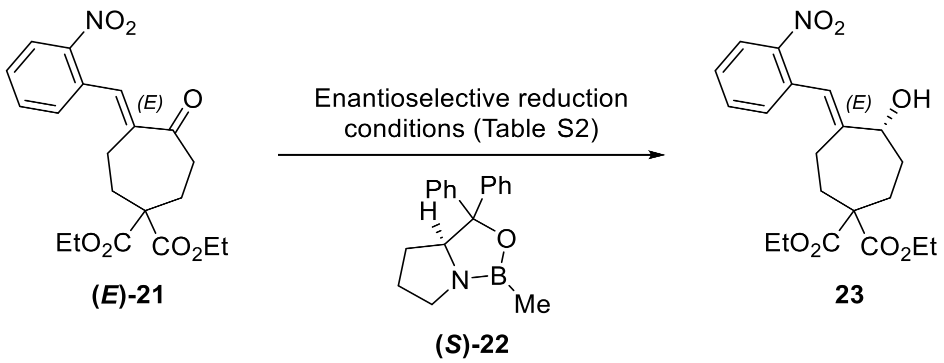

Entry	Isomer	Conditions	Yield (% *ee*)
1	(*E*)-21	**(*S*)-22** (10 mol%), BH_3.DMS (1.1 equiv), THF, -20 °C, 1 h	70% (10% *ee*)
2	(*E*)-21	**(*S*)-22** (100 mol%), BH_3.THF (1.1 equiv), THF, 0 °C, 1 h	95% (95% *ee*)
3	(*E*)-21	**(*S*)-22** (100 mol%), BH_3.THF (1.1 equiv), THF, -20 °C, 1 h	98% (97% *ee*)
4	(*E*)-21	**(*S*)-22** (10 mol%), BH_3.THF (1.1 equiv), THF, -20 °C, 1 h	82% (55% *ee*)
**5**	(*E*)-21	**(*S*)-22 (50 mol%), BH_3.THF (1.1 equiv), THF, -20 °C, 1 h**	**98% (97% *ee*)**
6	(*Z*)-21	**(*R*)-22** (50 mol%), BH_3.THF (1.1 equiv), THF, -20 °C, 1 h	58% (60% conv) (<5% *ee*)
**7**	**(*E*)-21/ (Z)-21 1:1**	**(*S*)-22 (60 mol%), BH_3.THF (55 mol%), THF, -20 °C, 1 h**	**94% (*E*)-21 (97% *ee*) + 91% (*Z*)-21**

Table S4. Condition screening for the enantioselective 1,2-reduction of enone 21.

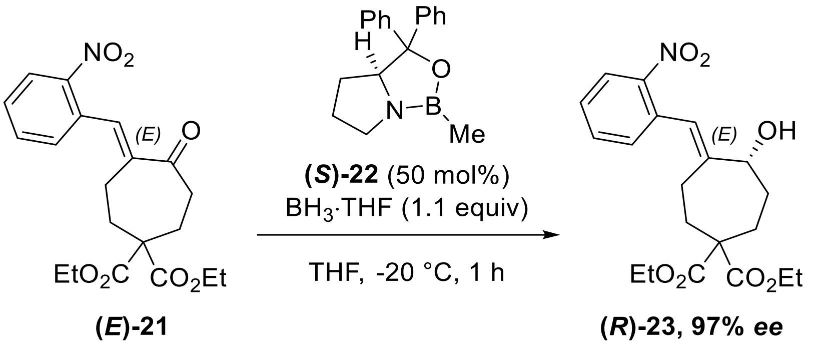

To a stirred solution of CBS catalyst **(*S*)-22** (1.61 g, 5.81 mmol, 50 mol%) in dry THF (11.5 mL) under argon at rt was added dropwise BH_3·THF (1.0 M solution in THF, 12.7 mL, 12.7 mmol, 1.1 equiv). The mixture was stirred for 10 min and then cooled to -20 °C using a cryostat. A solution of enone **(*E*)-21** (4.50 g, 11.6 mmol, 1.0 equiv) in dry THF (46 mL) was added dropwise over 30 min and the resulting mixture was stirred at -20 °C for an additional 1 h. The reaction was quenched at -20 °C by the slow addition of MeOH. The mixture was then poured into a saturated aqueous solution of $NaHCO_3$ and extracted three times with EtOAc. The combined organic layers were washed with brine, dried over Na_2SO_4, filtered and concentrated under reduced pressure. The crude product was purified by FCC (SiO_2, 1/2 EtOAc/hexane) to give compound **(*R*)-23** (4.51 g, 11.5 mmol, 99%, 97% *ee*) as a light-yellow oil.

Preparation of racemic 23:

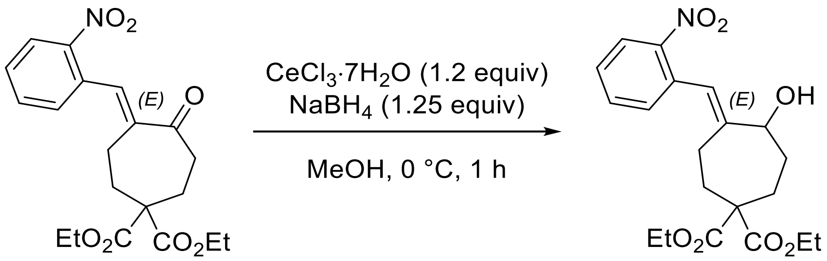

A solution of enone **(*E*)-21** (431 mg, 1.11 mmol, 1.0 equiv) and $CeCl_3 \cdot 7H_2O$ (495 mg, 1.33 mmol, 1.2 equiv) in MeOH (7.4 mL) was stirred at 0 °C for 10 min, followed by the slow addition of $NaBH_4$ (52.3 mg, 1.38 mmol, 1.25 equiv). The mixture was stirred at 0 °C for 1 h, then quenched with a saturated aqueous solution of NH_4Cl and extracted three times with EtOAc. The combined organic layers were washed with brine, dried over Na_2SO_4, filtered and concentrated under reduced pressure to give pure ***rac*-23** (428 mg, 1.09 mmol, 99%) as a light-yellow oil.

diethyl (*R*,*E*)-4-hydroxy-5-(2-nitrobenzylidene)cycloheptane-1,1-dicarboxylate (23)

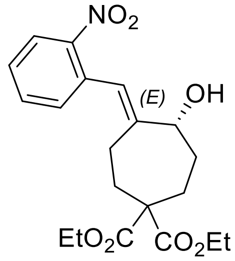

23, 97% *ee*

Light-yellow oil.

Rf = 0.20 (3/7 EtOAc/Hexane).

^{1}H NMR (400 MHz, $CDCl_3$) δ 8.02 (dd, *J* = 8.2, 1.3 Hz, 1H), 7.59 (td, *J* = 7.5, 1.3 Hz, 1H), 7.43 (dddd, *J* = 8.1, 7.4, 1.5, 0.6 Hz, 1H), 7.34 (dt, *J* = 7.6, 1.2 Hz, 1H), 6.77 (s, 1H), 4.46 (ddd, *J* = 7.6, 4.7, 1.1 Hz, 1H), 4.19 – 4.11 (m, 4H), 2.42 – 2.32 (m, 2H), 2.28 – 2.19 (m, 2H), 2.12 – 1.98 (m, 2H), 1.85 – 1.74 (m, 3H), 1.22 (t, *J* = 7.6 Hz, 3H), 1.21 (t, *J* = 7.6 Hz, 3H).

^{13}C NMR (101 MHz, $CDCl_3$) δ 172.2, 171.8, 148.4, 146.4, 133.1, 132.9, 131.9, 128.1, 124.8, 124.0, 75.2, 61.5, 61.5, 57.5, 33.0, 30.8, 27.4, 23.2, 14.1, 14.1.

HRMS (ESI/QTOF) m/z: $[M + Na]^+$ Calcd for $C_{20}H_{25}NNaO_7^+$ 414.1523; Found 414.1532.

IR (ν_{max}, cm^{-1}) 3480 (w), 2978 (w), 2938 (w), 2873 (w), 1725 (s), 1523 (m), 1446 (w), 1346 (m), 1296 (m), 1230 (s), 1183 (s), 1093 (m), 1049 (m), 1023 (m), 959 (w), 857 (m), 788 (w), 745 (w), 706 (w).

$[\alpha]_D^{24}$ = -32 (*c* 0.25, $CHCl_3$).

SFC: IC column, 2.0 mL/min, 16% MeOH in supercritical CO_2, λ = 214.4 nm. Rt (minor) = 2.0 min, Rt (major) = 2.6 min.

SFC chromatogram of racemic compound (*E*)-23:

**Acq. method:**	Run IC 16% MeOH 10 minutes.amx	**Type:**	Sample
**Processing method:**	*Manual Integration.pmx	**Column:**	IC-3
**Manually modified:**	Manual Integration		

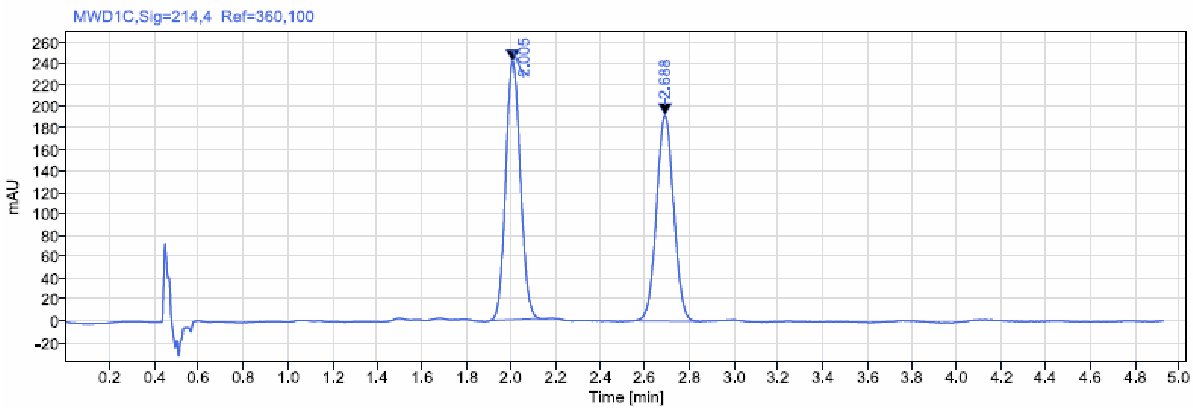

Signal: MWD1C,Sig=214,4 Ref=360,100

RT [min]	Type	Width [min]	Area	Height	Area%	Name
2,005	MM m	0,22	1096,46	240,73	51,65	
2,688	MM m	0,29	1026,24	191,76	48,35	
		**Sum**	**2122,70**			

SFC chromatogram of enantioenriched compound (*E*)-23:

**Acq. method:**	Run IC 16% MeOH 10 minutes.amx	**Type:**	Sample
**Processing method:**	*Manual Integration.pmx	**Column:**	IC-3
**Manually modified:**	Manual Integration		

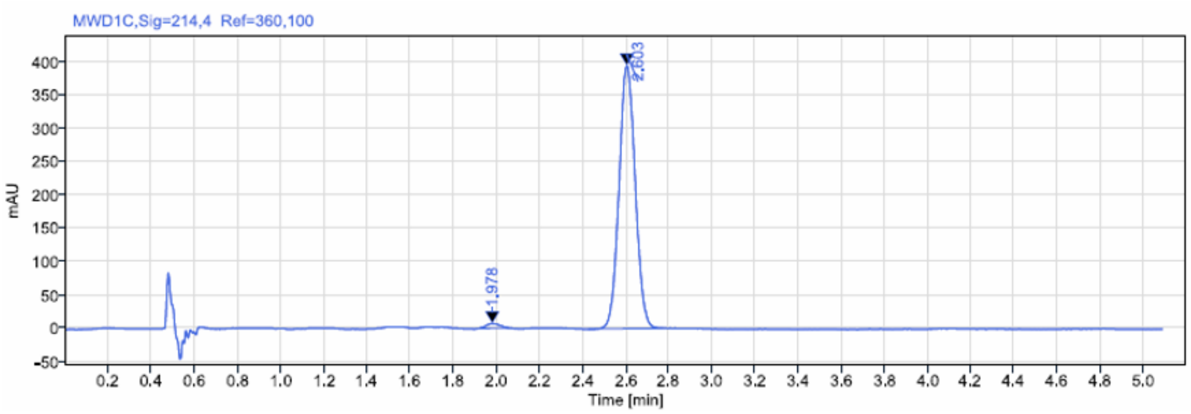

Signal: MWD1C,Sig=214,4 Ref=360,100

RT [min]	Type	Width [min]	Area	Height	Area%	Name
1,978	MM m	0,15	32,06	7,38	1,53	
2,603	MM m	0,35	2057,95	394,54	98,47	
		**Sum**	**2090,01**			

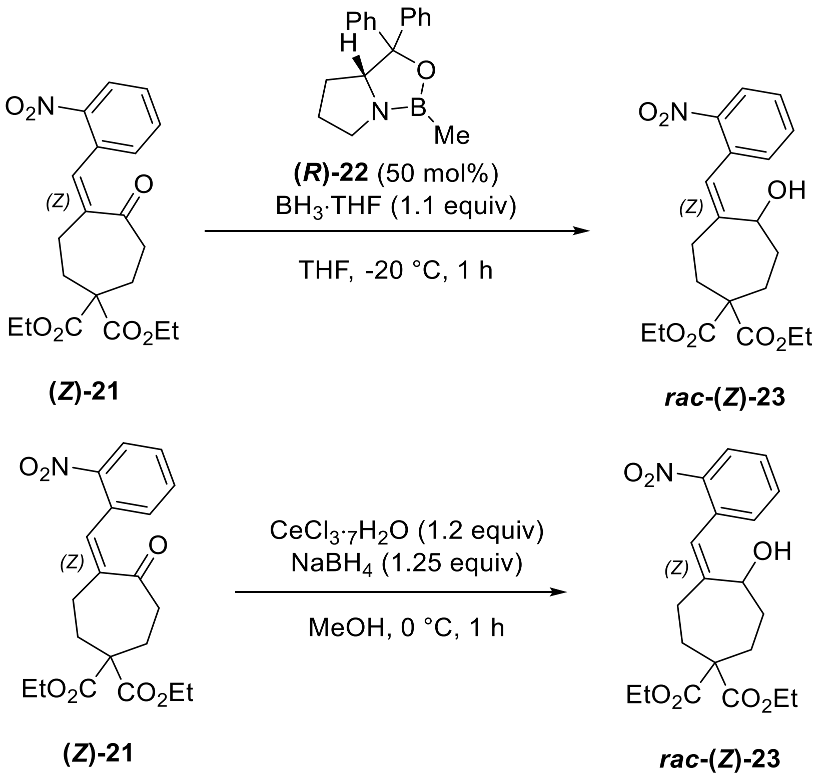

Racemic **(*Z*)-23** (10.5 mg, 26.8 µmol, 60% conversion, 58% yield) was obtained following the above CBS reduction procedure used for **(*E*)-23**, using CBS catalyst **(*R*)-22** (50 mol%).

Racemic **(*Z*)-23** (8.1 mg, 20.7 µmol, 99% yield) was also prepared using the above Luche reduction procedure used for ***rac*-(*E*)-21**.

N.B.: The active reducing species of the CBS reduction appeared to be ineffective in this case probably due to the steric hindrance associated with the (Z)-alkene configuration. ***(Z)-21*** *was only partially converted to racemic allylic alcohol* ***(Z)-23****, suggesting that reduction occurred primarily via free* $BH_3{\cdot}THF$ *rather than the catalyst-bound species.*

diethyl (*Z*)-4-hydroxy-5-(2-nitrobenzylidene)cycloheptane-1,1-dicarboxylate ((*Z*)-23)

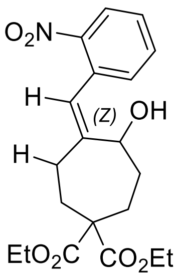

***rac*-(*Z*)-23**

Pale-yellow oil.

Rf = 0.27 (1/2 EtOAc/Hexane).

^{1}H NMR (600 MHz, $CDCl_3$) δ 7.99 (dd, *J* = 8.1, 1.3 Hz, 1H), 7.58 (td, *J* = 7.5, 1.3 Hz, 1H), 7.46 – 7.41 (m, 2H), 6.66 (s, 1H), 4.36 (dd, *J* = 8.2, 5.3 Hz, 1H), 4.26 – 4.12 (m, 4H), 2.72 – 2.66 (m, 2H), 2.39 (ddd, *J* = 14.4, 6.7, 2.5 Hz, 1H), 2.33 (dd, *J* = 14.9, 10.0 Hz, 1H), 1.98 – 1.92 (m, 1H), 1.83 – 1.77 (m, 2H), 1.62 – 1.55 (m, 1H), 1.26 (t, *J* = 7.1 Hz, 3H), 1.24 (t, *J* = 7.1 Hz, 3H).

^{13}C NMR (151 MHz, $CDCl_3$) δ 172.2, 171.9, 148.5, 146.0, 133.1, 132.4, 132.2, 128.3, 126.7, 124.6, 68.5, 61.5, 61.5, 57.8, 35.5, 30.0, 29.3, 26.9, 14.2, 14.2.

HRMS (ESI/QTOF) m/z: $[M + Na]^+$ Calcd for $C_{20}H_{25}NNaO_7^+$ 414.1523; Found 414.1532.

IR (ν_{max}, cm^{-1}) 3450 (w), 2956 (m), 2926 (m), 2862 (w), 1725 (s), 1721 (s), 1523 (s), 1457 (m), 1344 (m), 1298 (m), 1233 (s), 1187 (m), 1095 (w), 1050 (m), 1021 (m), 859 (w), 790 (w), 745 (w), 670 (m).

SFC: IC column, 2.0 mL/min, 15% MeOH in supercritical CO_2, λ = 214.4 nm. Rt_1 = 1.6 min, Rt_2 = 2.2 min.

SFC chromatogram of racemic compound (*Z*)-23:

**Acq. method:**	Run IC 15% MeOH 10 minutes.amx	**Type:**	Sample
**Processing method:**	*Manual Integration.pmx	**Column:**	IC-3
**Manually modified:**	Manual Integration		

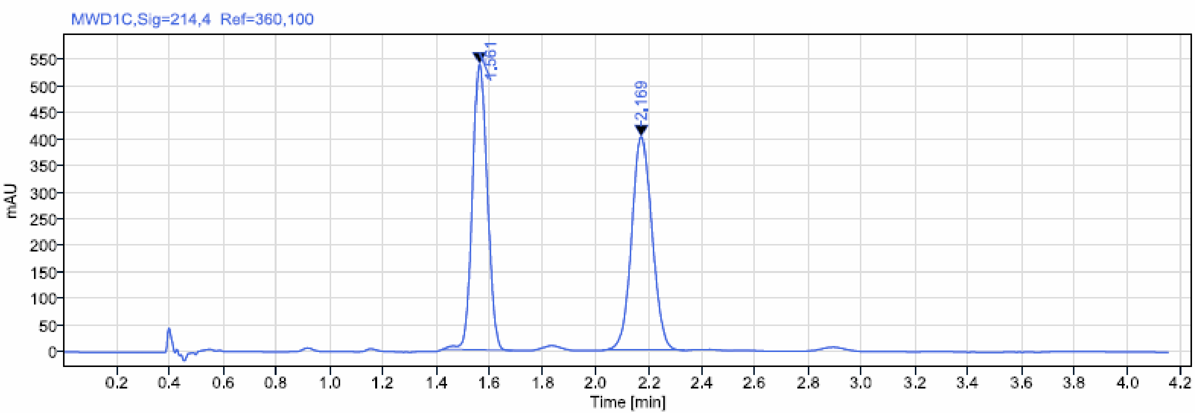

Signal: MWD1C,Sig=214,4 Ref=360,100

RT [min]	Type	Width [min]	Area	Height	Area%	Name
1,561	MM m	0,26	2085,43	538,30	49,29	
2,169	MM m	0,30	2145,68	401,83	50,71	
		**Sum**	**4231.11**			

Selective reduction of (*E*)-21 enone from a (*E*)/(*Z*) mixture:

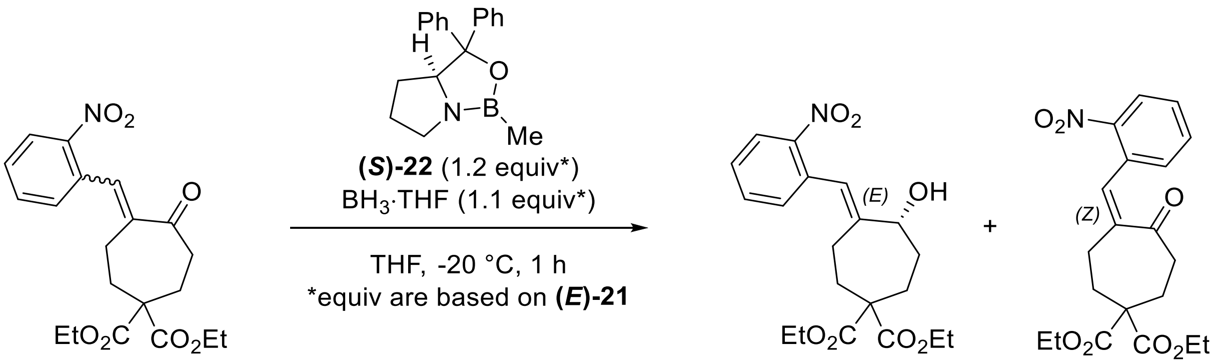

To a stirred solution of (*S*)-CBS catalyst (768 mg, 2.77 mmol, 1.2 equiv) in dry THF (3.0 mL) under argon at rt was added dropwise BH_3·THF (1.0 M solution in THF, 2.5 mL, 2.54 mmol, 1.1 equiv). The mixture was stirred for 10 min and then cooled to -20 °C using a cryostat. A solution of enone **(*E*)-21** (900 mg, 2.31 mmol, 1.0 equiv) and **(*Z*)-21** (900 mg, 2.31 mmol, 1.0 equiv) in dry THF (10 mL) was added dropwise over 30 min and the resulting mixture was stirred at -20 °C for an additional 1 h. The reaction was quenched at -20 °C by the slow addition of MeOH. The

mixture was then poured into a saturated aqueous solution of $NaHCO_3$ and extracted three times with EtOAc. The combined organic layers were washed with brine, dried over Na_2SO_4, filtered and concentrated under reduced pressure. The crude products were purified by FCC (SiO_2, 1/4 for **(*Z*)-21** then 1/2 EtOAc/hexane for **(*E*)-23**) to give compound **(*E*)-23** (850 mg, 2.17 mmol, 94%, 97% *ee*) and to recover unreduced **(*Z*)-21** (820 mg, 2.11 mmol, 91%) as light-yellow oils.

*NB: If some enone **(Z)-21** has been reduced, the resulting alcohol **(Z)-23** could still be separated from **(E)-23** by FCC (3/7 EtOAc/Hexane).*

C) Stereospecific Johnson-Claisen rearrangement and key intermediate synthesis

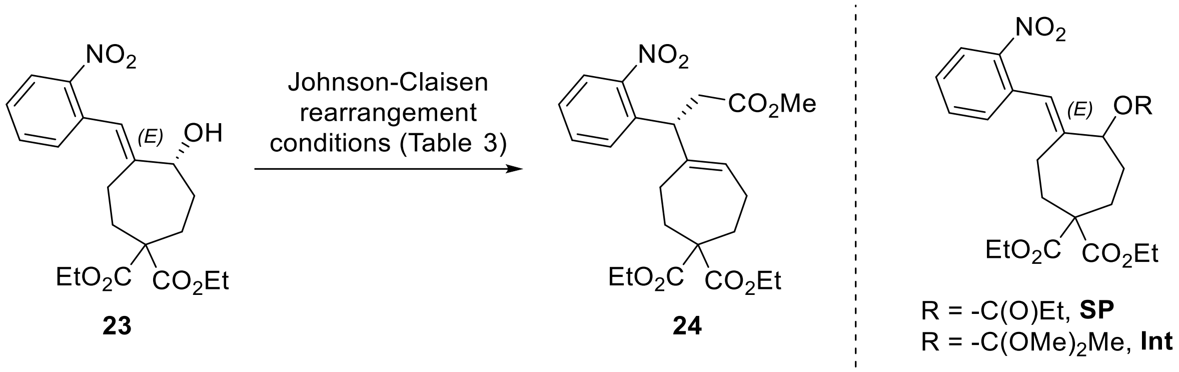

Entry	Conditions	Yield of 24
1	$EtCO_2H$ (10 mol%), $MeC(OMe)_3$ (100.0 equiv), *p*-xylene, 130 °C, 4 h	Only **SP**
2	PivOH (10 mol%), $MeC(OMe)_3$ (10.0 equiv), toluene, 115 °C, 18 h	(11%)
3	PivOH (10 mol%), $MeC(OMe)_3$ (10.0 equiv), *p*-xylene, 135 °C, 4 h	(25%)
4	PivOH (10 mol%), $MeC(OMe)_3$ (10.0 equiv), *p*-xylene, 135 °C, 18 h	Decomp.
5	PivOH (10 mol%), $MeC(OMe)_3$ (100.0 equiv), *p*-xylene, 135 °C, 4 h	32%
6	PivOH (10 mol%), $MeC(OMe)_3$, 135 °C, 3 h	36% (45%)
7	PivOH (10 mol%), $MeC(OMe)_3$, 115 °C, 3 h	(68%)
8	*o*-NO_2-Phenol (50 mol%), $MeC(OMe)_3$, 135 °C, 3 h	(42%)
9	*o*-NO_2-Phenol (50 mol%), $MeC(OMe)_3$, 115 °C, 3 h	(66%)
10	PivOH (10 mol%), $MeC(OMe)_3$, rt, 18 h	(30%) **23** + (38%) **Int**
11	PivOH (10 mol%), $MeC(OMe)_3$, 80 °C, 5 h	(16%) + (48%) **Int**
12	PivOH (10 mol%), $MeC(OMe)_3$, 100 °C, 3 h	(35%) + (35%) **Int**
13	PivOH (10 mol%), $MeC(OMe)_3$, 115 °C, 4 h	(46%) + (26%) **Int**
14[a]	PivOH (20 mol%), $MeC(OMe)_3$ (0.25 M), 75 °C, 1 h then 120 °C, 4 h	42%

**15[a]**	PivOH (20 mol%), MeC(OMe)3 (0.1 M), 75 °C, 1 h then 120 °C, 4 h	60%
**16[a]**	PivOH (20 mol%), MeC(OMe)3, 75 °C, 3 h then 120 °C, 6 h	64%
**17[b]**	PivOH (20 mol%), MeC(OMe)3, 80 °C, 3 h then 120 °C, 4 h	66%

Table S5. Condition screening for the stereospecific [3,3]-sigmatropic Johnson-Claisen rearrangement of 23 to 24.

Reactions were performed on 10 mg scale. [a] 100 mg scale. [b] 500 mg scale.

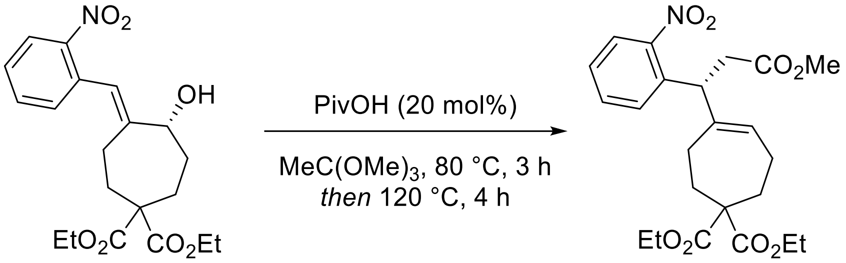

23, 97% *ee*   **24, 97% *ee***

A mixture of allylic alcohol **23** (4.0 g, 10.2 mmol, 1.0 equiv) and PivOH (209 mg, 2.04 mmol, 20 mol%) in degassed trimethylorthoacetate (102 mL) was gradually heated to 80 °C and maintained at this temperature for 3 h while continuously distilling off MeOH to pre-form the mixed orthoester. The temperature was then progressively increased to 120 °C and the reaction mixture was stirred at this temperature for an additional 4 h. After cooling to rt, the volatiles were removed under reduced pressure and the crude product was purified by FCC (SiO_2, 3/7 EtOAc/hexane) to give compound **24** (2.8 g, 6.26 mmol, 61%, 97% *ee*) as a light-yellow oil.

diethyl (*R*)-4-(3-methoxy-1-(2-nitrophenyl)-3-oxopropyl)cyclohept-4-ene-1,1-dicarboxylate (24)

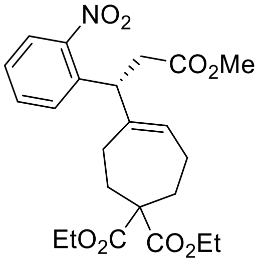

24, 97% *ee*

Light-yellow oil.

Rf = 0.40 (3/7 EtOAc/Hexane).

^{1}H NMR (400 MHz, $CDCl_3$) δ 7.75 – 7.71 (m, 1H), 7.51 (td, *J* = 7.7, 1.4 Hz, 1H), 7.37 – 7.32 (m, 2H), 5.63 (t, *J* = 5.7 Hz, 1H), 4.41 (t, *J* = 7.8 Hz, 1H), 4.19 – 4.09 (m, 4H), 3.60 (s, 3H), 2.78 (dd, *J* = 15.6, 7.5 Hz, 1H), 2.71 (dd, *J* = 15.6, 8.0 Hz, 1H), 2.27 – 2.20 (m, 2H), 2.16 – 1.95 (m, 6H), 1.21 (t, *J* = 7.1 Hz, 3H), 1.19 (t, *J* = 7.1 Hz, 3H).

^{13}C NMR (101 MHz, $CDCl_3$) δ 172.0, 172.0, 171.7, 150.8, 142.8, 136.1, 132.6, 129.2, 127.8, 126.8, 124.5, 61.4, 61.3, 58.3, 52.0, 43.3, 39.1, 31.3, 31.2, 28.0, 23.9, 14.1, 14.1.

HRMS (ESI/QTOF) m/z: $[M + Na]^+$ Calcd for $C_{23}H_{29}NNaO_8^+$ 470.1785; Found 470.1800.

IR (ν_{max}, cm^{-1}) 2976 (w), 2935 (w), 2853 (w), 1725 (s), 1527 (m), 1441 (m), 1357 (m), 1294 (m), 1233 (s), 1177 (s), 1162 (s), 1094 (m), 1067 (m), 1021 (m), 856 (m), 785 (w), 749 (w), 712 (w), 671 (w).

$[\alpha]_D^{24}$ = +46 (*c* 0.25, $CHCl_3$).

SFC: IC column, 2.0 mL/min, 5% MeOH in supercritical CO_2, λ = 214.4 nm. Rt (minor) = 4.7 min, Rt (major) = 5.1 min.

SFC chromatogram of racemic compound 24:

**Acq. method:**	Run IC 5% MeOH 10 minutes.amx	**Type:**	Sample
**Processing method:**	*Manual Integration.pmx	**Column:**	IC-3
**Manually modified:**	Manual Integration		

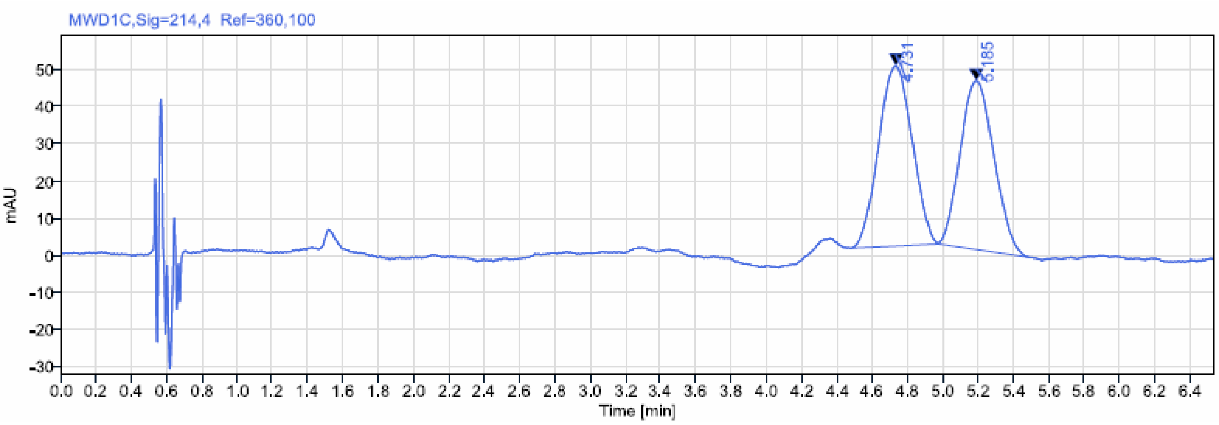

Signal: MWD1C,Sig=214,4 Ref=360,100

RT [min]	Type	Width [min]	Area	Height	Area%	Name
4,731	MM m	0,49	641,70	48,36	51,48	
5,185	MM m	0,51	604,72	45,33	48,52	
		**Sum**	**1246,42**			

SFC chromatogram of enantioenriched compound (*R*)-24:

**Acq. method:**	Run IC 5% MeOH 10 minutes.amx	**Type:**	Sample
**Processing method:**	*Manual Integration.pmx	**Column:**	IC-3
**Manually modified:**	Manual Integration		

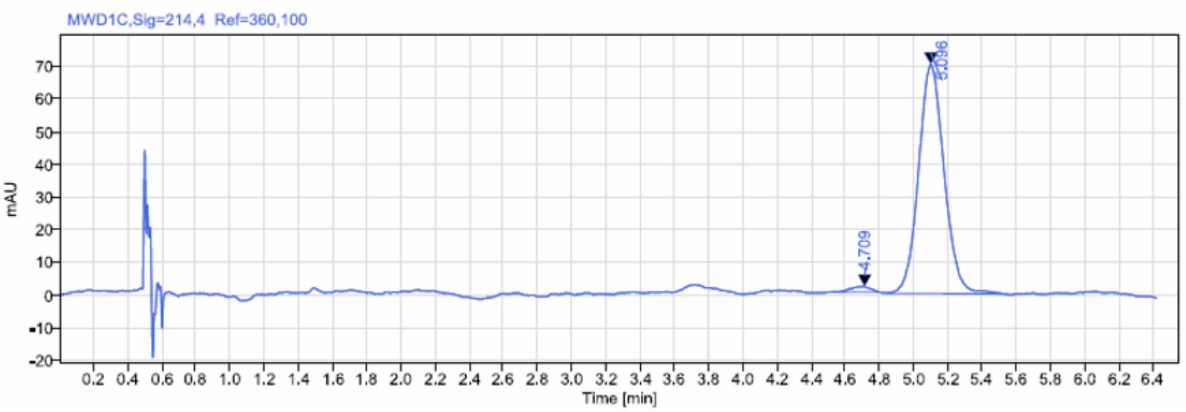

Signal: MWD1C,Sig=214,4 Ref=360,100

RT [min]	Type	Width [min]	Area	Height	Area%	Name
4,709	MM m	0,24	12,26	1,66	1,65	
5,096	MM m	0,69	730,26	70,15	98,35	
		**Sum**	**742,52**			

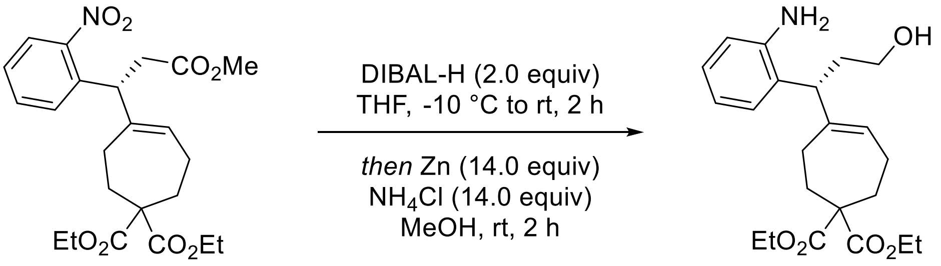

24 **25**

Two-step procedure: Ester **24** (1.02 g, 2.28 mmol, 1.0 equiv) was dissolved in dry THF (23 mL) and cooled to -10 °C (acetone/ice bath). DIBAL-H (1.0 M solution in toluene, 4.56 mL, 4.56 mmol, 2.0 equiv) was added dropwise to the reaction mixture over 30 minutes, and the reaction mixture was stirred at -10 °C for 1 h, then at rt for 1 h. The mixture was then treated with a saturated solution of Rochelle's salt, stirred at rt for 10 min and extracted three times with EtOAc. The combined organic layers were washed with brine, dried over Na_2SO_4, filtered and concentrated under reduced pressure. The crude product was used in the next step without further purification.

NH_4Cl (1.22 g, 22.8 mmol, 10.0 equiv) and Zn dust (1.64 g, 25.1 mmol, 11.0 equiv) were sequentially added to a stirred solution of the above crude product (956 mg, 2.28 mmol, 1.0 equiv) in dry MeOH (23 mL) at room temperature. The mixture was stirred at rt for 2 h. The resulting

solids were filtered through a pad of Celite, washed with MeOH and the filtrate was concentrated under reduced pressure. The residue was basified with a saturated aqueous solution of Na_2CO_3 and extracted four times with EtOAc. The combined organic layers were washed with brine, dried over Na_2SO_4, filtered and concentrated under reduced pressure. The crude product was purified by FCC (SiO_2, 2/1 EtOAc/Hexane) to give aniline **25** (702 mg, 1.8 mmol, 79% over 2 steps) as a colorless oil.

One-pot procedure: Ester **24** (3.4 g, 7.6 mmol, 1.0 equiv) was dissolved in dry THF (76 mL) and cooled to -10 °C (acetone/ice bath). DIBAL-H (1.0 M solution in toluene, 15.2 mL, 15.2 mmol, 2.0 equiv) was added dropwise to the reaction mixture over 30 minutes, and the reaction mixture was stirred for 1 h, then at rt for 1 h. NH_4Cl (5.7 g, 106 mmol, 14.0 equiv), Zn dust (7.0 g, 106 mmol, 14.0 equiv) and MeOH (76 mL) were sequentially added to the stirred solution at room temperature (slightly exothermic). The mixture was stirred at rt for 2 h. The resulting solids were filtered through a pad of Celite, washed with MeOH and the filtrate was concentrated under reduced pressure. The residue was basified with a saturated aqueous solution of Na_2CO_3 and extracted four times with EtOAc. The combined organic layers were washed with brine, dried over Na_2SO_4, filtered and concentrated under reduced pressure. The crude product was purified by FCC (SiO_2, 2/1 EtOAc/Hexane) to give aniline **25** (2.3 g, 5.9 mmol, 78%) as a pale-yellow oil.

diethyl (*R*)-4-(1-(2-aminophenyl)-3-hydroxypropyl)cyclohept-4-ene-1,1-dicarboxylate (25)

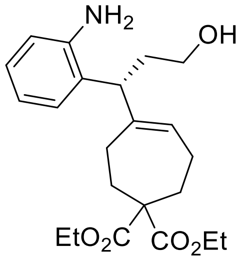

25

Pale-yellow oil.

Rf = 0.54 (2/1 EtOAc/Hexane).

^{1}H NMR (600 MHz, $CDCl_3$) δ 7.04 (t, J = 7.4 Hz, 2H), 6.78 (td, J = 7.5, 1.3 Hz, 1H), 6.69 – 6.66 (m, 1H), 5.77 (t, J = 5.8 Hz, 1H), 4.19 – 4.08 (m, 4H), 3.66 (dt, J = 11.1, 5.7 Hz, 1H), 3.52 – 3.46 (m, 2H), 2.94 (brs, 2H), 2.32 – 2.21 (m, 2H), 2.18 – 2.09 (m, 2H), 2.08 – 1.92 (m, 5H), 1.90 – 1.84 (m, 1H), 1.21 (t, J = 7.1 Hz, 3H), 1.20 (t, J = 7.1 Hz, 3H).

^{13}C NMR (151 MHz, $CDCl_3$) δ 172.1, 144.9, 144.5, 128.1, 127.4, 127.4, 126.3, 119.6, 116.9, 61.3, 60.9, 58.5, 44.4, 35.0, 31.7, 31.4, 26.5, 23.9, 14.2.

HRMS (ESI/QTOF) m/z: $[M + H]^+$ Calcd for $C_{22}H_{32}NO_5^+$ 390.2275; Found 390.2275.

IR (ν_{max}, cm^{-1}) 3450 (w), 3369 (w), 3254 (w), 2979 (w), 2936 (w), 2872 (w), 1721 (s), 1623 (w), 1494 (w), 1453 (m), 1367 (w), 1296 (m), 1233 (s), 1180 (s), 1062 (m), 1024 (m), 750 (s).

$[\alpha]_D^{24}$ = +28 (*c* 0.25, $CHCl_3$).

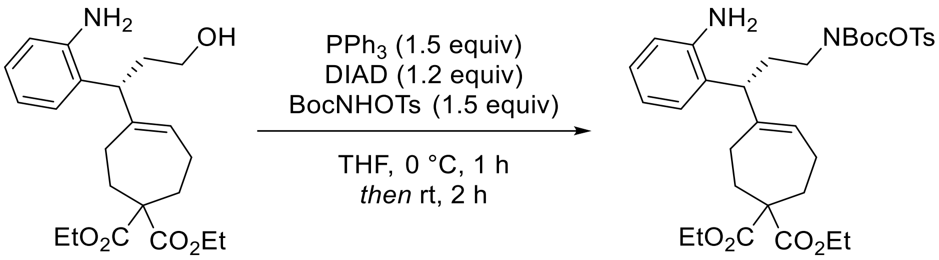

25 **26**

DIAD (1.4 mL, 7.1 mmol, 1.2 equiv) was added dropwise to a solution of PPh_3 (2.3 g, 8.9 mmol, 1.5 equiv) in dry THF (12 mL) at 0 °C and the mixture was stirred for 30 minutes. At 0 °C, a solution of alcohol **25** (2.3 g, 5.9 mmol, 1.0 equiv) in THF (12 mL) was added, followed by the addition of solid NHBocOTs (2.1 g, 7.1 mmol, 1.2 equiv) in one portion. The reaction mixture was stirred at 0 °C for 1 h, then at rt for 2 h. The reaction was quenched with a saturated aqueous solution of $NaHCO_3$ and extracted three times with EtOAc. The combined organic layers were washed with brine, dried over Na_2SO_4, filtered and concentrated under reduced pressure. The crude product was purified by FCC (SiO_2, 1/8 EtOAc/Toluene) to give compound **26** (2.4 g, 3.6 mmol, 62%) as a pale-orange oil.

N.B.: Even though commercially available, NHBocOTs (2.75 g, 9.6 mmol, 91%) was prepared according to reported procedure.[10]

diethyl (*R*)-4-(1-(2-aminophenyl)-3-((*tert*-butoxycarbonyl)(tosyloxy)amino)propyl)cyclohept-4-ene-1,1-dicarboxylate (26)

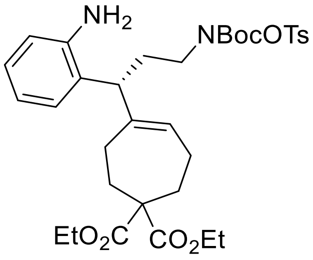

26

Pale-orange oil.

Rf = 0.33 (1/6 EtOAc/Toluene).

^{1}H NMR (400 MHz, $CDCl_3$) δ 7.83 (d, *J* = 8.3 Hz, 2H), 7.31 (d, *J* = 8.1 Hz, 2H), 7.05 – 6.98 (m, 2H), 6.74 (td, *J* = 7.5, 1.3 Hz, 1H), 6.62 (dd, *J* = 7.8, 1.3 Hz, 1H), 5.73 (t, *J* = 5.6 Hz, 1H), 4.19 – 4.07 (m, 4H), 3.73 (brs, 2H), 3.55 (brs, 2H), 3.16 (t, *J* = 7.5 Hz, 1H), 2.43 (s, 3H), 2.28 – 2.20 (m, 2H), 2.17 – 1.87 (m, 8H), 1.24 (s, 9H), 1.22 (t, *J* = 7.1 Hz, 3H), 1.19 (t, *J* = 7.1 Hz, 3H).

^{13}C NMR (101 MHz, $CDCl_3$) δ 172.0, 171.9, 155.5, 145.7, 145.3, 143.4, 131.3, 129.8, 129.6, 127.7, 127.5, 127.4, 125.6, 118.7, 116.5, 83.4, 61.3, 61.3, 58.4, 52.1, 46.9, 31.5, 31.4, 27.8, 27.2, 25.2, 23.9, 21.8, 14.1, 14.1.

HRMS (APCI/QTOF) m/z: $[M + H]^+$ Calcd for $C_{34}H_{47}N_2O_9S^+$ 659.2997; Found 659.3000.

IR (ν_{max}, cm^{-1}) 3448 (w), 3376 (w), 2979 (w), 2936 (w), 1723 (s), 1625 (w), 1599 (w), 1494 (w), 1452 (m), 1370 (m), 1297 (m), 1233 (s), 1177 (s), 1157 (s), 1092 (m), 1067 (m), 1024 (w), 847 (m), 815 (m), 743 (s), 664 (m).

$[\alpha]_D^{24}$ = +3 (*c* 0.25, $CHCl_3$).

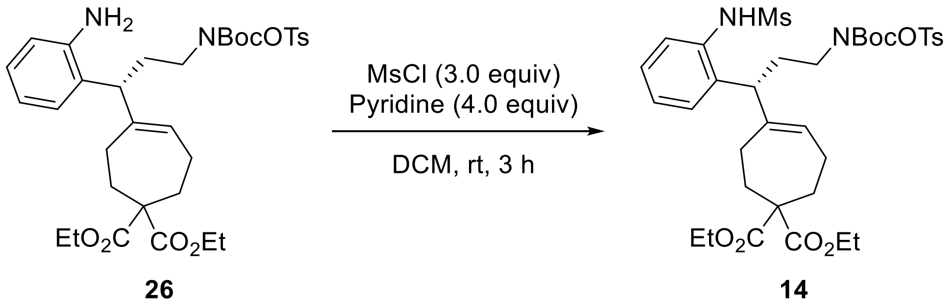

MsCl (813 µL, 10.5 mmol, 3.0 equiv) was slowly added to a stirred solution of aniline **26** (2.3 g, 3.5 mmol, 1.0 equiv) and pyridine (1.13 mL, 14.0 mmol, 4.0 equiv) in dry DCM (24 mL) at 0 °C. The reaction mixture was stirred at 0 °C for 10 minutes then at rt for 3 h. The reaction was quenched with a saturated aqueous solution of NH_4Cl and extracted three times with DCM. The combined organic layers were washed with brine, dried over Na_2SO_4, filtered and concentrated under reduced pressure. The crude product was purified by FCC (SiO_2, 1/1 EtOAc/Hexane) to give compound **14** (2.5 g, 3.4 mmol, 97%) as a beige foam.

diethyl (*R*)-4-(3-((*tert*-butoxycarbonyl)(tosyloxy)amino)-1-(2-(methylsulfonamido)phenyl)propyl)cyclohept-4-ene-1,1-dicarboxylate (14)

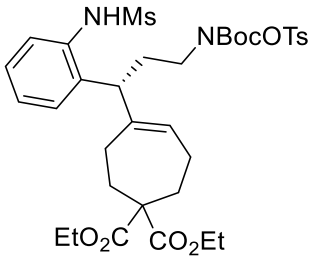

14

Beige foam.

Rf = 0.40 (2/3 EtOAc/Hexane).

^{1}H NMR (400 MHz, $CDCl_3$) δ 7.85 – 7.81 (m, 2H), 7.50 (dd, *J* = 8.0, 1.2 Hz, 1H), 7.34 (d, *J* = 8.0 Hz, 2H), 7.29-7.24 (m, 1H), 7.23 – 7.14 (m, 2H), 6.90 (s, 1H), 5.85 (t, *J* = 5.6 Hz, 1H), 4.19 – 4.09 (m, 4H), 3.57 (brs, 2H), 3.37 (t, *J* = 7.5 Hz, 1H), 3.05 (s, 3H), 2.45 (s, 3H), 2.32-2.25 (m, 2H), 2.21 – 1.95 (m, 6H), 1.93-1.86 (m, 2H), 1.23 (s, 9H), 1.22 – 1.19 (m, 6H).

^{13}C NMR (101 MHz, $CDCl_3$) δ 171.9, 171.8, 155.6, 146.0, 143.5, 136.0, 131.9, 131.2, 129.8, 129.8, 129.1, 128.5, 128.2, 125.6, 122.1, 83.8, 61.4, 58.2, 51.7, 47.3, 40.4, 31.4, 31.3, 27.8, 27.6, 25.2, 24.0, 21.9, 14.2, 14.1.

HRMS (ESI/QTOF) m/z: $[M + Na]^+$ Calcd for $C_{35}H_{48}N_2NaO_{11}S_2^+$ 759.2592; Found 759.2621.

IR (ν_{max}, cm^{-1}) 3306 (w), 2978 (w), 2935 (w), 2907 (w), 2853 (w), 1720 (s), 1458 (m), 1369 (m), 1329 (m), 1298 (m), 1236 (m), 1190 (m), 1178 (s), 1153 (s), 1092 (w), 1066 (w), 1020 (w), 971 (w), 913 (m), 847 (w), 816 (m), 733 (s), 669 (w).

$[\alpha]_D^{24}$ = +5 (*c* 0.25, $CHCl_3$).

D) Two-step key sequence towards melonine core

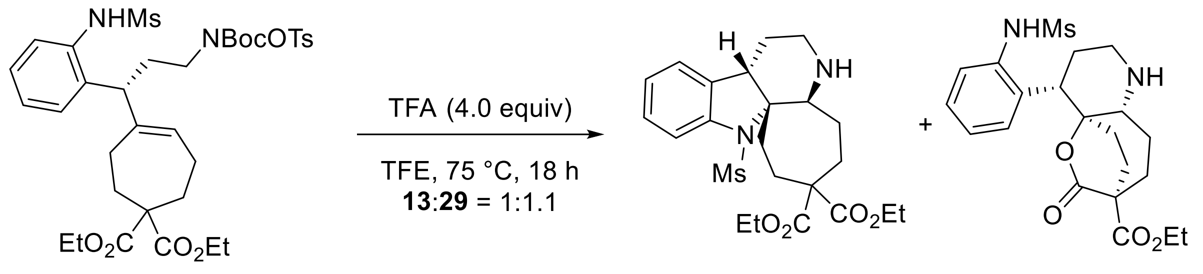

14 **13** **29**

In a dry flask under argon, TFA (1.0 mL, 13.2 mmol, 4.0 equiv) was added to a stirred solution of alkene **14** (2.4 g, 3.3 mmol, 1.0 equiv) in dry and degassed TFE (33 mL) at rt. The reaction mixture was then stirred at 75 °C for 18 h. After cooling to rt, volatiles were removed under reduced pressure. A saturated aqueous solution of Na_2CO_3 was added and the mixture was extracted three times with EtOAc. The combined organic layers were washed with brine, dried over Na_2SO_4, filtered and concentrated under reduced pressure. The crude products were purified by FCC (SiO_2, EtOAc +1% NEt_3 to 1/15 MeOH/EtOAc + 1% NEt_3 for **15** then to 1/4 MeOH/EtOAc + 1% NEt_3 for **29**) to give compound **13** (721 mg, 1.55 mmol, 48%) as a light-brown foam and compound **29** (738 mg, 1.69 mmol, 52%) as a white foam.

Hexane, was added to compound **29**, followed by gradual addition of a few drops of MeOH until complete dissolution was achieved, yielding a homogeneous solution. The product **29** was then recrystallized by slow open-air evaporation to afford light-brown needle crystals.

diethyl (5a*S*,8a*S*,13a*R*)-13-(methylsulfonyl)-1,2,4,5,5a,6,7,8,8a,13-decahydro-3H-cyclohepta[2,3]pyrido[3,4-b]indole-3,3-dicarboxylate (13)

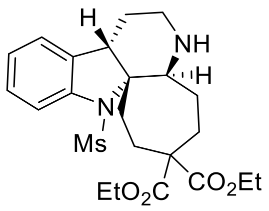

13

Light-brown foam.

Rf = 0.27 (1/19 MeOH/EtOAc).

^{1}H NMR (400 MHz, $CDCl_3$) δ 7.49 (d, *J* = 8.1 Hz, 1H), 7.21 – 7.13 (m, 2H), 7.00 (td, *J* = 7.4, 1.0 Hz, 1H), 4.25 – 4.20 (m, 1H), 4.20 – 4.09 (m, 4H), 3.20 (s, 3H), 3.11 – 3.02 (m, 1H), 2.83 (ddd, *J*

= 12.4, 9.9, 4.1 Hz, 1H), 2.65 (dt, J = 12.4, 5.1 Hz, 1H), 2.52 (dd, J = 14.6, 7.6 Hz, 1H), 2.34 (dd, J = 15.0, 9.8 Hz, 1H), 2.23 – 2.05 (m, 3H), 2.00 – 1.89 (m, 2H), 1.82 (ddt, J = 15.0, 7.7, 1.7 Hz, 1H), 1.73 (brs, 1H), 1.52 (dd, J = 15.1, 9.8 Hz, 1H), 1.44 – 1.32 (m, 1H), 1.21 (t, J = 7.1 Hz, 3H), 1.21 (t, J = 7.1 Hz, 3H).

^{13}C NMR (101 MHz, $CDCl_3$) δ 172.2, 172.0, 141.0, 135.1, 128.0, 124.1, 123.5, 115.5, 77.6, 61.5, 61.5, 57.1, 55.9, 44.5, 40.1, 36.8, 33.3, 31.8, 31.1, 30.3, 25.9, 14.1.

HRMS (nanochip-ESI/LTQ-Orbitrap) m/z: $[M + H]^+$ Calcd for $C_{23}H_{33}N_2O_6S^+$ 465.2054; Found 465.2056.

IR (ν_{max}, cm^{-1}) 2978 (w), 2950 (w), 2929 (w), 2854 (w), 1724 (s), 1462 (m), 1332 (m), 1238 (s), 1183 (m), 1153 (s), 1081 (w), 1027 (m), 1011 (m), 969 (m), 911 (m), 856 (w), 755 (s), 731 (s).

$[\alpha]_D^{24}$ = +5 (c 0.25, $CHCl_3$).

ethyl (4*S*,4a*S*,7*R*,9a*R*)-4-(2-(methylsulfonamido)phenyl)-10-oxooctahydro-4a,7-(epoxymethano)cyclohepta[b]pyridine-7(2H)-carboxylate (29)

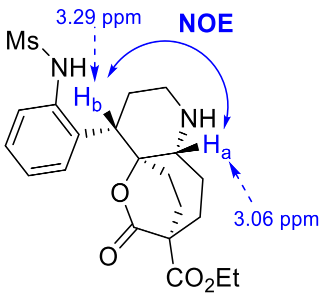

29

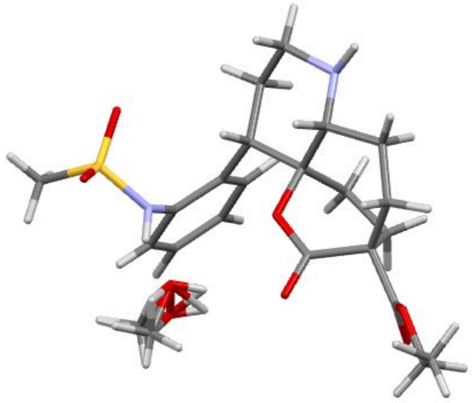

White foam/Light-brown needle crystals.

Rf = 0.22 (1/8 MeOH/EtOAc).

X-Ray structure of 29.

^{1}H NMR (400 MHz, $CDCl_3$) δ 7.52 – 7.48 (m, 1H), 7.34 – 7.27 (m, 1H), 7.21 – 7.16 (m, 2H), 4.24 – 4.11 (m, 2H), 3.29 (dd, J = 13.2, 3.5 Hz, 1H), 3.17 (s, 3H), 3.12 (ddd, J = 12.0, 4.0, 2.3 Hz, 1H), 3.06 (ddd, J = 10.9, 5.3, 1.6 Hz, 1H), 2.82 (td, J = 11.9, 2.4 Hz, 1H), 2.37 (ddd, J = 14.6, 12.4, 2.8 Hz, 1H), 2.27 (ddd, J = 14.1, 11.1, 2.9 Hz, 1H), 2.21-2.19 (m, 1H), 2.18 (d, J = 3.7 Hz, 1H), 2.07 – 1.92 (m, 3H), 1.84 (dddd, J = 14.2, 12.4, 6.0, 1.7 Hz, 1H), 1.76 – 1.64 (m, 2H), 1.24 (t, J = 7.1 Hz, 3H).

^{13}C NMR (101 MHz, $CDCl_3$) δ 171.4, 170.8, 137.0, 131.7, 130.1, 128.4, 124.8, 121.9, 88.9, 62.1, 61.3, 50.9, 46.5, 46.2, 41.0, 30.6, 29.3, 29.1, 23.6, 18.2, 14.1.

HRMS (nanochip-ESI/LTQ-Orbitrap) m/z: $[M + H]^+$ Calcd for $C_{21}H_{29}N_2O_6S^+$ 437.1741; Found 437.1752.

IR (ν_{max}, cm^{-1}) 3332 (w), 2923 (w), 2858 (w), 1734 (s), 1492 (w), 1452 (w), 1368 (m), 1324 (m), 1273 (m), 1233 (s), 1183 (m), 1152 (s), 1095 (m), 1051 (s), 970 (m), 912 (m), 828 (w), 760 (m), 730 (s).

Mp = 73 – 75 °C.

$[\alpha]_D^{24}$ = +27 (*c* 0.25, $CHCl_3$).

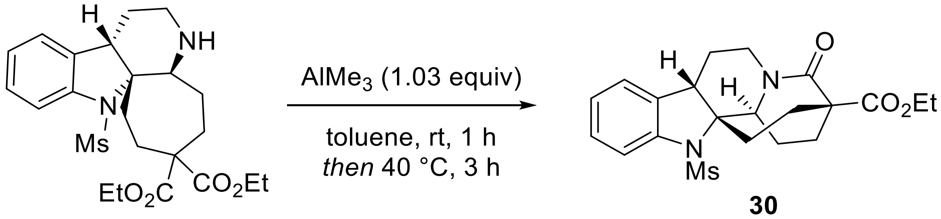

13 **30**

To an argon-flushed flask cooled at -10 °C (acetone/ice bath) containing amine **13** (695 mg, 1.50 mmol, 1.0 equiv) in dry and degassed toluene (50 mL), was added dropwise a solution of $AlMe_3$ (2.0 M in toluene, 770 μL, 1.54 mmol, 1.03 equiv). The reaction mixture was stirred at rt for 1 h, then at 40 °C for 3 h. The reaction was quenched by pouring into a saturated aqueous solution of Na_2CO_3. The resulting mixture was extracted three times with EtOAc. The combined organic layers were washed with brine, dried over Na_2SO_4, filtered and concentrated under reduced pressure. The crude product was purified by FCC (SiO_2, 3/2 EtOAc/Hexane) to give compound **30** (464 mg, 1.11 mmol, 74%) as a beige foam.

*N.B.: Equivalents of $AlMe_3$ need to be carefully controlled to avoid the ester bis-methylation side reaction (see compound **S22** below).*

ethyl (3*S*,5a*S*,8a*S*,13a*R*)-13-(methylsulfonyl)-14-oxo-1,2,5,5a,7,8,8a,13-octahydro-3,6-methanocyclohepta[2,3]pyrido[3,4-b]indole-3(4H)-carboxylate (30)

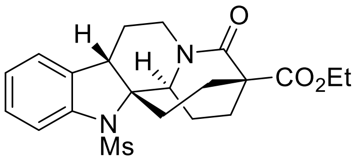

30

Beige foam.

Rf = 0.30 (1/1 EtOAc/Hexane).

^{1}H NMR (400 MHz, $CDCl_3$) δ 7.46 (d, *J* = 8.1 Hz, 1H), 7.23 (t, *J* = 7.8 Hz, 1H), 7.17 (d, *J* = 7.3 Hz, 1H), 7.08 (t, *J* = 7.4 Hz, 1H), 4.46 (dt, *J* = 12.9, 7.5 Hz, 1H), 4.23 (q, *J* = 7.1 Hz, 2H), 3.77 (d, *J* = 6.9 Hz, 1H), 3.10 (dd, *J* = 9.0, 5.0 Hz, 1H), 3.05 (s, 3H), 2.92-2.77 (m, 2H), 2.65 – 2.50 (m, 2H), 2.41-2.31 (m, 1H), 2.24-2.13 (m, 2H), 2.04 – 1.85 (m, 4H), 1.29 (t, *J* = 7.1 Hz, 3H).

^{13}C NMR (101 MHz, $CDCl_3$) δ 174.3, 172.0, 141.8, 131.9, 128.6, 124.5, 124.5, 116.0, 77.3, 61.6, 56.6, 53.3, 45.8, 41.2, 40.8, 29.3, 28.8, 25.9, 24.9, 19.6, 14.2.

HRMS (nanochip-ESI/LTQ-Orbitrap) m/z: $[M + H]^+$ Calcd for $C_{21}H_{27}N_2O_5S^+$ 419.1635; Found 419.1641.

IR (ν_{max}, cm^{-1}) 2934 (w), 2885 (w), 1730 (m), 1658 (m), 1477 (m), 1458 (m), 1430 (w), 1345 (s), 1238 (m), 1215 (m), 1160 (s), 1118 (m), 1099 (m), 1026 (m), 971 (m), 911 (m), 756 (m), 730 (s).

$[\alpha]_D^{24}$ = -47 (*c* 0.25, $CHCl_3$).

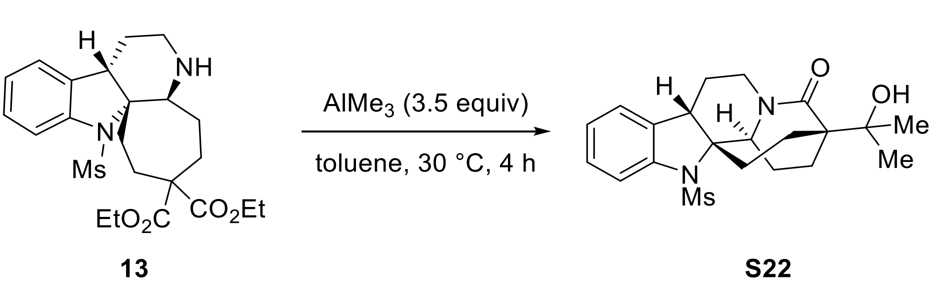

13 **S22**

In an argon-flushed flask containing amine **13** (20 mg, 43 µmol, 1.0 equiv) in dry and degassed toluene (1.4 mL) was added dropwise a solution of $AlMe_3$ (2.0 M in toluene, 75 µL, 151 µmol, 3.5 equiv) at rt (exothermic). The reaction mixture was stirred at 30 °C for 4 h before being poured into a saturated aqueous solution of Na_2CO_3. The mixture was extracted three times with EtOAc.

The combined organic layers were washed with brine, dried over Na_2SO_4, filtered and concentrated under reduced pressure. The crude product was purified by FCC (SiO_2, 3/1 EtOAc/Hexane) to give compound **S22** (13 mg, 31 µmol, 72%) as a beige foam.

(3*R*,5a*S*,8a*S*,13a*R*)-3-(2-hydroxypropan-2-yl)-13-(methylsulfonyl)-1,2,3,4,5,5a,7,8,8a,13-decahydro-3,6-methanocyclohepta[2,3]pyrido[3,4-b]indol-14-one (S22)

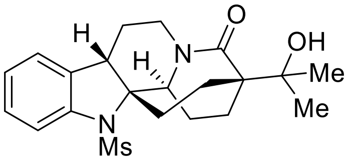

S22

Beige foam.

Rf = 0.43 (3/2 EtOAc/Hexane).

^{1}H NMR (400 MHz, $CDCl_3$) δ 7.55 (d, J = 8.1 Hz, 1H), 7.28 – 7.23 (m, 1H), 7.20 (d, J = 7.2 Hz, 1H), 7.13 (t, J = 7.3 Hz, 1H), 5.41 (brs, 1H), 4.36 (ddd, J = 13.0, 6.8, 3.1 Hz, 1H), 3.40 (d, J = 9.0 Hz, 1H), 3.25 (t, J = 5.1 Hz, 1H), 2.98 (s, 3H), 2.69 (dq, J = 14.7, 4.5 Hz, 2H), 2.59-2.48 (m, 1H), 2.38 – 2.19 (m, 2H), 2.09 – 1.85 (m, 4H), 1.81-1.71 (m, 2H), 1.35 (s, 3H), 1.12 (s, 3H).

^{13}C NMR (101 MHz, $CDCl_3$) δ 179.9, 141.7, 132.5, 128.8, 125.0, 123.7, 117.3, 76.2, 74.3, 56.0, 50.0, 48.9, 42.0, 40.9, 29.5, 26.9, 26.5, 23.7, 22.0, 19.1.

HRMS (ESI/QTOF) m/z: $[M + H]^+$ Calcd for $C_{21}H_{29}N_2O_4S^+$ 405.1843; Found 405.1825.

IR (ν_{max}, cm^{-1}) 3410 (w), 3062 (w), 2978 (w), 2938 (w), 1630 (m), 1475 (w), 1458 (w), 1345 (m), 1266 (w), 1229 (w), 1203 (w), 1161 (m), 1096 (w), 1041 (w), 1018 (w), 969 (w), 732 (s), 701 (m).

$[\alpha]_D^{24}$ = -32 (c 0.25, $CHCl_3$).

E) End-game of the total synthesis of (+)-melonine

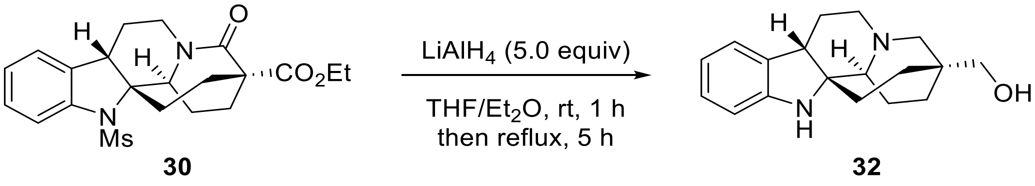

A solution of $LiAlH_4$ (1.0 M in Et_2O, 514 µL, 514 µmol, 5.0 equiv) was added dropwise to a stirred solution of β-amido-ester **30** (43 mg, 103 µmol, 1.0 equiv) in dry THF (1.0 mL) at 0 °C. The

reaction mixture was stirred at rt for 1 h, then refluxed for 5 h. After cooling, the reaction was quenched using a Fieser work-up. The crude product was purified by preparative TLC (SiO_2, 2/9 MeOH/EtOAc + 1% NEt_3) to give compound **32** (23 mg, 80.9 µmol, 79%) as a beige foam.

A minimum amount of MeOH was added to a heterogeneous solution of product **32** in hexane until complete dissolution. The product was then recrystallized by storing the solution in a freezer (-35 °C) for 5 days, followed by careful removal of the solvent at low temperature to afford yellow crystals.

((3*S*,5a*S*,8a*S*,13a*R*)-1,2,5,5a,7,8,8a,13-octahydro-3,6-methanocyclohepta[2,3]pyrido[3,4-b]indol-3(4H)-yl)methanol (32)

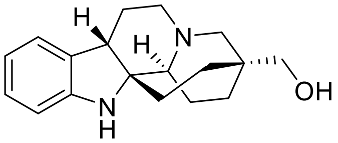

32

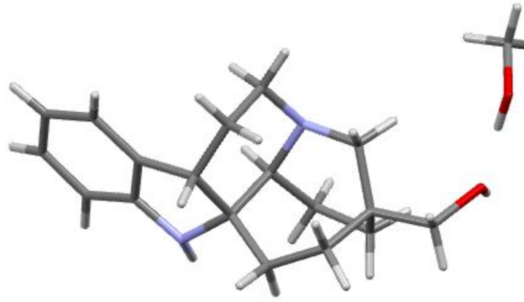

Beige foam/Yellow crystals.

Rf = 0.40 (2/9 MeOH/DCM + 1% NEt_3).

X-Ray structure of 32.

^{1}H NMR (600 MHz, $CDCl_3$) δ 7.04 – 7.00 (m, 2H), 6.74 (td, *J* = 7.4, 1.0 Hz, 1H), 6.55 (dt, *J* = 7.3, 1.0 Hz, 1H), 3.33 (d, *J* = 10.6 Hz, 1H), 3.29 (d, *J* = 10.5 Hz, 1H), 3.18 (d, *J* = 5.5 Hz, 1H), 3.04 (d, *J* = 11.6 Hz, 1H), 2.92 (td, *J* = 13.4, 3.1 Hz, 1H), 2.80 (d, *J* = 5.6 Hz, 1H), 2.69 – 2.64 (m, 1H), 2.55 (dd, *J* = 11.6, 2.6 Hz, 1H), 2.23 – 2.15 (m, 1H), 1.99 – 1.91 (m, 2H), 1.89-1.85 (m, 2H), 1.81 (dddd, *J* = 14.8, 11.5, 3.8, 1.7 Hz, 1H), 1.73 – 1.66 (m, 2H), 1.51 (dddd, *J* = 14.1, 11.6, 5.0, 2.7 Hz, 1H), 1.41 (td, *J* = 12.9, 3.8 Hz, 1H).

^{13}C NMR (151 MHz, $CDCl_3$) δ 149.6, 131.4, 127.8, 122.9, 119.0, 109.6, 72.1, 67.7, 57.0, 56.5, 48.8, 47.0, 38.7, 34.9, 33.0, 23.5, 21.8, 20.3.

HRMS (ESI/QTOF) m/z: $[M + H]^+$ Calcd for $C_{18}H_{25}N_2O^+$ 285.1961; Found 285.1956.

IR (ν_{max}, cm^{-1}) 3319 (w), 2923 (m), 2859 (w), 1606 (w), 1482 (w), 1461 (m), 1263 (m), 1054 (m), 1037 (w), 1018 (w), 802 (w), 734 (s), 702 (m).

Mp = 87 – 89 °C.

$[\alpha]_D^{24}$ = -60 (*c* 0.25, $CHCl_3$).

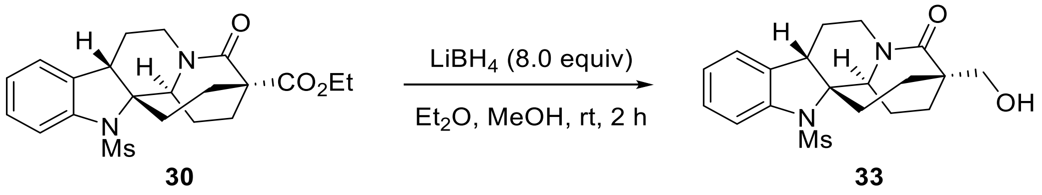

In a dry flask under argon, *β*-amido-ester **30** (440 mg, 1.05 mmol, 1.0 equiv) was dissolved in dry Et_2O (10.5 mL) and MeOH (1.3 mL). While maintaining the solution at rt using a water bath, $LiBH_4$ (183 mg, 8.4 mmol, 8.0 equiv) was added portionwise and the reaction mixture was stirred at rt for 1 h. MeOH (1.3 mL) was added, and the mixture was stirred at rt for 1 h. The mixture was then poured into a saturated aqueous solution of Na_2CO_3 and extracted three times with EtOAc. The combined organic layers were washed with brine, dried over Na_2SO_4, filtered and concentrated under reduced pressure. The crude product was purified by FCC (SiO_2, EtOAc) to give compound **33** (363 mg, 964 µmol, 92%) as a white foam.

(3*R*,5a*S*,8a*S*,13a*R*)-3-(hydroxymethyl)-13-(methylsulfonyl)-1,2,3,4,5,5a,7,8,8a,13-decahydro-3,6-methanocyclohepta[2,3]pyrido[3,4-b]indol-14-one (33)

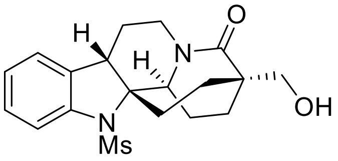

33

White foam.

Rf = 0.50 (EtOAc).

^{1}H NMR (400 MHz, $CDCl_3$) δ 7.46 (d, *J* = 8.2 Hz, 1H), 7.23 (t, *J* = 7.9 Hz, 1H), 7.18 (d, *J* = 7.3 Hz, 1H), 7.07 (t, *J* = 7.4 Hz, 1H), 4.45 (dt, *J* = 12.9, 7.7 Hz, 1H), 3.75 (d, *J* = 6.8 Hz, 1H), 3.57 – 3.45 (m, 3H), 3.08 (d, *J* = 5.3 Hz, 1H), 3.06 (s, 3H), 2.95 – 2.85 (m, 2H), 2.64 (ddd, *J* = 14.1, 10.5, 5.7 Hz, 1H), 2.18 – 1.84 (m, 6H), 1.76 – 1.58 (m, 2H).

^{13}C NMR (101 MHz, $CDCl_3$) δ 179.3, 141.9, 131.8, 128.6, 124.6, 124.3, 115.9, 77.5, 68.7, 56.5, 45.7, 45.4, 40.9, 40.8, 30.0, 29.2, 25.4, 24.8, 20.1.

HRMS (ESI/QTOF) m/z: $[M + H]^+$ Calcd for $C_{19}H_{25}N_2O_4S^+$ 377.1530; Found 377.1519.

IR (ν_{max}, cm^{-1}) 3386 (w), 2924 (m), 2854 (m), 1726 (w), 1666 (m), 1478 (m), 1459 (m), 1422 (m), 1340 (s), 1263 (m), 1235 (m), 1192 (w), 1158 (s), 1115 (m), 1080 (m), 1013 (m), 971 (m), 800 (w), 755 (s), 736 (s), 701 (w).

$[\alpha]_D^{24}$ = -41 (*c* 0.25, $CHCl_3$).

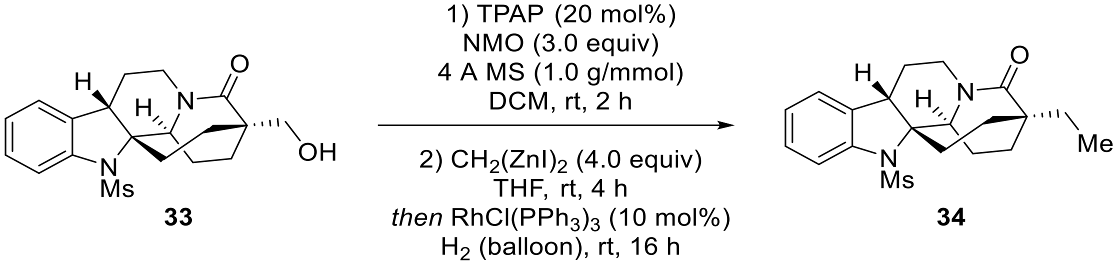

33 → **34**

To a solution of alcohol **33** (340 mg, 903 μmol, 1.0 equiv) in dry DCM (18 mL) were sequentially added 4 Å MS (903 mg, 1.0 g/mmol) and NMO (317 mg, 2.75 mmol, 3.0 equiv) at rt under Ar. The reaction mixture was stirred for 5 min before the addition of TPAP (63.4 mg, 180 μmol, 20 mol%) in one portion. The reaction mixture was then stirred at rt for 2 h. The solids were removed by filtration, and aqueous solutions of $NaHCO_3$ and $Na_2S_2O_3$ were added to the filtrate, which was extracted three times with EtOAc. The combined organic layers were washed with brine, dried over Na_2SO_4, filtered and concentrated under reduced pressure. The crude product was used directly in the next step without further purification.

A stock solution of $CH_2(ZnI)_2$ in THF was prepared according to reported procedure.[11] Titrations by iodolysis and methylenation of benzaldehyde showed a concentration of 0.6 M solution of $CH_2(ZnI)_2$ in THF with a 6:1 ratio of $CH_2(ZnI)_2/CH_3ZnI$.

Under argon, a freshly prepared solution of $CH_2(ZnI)_2$ (0.6 M in THF, 6.0 mL, 3.61 mmol, 4.0 equiv) was added dropwise to a solution of the crude aldehyde (338 mg, 903 μmol, 1.0 equiv) in dry THF (9 mL) at rt. The reaction mixture was stirred at rt for 4 h to complete the methylenation. Wilkinson catalyst (85 mg, 90 μmol, 10 mol%) was added and the mixture was subjected to three cycles of evacuation and hydrogen backfilling before being stirred at rt for 16 h under hydrogen atmosphere (balloon). The reaction mixture was then poured into a saturated aqueous solution of Na_2CO_3 and extracted three times with EtOAc. The combined organic layers were washed with brine, dried over Na_2SO_4, filtered and concentrated under reduced pressure. The crude product was purified by FCC (SiO_2, 3/2 EtOAc/Hexane) to give compound **34** (243 mg, 649 μmol, 72% over 2 steps) as a brown solid.

N.B.: For step 1, NMO and TPAP were stored and weighed in a glovebox. The 4 Å MS were activated in a 200 °C oven under vacuum for at least 24 h.

(3*R*,5a*S*,8a*S*,13a*R*)-3-ethyl-13-(methylsulfonyl)-1,2,3,4,5,5a,7,8,8a,13-decahydro-3,6-methanocyclohepta[2,3]pyrido[3,4-b]indol-14-one (34)

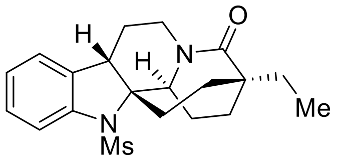

34

Brown solid.

Rf = 0.36 (5/9 EtOAc/Hexane).

^{1}H NMR (400 MHz, $CDCl_3$) δ 7.47 (d, *J* = 8.1 Hz, 1H), 7.22 (t, *J* = 8.1 Hz, 1H), 7.17 (d, *J* = 7.3 Hz, 1H), 7.07 (td, *J* = 7.4, 1.0 Hz, 1H), 4.49 (dt, *J* = 12.8, 7.3 Hz, 1H), 3.68 (dt, *J* = 7.2, 1.6 Hz, 1H), 3.11 – 3.06 (m, 1H), 3.03 (s, 3H), 2.86 – 2.73 (m, 2H), 2.60 (ddd, *J* = 14.9, 9.8, 5.7 Hz, 1H), 2.15 – 1.98 (m, 2H), 1.96 – 1.63 (m, 7H), 1.47 – 1.36 (m, 1H), 0.89 (t, *J* = 7.4 Hz, 3H).

^{13}C NMR (101 MHz, $CDCl_3$) δ 178.9, 141.9, 132.4, 128.5, 124.5, 124.3, 116.1, 77.7, 56.3, 46.0, 43.9, 41.0, 40.8, 33.3, 30.6, 29.6, 26.1, 24.9, 20.2, 8.9.

HRMS (ESI/QTOF) m/z: $[M + Na]^+$ Calcd for $C_{20}H_{26}N_2NaO_3S^+$ 397.1556; Found 397.1541.

IR (ν_{max}, cm^{-1}) 2930 (w), 2875 (w), 1652 (s), 1476 (m), 1458 (m), 1426 (m), 1347 (s), 1227 (m), 1197 (w), 1162 (s), 1115 (w), 1018 (w), 971 (m), 913 (w), 756 (m), 732 (m).

$[\alpha]_D^{24}$ = -59 (*c* 0.25, $CHCl_3$).

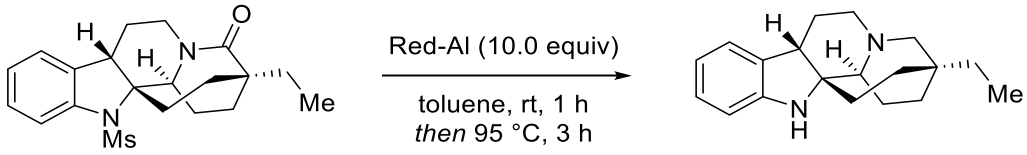

34 **1 (+)-melonine**

A solution of Red-Al (70% wt in toluene, 1.8 mL, 6.1 mmol, 10.0 equiv) was added dropwise to a stirred solution of amide **34** (230 mg, 614 µmol, 1.0 equiv) in dry toluene (6.1 mL) at 0 °C. The reaction mixture was stirred at rt for 1 h, then at 95 °C for 3 h. The reaction was cooled down to rt and quenched with saturated Rochelle's salt solution and stirred for 10 min. Aqueous saturated solution of Na_2CO_3 was added and the crude product was extracted five times with EtOAc. The combined organic layers were washed with brine, dried over Na_2SO_4, filtered and concentrated

under reduced pressure. The crude product was purified by FCC (SiO_2, 1/9 4 M NH_3 in MeOH/DCM) to give (+)-melonine (**1**) (165 mg, 584 µmol, 95%) as a white amorphous solid.

(+)-Melonine (**1**) (30.0 mg, 106 µmol, 1.0 equiv) was dissolved in a solution of TFA in MeOH (0.1 M in MeOH, 2.7 mL, 266 µmol, 2.5 equiv). After stirring at rt for 5 min, the solvent was removed under reduced pressure to give melonine·TFA salt (**1·TFA**) (42.1 mg, 106 µmol). A sample was recrystallized by slow open-air evaporation (with glass pipette friction and vial rotation) from MeOH/MeCN (1/4) to afford colorless needle-shaped crystals.

*N.B.: Significant variations in the ^{1}H and ^{13}C NMR data of the free base of (+)-melonine (**1**) in $CDCl_3$ have been observed among different samples.*

(+)-melonine (1)

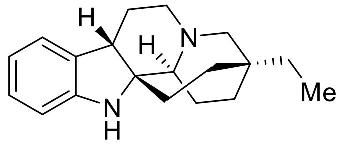

1 (+)-melonine

White amorphous solid.

Rf = 0.54 (1/9 MeOH/DCM).

^{1}H NMR (500 MHz, $CDCl_3$) δ 7.01 (t, J = 7.6, 1H), 6.99 (d, J = 7.2, 1H), 6.73 (t, J = 7.3 Hz, 1H), 6.55 (d, J = 7.7 Hz, 1H), 3.94 (br s, 1H), 3.17 (d, J = 5.4 Hz, 1H), 3.02 (d, J = 12.1 Hz, 1H), 2.98 (td, J = 13.4, 3.0 Hz, 1H), 2.86 (d, J = 5.6 Hz, 1H), 2.72 (dt, J = 13.8, 3.5 Hz, 1H), 2.52 (dd, J = 11.9, 2.8 Hz, 1H), 2.19 (tt, J = 13.0, 5.0 Hz, 1H), 2.11 – 2.01 (m, 2H), 1.91 – 1.78 (m, 3H), 1.68 – 1.52 (m, 3H), 1.35 (td, J = 12.7, 3.6 Hz, 1H), 1.25 (qd, J = 7.4, 1.9 Hz, 2H), 0.83 (t, J = 7.6 Hz, 3H).

^{13}C NMR (126 MHz, $CDCl_3$) δ 149.3, 130.6, 128.0, 122.7, 119.1, 109.7, 67.5, 58.9, 57.0, 48.7, 46.5, 35.7, 35.7, 35.1, 34.2, 26.6, 21.3, 20.0, 7.7.

HRMS (ESI/QTOF) m/z: $[M + H]^+$ Calcd for $C_{19}H_{27}N_2^+$ 283.2169; Found 283.2167.

IR (ν_{max}, cm^{-1}) 3355 (m), 2923 (s), 2857 (s), 1607 (m), 1482 (m), 1462 (s), 1298 (m), 1260 (m), 1134 (w), 1051 (w), 746 (s), 707 (m), 675 (m), 652 (m).

$[\alpha]_D^{24}$ = +49 (c 0.39, MeOH).

Table S6: ^{1}H NMR data for our synthetic (+)-melonine (**1**) ($CDCl_3$).

**Proton**	**our synthetic δH (ppm, multi, *J* (Hz)) $CDCl_3$, 500 MHz**
**11**	7.01 (1H, t, 7.6)
**9**	6.99 (1H, d, 7.2)
**10**	6.73 (1H, t, 7.3)
**12**	6.55 (1H, d, 7.7)
**1**	3.94 (1H, br s)
**7**	3.17 (1H, d, 5.4)
**21**	3.02 (1H, d, 12.1)
**5**	2.98 (1H, td, 13.4, 3.0)
**3**	2.86 (1H, d, 5.6)
**5’**	2.72 (1H, dt, 13.8, 3.5)
**21’**	2.52 (1H, dd, 11.9, 2.8)
**6**	2.19 (1H, tt, 13.0, 5.0)
**14**	2.11–2.01 (2H, m)
**6’**	
**16**	1.91–1.78 (m, 3H)
**14’**	
**17**	1.68-1.52 (3H, m)
**17’**	
**15**	
**15’**	1.35 (1H, td, 12.7, 3.6)
**19**	1.25 (2H, qd, *J* = 7.4, 1.9)
**18**	0.83 (3H, t, 7.6)

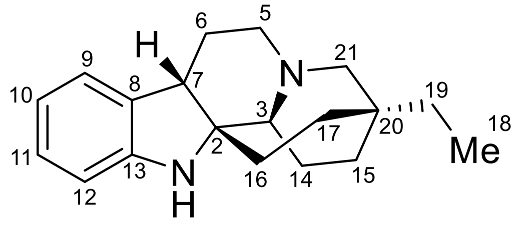

1 (+)-melonine

Table S7: ^{13}C NMR data for natural,[12,13] and our synthetic (+)-melonine (**1**) ($CDCl_3$).

Carbon	natural<br>δC (ppm), $CDCl_3$	our synthetic<br>δC (ppm), $CDCl_3$, 126 MHz	Δ (ppm)
**13**	149.4 (C)	149.3	-0.1
**8**	128.8 (C)	130.6	+1.8
**11**	128.4 (CH)	128.0	-0.4
**9**	122.6 (CH)	122.7	+0.1
**10**	118.4 (CH)	119.1	+0.7
**12**	109.5 (CH)	109.7	+0.2
**2**	66.8 (C)	67.5	+0.7
**21**	57.2 (CH_2)	58.9	+1.7
**3**	57.2 (CH)	57.0	-0.2
**5**	48.0 (CH_2)	48.7	+0.7
**7**	45.1 (CH)	46.5	+1.4
**19**	35.1 (CH_2)	35.7	+0.6
**20**	34.4 (C)	35.7	+1.3
**16**	33.9 (CH_2)	35.1	+1.2
**17**	33.2 (CH_2)	34.2	+1.0
**15**	24.9 (CH_2)	26.6	+1.7
**14**	18.4 (CH_2)	21.3	+2.9
**6**	18.4 (CH_2)	20.0	+1.6
**18**	7.4 (CH_3)	7.7	+0.3

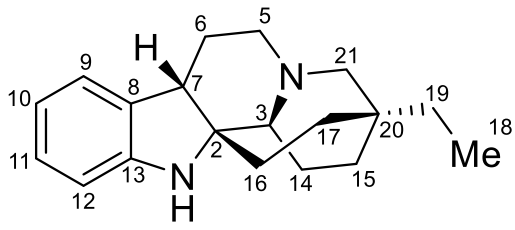

1 (+)-melonine

(+)-melonine·TFA salt (1·TFA salt)

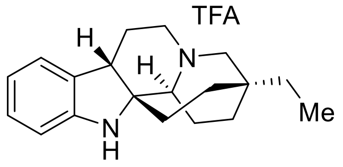

1·TFA (+)-melonine·TFA salt

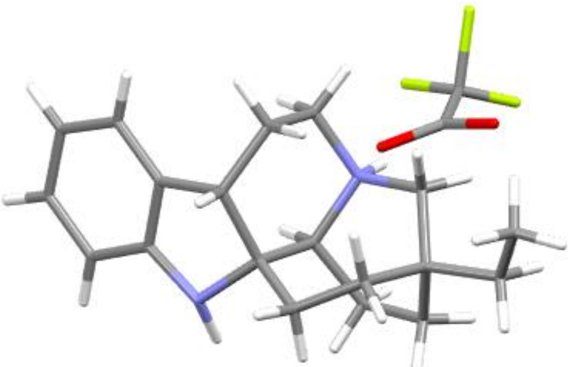

Colorless needle-shaped crystals.

X-Ray structure of 1·TFA.

^{1}H NMR (600 MHz, CD_3CN) δ 10.30 (s, 1H), 7.07 (dt, *J* = 7.4, 1.5 Hz, 1H), 7.05 (tt, *J* = 7.7, 1.2 Hz, 1H), 6.75 (td, *J* = 7.4, 1.0 Hz, 1H), 6.58 (dt, *J* = 7.7, 1.0 Hz, 1H), 3.24 (t, *J* = 4.1 Hz, 1H), 3.21 (dd, *J* = 13.3, 8.3 Hz, 1H), 3.06 – 2.98 (m, 2H), 2.95 (dt, *J* = 12.9, 4.2 Hz, 1H), 2.89 (dt, *J* = 13.2, 3.1 Hz, 1H), 2.39 – 2.36 (m, 2H), 2.09 (dddd, *J* = 15.7, 12.0, 3.9, 1.5 Hz, 1H), 2.05 – 1.99 (m, 1H), 1.99 – 1.94 (m, 1H), 1.90 (dtd, *J* = 14.6, 4.1, 1.4 Hz, 1H), 1.73 – 1.67 (m, 3H), 1.36 (ddd, *J* = 14.3, 11.9, 3.8 Hz, 1H), 1.32 (q, *J* = 7.6 Hz, 2H), 0.85 (t, *J* = 7.6 Hz, 3H).

^{13}C NMR (151 MHz, CD_3CN) δ 149.8, 130.2, 129.3, 123.8, 119.9, 110.8, 67.9, 58.6, 58.5, 49.3, 45.8, 35.9, 35.3, 34.6, 33.9, 25.6, 19.8, 19.2, 7.8.

HRMS (ESI/QTOF) m/z: $[M + H]^+$ Calcd for $C_{19}H_{27}N_2^+$ 283.2169; Found 283.2167.

IR (ν_{max}, cm^{-1}) 3383 (w), 2924 (m), 2854 (m), 1680 (s), 1462 (m), 1202 (s), 1187 (s), 1133 (s), 950 (w), 839 (m), 801 (m), 723 (m).

Mp = 180 – 182 °C.

$[\alpha]_D^{24}$ = +20.4 (*c* 0.48, MeOH). (lit.[13] **$[\alpha]_D^{24}$** = +20.8 (*c* 0.48, MeOH)).

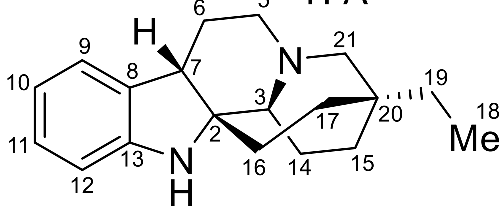

1·TFA (+)-melonine·TFA

Table S8: ^{1}H NMR data for natural,[13] Yokoshima's synthetic,[14] and our synthetic (+)-melonine.TFA (**1·TFA**) (CD_3CN).

Proton	natural δH (ppm, multi, *J*, Hz) CD_3CN, 700 MHz	reported synthetic δH (ppm, multi, *J*, Hz) CD_3CN, 400 MHz	our synthetic δH (ppm, multi, *J*, Hz) CD_3CN, 600 MHz
**-**	-	11.21 (1H, br s)	10.30 (1H, br s)
**9**	7.07 (1H, dt, 7.4, 1.2)	7.07 (1H, d, 6.8)	7.07 (1H, dt, 7.4, 1.5)
**11**	7.04 (1H, td, 7.7, 1.0)	7.04 (1H, t, 7.2)	7.05 (1H, tt, 7.7, 1.2)
**10**	6.74 (1H, td, 7.5, 0.9)	6.74 (1H, t, 7.2)	6.75 (1H, td, 7.4, 1.0)
**12**	6.57 (1H, dt, 7.8, 1.0)	6.57 (1H, d, 7.2)	6.58 (1H, dt, 7.7, 1.0)
**7**	3.27 (1H, overlapped)	3.27-3.22 (2H, m)	3.24 (1H, t, 4.1)
**21**	3.24 (1H, overlapped)		3.21 (1H, dd, 13.3, 8.3)
**5**	3.07 (1H, overlapped)	3.07 (1H, m)	3.06 – 2.98 (1H, m)
**3**	3.04 (1H, overlapped)	3.05 (1H, m)	3.06 – 2.98 (1H, m)
**5**	2.96 (1H, dt, 13.4, 3.5)	2.97 (1H, m)	2.95 (1H, dt, 12.9, 4.2)
**21**	2.88 (1H, dd, 13.1, 2.8)	2.90 (1H, m)	2.89 (1H, dt, 13.2, 3.1)
**6**	2.37 (2H, m)	2.38 (2H, m)	2.39-2.36 (2H, m)
**14**	2.07 (1H, ddd, 14.3, 4.2, 1.3)	2.08 (1H, m)	2.09 (1H, dddd, 15.7, 12.0, 3.9, 1.5)
**14**	2.03 (1H, m)	2.01-1.88 (3H, m)	2.05 – 1.99 (1H, m)
**16**	2.00 (1H, m)		1.99 – 1.94 (1H, m)
**16**	1.91 (1H, dtd, 13.6, 4.0, 1.3)		1.90 (1H, dtd, 14.6, 4.1, 1.4)
**17**	1.72 (2H, overlapped)	1.75-1.67 (3H, m)	1.73 – 1.67 (3H, m)
**15**	1.70 (1H, m)		
**15**	1.39 (1H, ddd, 14.0, 4.2, 3.3)	1.39 (1H, m)	1.36 (1H, ddd, 14.3, 11.9, 3.8)
**19**	1.33 (2H, q, 7.7)	1.34 (2H, q, 7.6)	1.32 (2H, q, 7.6)
**18**	0.86 (3H, t, 7.7)	0.86 (3H, t, 7.6)	0.85 (3H, t, 7.6)

Table S9: ^{13}C NMR data of the natural,[13] Yokoshima's synthetic,[14] and our synthetic (+)-melonine.TFA (**1·TFA**) (CD_3CN).

Carbon	natural<br>*δ*C (ppm)<br>CD_3CN, 176 MHz	reported synthetic<br>*δ*C (ppm)<br>CD_3CN, 100 MHz	our synthetic<br>*δ*C (ppm)<br>CD_3CN, 151 MHz
**13**	150.1 (C)	150.0 (C)	149.8 (C)
**8**	130.3 (C)	130.2 (C)	130.2 (C)
**11**	129.2 (CH)	129.2 (CH)	129.3 (CH)
**9**	123.7 (CH)	123.7 (CH)	123.8 (CH)
**10**	119.7 (CH)	119.7 (CH)	119.9 (CH)
**12**	110.6 (CH)	110.6 (CH)	110.8 (CH)
**2**	67.9 (C)	67.9 (C)	67.9 (C)
**21**	58.2 (CH_2)	58.4 (CH_2)	58.6 (CH_2)
**3**	58.0 (CH)	58.2 (CH)	58.5 (CH)
**5**	48.8 (CH_2)	49.0 (CH_2)	49.3 (CH_2)
**7**	46.0 (CH)	45.8 (CH)	45.8 (CH)
**19**	36.0 (CH_2)	35.9 (CH_2)	35.9 (CH_2)
**20**	35.3 (C)	35.3 (C)	35.3 (C)
**16**	34.7 (CH_2)	34.7 (CH_2)	34.6 (CH_2)
**17**	34.0 (CH_2)	33.9 (CH_2)	33.9 (CH_2)
**15**	25.8 (CH_2)	25.7 (CH_2)	25.6 (CH_2)
**14**	19.7 (CH_2)	19.8 (CH_2)	19.8 (CH_2)
**6**	19.2 (CH_2)	19.2 (CH_2)	19.2 (CH_2)
**18**	7.8 (CH_3)	7.8 (CH_3)	7.8 (CH_3)

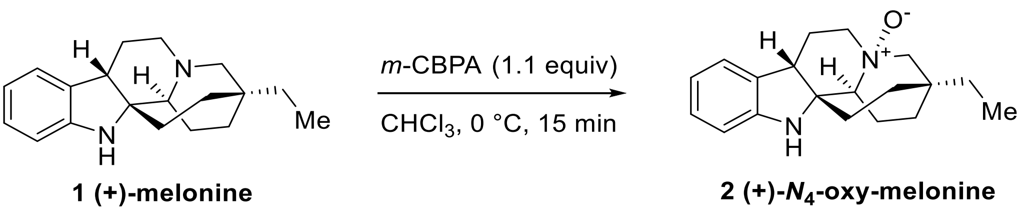

Under argon, a solution of *m*-CPBA (≤ 77% wt, 26.2 mg, 117 µmol, 1.1 equiv) in $CHCl_3$ (1.0 mL) was added dropwise to a stirred solution of (+)-melonine (**1**) (30.0 mg, 106 µmol, 1.0 equiv) in $CHCl_3$ (1.0 mL) at 0 °C. The reaction mixture was stirred at 0 °C for 15 min. The reaction was poured into a saturated aqueous solution of Na_2CO_3 and the crude product was extracted three times with $CHCl_3$. The combined organic layers were washed with brine, dried over Na_2SO_4, filtered and concentrated under reduced pressure. The crude product was purified by FCC (SiO_2, 1/4 MeOH/DCM) to give compound (+)-N_4-oxy melonine (**2**) (31.5 mg, 106 µmol, 99%) as colorless to pale-yellow crystals.

*N.B.: Similarly to (+)-melonine (**1**), albeit to an even lesser extent, some variations in the ^1H and ^{13}C NMR data of (+)-N_4-oxy melonine (**2**) in $CDCl_3$ have been observed among different samples. Notably, one of the two H14 appeared as a broad signal.*

(+)-N_4-oxy-melonine (2)

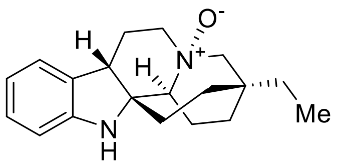

2 (+)-N_4-oxy-melonine

Colorless to pale yellow crystals.

Rf = 0.32 (1/9 MeOH/DCM).

^{1}H NMR (800 MHz, $CDCl_3$) δ 7.01 (t, *J* = 7.6 Hz, 1H), 6.97 (d, *J* = 7.3 Hz, 1H), 6.72 (t, *J* = 7.4 Hz, 1H), 6.63 (d, *J* = 7.6 Hz, 1H), 4.38 (br s, 1H), 3.59 (d, *J* = 13.4 Hz, 1H), 3.47 – 3.41 (m, 2H), 3.29 (t, *J* = 13.3 Hz, 1H), 3.18 (d, *J* = 5.1 Hz, 1H), 3.05 (d, *J* = 5.5 Hz, 1H), 2.90 – 2.65 (m, 1H), 2.44 (d, *J* = 15.8 Hz, 1H), 2.23 (tt, *J* = 14.5, 4.1 Hz, 1H), 2.05 (td, *J* = 14.5, 6.1 Hz, 1H), 1.94 (dd, *J* = 15.8, 3.9 Hz, 1H), 1.78 (t, *J* = 12.7 Hz, 1H), 1.68 – 1.60 (m, 3H), 1.53 (td, *J* = 14.6, 4.8 Hz, 1H), 1.26 (q, *J* = 7.6 Hz, 2H), 0.82 (t, *J* = 7.5 Hz, 3H).

^{13}C NMR (201 MHz, $CDCl_3$) δ 148.7, 129.2, 128.4, 122.4, 119.4, 110.6, 78.3, 73.9, 69.0, 65.5, 44.8, 35.9, 35.0, 34.2, 34.2, 24.9, 23.4, 16.5, 7.6.

HRMS (ESI/QTOF) m/z: $[M + H]^+$ Calcd for $C_{19}H_{27}N_2O^+$ 299.2118; Found 299.2117.

IR (ν_{max}, cm^{-1}) 3247 (w), 2922 (m), 2856 (w), 1608 (w), 1464 (m), 1261 (w), 1103 (w), 1040 (w), 1018 (w), 972 (w), 909 (m), 828 (w), 802 (w), 729 (s), 643 (w).

Mp = 197 – 198 °C. (lit.[12] **Mp** = 198 °C).

$[\alpha]_D^{24}$ = +88 (*c* 0.37, $CDCl_3$). (lit.[12] $[\alpha]_{578}^{22}$ = +110 (*concentration and solvent were not specified*)).

Table S10: ^{1}H NMR data for our synthetic (+)-N_4-oxy-melonine (**2**) ($CDCl_3$).

Proton	our synthetic δH (ppm, multi, *J* (Hz)), $CDCl_3$, 800 MHz
**11**	7.01 (1H, t, 7.6)
**9**	6.97 (1H, d, 7.3)
**10**	6.72 (1H, t, 7.4)
**12**	6.63 (1H, d, 7.3)
**1**	4.38 (1H, br s)
**21**	3.59 (1H, d, 13.4)
**21’**	3.47 – 3.41 (2H, m)
**5**	
**5’**	3.29 (1H, t, 13.3)
**7**	3.18 (1H, d, 5.1)
**3**	3.05 (1H, d, 5.5)
**14**	2.90 – 2.65 (1H, m)
**6**	2.44 (1H, d, 15.8)
**6’**	2.23 (1H, tt, 14.5, 4.1)
**16**	2.05 (1H, td, 14.5, 6.1)
**16’**	1.94 (1H, dd, 15.8, 3.9)
**14’**	1.78 (1H, t, 12.7)
**15**	1.68 – 1.60 (3H, m)
**17**	
**17’**	1.53 (1H, td, 14.6, 4.8)
**19**	1.26 (2H, q, *J* = 7.6)
**18**	0.82 (3H, t, 7.5)

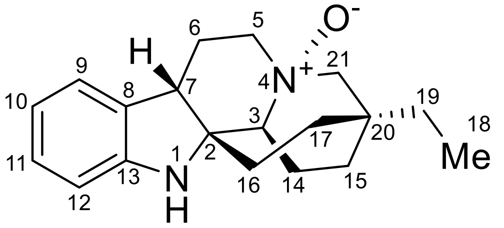

2 (+)-N_4-oxy melonine

Table S11: ^{13}C NMR data for natural,[12,13] and our synthetic (+)-N_4-oxy-melonine (**2**) ($CDCl_3$).

Carbon	natural<br>δC (ppm), $CDCl_3$	our synthetic<br>δC (ppm), $CDCl_3$, 201 MHz	Δ (ppm)
**13**	148.5 (C)	148.7 (C)	+0.2
**8**	129.3 (C)	129.2 (C)	-0.1
**11**	128.4 (CH)	128.4 (CH)	0
**9**	122.4 (CH)	122.4 (CH)	0
**10**	119.5 (CH)	119.4 (CH)	-0.1
**12**	110.3 (CH)	110.6 (CH)	+0.3
**21**	78.3 (CH_2)	78.3 (CH_2)	0
**3**	73.9 (CH)	73.9 (CH)	+0.1
**2**	68.9 (C)	69.0 (C)	+0.1
**5**	65.6 (CH_2)	65.5 (CH_2)	-0.1
**7**	44.8 (CH)	44.8 (CH)	0
**19**	35.8 (CH_2)	35.9 (CH_2)	+0.1
**16**	35.0 (CH_2)	35.0 (CH_2)	0
**20**	34.2 (C)	34.2 (C)	0
**17**	34.2 (CH_2)	34.2 (CH_2)	0
**15**	24.9 (CH_2)	24.9 (CH_2)	0
**6**	23.4 (CH_2)	23.4 (CH_2)	0
**14**	16.4 (CH_2)	16.5 (CH_2)	+0.1
**18**	7.5 (CH_3)	7.6 (CH_3)	+0.1

4) References

[1] S. O'Brien, D. C. C. Smith, "892. The Reduction of Indole and Carbazole by Metal–Ammonia Solutions", *J. Chem. Soc.* **1960**, *0*, 4609–4612. https://doi.org/10.1039/JR9600004609.

[2] W. A. Remers, G. J. Gibs, C. Pidacks, M. J. Weiss, "Reduction of Nitrogen Heterocycles by Lithium in Liquid Ammonia. III. Indoles and Quinolines", *J. Org. Chem.* **1971**, *36*, 279–284. https://doi.org/10.1021/jo00801a009.

[3] J. Lévy, P. Maupérin, M. D. de Maindreville, J. Le Men, "Methylene-Indolines, Indolenines et Indoleniniums-III(1). Action de Reactifs Reducteurs 3. Hemisynthese de La (+)-Vallesamidine", *Tetrahedron Lett.* **1971**, *12*, 1003–1006. https://doi.org/10.1016/S0040-4039(01)96610-9.

[4] X. Zeng, D. L. Boger, "Total Synthesis of (−)-Strempeliopine", *J. Am. Chem. Soc.* **2021**, *143*, 12412–12417. https://doi.org/10.1021/jacs.1c06913.

[5] H. Liu, W. Yuan, M.-Y. Ran, G. Wei, Y. Zhao, Z.-Q. Liao, H. Liang, Z.-F. Chen, F.-X. Wang, "Total Synthesis of Quebrachamine and Kopsiyunnanine D", *J. Org. Chem.* **2024**, *89*, 5905–5910. https://doi.org/10.1021/acs.joc.4c00363.

[6] G. K. Zieliński, C. Samojłowicz, T. Wdowik, K. Grela, "In Tandem or Alone: A Remarkably Selective Transfer Hydrogenation of Alkenes Catalyzed by Ruthenium Olefin Metathesis Catalysts", *Org. Biomol. Chem.* **2015**, *13*, 2684–2688. https://doi.org/10.1039/C4OB02480J.

[7] A. A. Scholte, M. H. An, M. L. Snapper, "Ruthenium-Catalyzed Tandem Olefin Metathesis−Oxidations", *Org. Lett.* **2006**, *8*, 4759–4762. https://doi.org/10.1021/ol061837n.

[8] L. Anhäuser, F. Muttach, A. Rentmeister, "Reversible Modification of DNA by Methyltransferase-Catalyzed Transfer and Light-Triggered Removal of Photo-Caging Groups", *Chem. Commun.* **2018**, *54*, 449–451. https://doi.org/10.1039/C7CC08300A.

[9] G. Sirvinskaite, C. S. Nardo, P. Müller, A. C. Gasser, B. Morandi, "Direct Synthesis of Unprotected Indolines Through Intramolecular Sp3 C−H Amination Using Nitroarenes as Aryl Nitrene Precursors", *Chem. Eur. J.* **2023**, *29*, e202301978. https://doi.org/10.1002/chem.202301978.

[10] P. Kumar, S. Verma, K. Rathi, D. Chandra, V. Prakash Verma, J. L. Jat, "Metal-Free Direct Transformation of Aryl Boronic Acid to Primary Amines", *Eur. J. Org. Chem.* **2022**, *2022*, e202200508. https://doi.org/10.1002/ejoc.202200508.

[11] R. Maazaoui, M. Pin-Nó, K. Gervais, R. Abderrahim, F. Ferreira, A. Perez-Luna, F. Chemla, O. Jackowski, "Domino Methylenation/Hydrogenation of Aldehydes and Ketones by Combining Matsubara's Reagent and Wilkinson's Catalyst", *Eur. J. Org. Chem.* **2016**, *2016*, 5732–5737. https://doi.org/10.1002/ejoc.201601137.

[12] S. Baassou, H. M. Mehri, A. Rabaron, M. Plat, "(+) Melonine and N_B-Oxy Melonine, a New Indoline Skeleton", *Tetrahedron Lett.* **1983**, *24*, 761–762. https://doi.org/10.1016/S0040-4039(00)81519-1.

[13] T. Kouamé, G. Bernadat, V. Turpin, M. Litaudon, A. T. Okpekon, J.-F. Gallard, K. Leblanc, S. Rharrabti, P. Champy, E. Poupon, M. A. Beniddir, P. Le Pogam, "Structure Reassignment of Melonine and Quantum-Chemical Calculations-Based Assessment of Biosynthetic Scenarios Leading to Its Revised and Original Structures", *Org. Lett.* **2021**, *23*, 5964–5968. https://doi.org/10.1021/acs.orglett.1c02055.

[14] Y. Matsuyuki, N. Umekubo, S. Yokoshima, "Total Synthesis of Melonine", *Org. Lett.* **2025**, *27*, 2065–2068. https://doi.org/10.1021/acs.orglett.4c04851.

5) Copies of NMR spectra

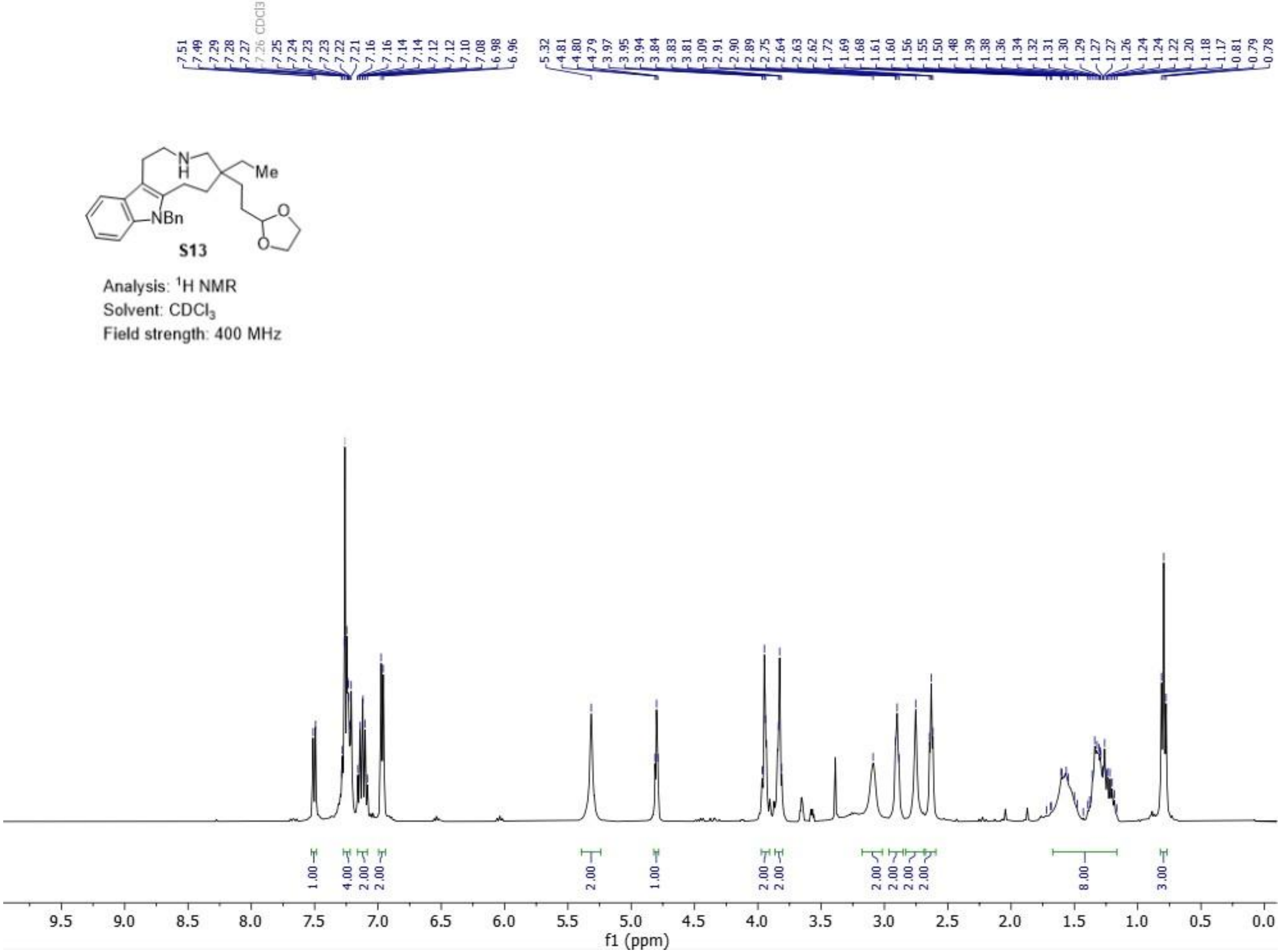

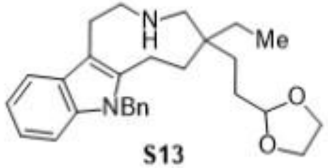

S13

Analysis: ^{13}C NMR
Solvent: $CDCl_3$
Field strength: 101 MHz

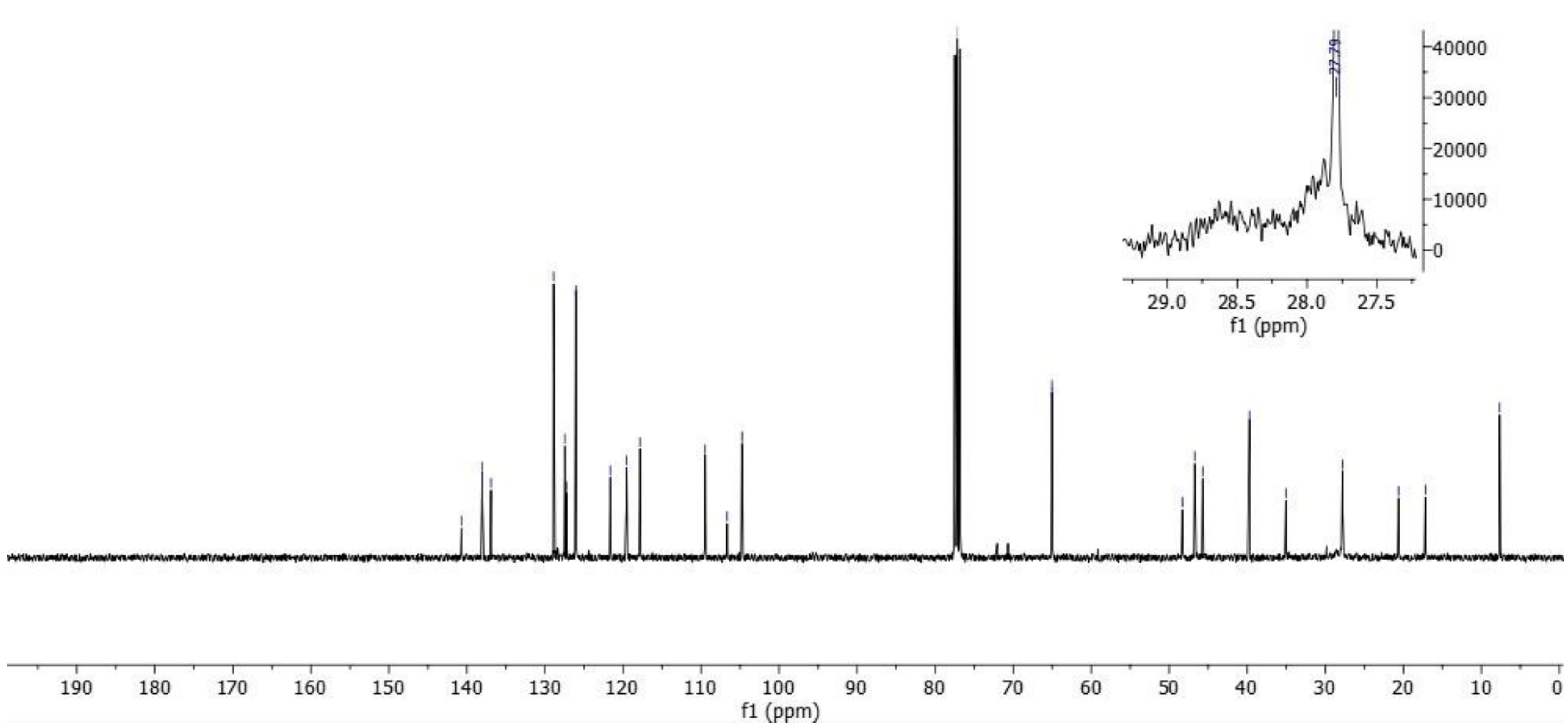

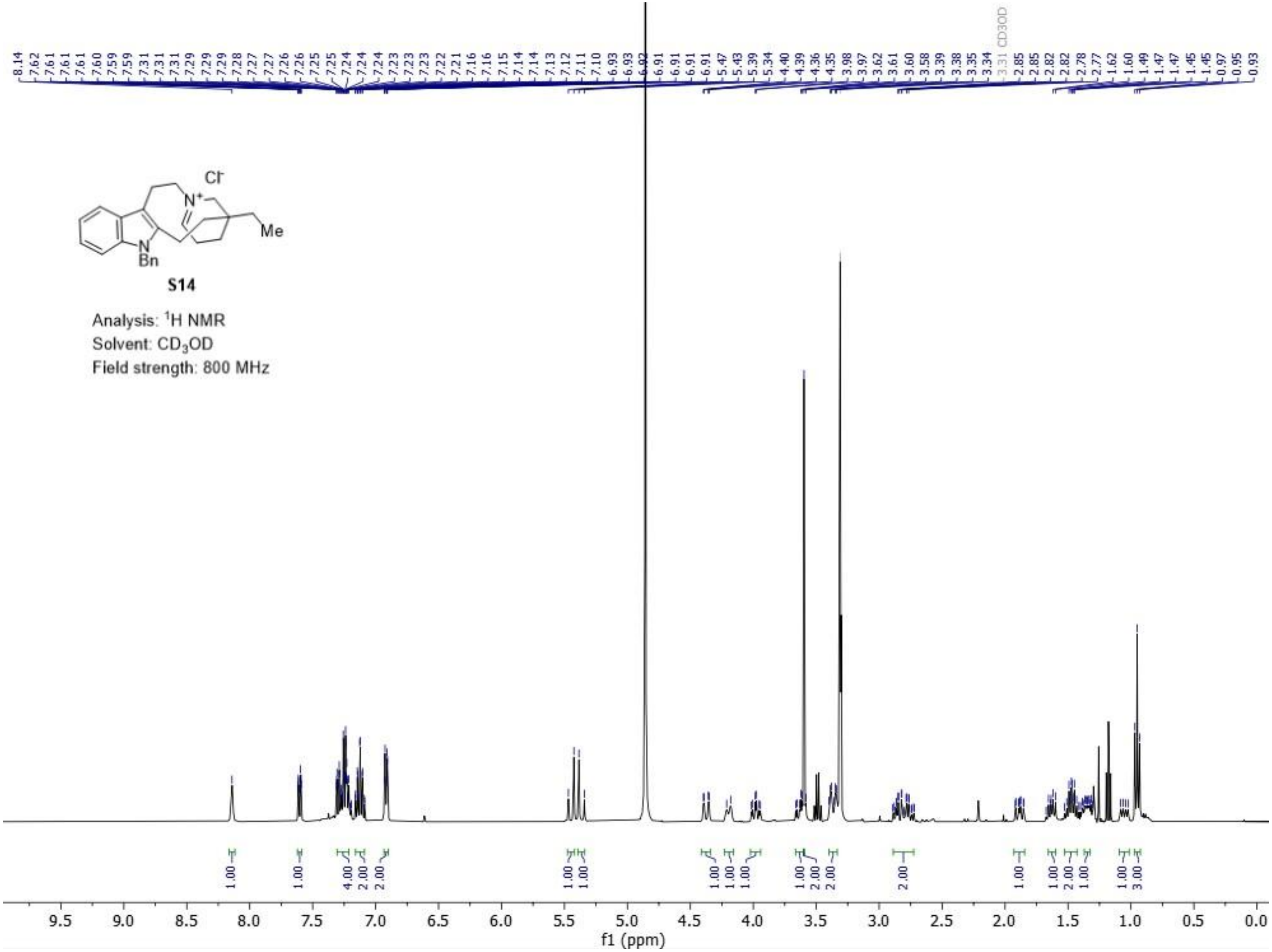
8.14 7.62 7.61 7.61 7.61 7.60 7.59 7.59 7.31 7.31 7.31 7.29 7.29 7.29 7.28 7.27 7.27 7.26 7.25 7.25 7.24 7.24 7.24 7.23 7.23 7.22 7.21 7.16 7.16 7.15 7.14 7.14 7.13 7.12 7.11 7.10 6.93 6.93 6.92 6.91 6.91 6.91 5.47 5.43 5.39 5.34 4.40 4.39 4.36 4.35 3.98 3.97 3.62 3.61 3.60 3.58 3.39 3.38 3.35 3.34 3.31 2.85 2.85 2.82 2.82 2.78 2.77 1.62 1.60 1.49 1.47 1.45 1.45 0.97 0.95 0.93
CD3OD
Cl⁻
Me
N
Bn
S14
Analysis: ¹H NMR
Solvent: CD₃OD
Field strength: 800 MHz
1.00 1.00 4.00 2.00 2.00 1.00 1.00 1.00 1.00 1.00 1.00 2.00 2.00 2.00 1.00 1.00 2.00 1.00 1.00 3.00
9.5 9.0 8.5 8.0 7.5 7.0 6.5 6.0 5.5 5.0 4.5 4.0 3.5 3.0 2.5 2.0 1.5 1.0 0.5 0.0
f1 (ppm)

S14

Analysis: ^{13}C NMR
Solvent: CD_3OD
Field strength: 201 MHz

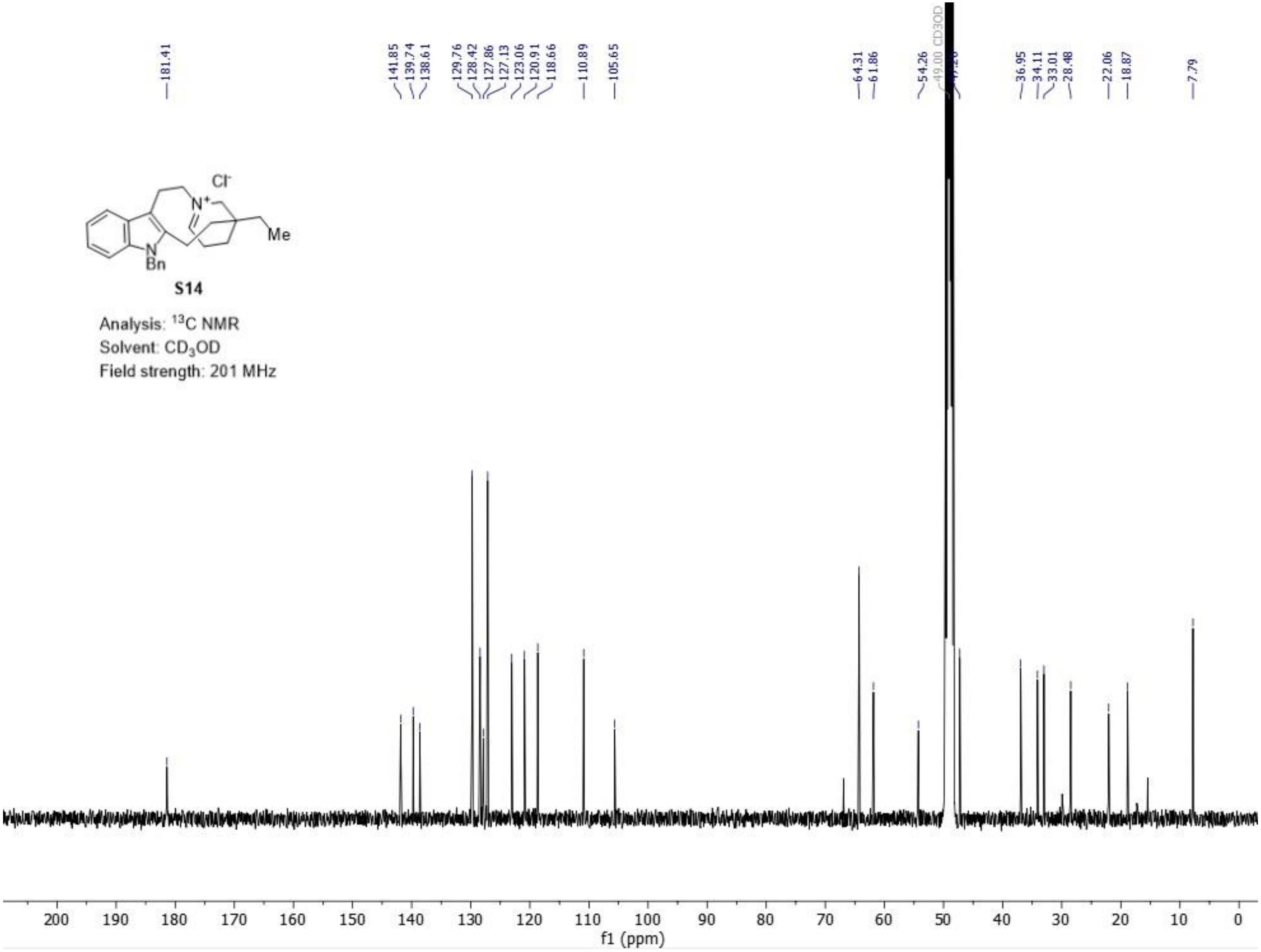

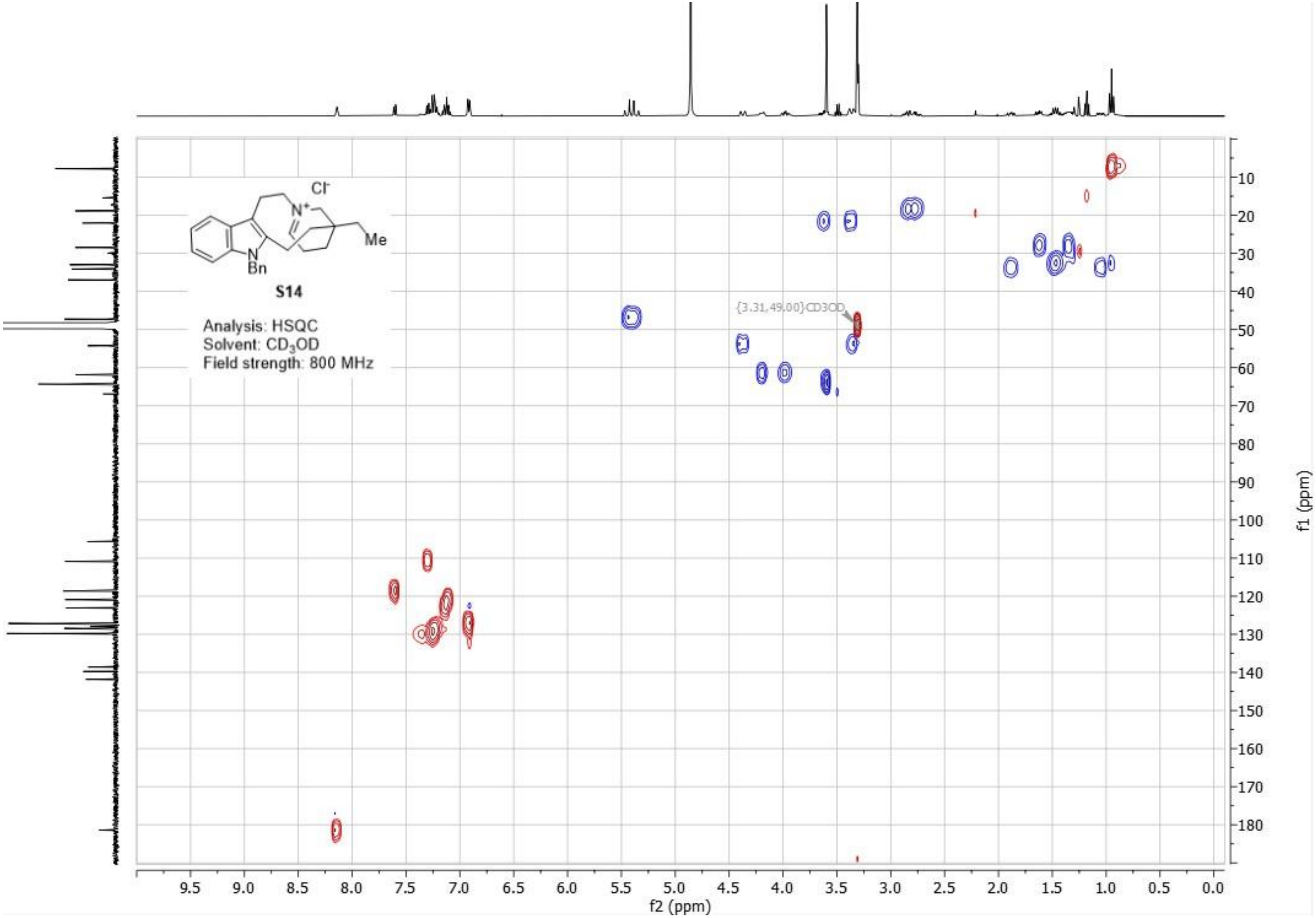

Cl⁻
N⁺
Me
N
Bn
S14
Analysis: HSQC
Solvent: CD3OD
Field strength: 800 MHz
{3.31,49.00}CD3OD
f2 (ppm)
f1 (ppm)

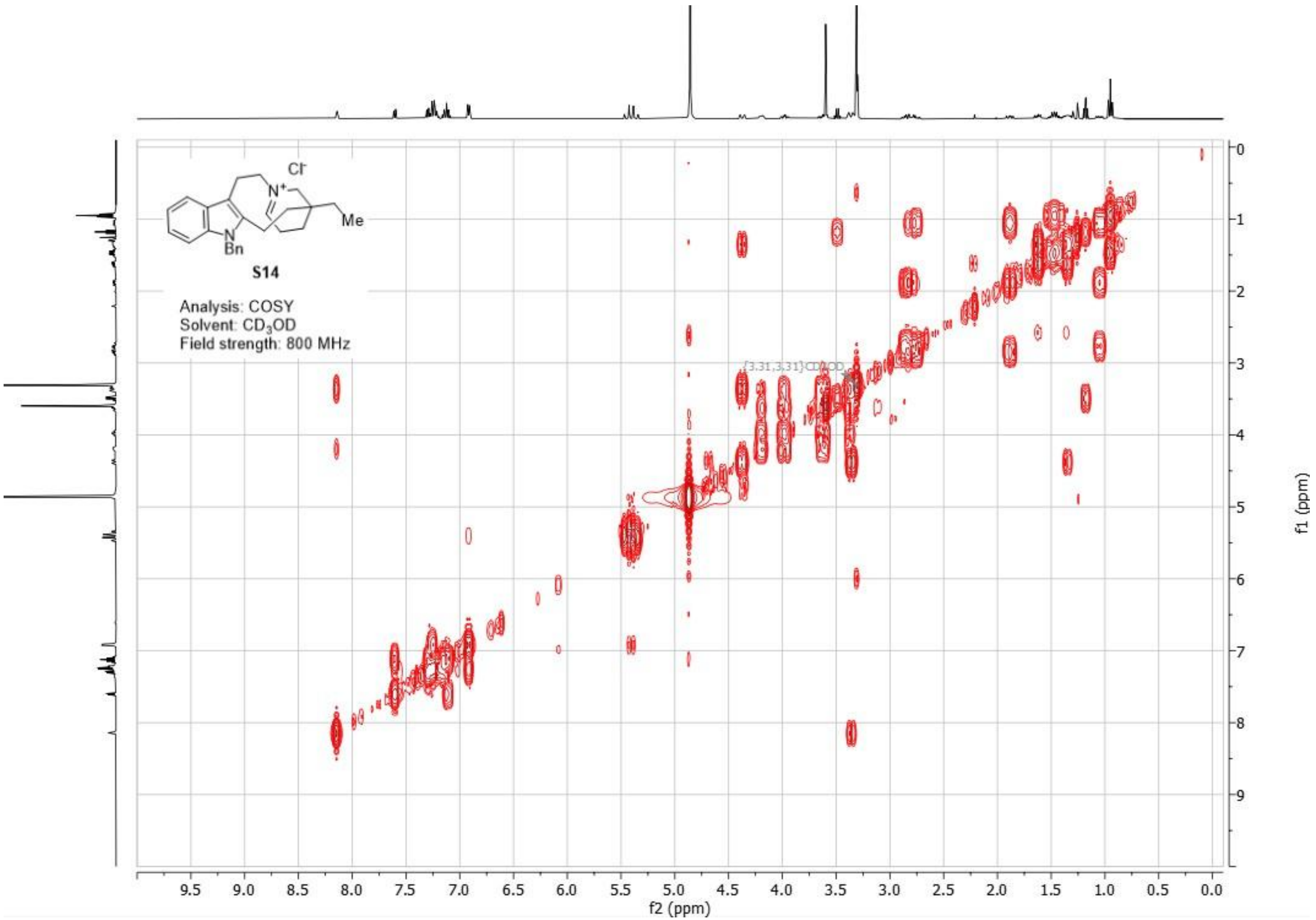
Cl⁻
N⁺
Me
N
Bn
S14
Analysis: COSY
Solvent: CD3OD
Field strength: 800 MHz
f1 (ppm)
f2 (ppm)

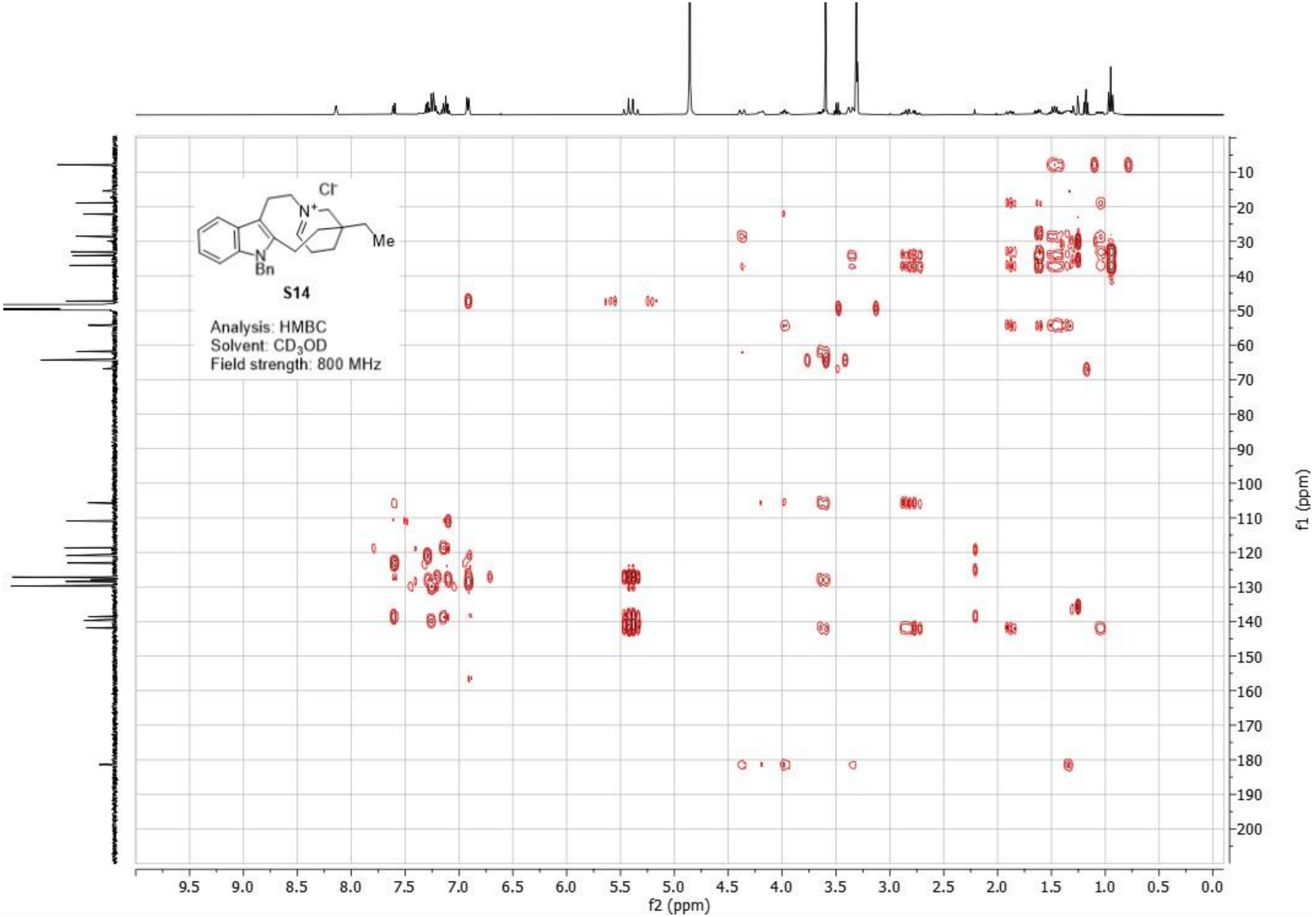

Cl⁻
N⁺
N
Bn
Me
S14
Analysis: HMBC
Solvent: CD3OD
Field strength: 800 MHz
f2 (ppm)
f1 (ppm)

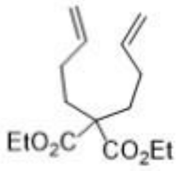

19

Analysis: ^{1}H NMR
Solvent: $CDCl_3$
Field strength: 400 MHz

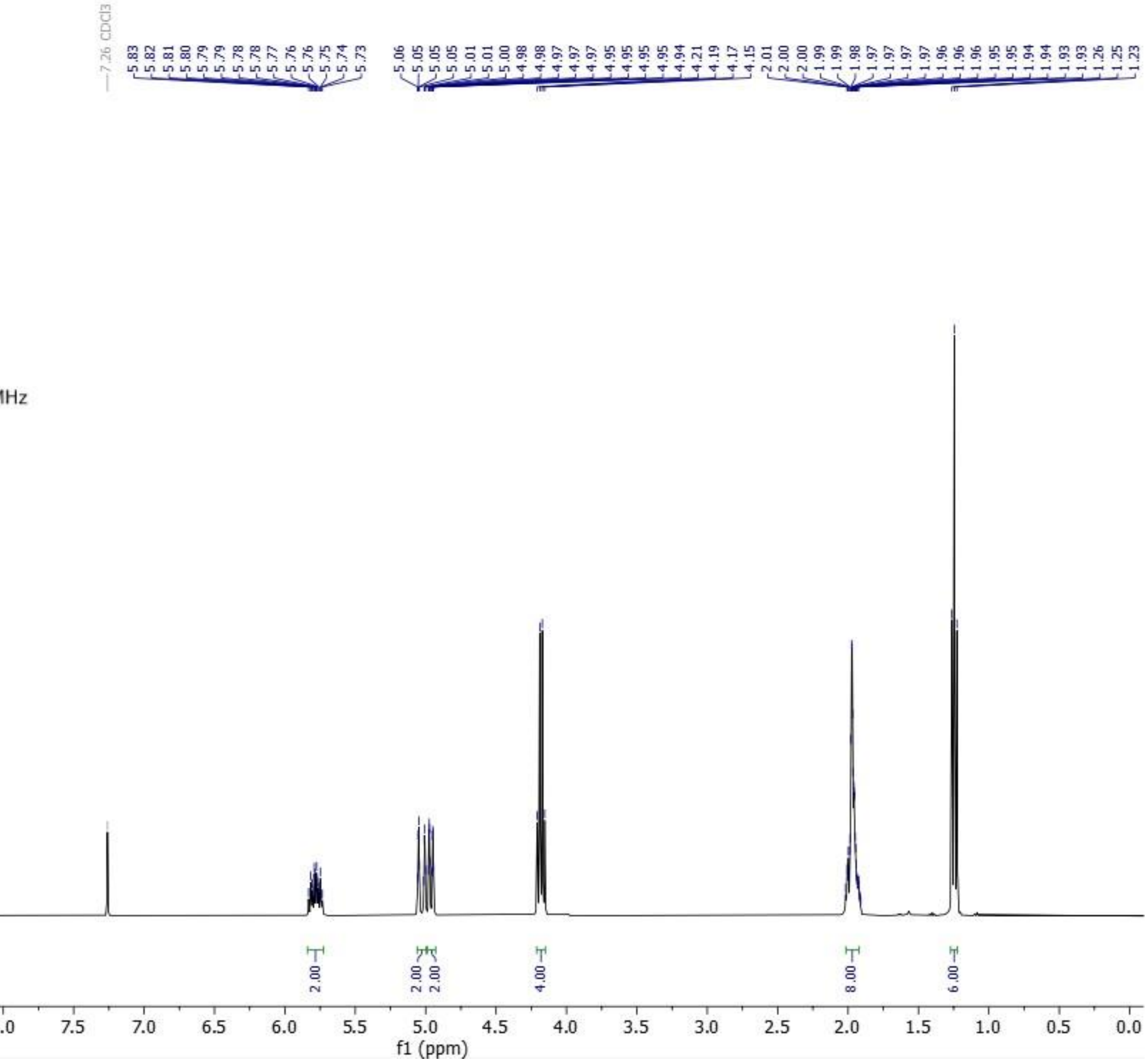

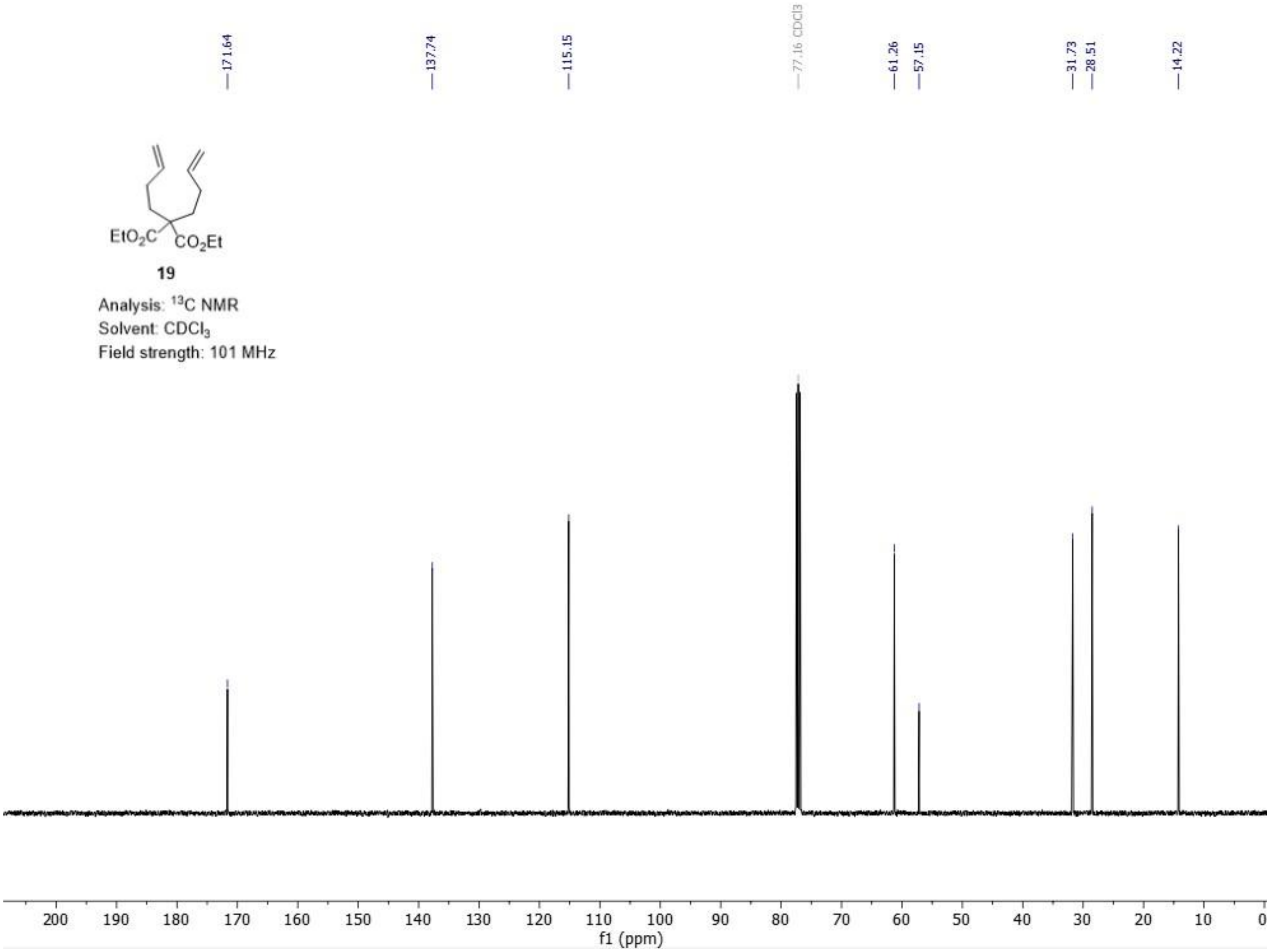

171.64
137.74
115.15
77.16 CDCl3
61.26
57.15
31.73
28.51
14.22
EtO2C
CO2Et
19
Analysis: 13C NMR
Solvent: CDCl3
Field strength: 101 MHz
200 190 180 170 160 150 140 130 120 110 100 90 80 70 60 50 40 30 20 10 0
f1 (ppm)

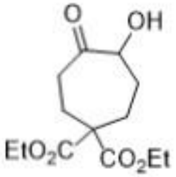

20

Analysis: ^{1}H NMR
Solvent: $CDCl_3$
Field strength: 400 MHz

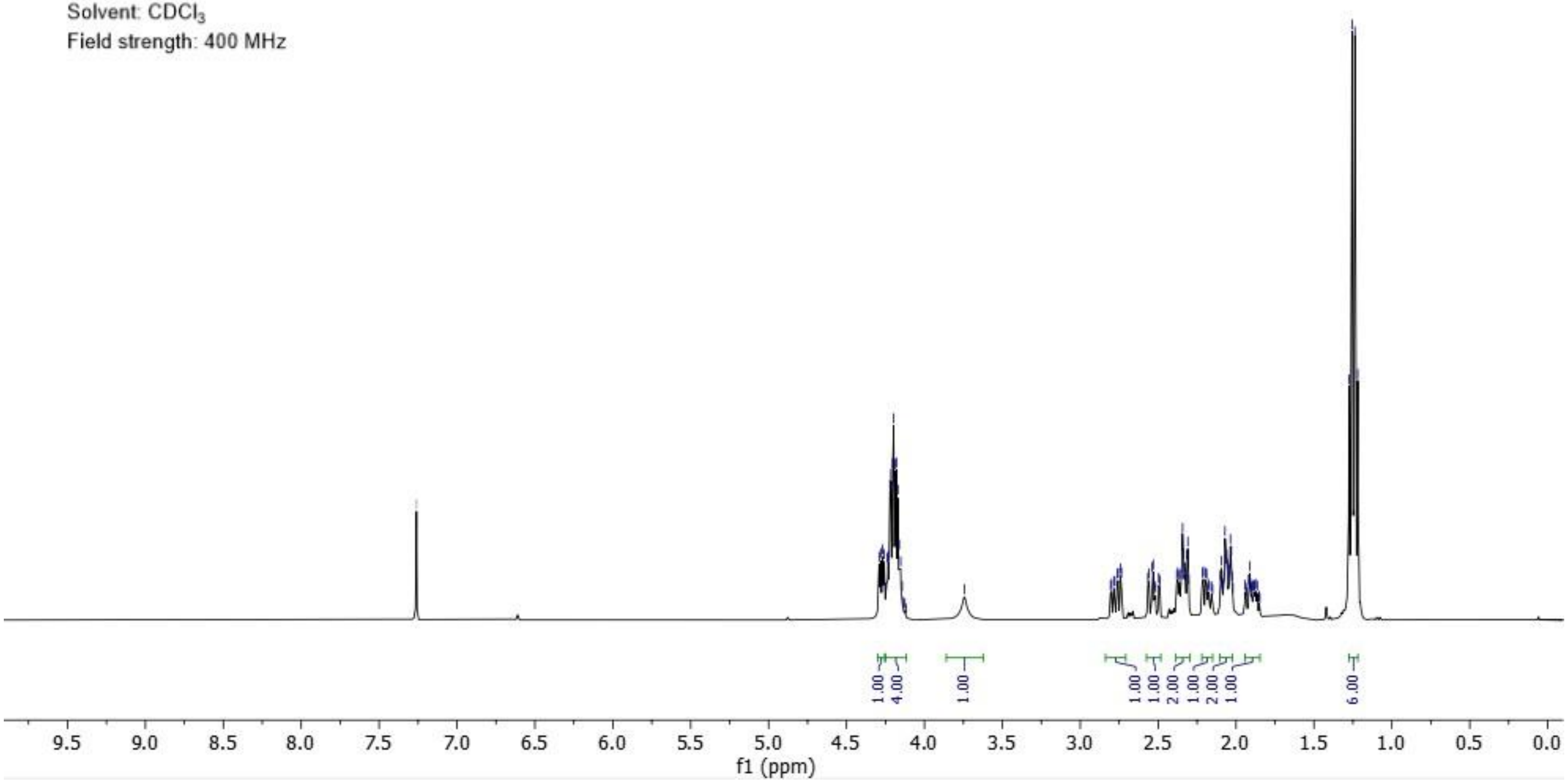

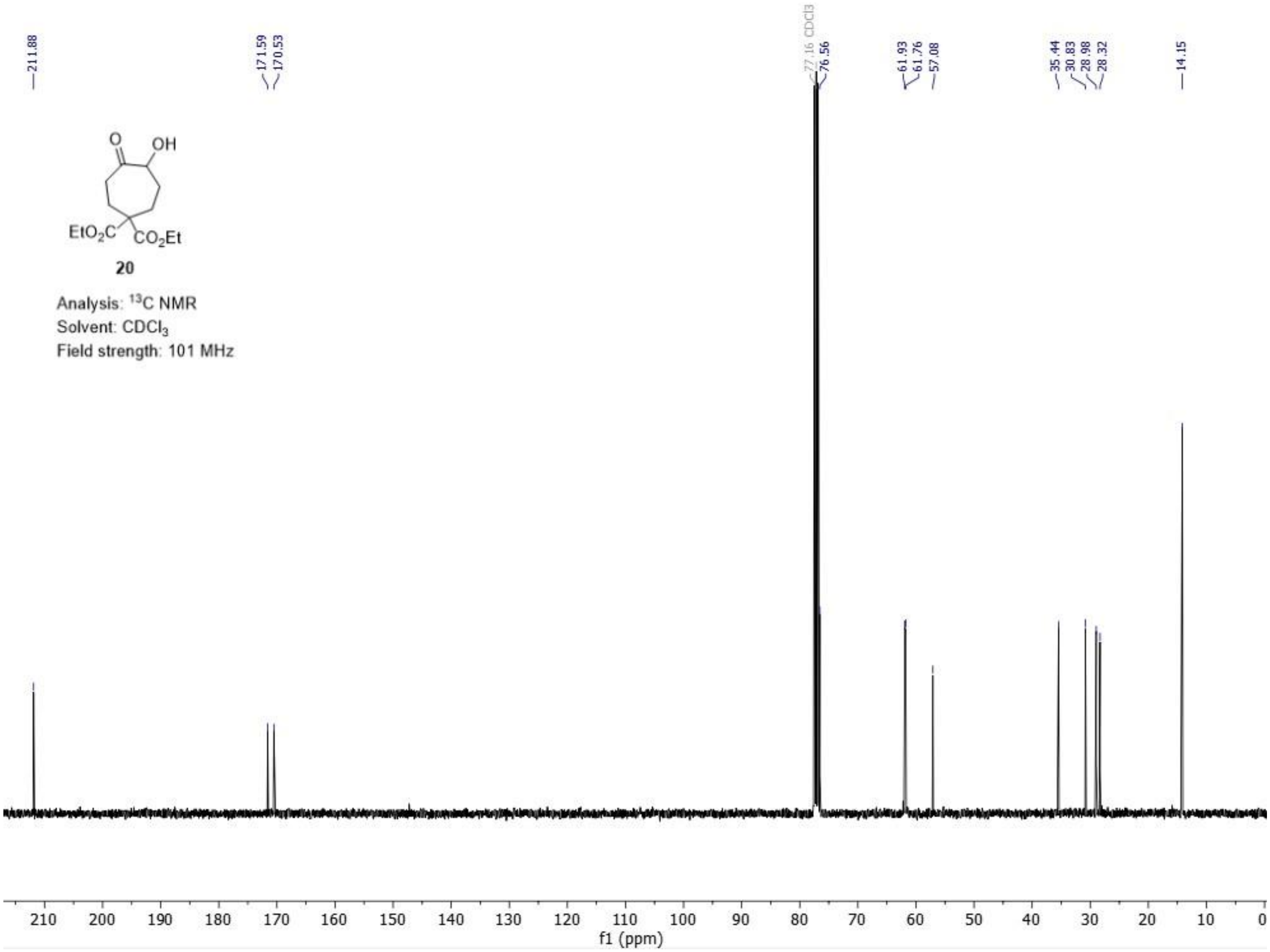

211.88
171.59
170.53
77.16 CDCl3
76.56
61.93
61.76
57.08
35.44
30.83
28.98
28.32
14.15
O
OH
EtO2C
CO2Et
20
Analysis: 13C NMR
Solvent: CDCl3
Field strength: 101 MHz
210 200 190 180 170 160 150 140 130 120 110 100 90 80 70 60 50 40 30 20 10 0
f1 (ppm)

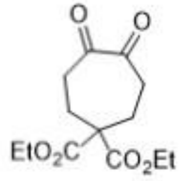

15

Analysis: ^{1}H NMR
Solvent: $CDCl_3$
Field strength: 400 MHz

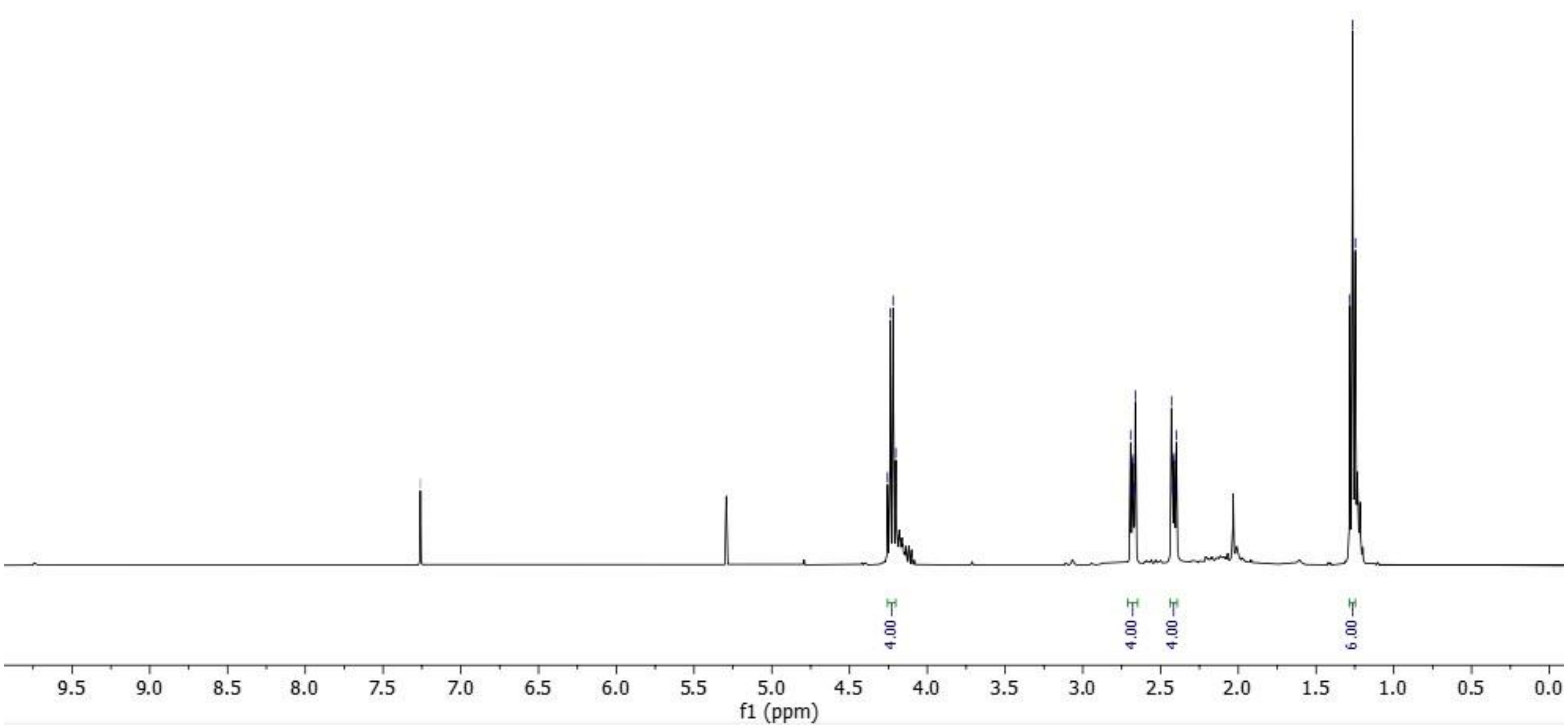

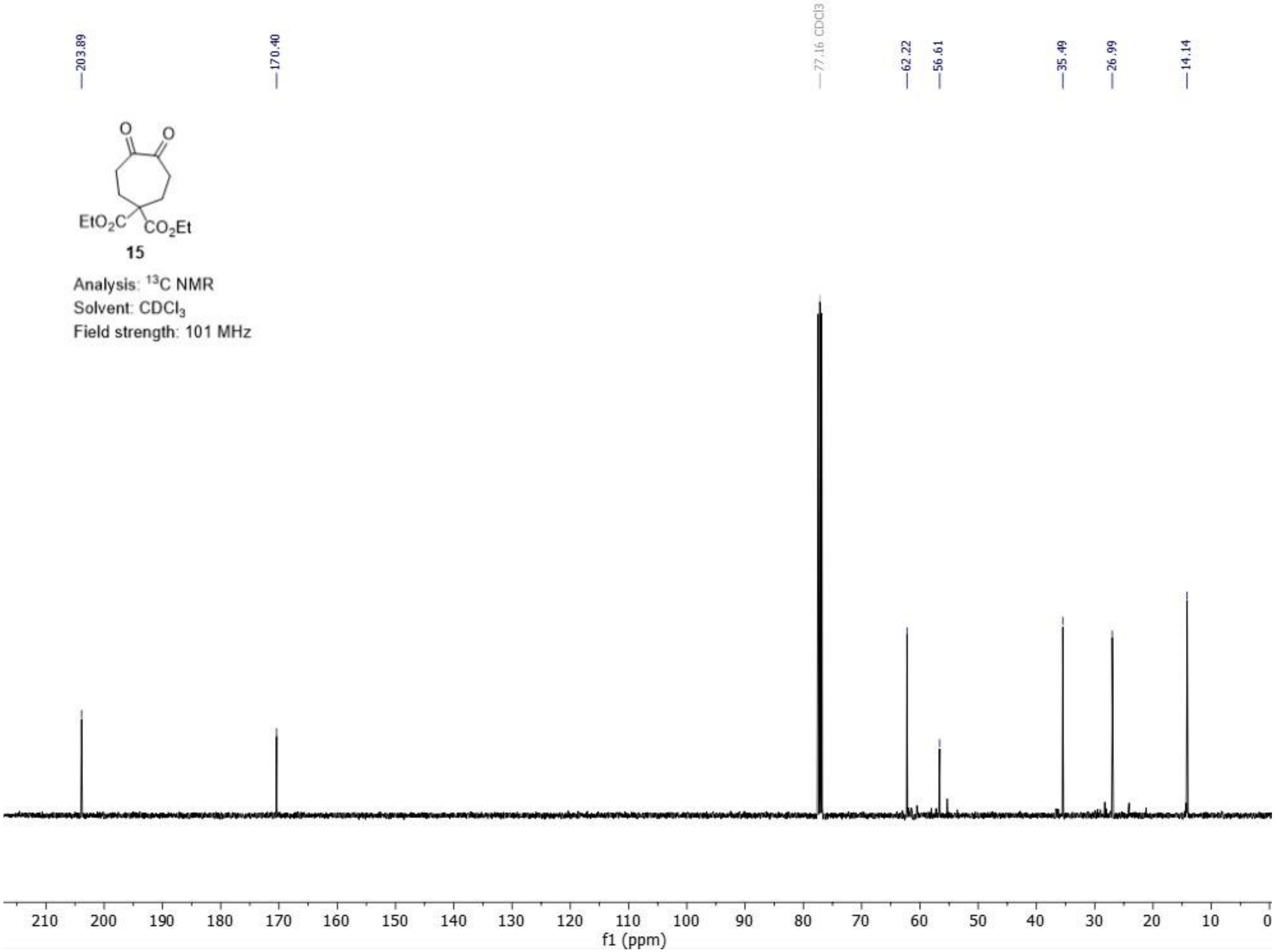
203.89
170.40
77.16 CDCl3
62.22
56.61
35.49
26.99
14.14
O
O
EtO2C
CO2Et
15
Analysis: 13C NMR
Solvent: CDCl3
Field strength: 101 MHz
210 200 190 180 170 160 150 140 130 120 110 100 90 80 70 60 50 40 30 20 10 0
f1 (ppm)

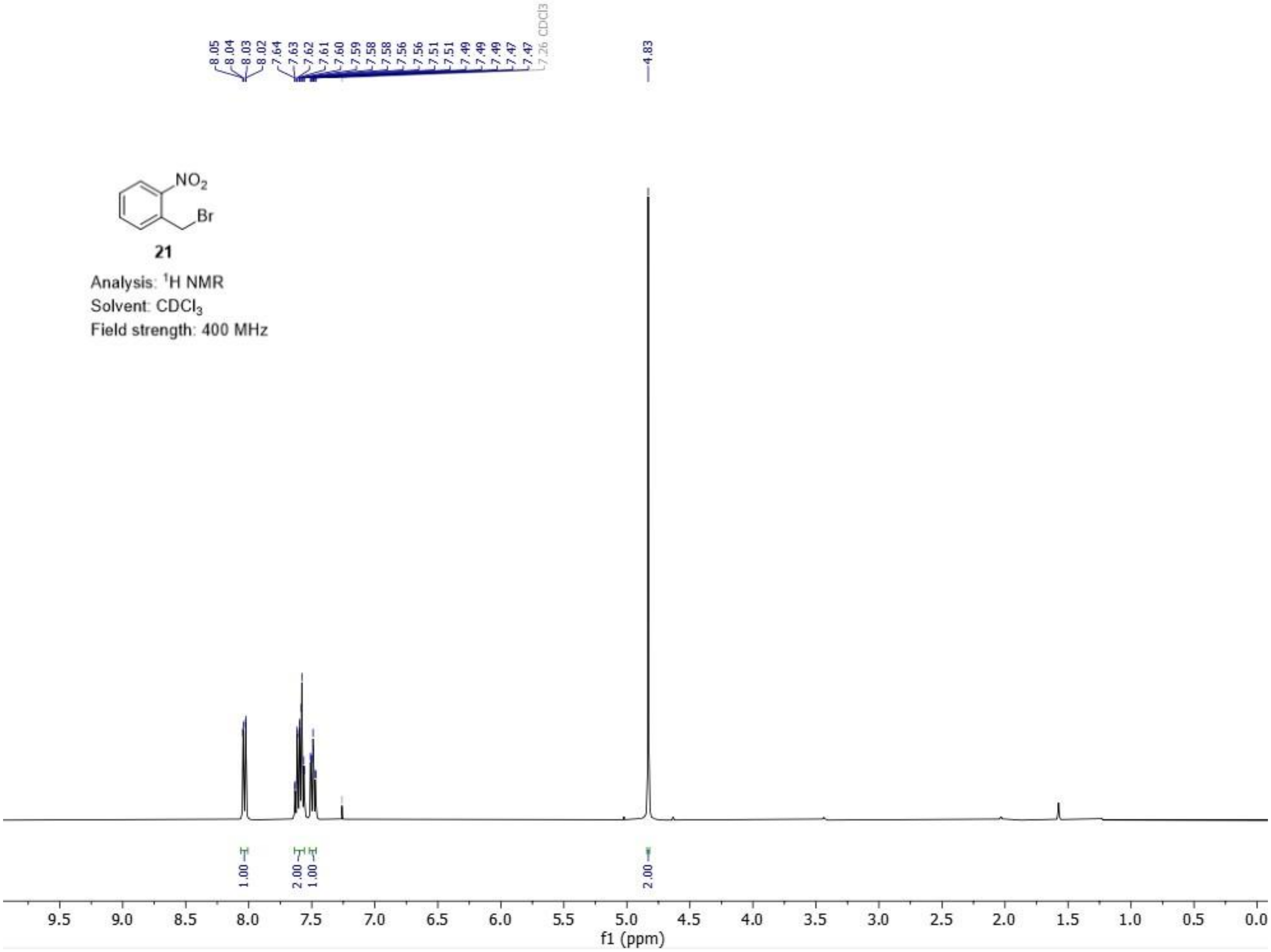

8.05
8.04
8.03
8.02
7.64
7.63
7.62
7.61
7.60
7.59
7.58
7.58
7.56
7.56
7.51
7.51
7.49
7.49
7.49
7.47
7.47
7.26 CDCl3
4.83
NO2
Br
21
Analysis: 1H NMR
Solvent: CDCl3
Field strength: 400 MHz
1.00
2.00
1.00
2.00
9.5
9.0
8.5
8.0
7.5
7.0
6.5
6.0
5.5
5.0
4.5
4.0
3.5
3.0
2.5
2.0
1.5
1.0
0.5
0.0
f1 (ppm)

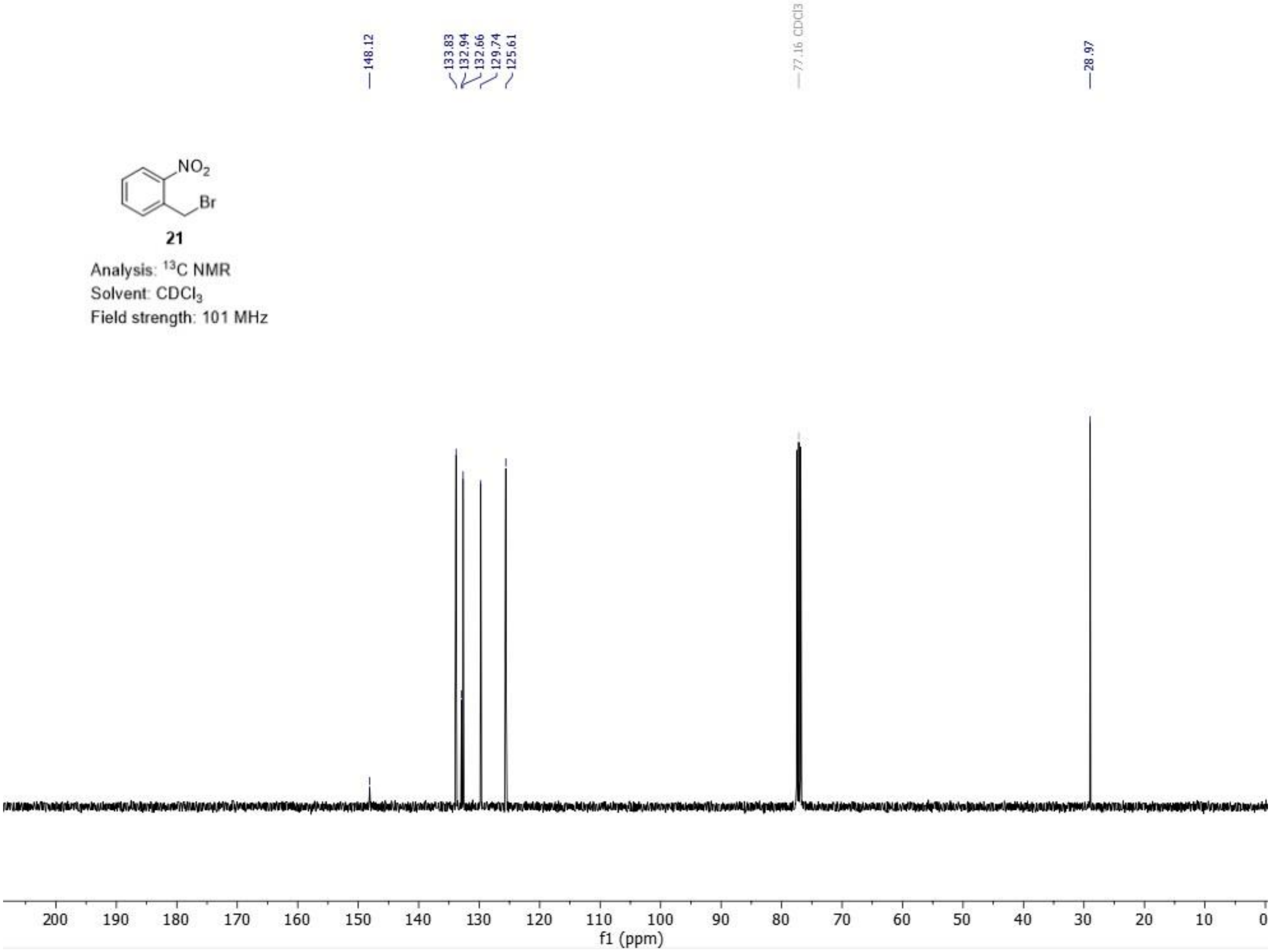

21

Analysis: ^{13}C NMR
Solvent: $CDCl_3$
Field strength: 101 MHz

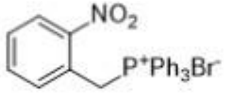

16

Analysis: ^{1}H NMR
Solvent: $CDCl_3$
Field strength: 400 MHz

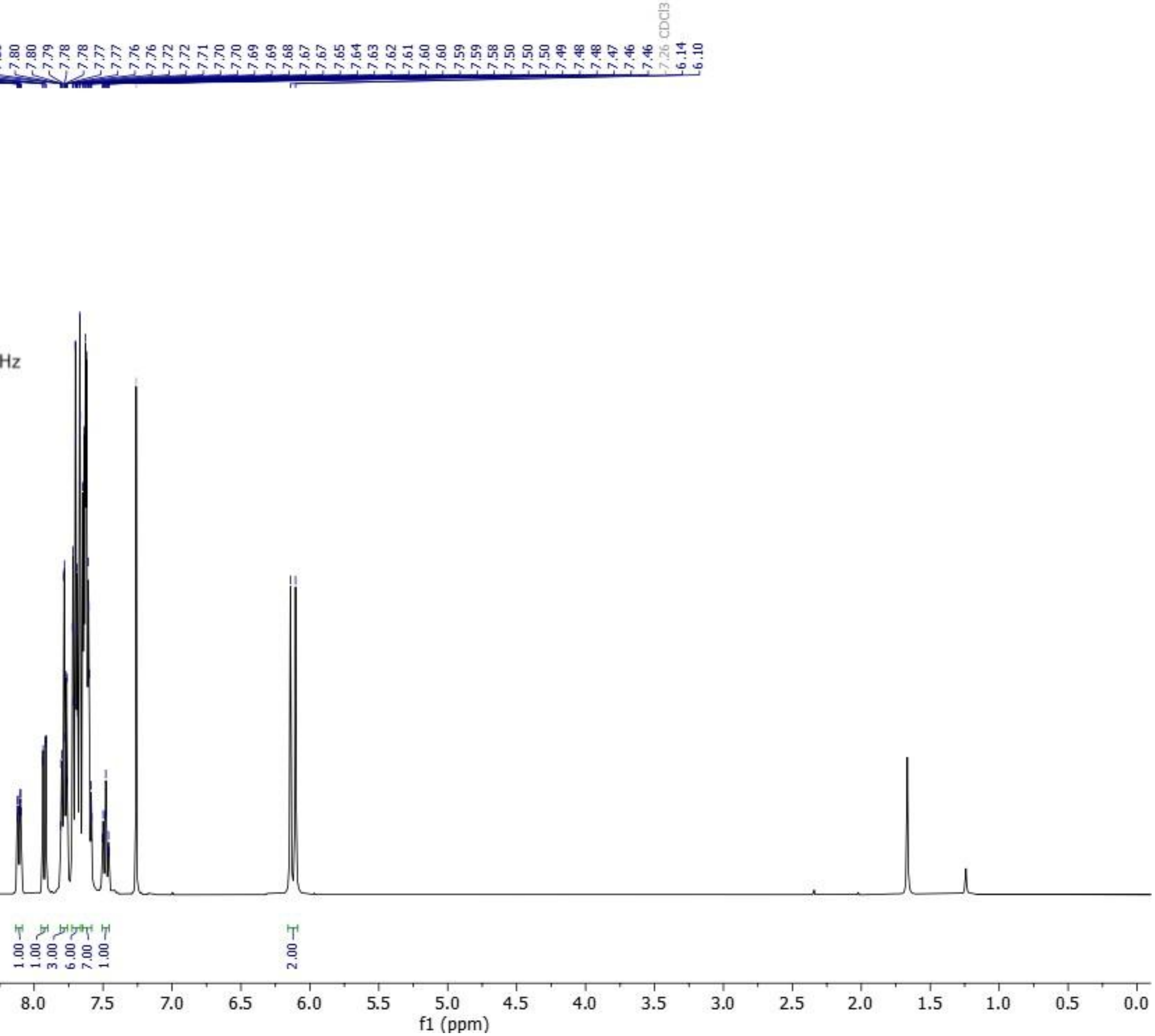

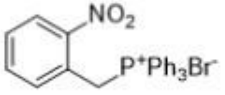

16

Analysis: ^{31}P NMR
Solvent: $CDCl_3$
Field strength: 162 MHz

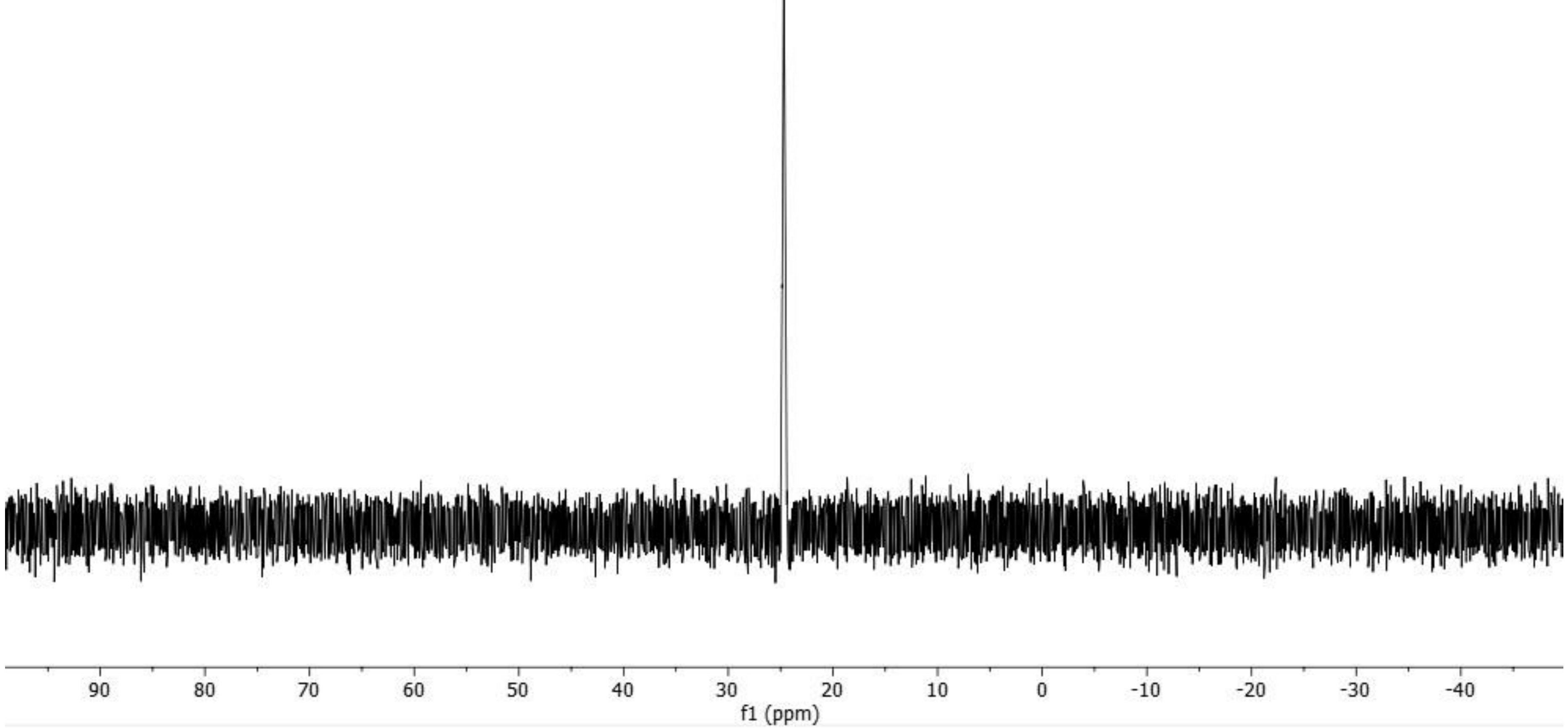

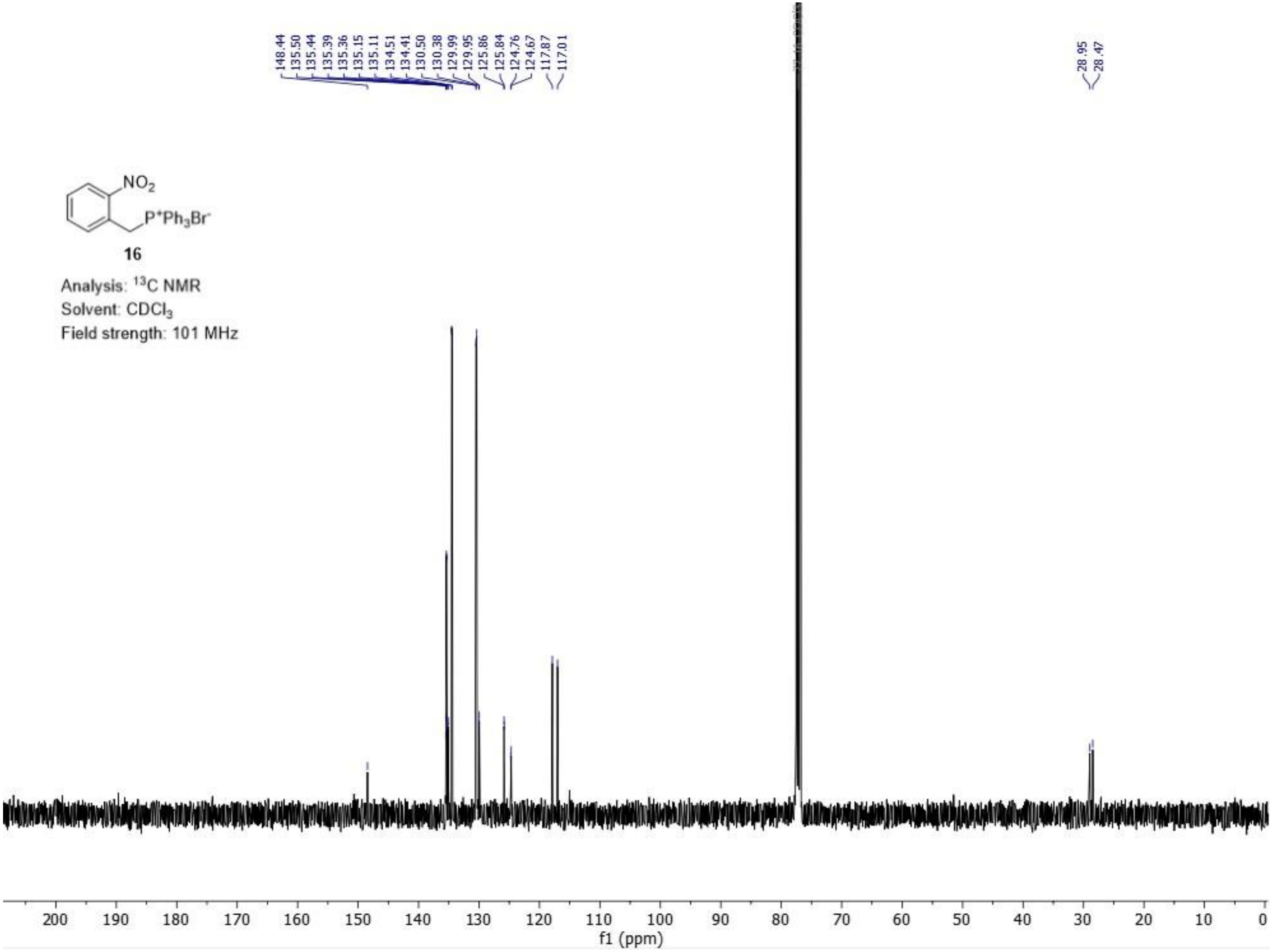
148.44
135.50
135.44
135.39
135.36
135.15
135.11
134.51
134.41
130.50
130.38
129.99
129.95
125.86
125.84
124.76
124.67
117.87
117.01
28.95
28.47
NO2
P+Ph3Br-
16
Analysis: 13C NMR
Solvent: CDCl3
Field strength: 101 MHz
200
190
180
170
160
150
140
130
120
110
100
90
80
70
60
50
40
30
20
10
0
f1 (ppm)

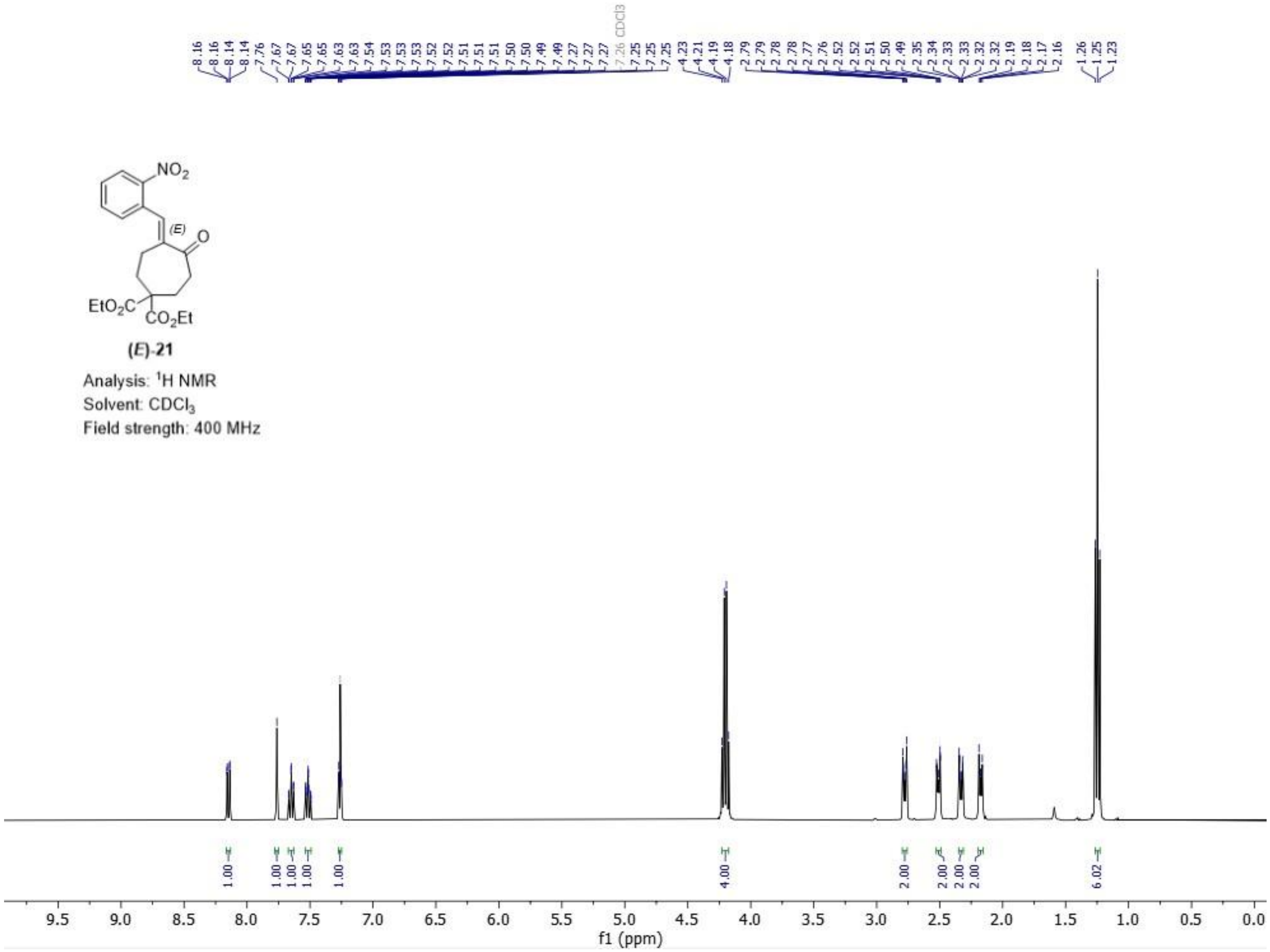

(*E*)-21

Analysis: ^{1}H NMR
Solvent: $CDCl_3$
Field strength: 400 MHz

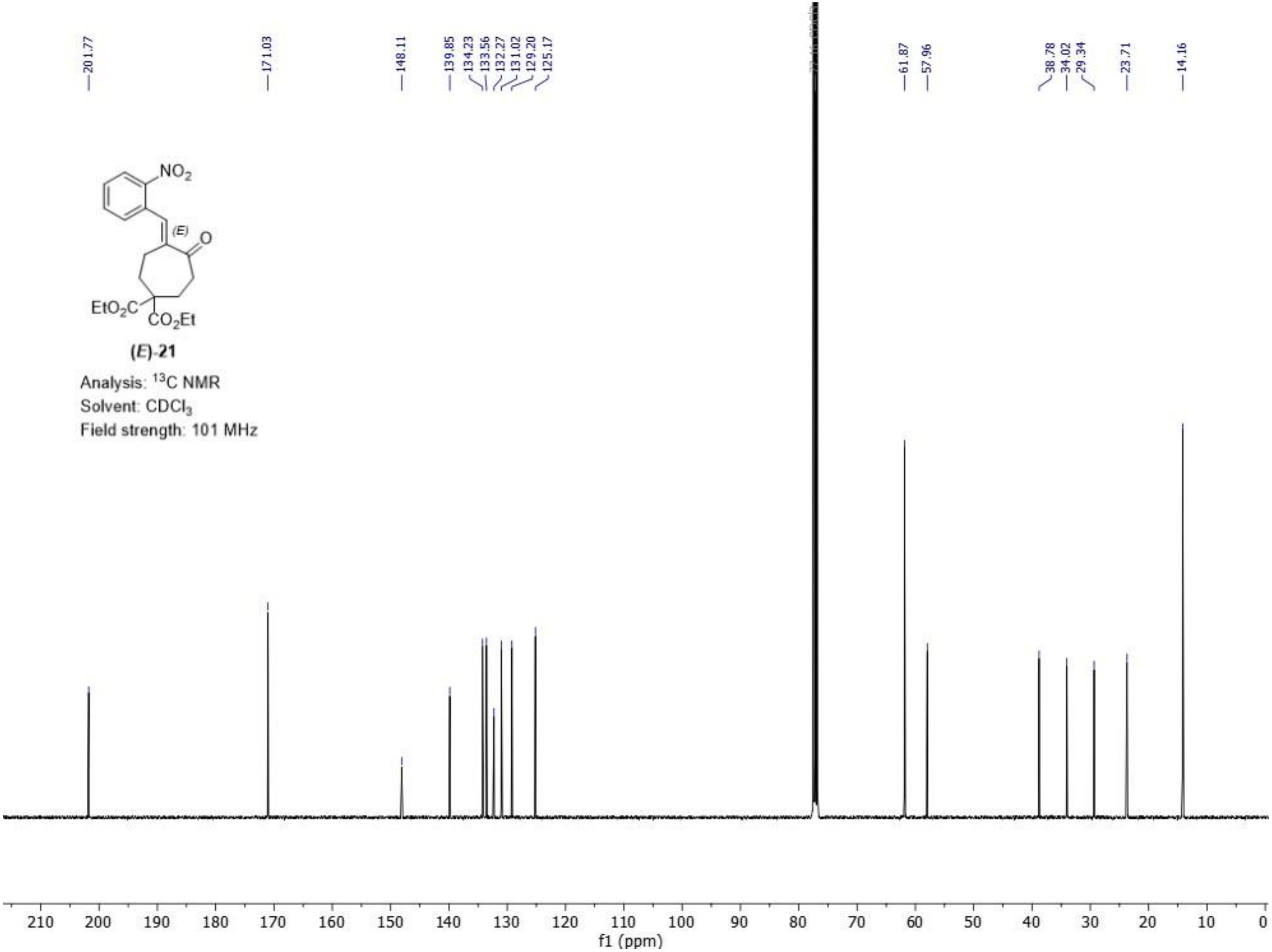

(*E*)-21

Analysis: ^{13}C NMR

Solvent: $CDCl_3$

Field strength: 101 MHz

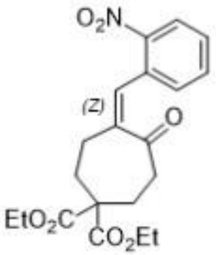

(*Z*)-21

Analysis: ^{1}H NMR
Solvent: $CDCl_3$
Field strength: 400 MHz

8.09 8.09 8.07 8.07 7.55 7.55 7.53 7.53 7.51 7.51 7.44 7.43 7.42 7.41 7.40 7.39 7.26 7.26 CDCl3 7.24 6.95
4.25 4.24 4.22 4.20 2.73 2.71 2.70 2.54 2.53 2.53 2.52 2.52 2.51 2.37 2.37 2.36 2.35 2.35 2.33 2.32 2.31 2.31 2.30 2.30 1.29 1.27 1.25

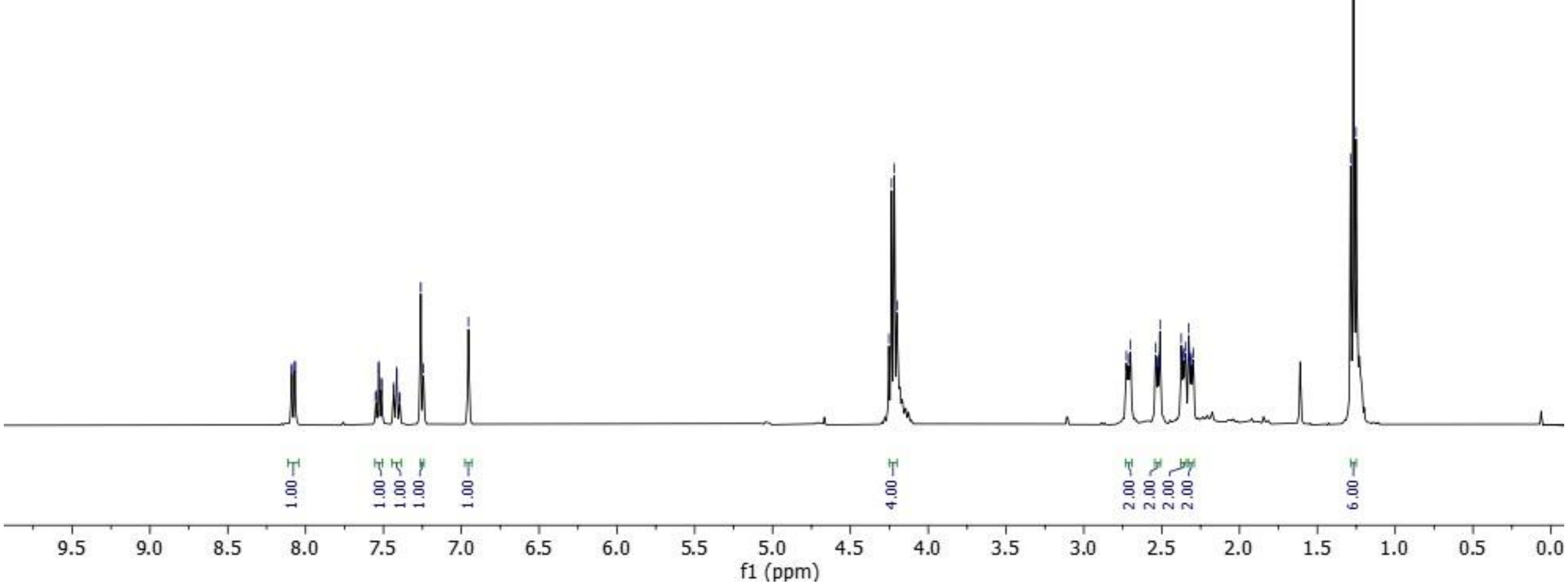

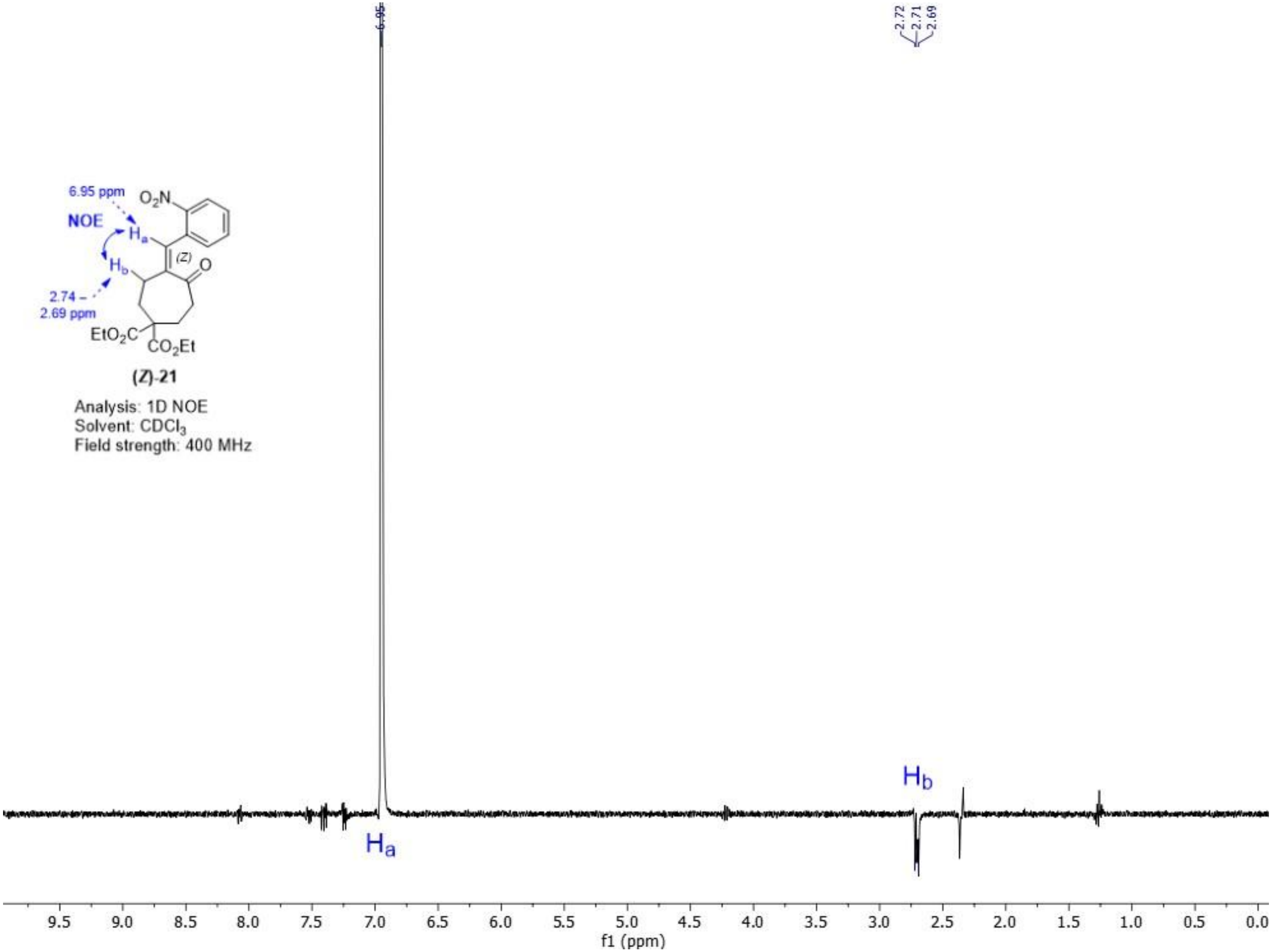
6.95 ppm
NOE
H_a
H_b
2.74 –
2.69 ppm
O_2N
(Z)
O
EtO_2C
CO_2Et
(Z)-21
Analysis: 1D NOE
Solvent: $CDCl_3$
Field strength: 400 MHz
6.95
2.72
2.71
2.69
H_a
H_b
9.5 9.0 8.5 8.0 7.5 7.0 6.5 6.0 5.5 5.0 4.5 4.0 3.5 3.0 2.5 2.0 1.5 1.0 0.5 0.0
f1 (ppm)

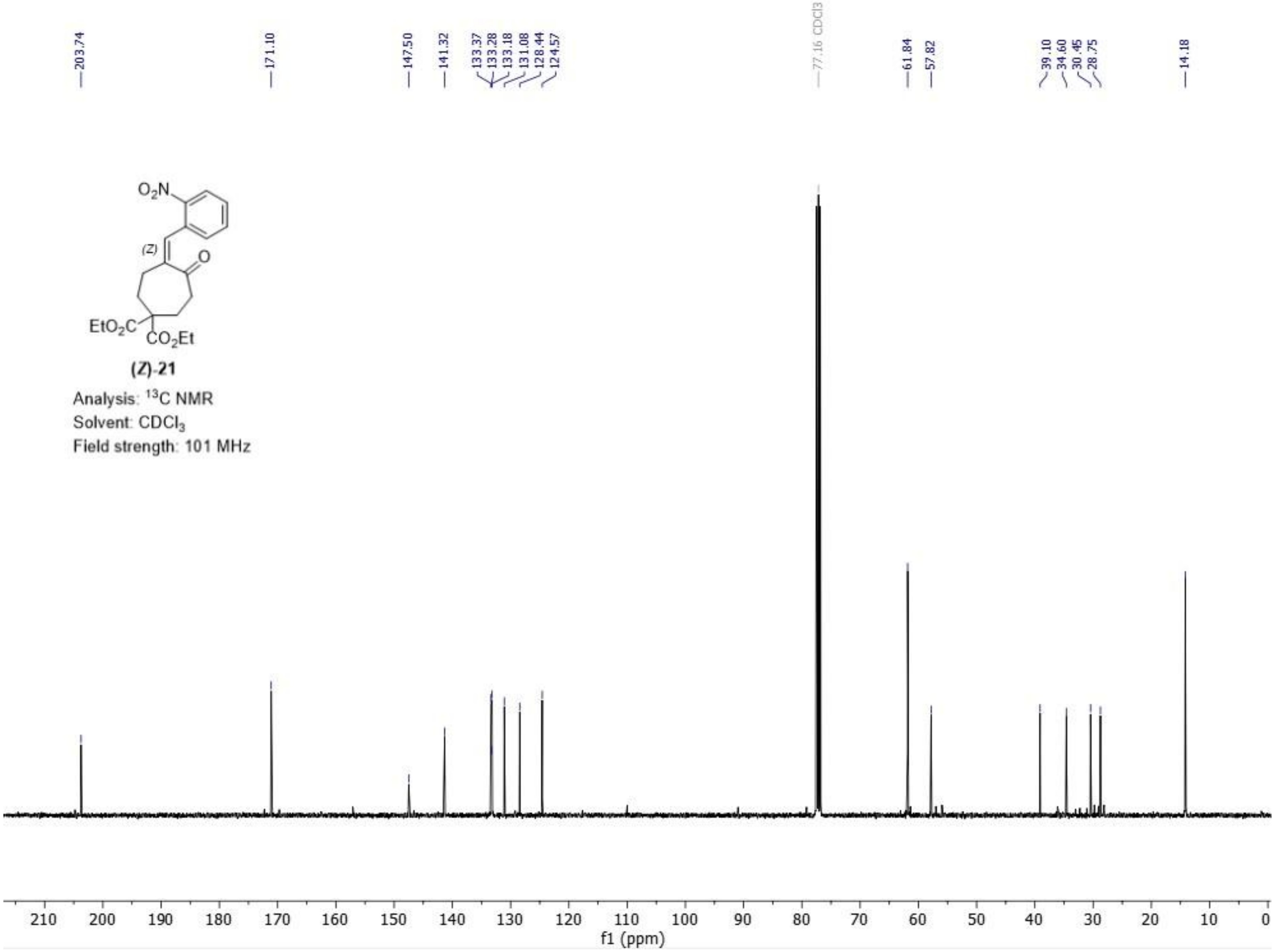
203.74
171.10
147.50
141.32
133.37
133.28
133.18
131.08
128.44
124.57
77.16 CDCl3
61.84
57.82
39.10
34.60
30.45
28.75
14.18
O_2N
(Z)
O
EtO_2C
CO_2Et
(Z)-21
Analysis: ^{13}C NMR
Solvent: $CDCl_3$
Field strength: 101 MHz
210 200 190 180 170 160 150 140 130 120 110 100 90 80 70 60 50 40 30 20 10 0
f1 (ppm)

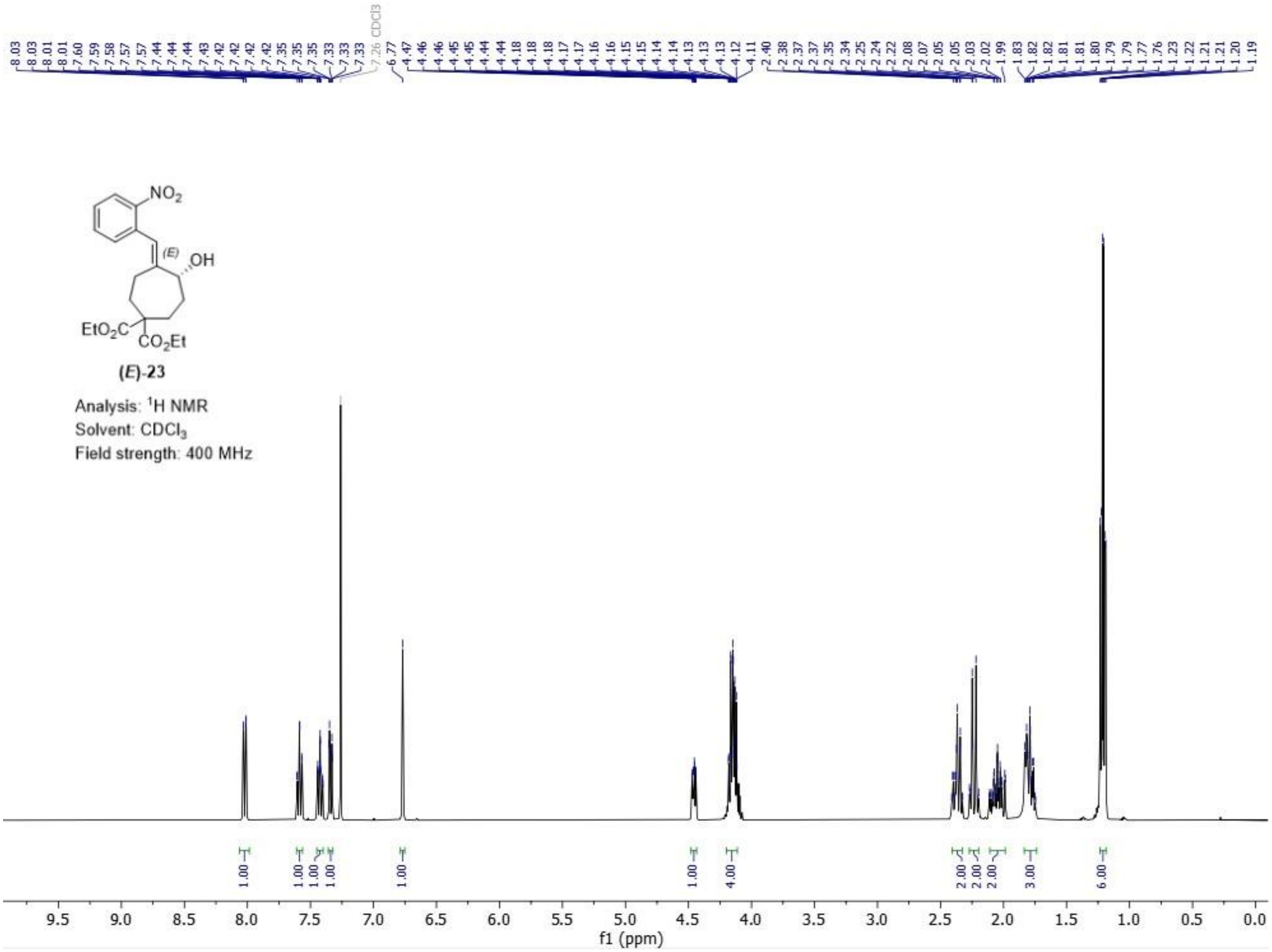
8.03 8.03 8.01 8.01 7.60 7.59 7.58 7.57 7.57 7.44 7.44 7.44 7.43 7.42 7.42 7.42 7.42 7.35 7.35 7.35 7.33 7.33 7.33 7.26 CDCl3 6.77 4.47 4.46 4.46 4.45 4.45 4.44 4.44 4.18 4.18 4.18 4.17 4.17 4.16 4.16 4.15 4.15 4.14 4.14 4.13 4.13 4.13 4.12 4.11 2.40 2.38 2.37 2.37 2.35 2.34 2.25 2.24 2.22 2.08 2.07 2.05 2.05 2.03 2.02 1.99 1.83 1.82 1.82 1.81 1.80 1.79 1.79 1.77 1.76 1.23 1.22 1.21 1.21 1.20 1.19
NO2
(E)
OH
EtO2C
CO2Et
(E)-23
Analysis: 1H NMR
Solvent: CDCl3
Field strength: 400 MHz
1.00 1.00 1.00 1.00 1.00 1.00 4.00 2.00 2.00 2.00 3.00 6.00
9.5 9.0 8.5 8.0 7.5 7.0 6.5 6.0 5.5 5.0 4.5 4.0 3.5 3.0 2.5 2.0 1.5 1.0 0.5 0.0
f1 (ppm)

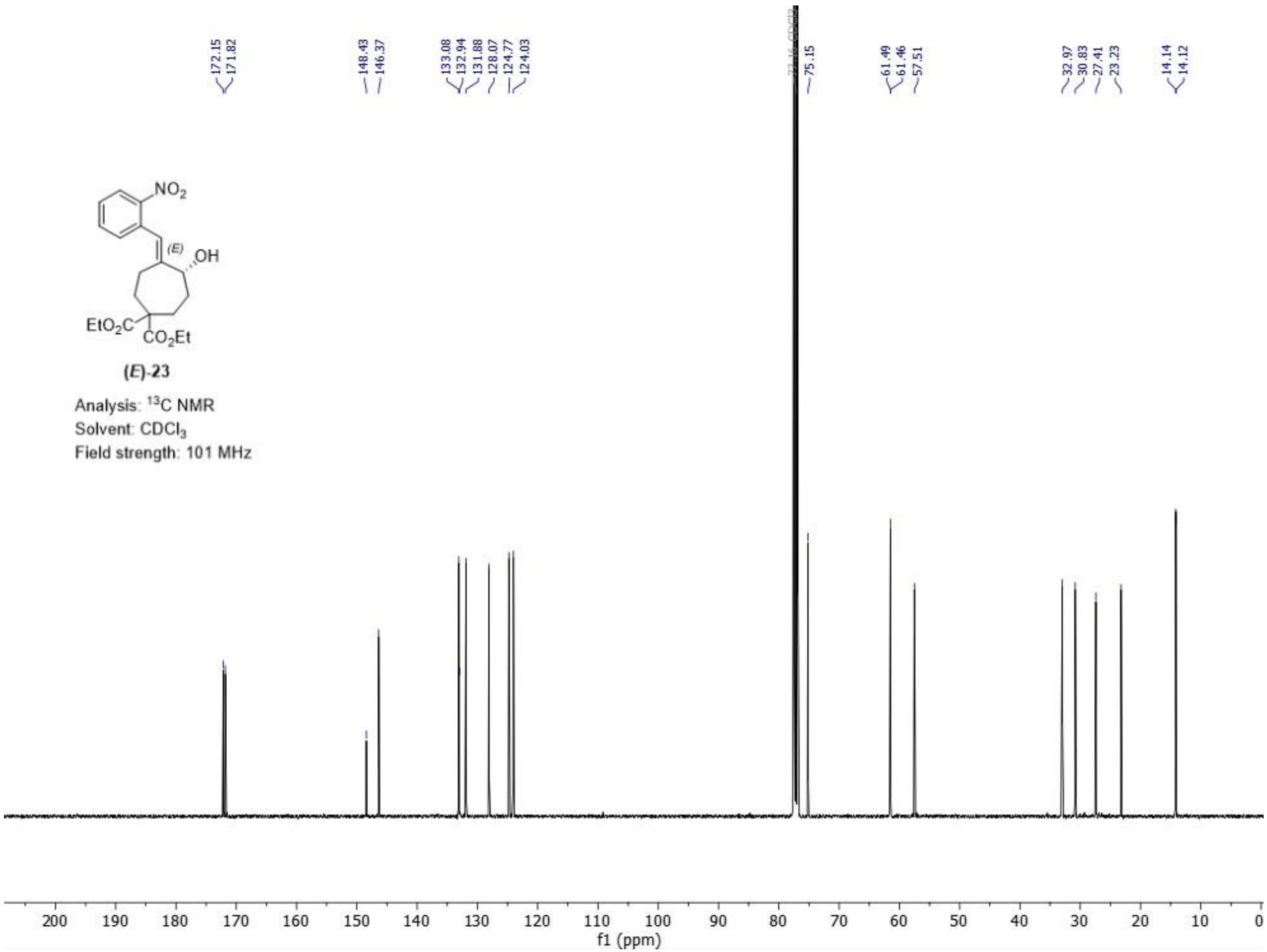

172.15
171.82
148.43
146.37
133.08
132.94
131.88
128.07
124.77
124.03
75.15
61.49
61.46
57.51
32.97
30.83
27.41
23.23
14.14
14.12
NO_2
(E)
OH
EtO_2C
CO_2Et
(E)-23
Analysis: ^{13}C NMR
Solvent: $CDCl_3$
Field strength: 101 MHz
200 190 180 170 160 150 140 130 120 110 100 90 80 70 60 50 40 30 20 10 0
f1 (ppm)

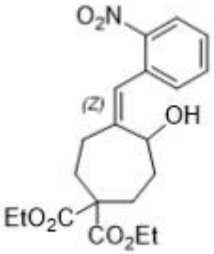

(Z)-23

Analysis: ^{1}H NMR

Solvent: $CDCl_3$

Field strength: 600 MHz

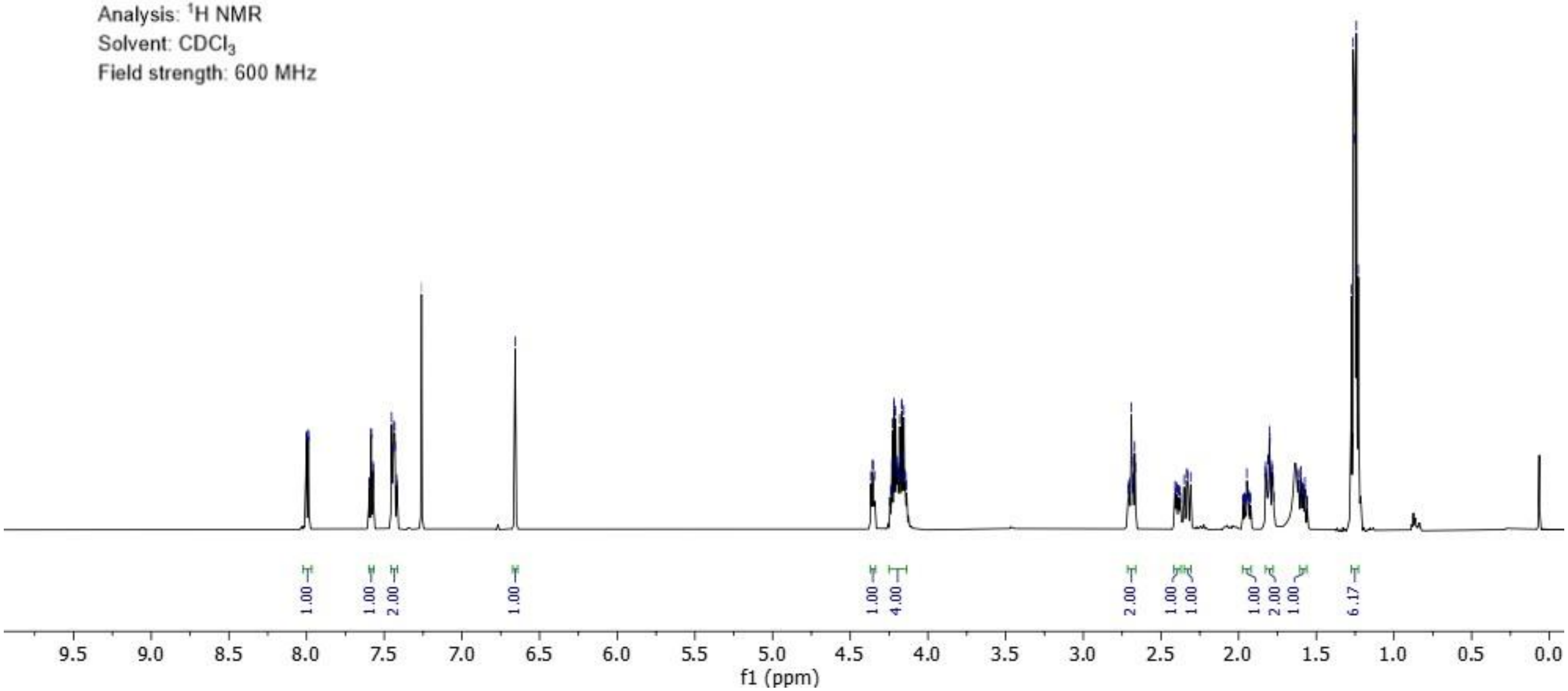

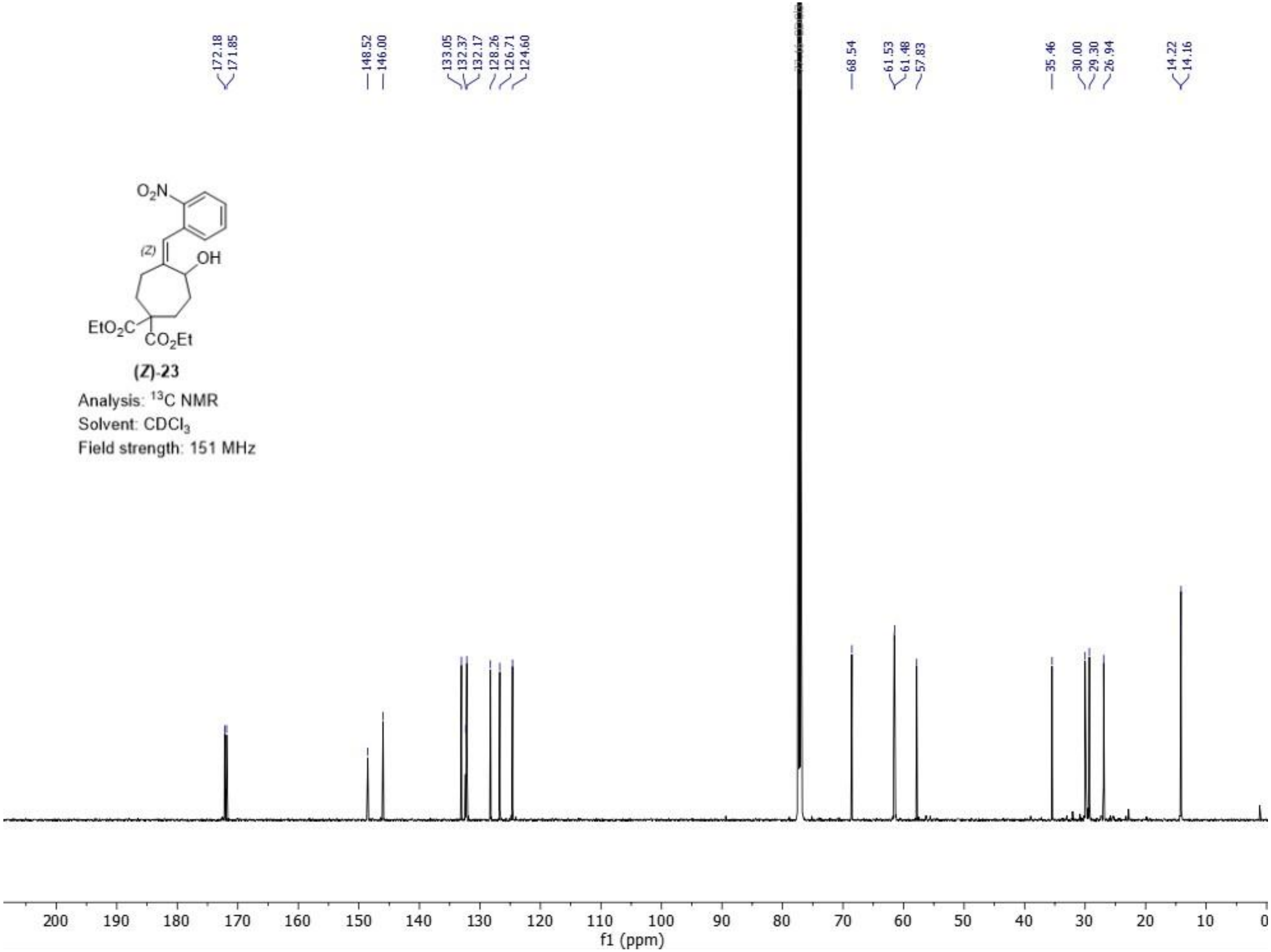
172.18
171.85
148.52
146.00
133.05
132.37
132.17
128.26
126.71
124.60
68.54
61.53
61.48
57.83
35.46
30.00
29.30
26.94
14.22
14.16
O2N
(Z)
OH
EtO2C
CO2Et
(Z)-23
Analysis: 13C NMR
Solvent: CDCl3
Field strength: 151 MHz
200 190 180 170 160 150 140 130 120 110 100 90 80 70 60 50 40 30 20 10 0
f1 (ppm)

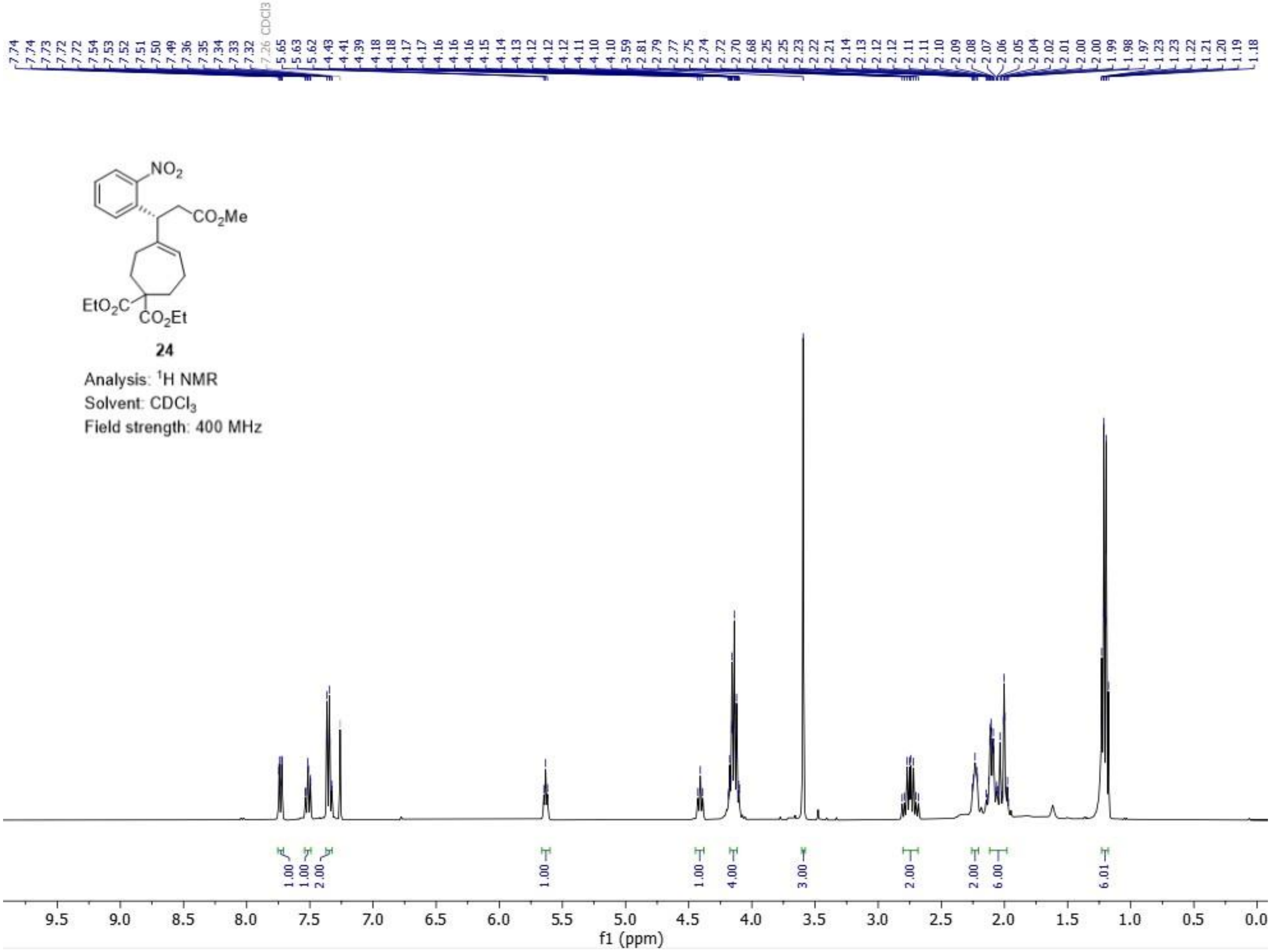
7.74 7.74 7.73 7.72 7.72 7.54 7.53 7.52 7.51 7.50 7.49 7.36 7.35 7.34 7.33 7.32 7.26 CDCl3 5.65 5.63 5.62 4.43 4.41 4.39 4.18 4.18 4.17 4.17 4.16 4.16 4.16 4.15 4.14 4.13 4.12 4.12 4.12 4.11 4.10 4.10 3.59 2.81 2.79 2.77 2.75 2.74 2.72 2.70 2.68 2.25 2.25 2.23 2.22 2.21 2.14 2.13 2.12 2.12 2.11 2.11 2.10 2.09 2.08 2.07 2.06 2.05 2.04 2.02 2.01 2.00 2.00 1.99 1.98 1.97 1.23 1.23 1.22 1.21 1.20 1.19 1.18
NO2
CO2Me
EtO2C
CO2Et
24
Analysis: 1H NMR
Solvent: CDCl3
Field strength: 400 MHz
1.00 1.00 2.00 1.00 1.00 4.00 3.00 2.00 2.00 6.00 6.01
9.5 9.0 8.5 8.0 7.5 7.0 6.5 6.0 5.5 5.0 4.5 4.0 3.5 3.0 2.5 2.0 1.5 1.0 0.5 0.0
f1 (ppm)

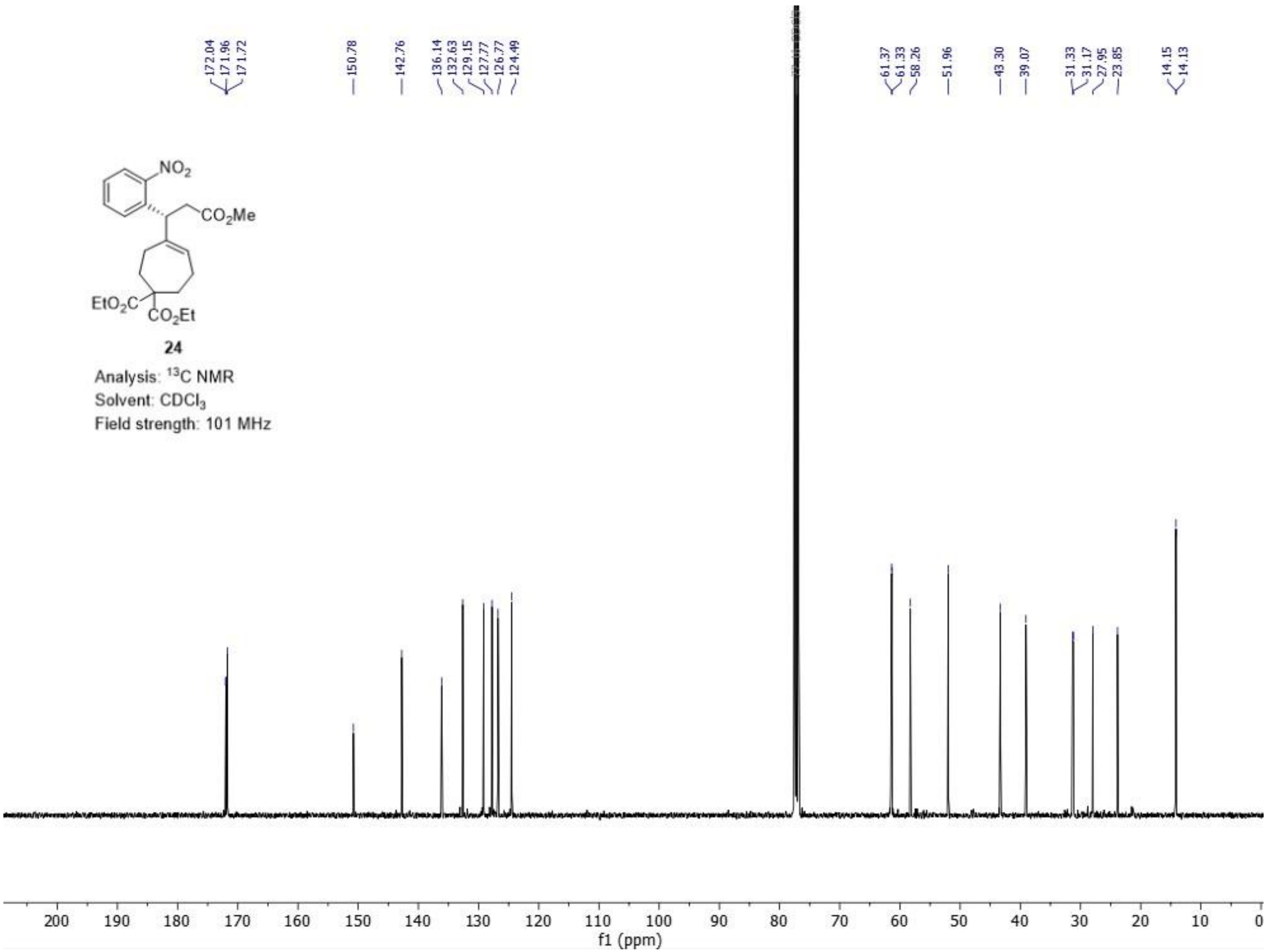

172.04
171.96
171.72
150.78
142.76
136.14
132.63
129.15
127.77
126.77
124.49
61.37
61.33
58.26
51.96
43.30
39.07
31.33
31.17
27.95
23.85
14.15
14.13
NO2
CO2Me
EtO2C
CO2Et
24
Analysis: 13C NMR
Solvent: CDCl3
Field strength: 101 MHz
200 190 180 170 160 150 140 130 120 110 100 90 80 70 60 50 40 30 20 10 0
f1 (ppm)

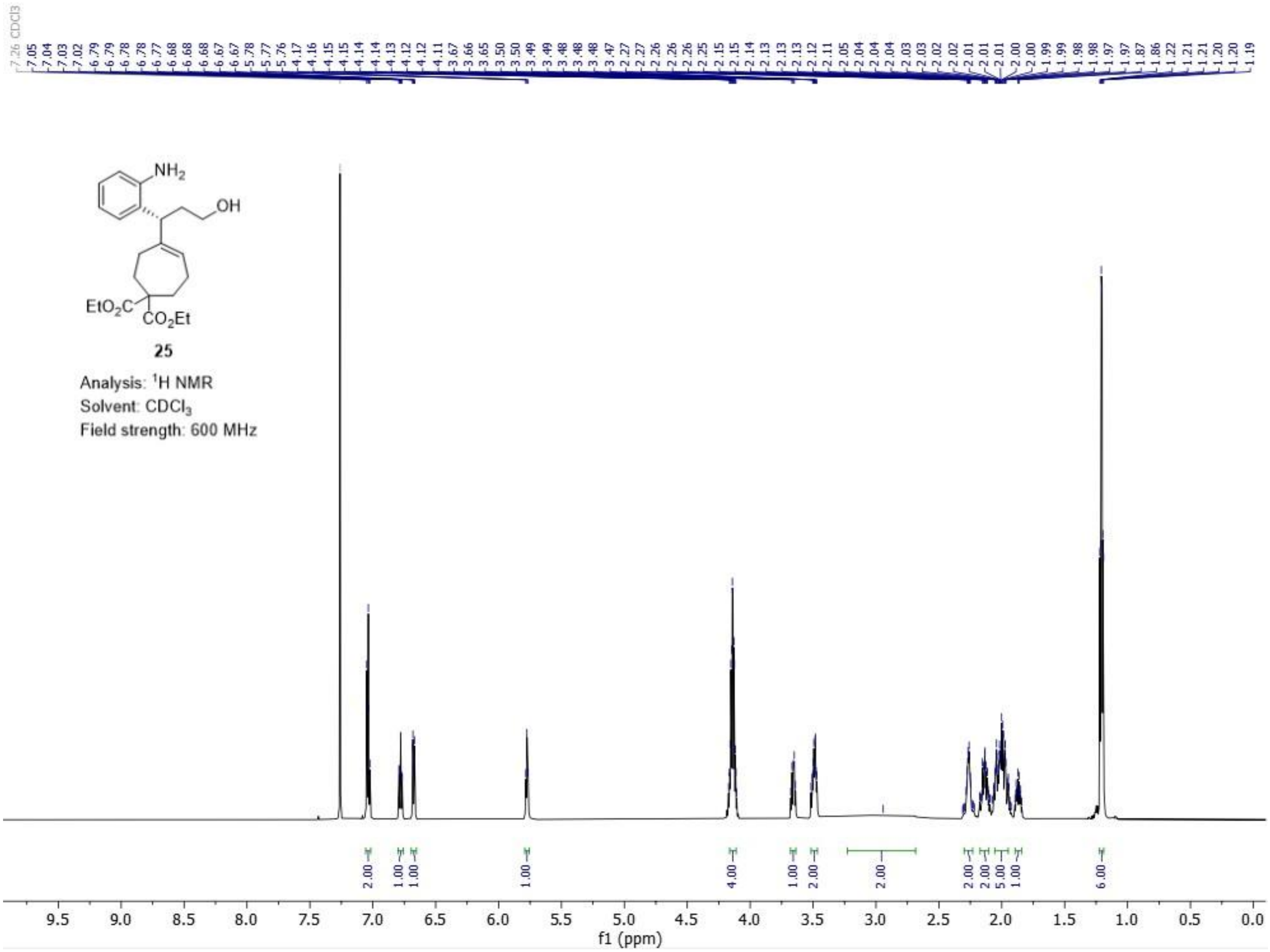
7.26 CDCl3
7.05 7.04 7.03 7.02 6.79 6.79 6.78 6.78 6.77 6.68 6.68 6.68 6.67 6.67 5.78 5.77 5.76 4.17 4.16 4.15 4.15 4.14 4.14 4.13 4.12 4.12 4.11 3.67 3.66 3.65 3.50 3.50 3.49 3.49 3.48 3.48 3.48 3.47 2.27 2.27 2.26 2.26 2.25 2.15 2.15 2.14 2.13 2.13 2.13 2.12 2.11 2.05 2.04 2.04 2.04 2.03 2.03 2.02 2.02 2.01 2.01 2.01 2.00 2.00 1.99 1.99 1.98 1.98 1.97 1.97 1.87 1.86 1.22 1.21 1.21 1.20 1.20 1.19
NH2
OH
EtO2C
CO2Et
25
Analysis: 1H NMR
Solvent: CDCl3
Field strength: 600 MHz
2.00 1.00 1.00 1.00 4.00 1.00 2.00 2.00 2.00 2.00 5.00 1.00 6.00
9.5 9.0 8.5 8.0 7.5 7.0 6.5 6.0 5.5 5.0 4.5 4.0 3.5 3.0 2.5 2.0 1.5 1.0 0.5 0.0
f1 (ppm)

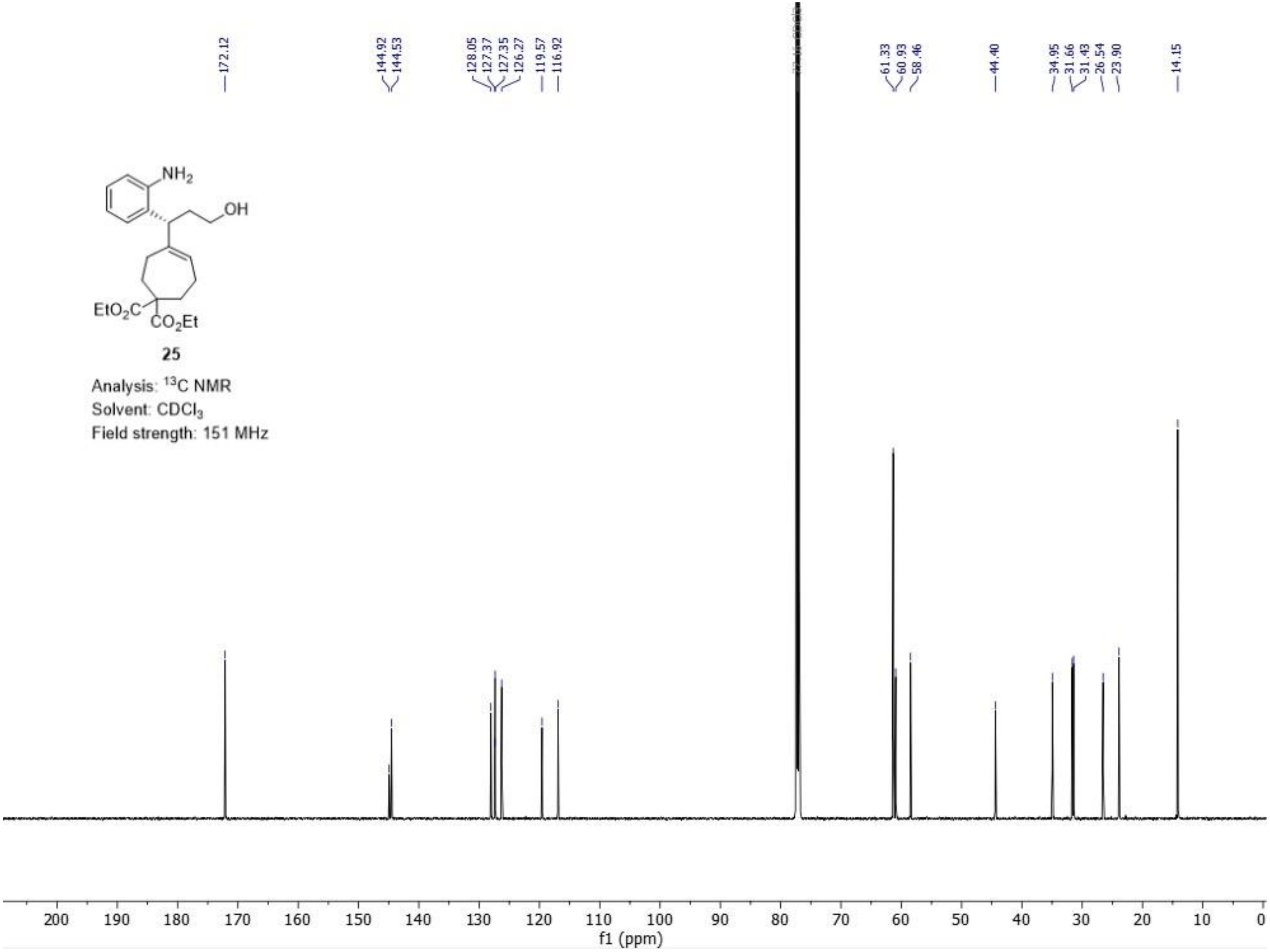

172.12
144.92
144.53
128.05
127.37
127.35
126.27
119.57
116.92
61.33
60.93
58.46
44.40
34.95
31.66
31.43
26.54
23.90
14.15
NH2
OH
EtO2C
CO2Et
25
Analysis: 13C NMR
Solvent: CDCl3
Field strength: 151 MHz
200 190 180 170 160 150 140 130 120 110 100 90 80 70 60 50 40 30 20 10 0
f1 (ppm)

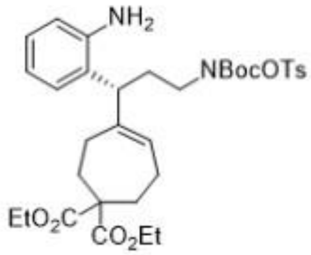

26

Analysis: ^1H NMR
Solvent: $CDCl_3$
Field strength: 400 MHz

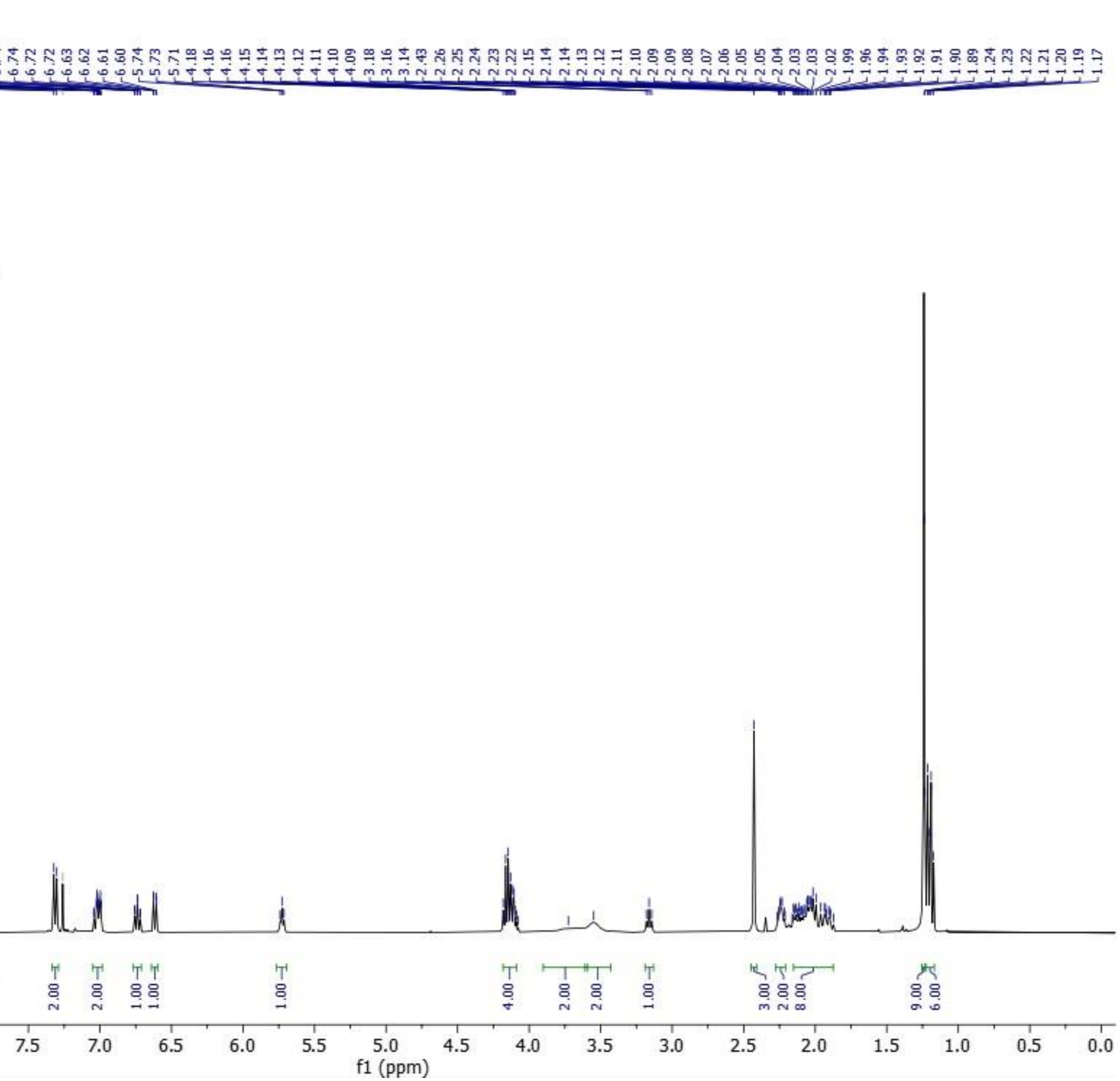

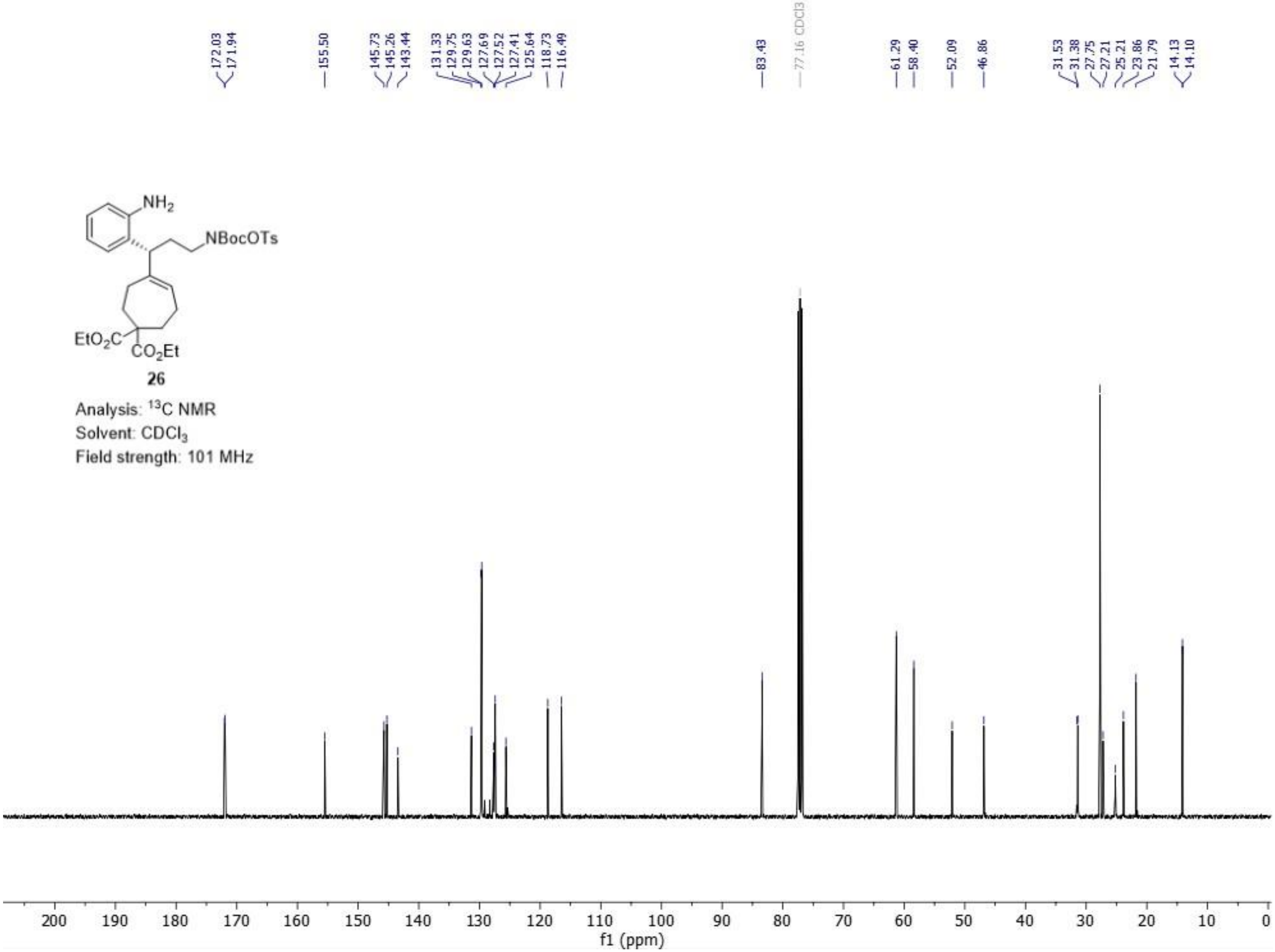

172.03
171.94
155.50
145.73
145.26
143.44
131.33
129.75
129.63
127.69
127.52
127.41
125.64
118.73
116.49
83.43
77.16 CDCl3
61.29
58.40
52.09
46.86
31.53
31.38
27.75
27.21
25.21
23.86
21.79
14.13
14.10
NH2
NBocOTs
EtO2C
CO2Et
26
Analysis: 13C NMR
Solvent: CDCl3
Field strength: 101 MHz
200 190 180 170 160 150 140 130 120 110 100 90 80 70 60 50 40 30 20 10 0
f1 (ppm)

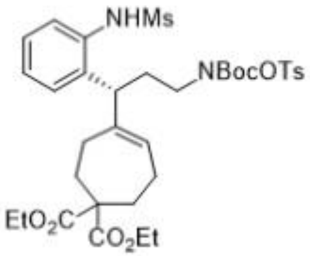

14

Analysis: ^{1}H NMR
Solvent: $CDCl_3$
Field strength: 400 MHz

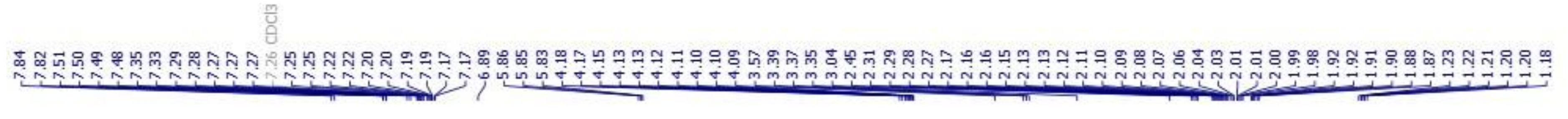
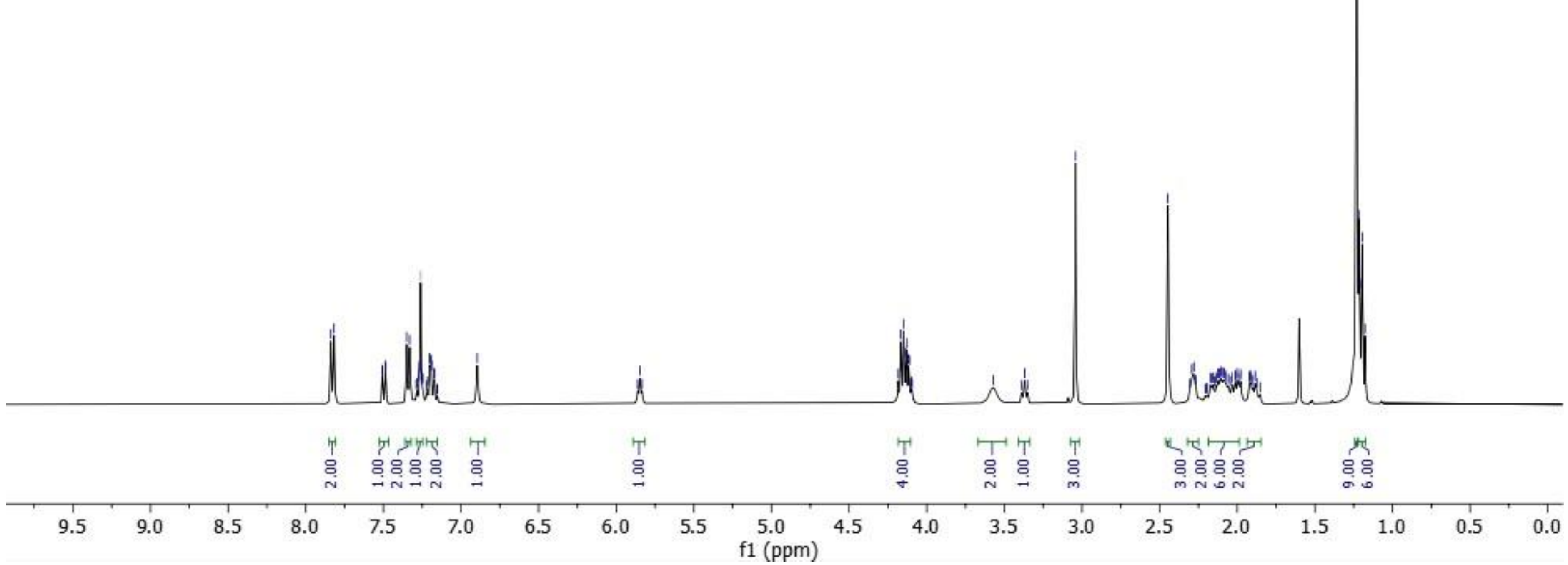

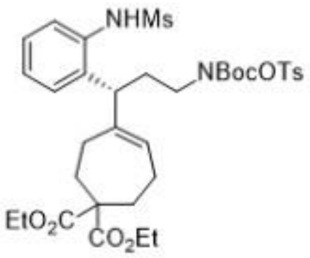

14

Analysis: ^{13}C NMR

Solvent: $CDCl_3$

Field strength: 101 MHz

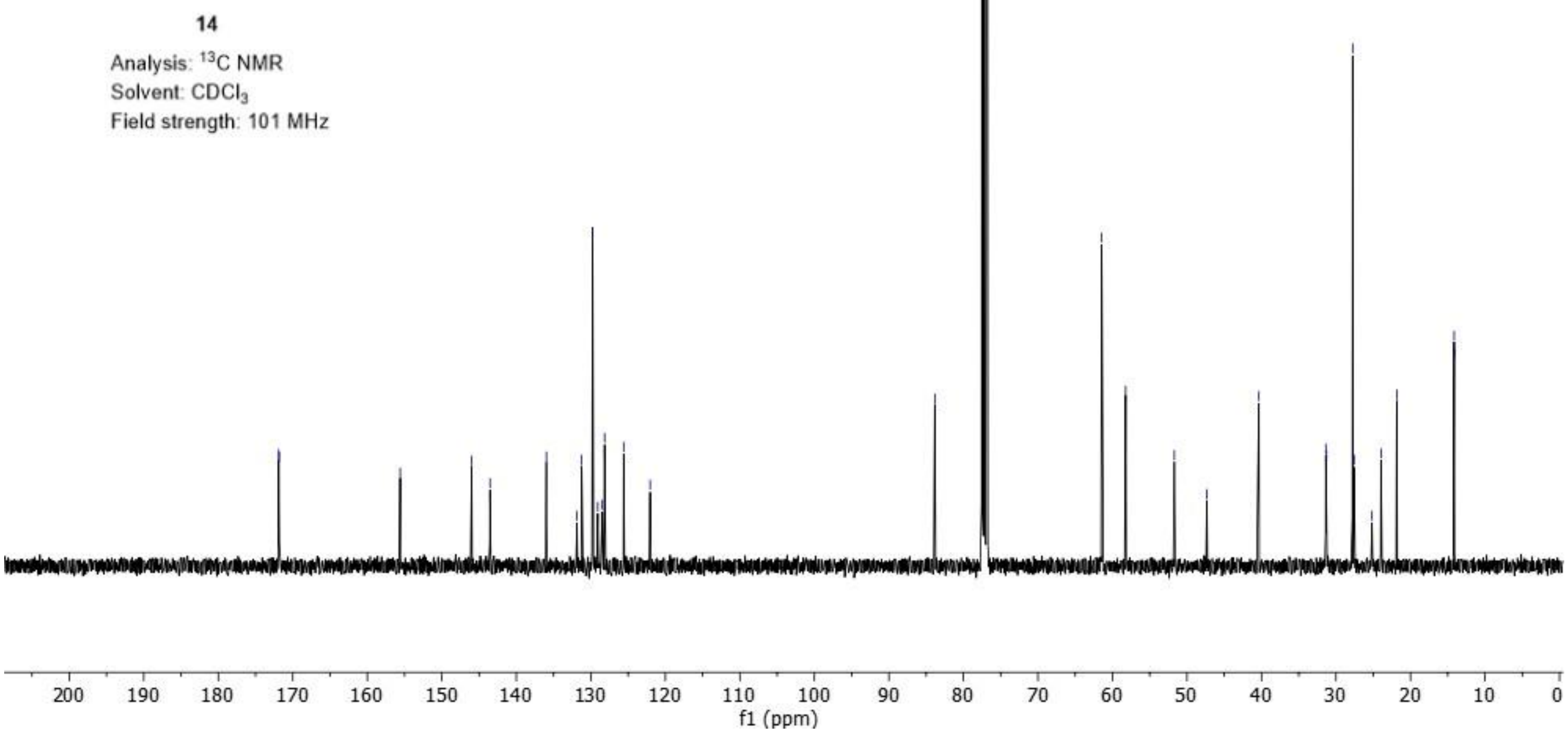

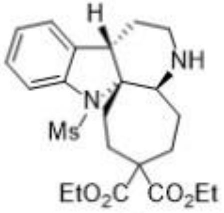

13

Analysis: ^1H NMR

Solvent: $CDCl_3$

Field strength: 400 MHz

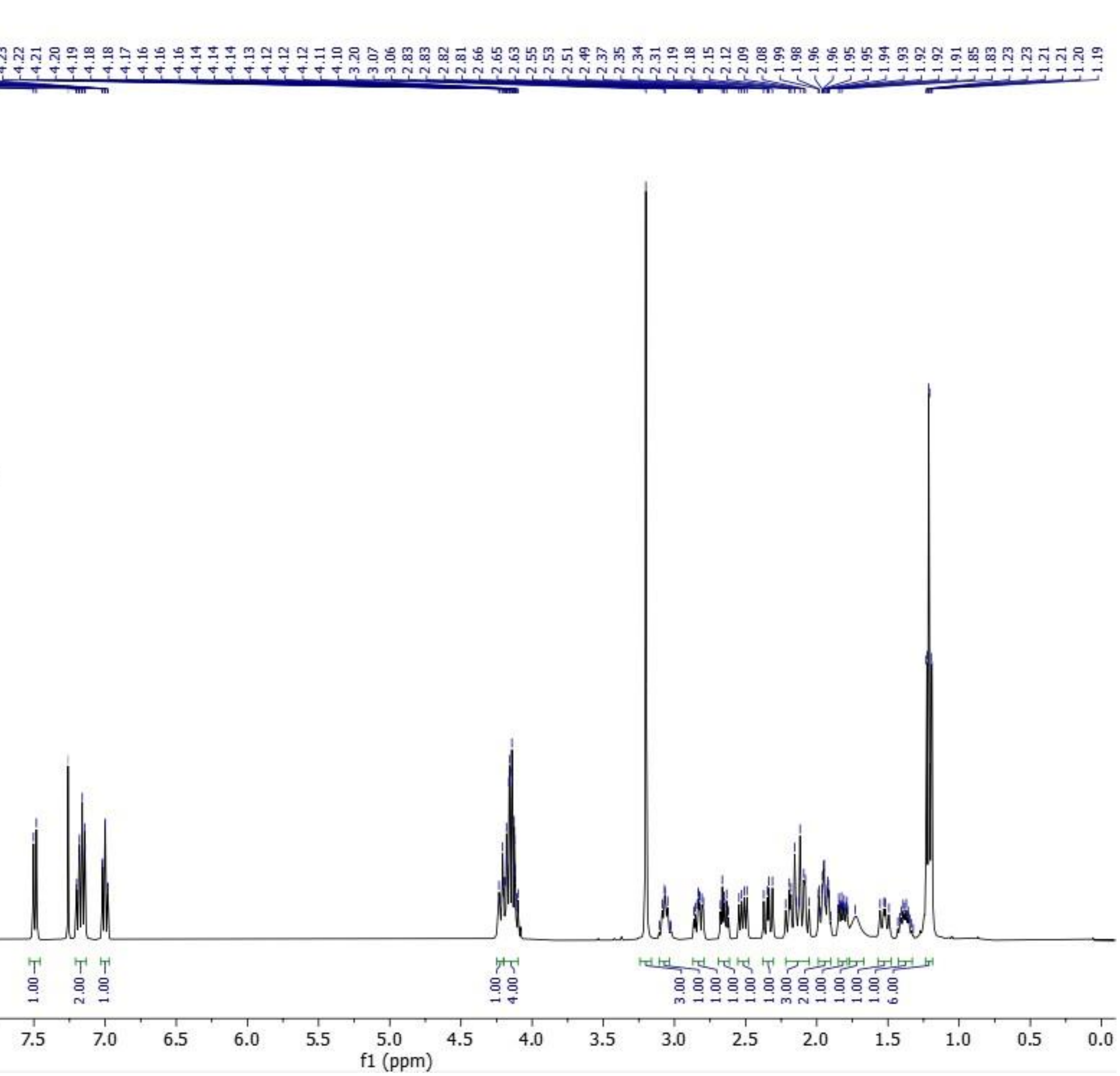

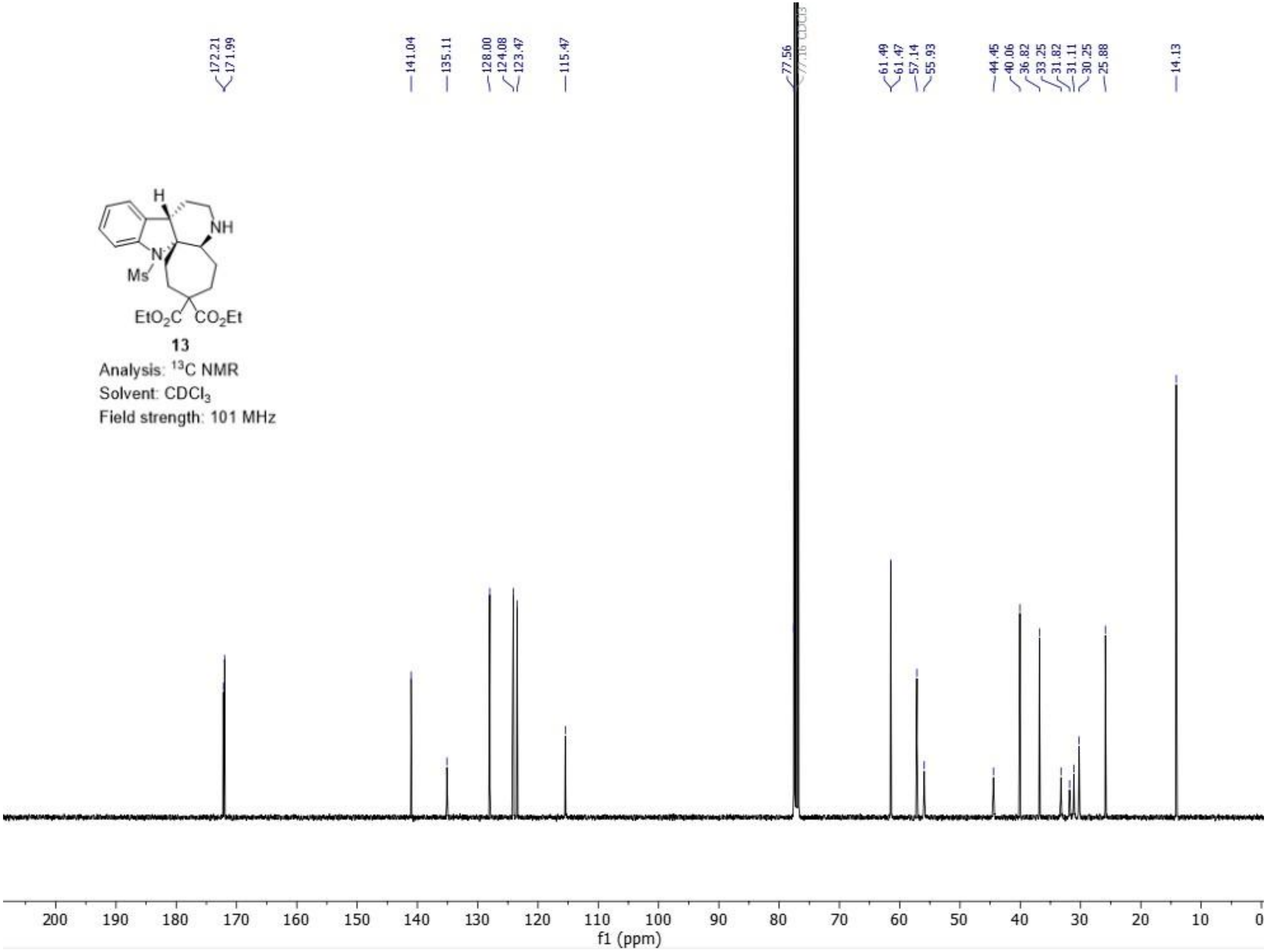
172.21
171.99
141.04
135.11
128.00
124.08
123.47
115.47
77.56
77.16 CDCl3
61.49
61.47
57.14
55.93
44.45
40.06
36.82
33.25
31.82
31.11
30.25
25.88
14.13
H
NH
N
Ms
EtO2C CO2Et
13
Analysis: 13C NMR
Solvent: CDCl3
Field strength: 101 MHz
200 190 180 170 160 150 140 130 120 110 100 90 80 70 60 50 40 30 20 10 0
f1 (ppm)

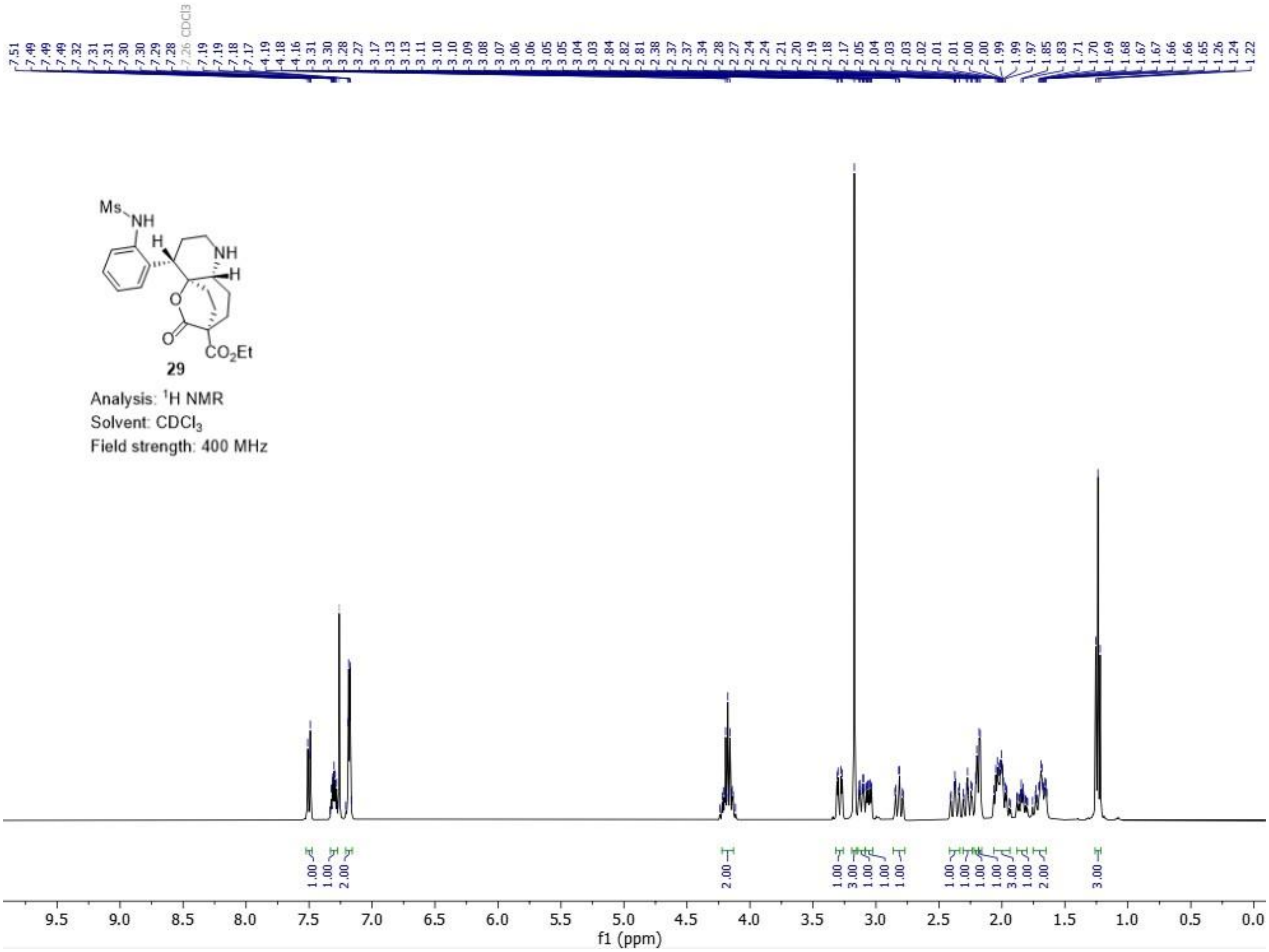

29

Analysis: ^{1}H NMR
Solvent: $CDCl_3$
Field strength: 400 MHz

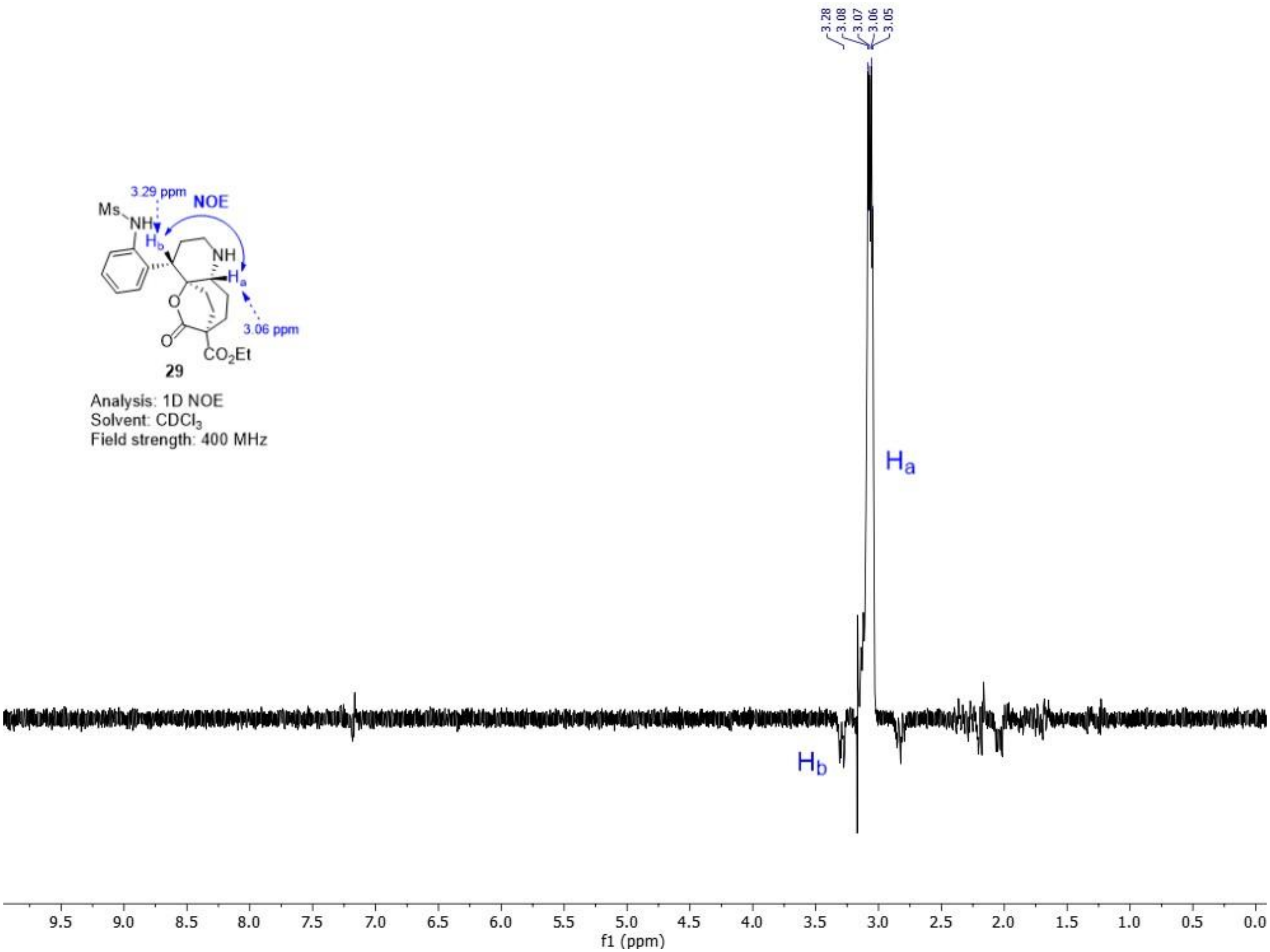
3.29 ppm
NOE
Ms
NH
H_b
NH
H_a
O
O
CO_2Et
3.06 ppm
29
Analysis: 1D NOE
Solvent: $CDCl_3$
Field strength: 400 MHz
3.28
3.08
3.07
3.06
3.05
H_a
H_b
9.5
9.0
8.5
8.0
7.5
7.0
6.5
6.0
5.5
5.0
4.5
4.0
3.5
3.0
2.5
2.0
1.5
1.0
0.5
0.0
f1 (ppm)

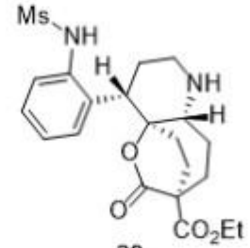

29

Analysis: ^{13}C NMR
Solvent: $CDCl_3$
Field strength: 101 MHz

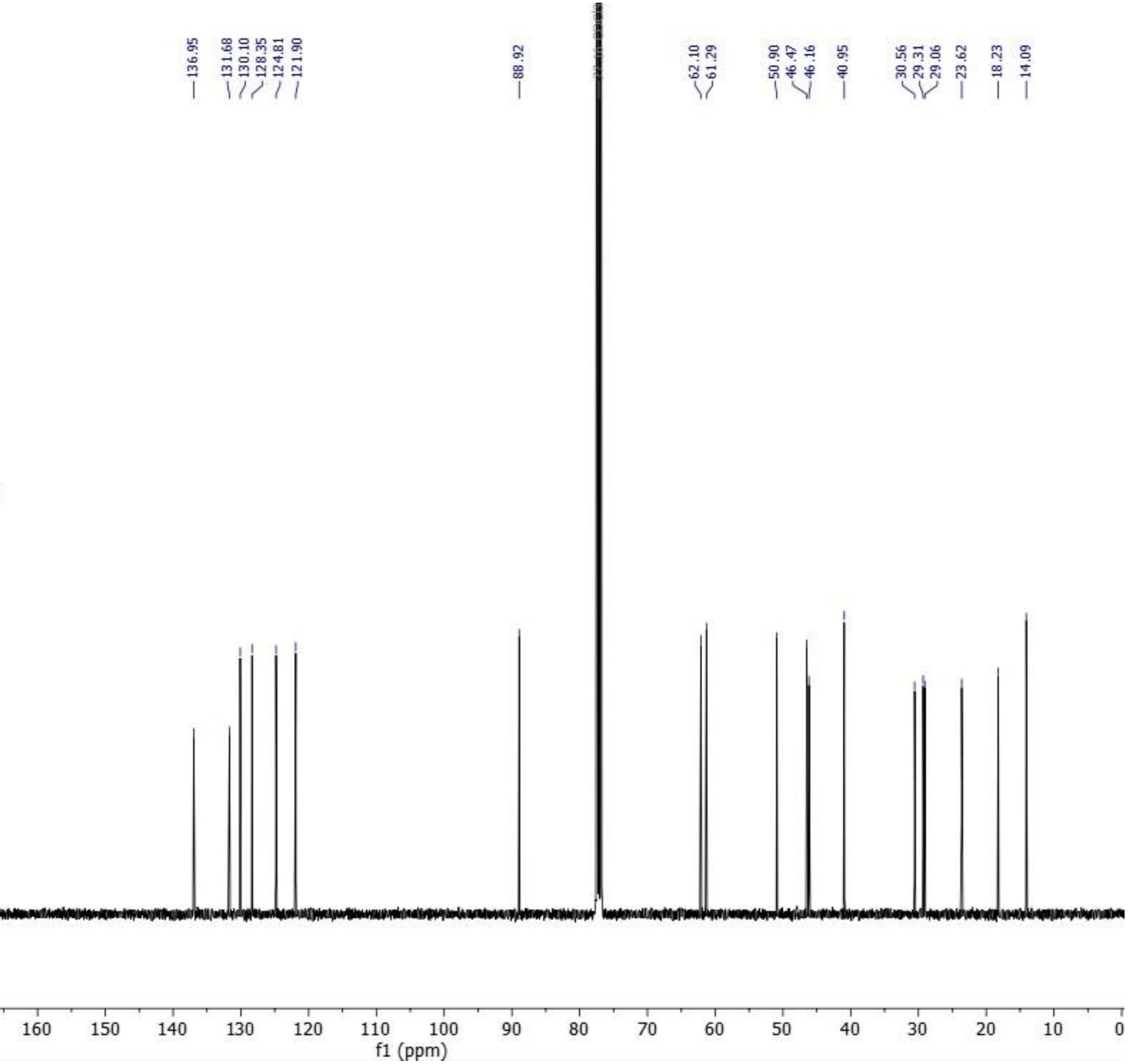

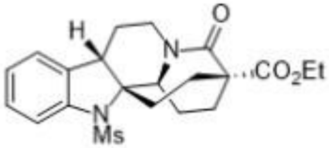

30

Analysis: ^{1}H NMR
Solvent: $CDCl_3$
Field strength: 400 MHz

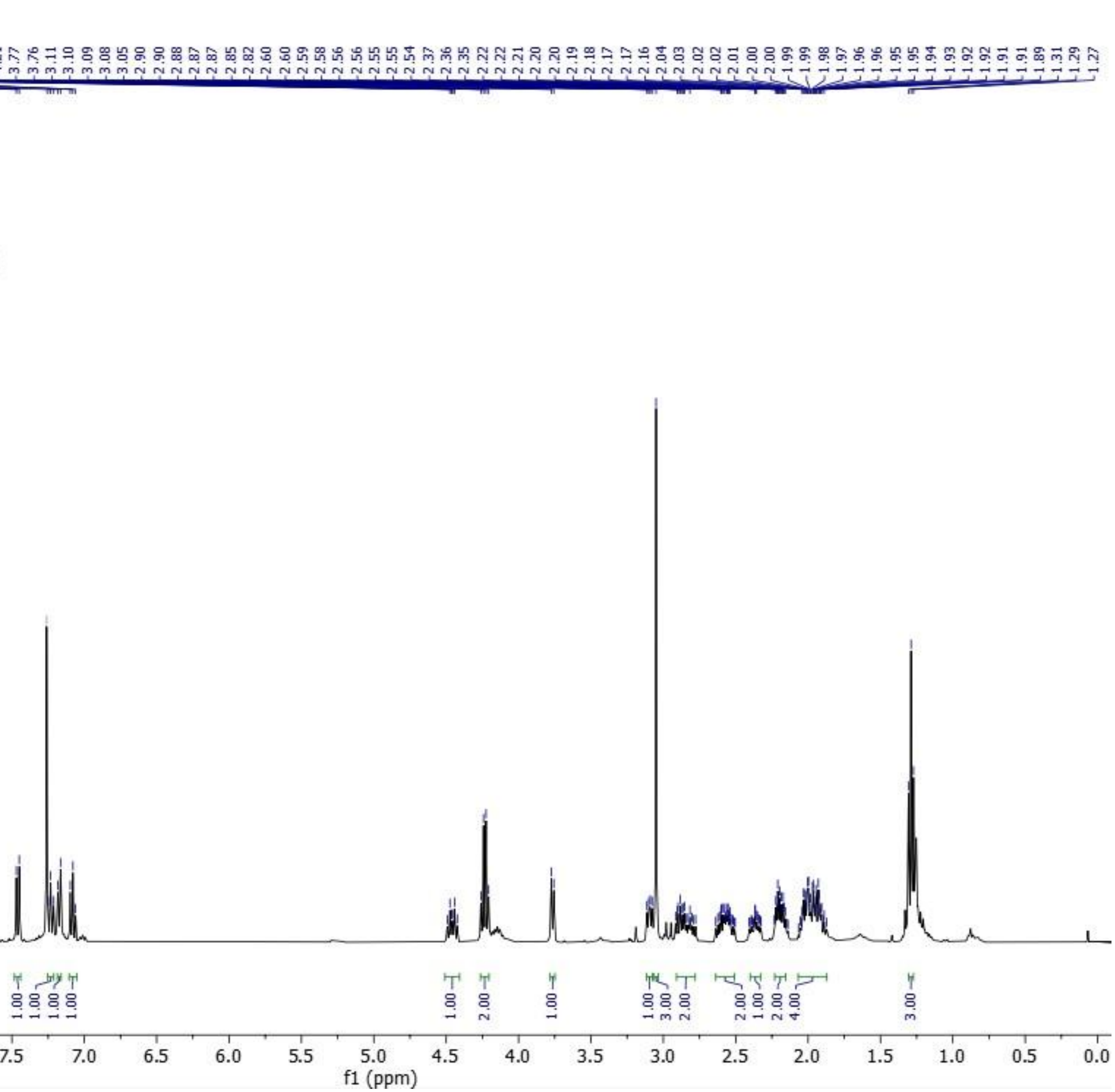

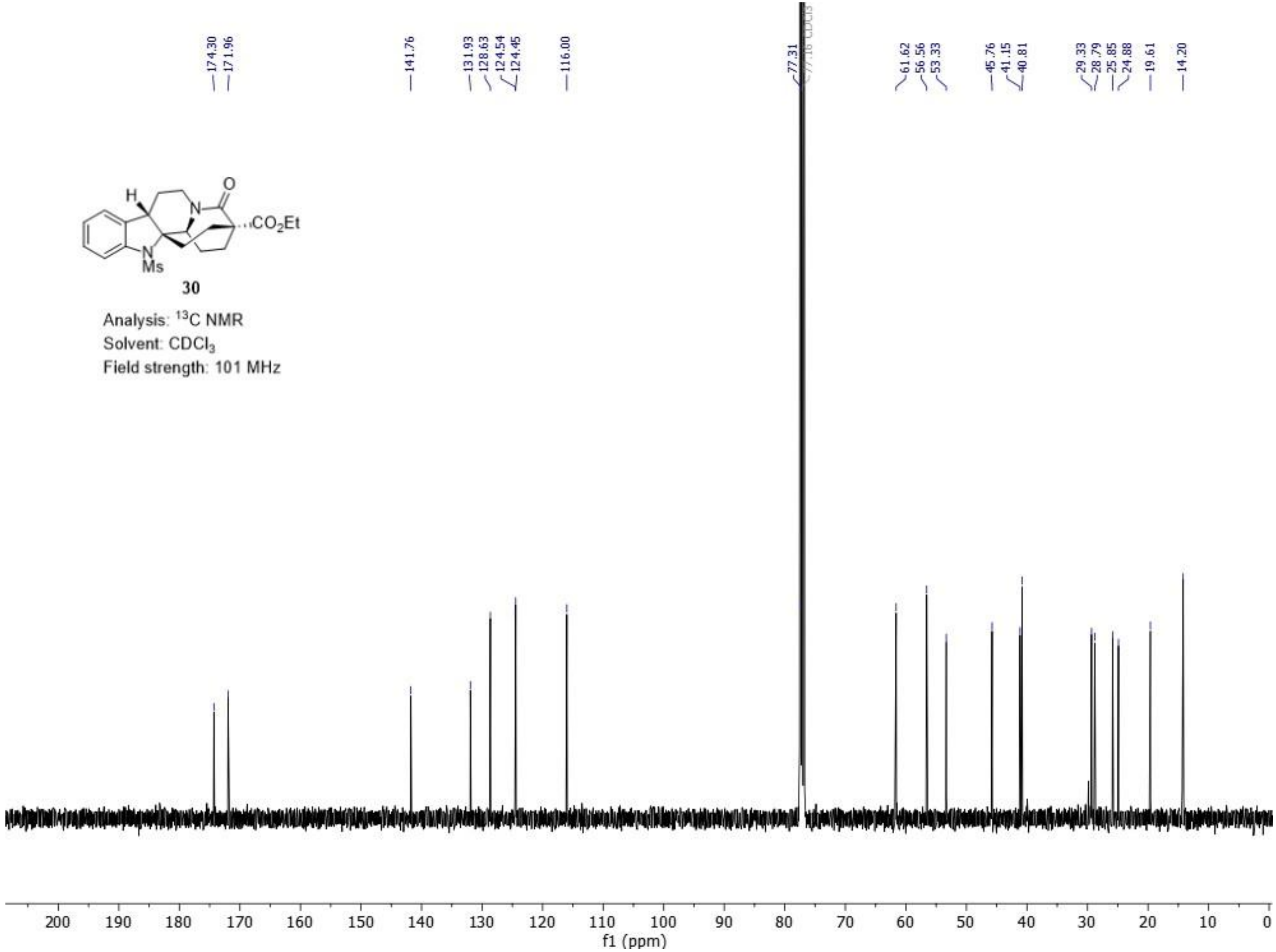

30

Analysis: ^{13}C NMR
Solvent: $CDCl_3$
Field strength: 101 MHz

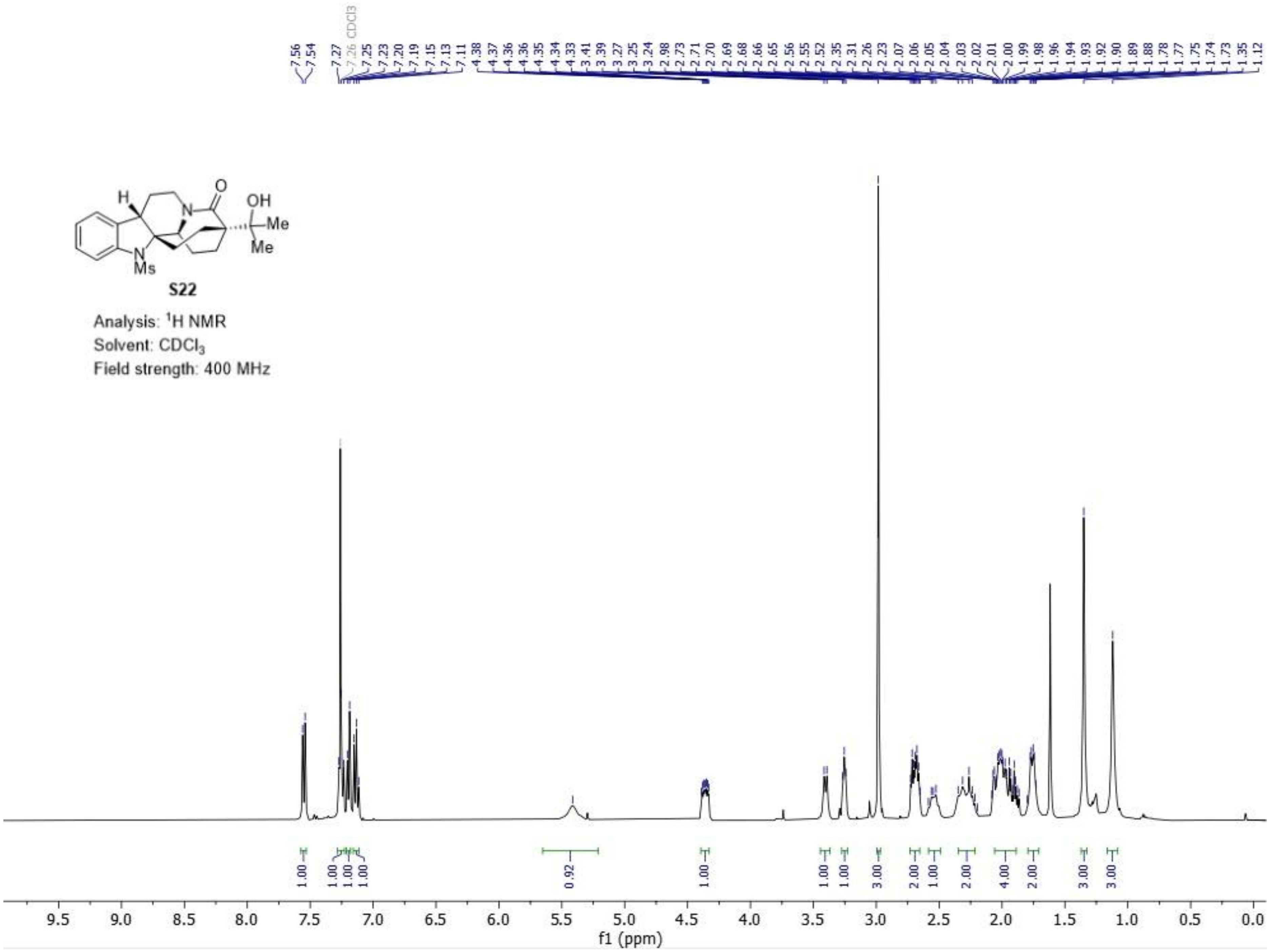
7.56 7.54 7.27 7.26 CDCl3 7.25 7.23 7.20 7.19 7.15 7.13 7.11 4.38 4.37 4.36 4.36 4.35 4.34 4.33 3.41 3.39 3.27 3.25 3.24 2.98 2.73 2.71 2.70 2.69 2.68 2.66 2.65 2.56 2.55 2.52 2.35 2.31 2.26 2.23 2.07 2.06 2.05 2.04 2.03 2.02 2.01 2.00 1.99 1.98 1.96 1.94 1.93 1.92 1.90 1.89 1.88 1.78 1.77 1.75 1.74 1.73 1.35 1.12
H
N
O
OH
Me
Me
N
Ms
S22
Analysis: 1H NMR
Solvent: CDCl3
Field strength: 400 MHz
1.00 1.00 1.00 1.00 0.92 1.00 1.00 1.00 3.00 2.00 1.00 2.00 4.00 2.00 3.00 3.00
9.5 9.0 8.5 8.0 7.5 7.0 6.5 6.0 5.5 5.0 4.5 4.0 3.5 3.0 2.5 2.0 1.5 1.0 0.5 0.0
f1 (ppm)

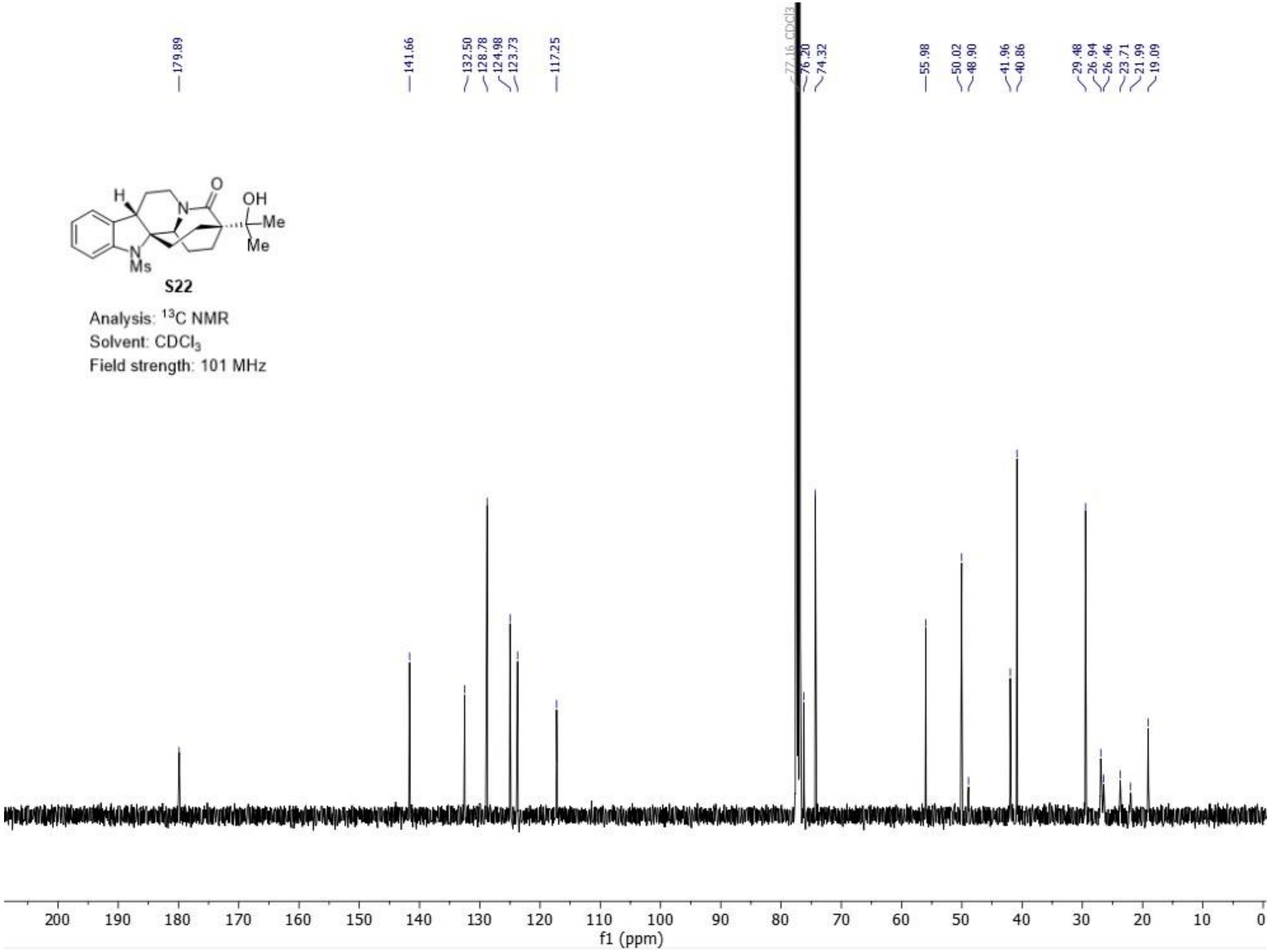

179.89
141.66
132.50
128.78
124.98
123.73
117.25
77.16 CDCl3
76.20
74.32
55.98
50.02
48.90
41.96
40.86
29.48
26.94
26.46
23.71
21.99
19.09
S22
Analysis: 13C NMR
Solvent: CDCl3
Field strength: 101 MHz
f1 (ppm)

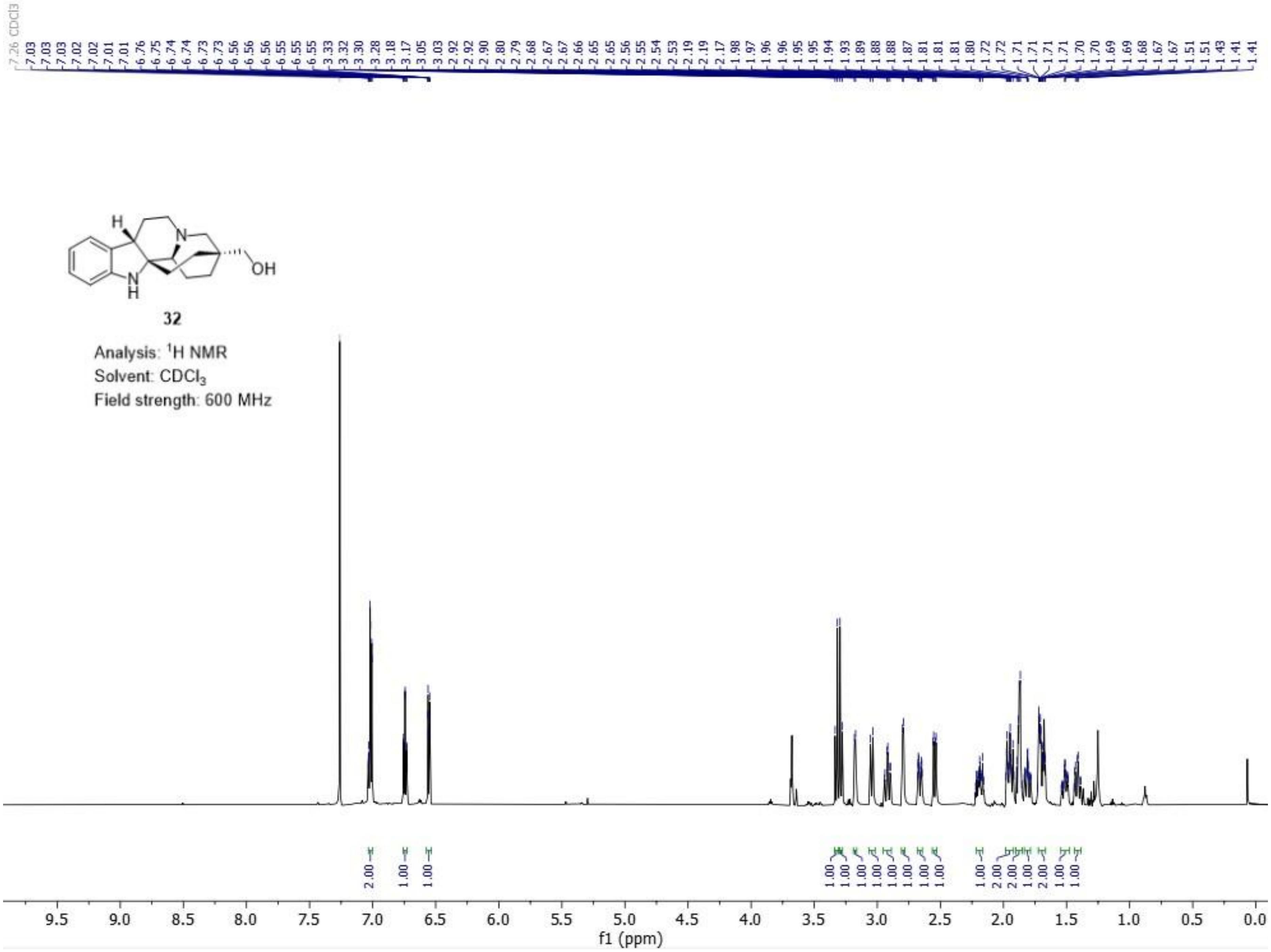

7.26 CDCl3
7.03 7.03 7.03 7.02 7.02 7.01 7.01 6.76 6.75 6.74 6.74 6.73 6.73 6.56 6.56 6.56 6.55 6.55 6.55 3.33 3.32 3.30 3.28 3.18 3.17 3.05 3.03 2.92 2.92 2.90 2.80 2.79 2.68 2.67 2.67 2.66 2.65 2.65 2.56 2.55 2.54 2.53 2.19 2.19 2.17 1.98 1.97 1.96 1.96 1.95 1.95 1.94 1.93 1.89 1.88 1.88 1.87 1.81 1.81 1.81 1.80 1.72 1.72 1.71 1.71 1.71 1.71 1.70 1.70 1.69 1.69 1.68 1.67 1.67 1.51 1.51 1.43 1.41 1.41
H
N
OH
N
H
32
Analysis: 1H NMR
Solvent: CDCl3
Field strength: 600 MHz
f1 (ppm)

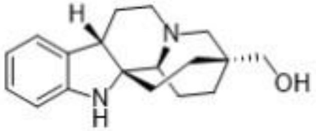

32

Analysis: ^{13}C NMR
Solvent: $CDCl_3$
Field strength: 151 MHz

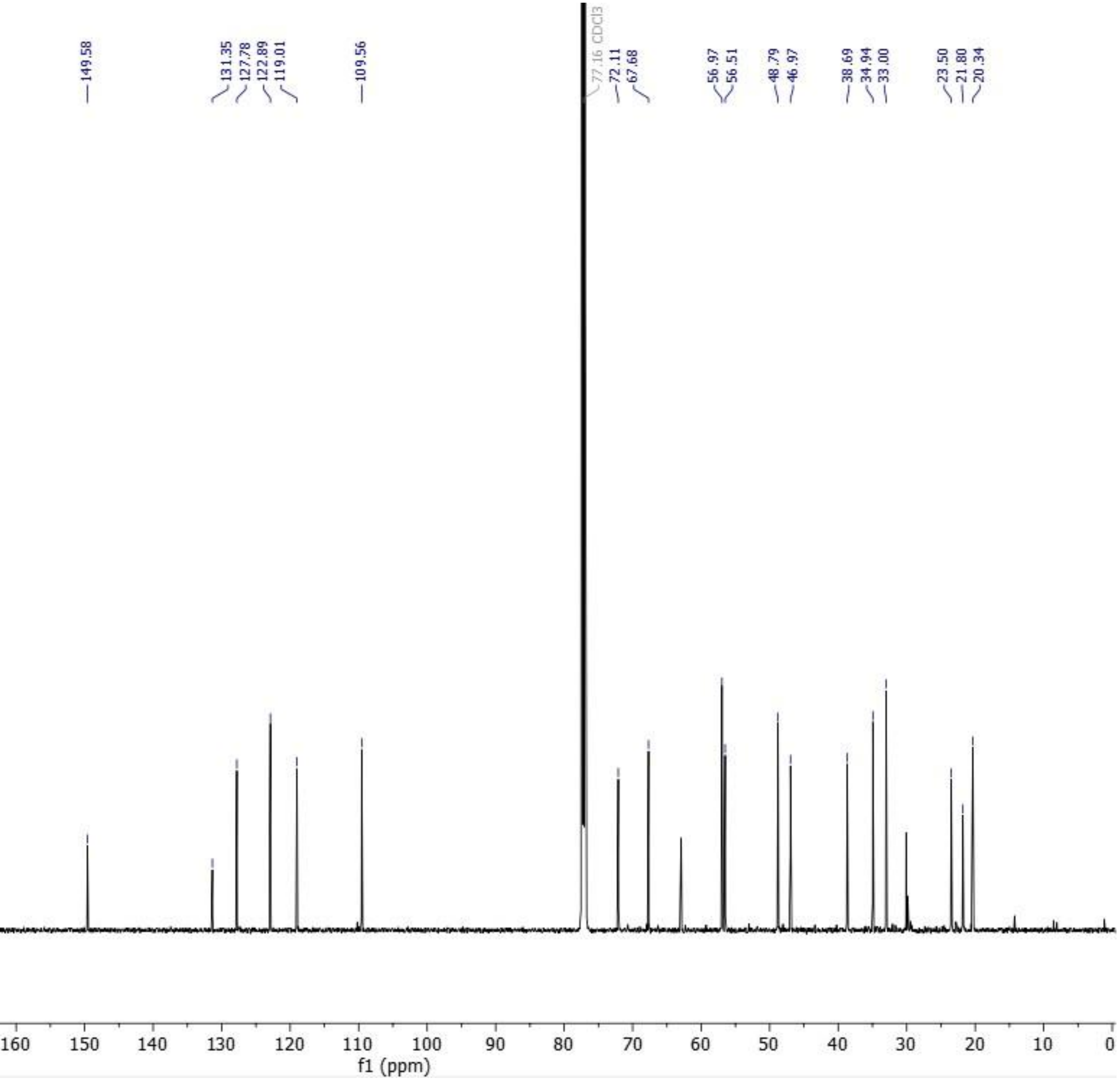

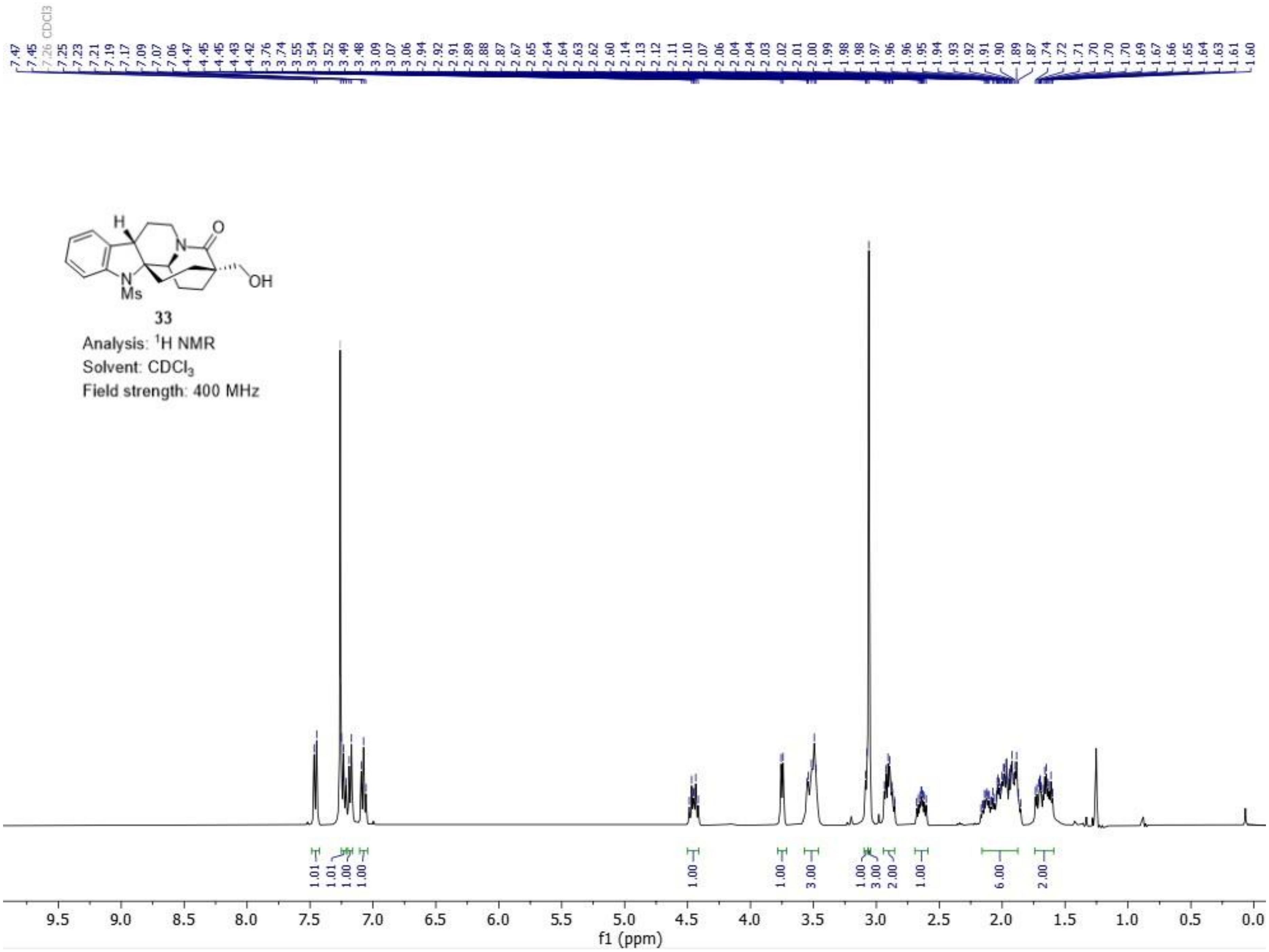
33
Analysis: 1H NMR
Solvent: CDCl3
Field strength: 400 MHz
f1 (ppm)

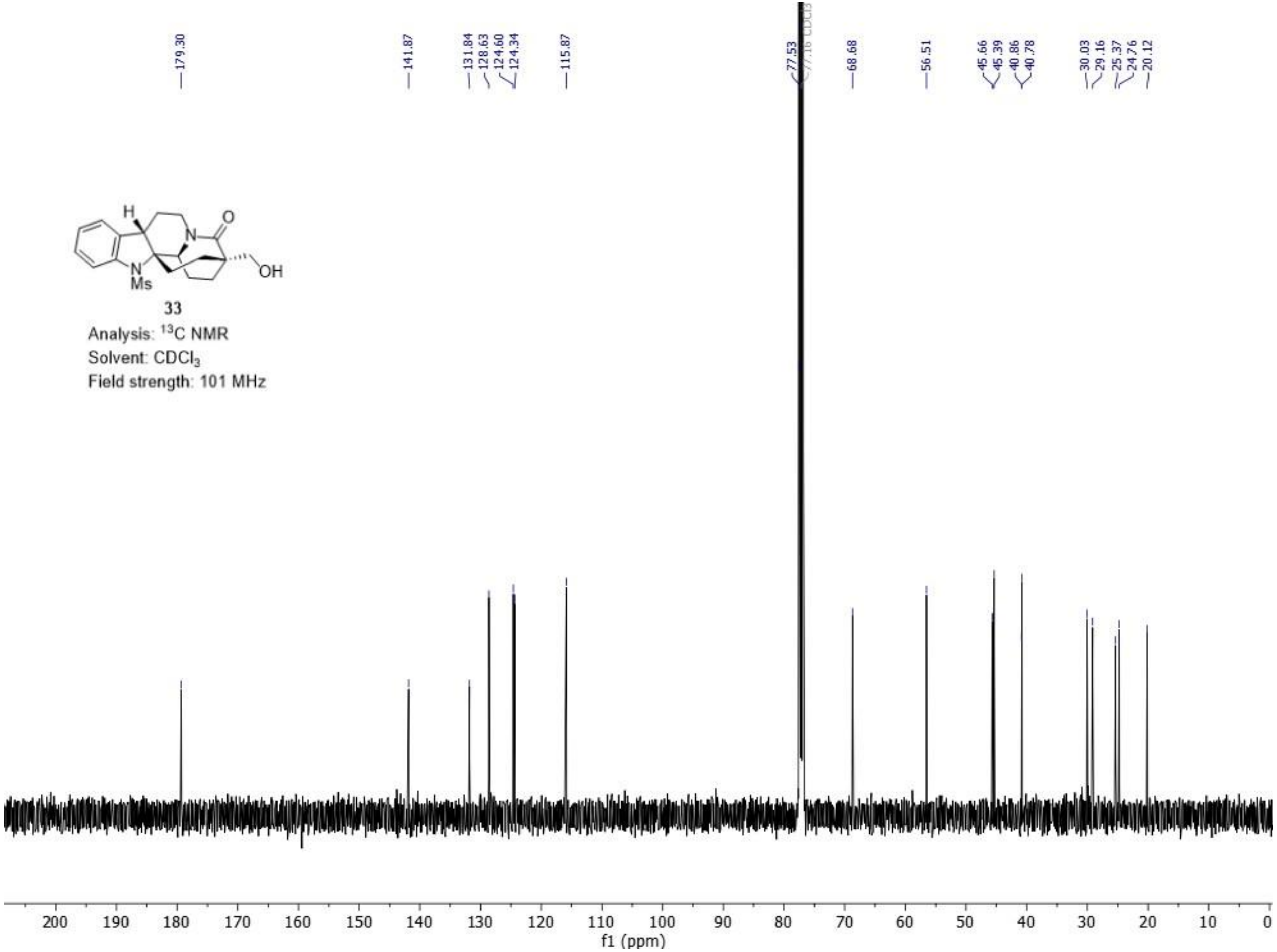

33

Analysis: ^{13}C NMR

Solvent: $CDCl_3$

Field strength: 101 MHz

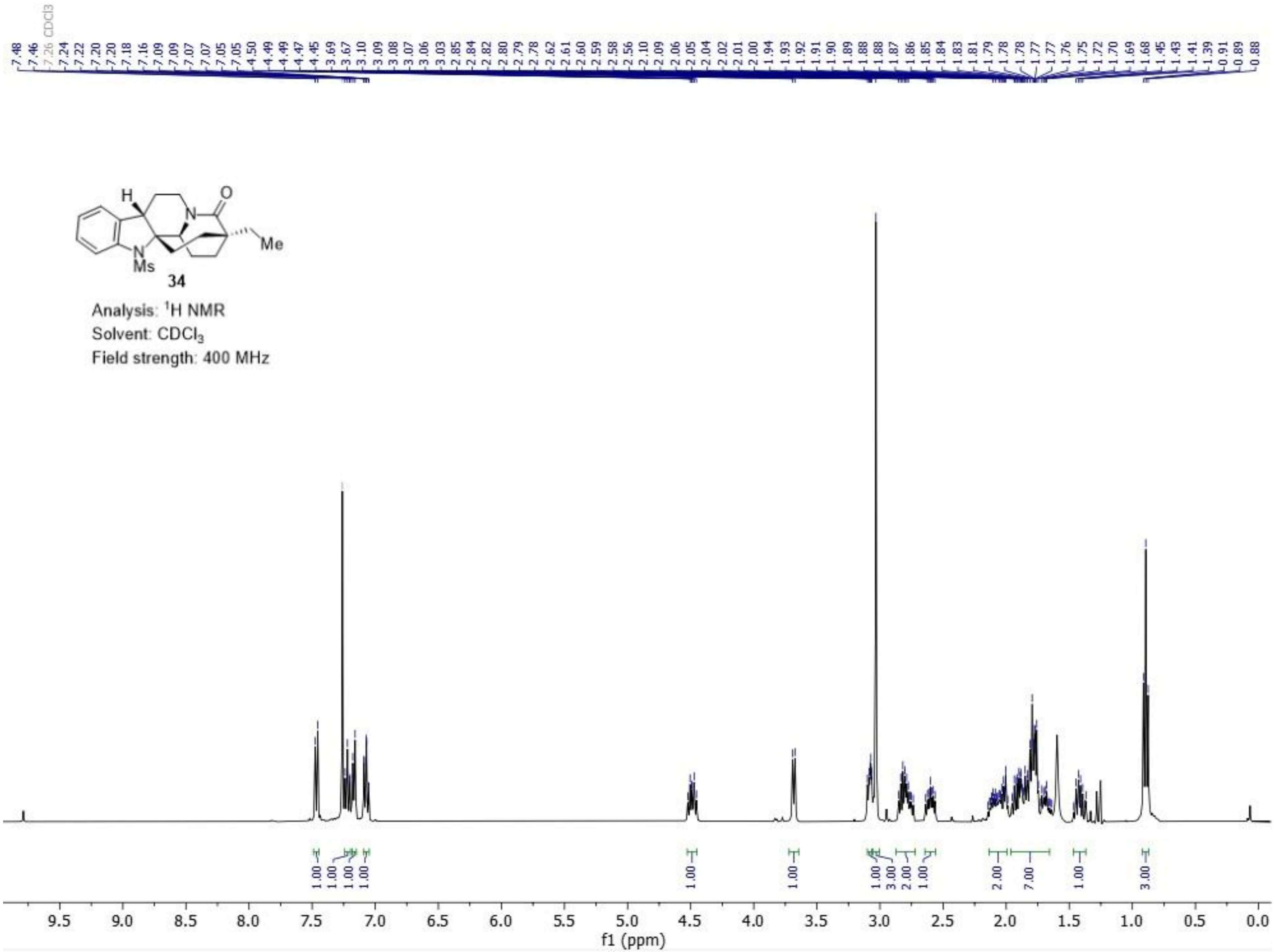
7.48 7.46 7.26 CDCl3 7.24 7.22 7.20 7.20 7.18 7.16 7.09 7.09 7.07 7.07 7.05 7.05 4.50 4.49 4.49 4.47 4.45 3.69 3.67 3.10 3.09 3.08 3.07 3.06 3.03 2.85 2.84 2.82 2.80 2.79 2.78 2.62 2.61 2.60 2.59 2.58 2.56 2.10 2.09 2.06 2.05 2.04 2.02 2.01 2.00 1.94 1.93 1.92 1.91 1.90 1.89 1.88 1.88 1.87 1.86 1.85 1.84 1.83 1.81 1.79 1.78 1.78 1.77 1.77 1.76 1.75 1.72 1.70 1.69 1.68 1.45 1.43 1.41 1.39 0.91 0.89 0.88
H
N
O
Me
N
Ms
34
Analysis: 1H NMR
Solvent: CDCl3
Field strength: 400 MHz
1.00 1.00 1.00 1.00 1.00 1.00 1.00 3.00 2.00 1.00 2.00 7.00 1.00 3.00
9.5 9.0 8.5 8.0 7.5 7.0 6.5 6.0 5.5 5.0 4.5 4.0 3.5 3.0 2.5 2.0 1.5 1.0 0.5 0.0
f1 (ppm)

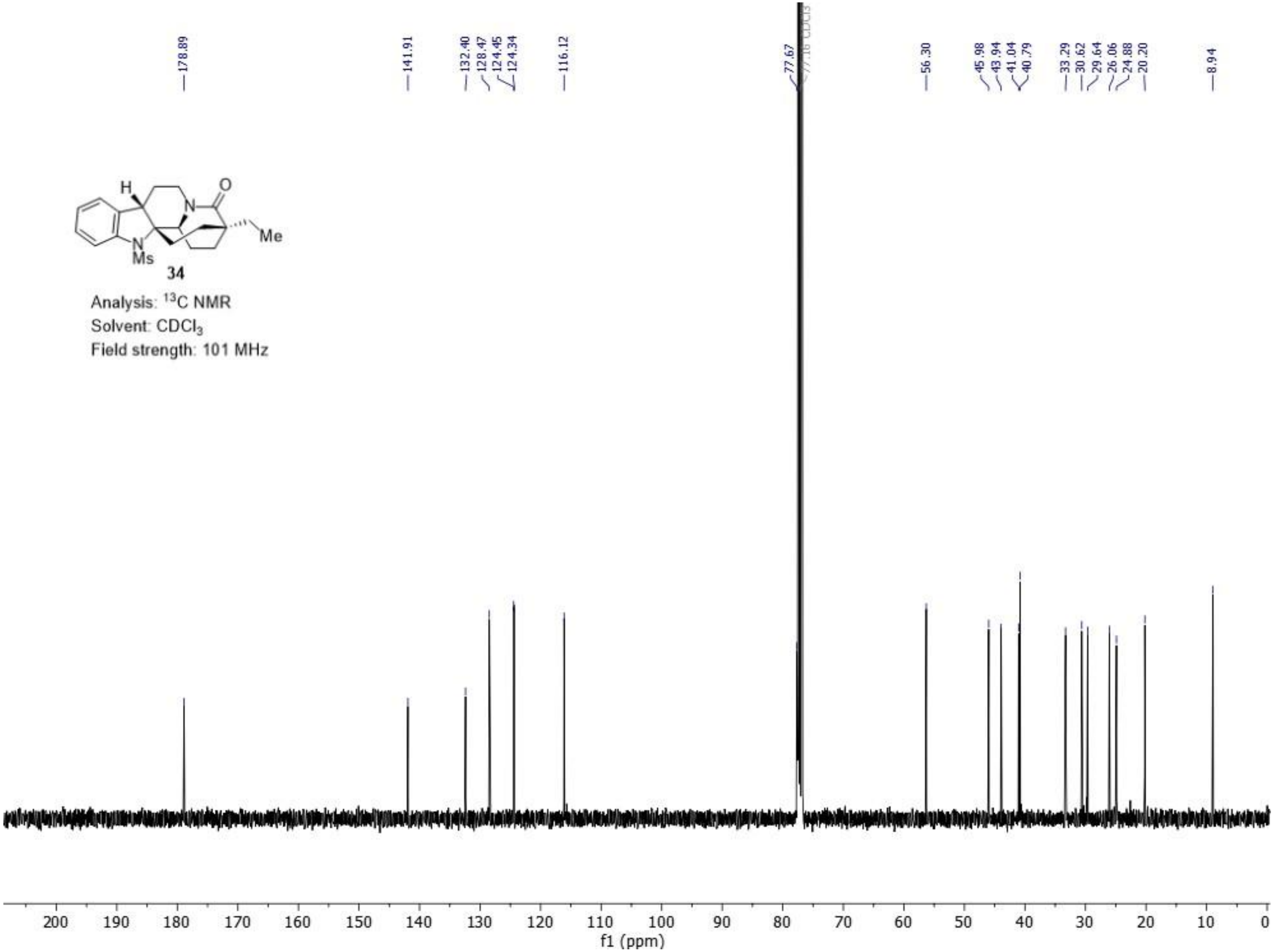

34
178.89
141.91
132.40
128.47
124.45
124.34
116.12
77.67
77.16 CDCl3
56.30
45.98
43.94
41.04
40.79
33.29
30.62
29.64
26.06
24.88
20.20
8.94
Analysis: 13C NMR
Solvent: CDCl3
Field strength: 101 MHz
200 190 180 170 160 150 140 130 120 110 100 90 80 70 60 50 40 30 20 10 0
f1 (ppm)

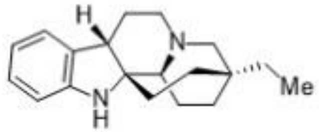

1 (+)-melonine

Analysis: ^{1}H NMR
Solvent: $CDCl_3$
Field strength: 500 MHz

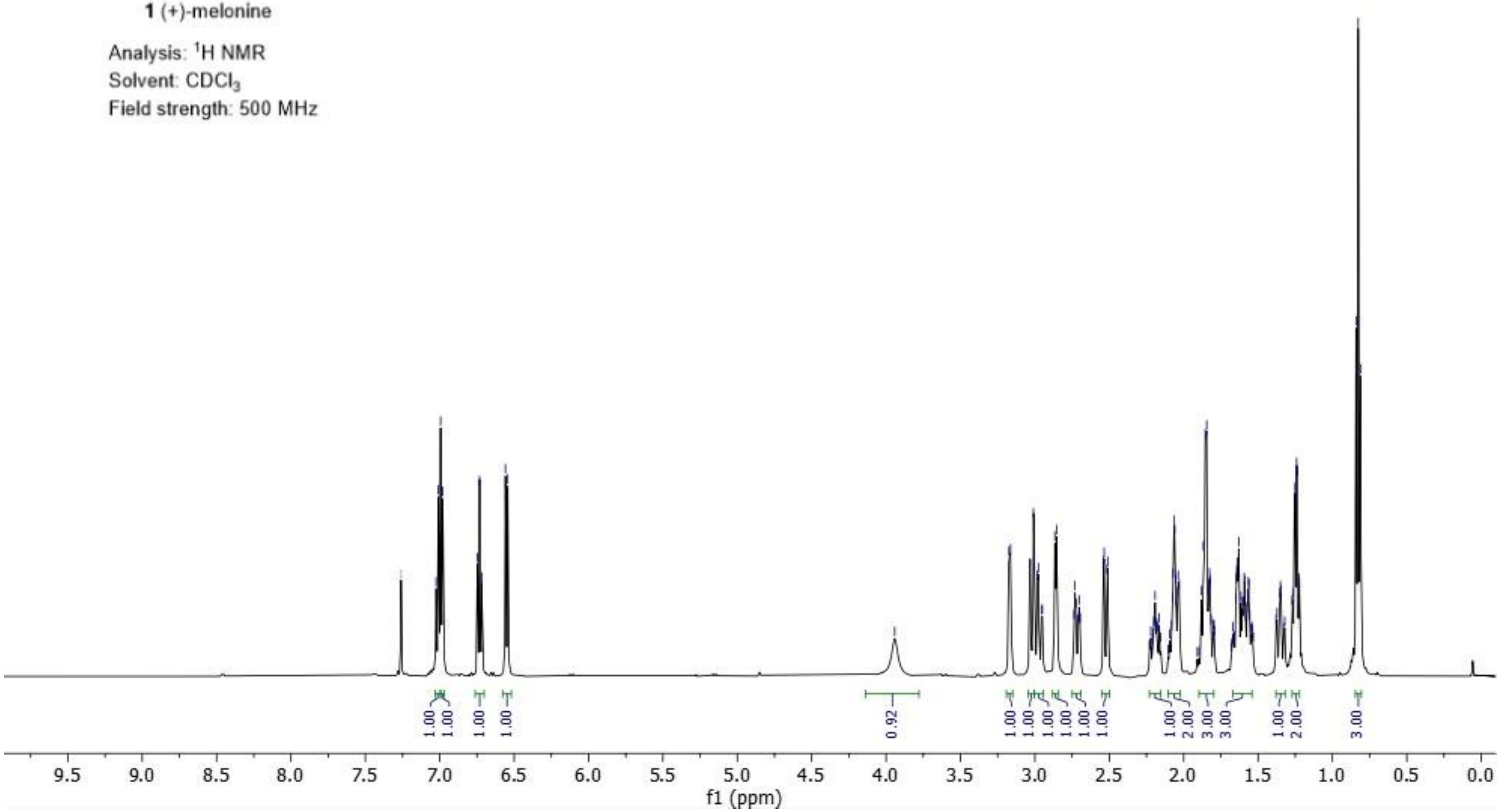

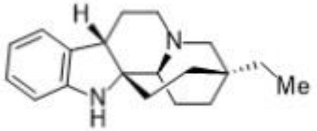

1 (+)-melonine

Analysis: ^{13}C NMR
Solvent: $CDCl_3$
Field strength: 126 MHz

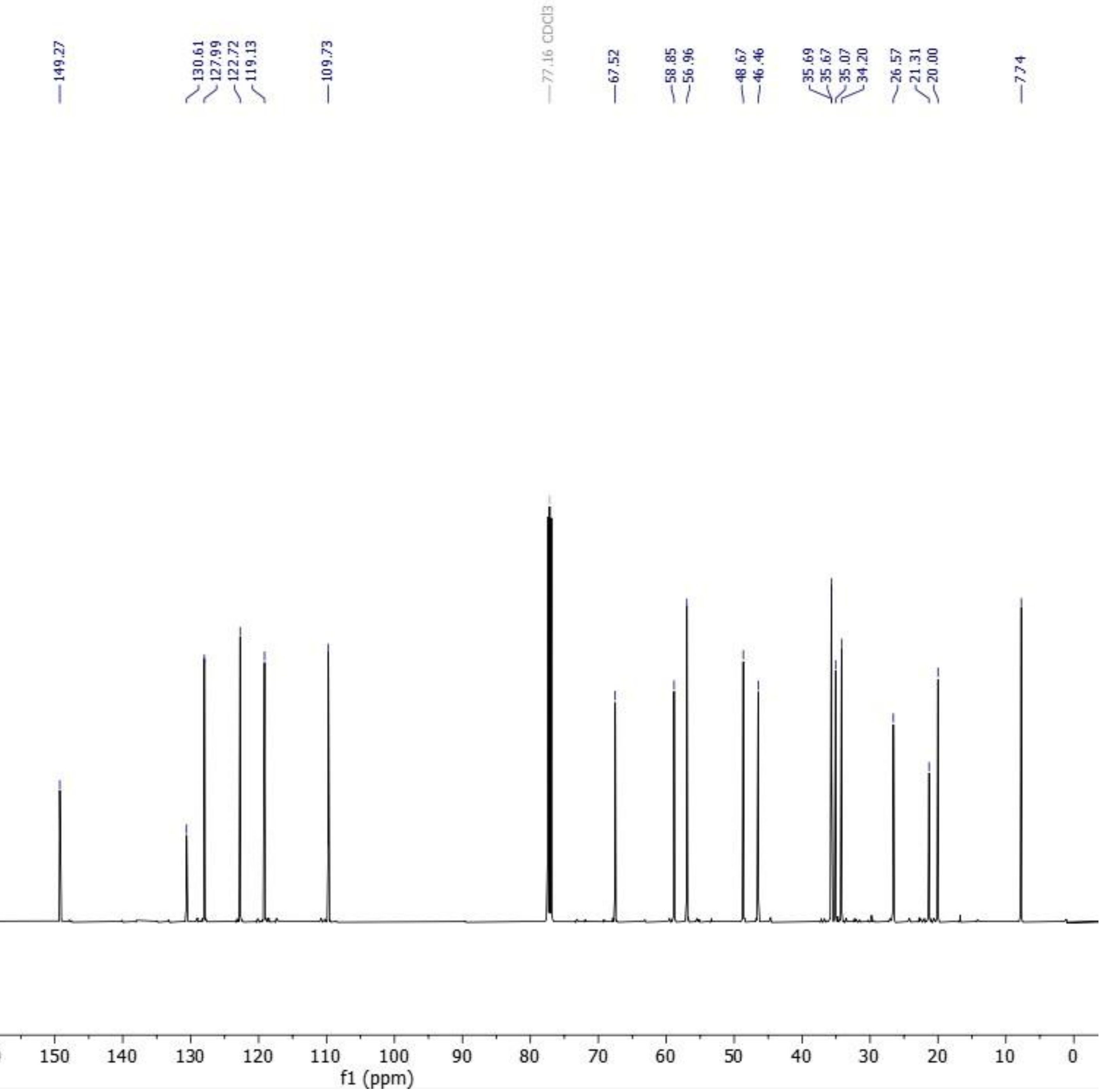

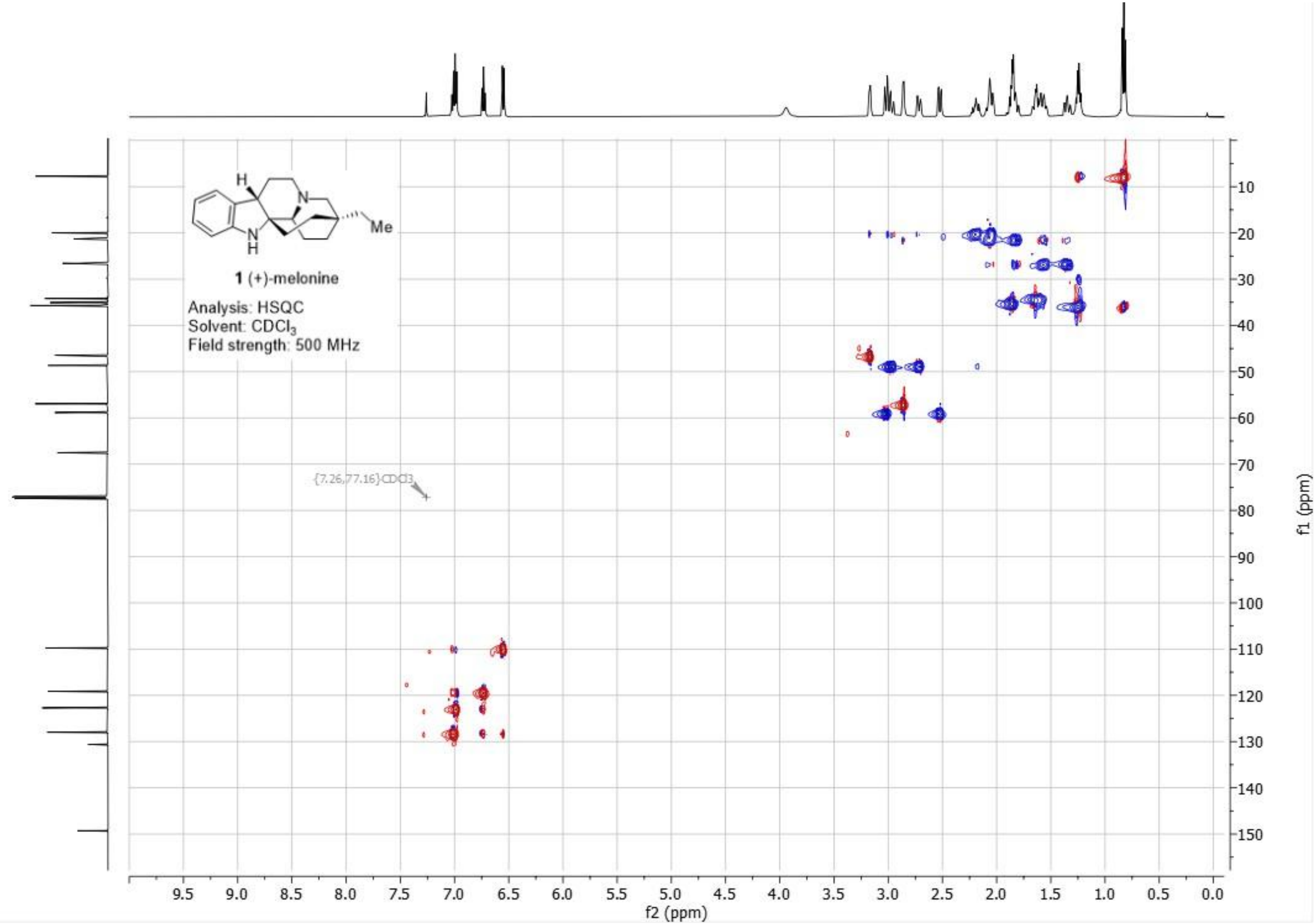

1 (+)-melonine
Analysis: HSQC
Solvent: CDCl3
Field strength: 500 MHz
{7.26,77.16}CDCl3
f2 (ppm)
f1 (ppm)

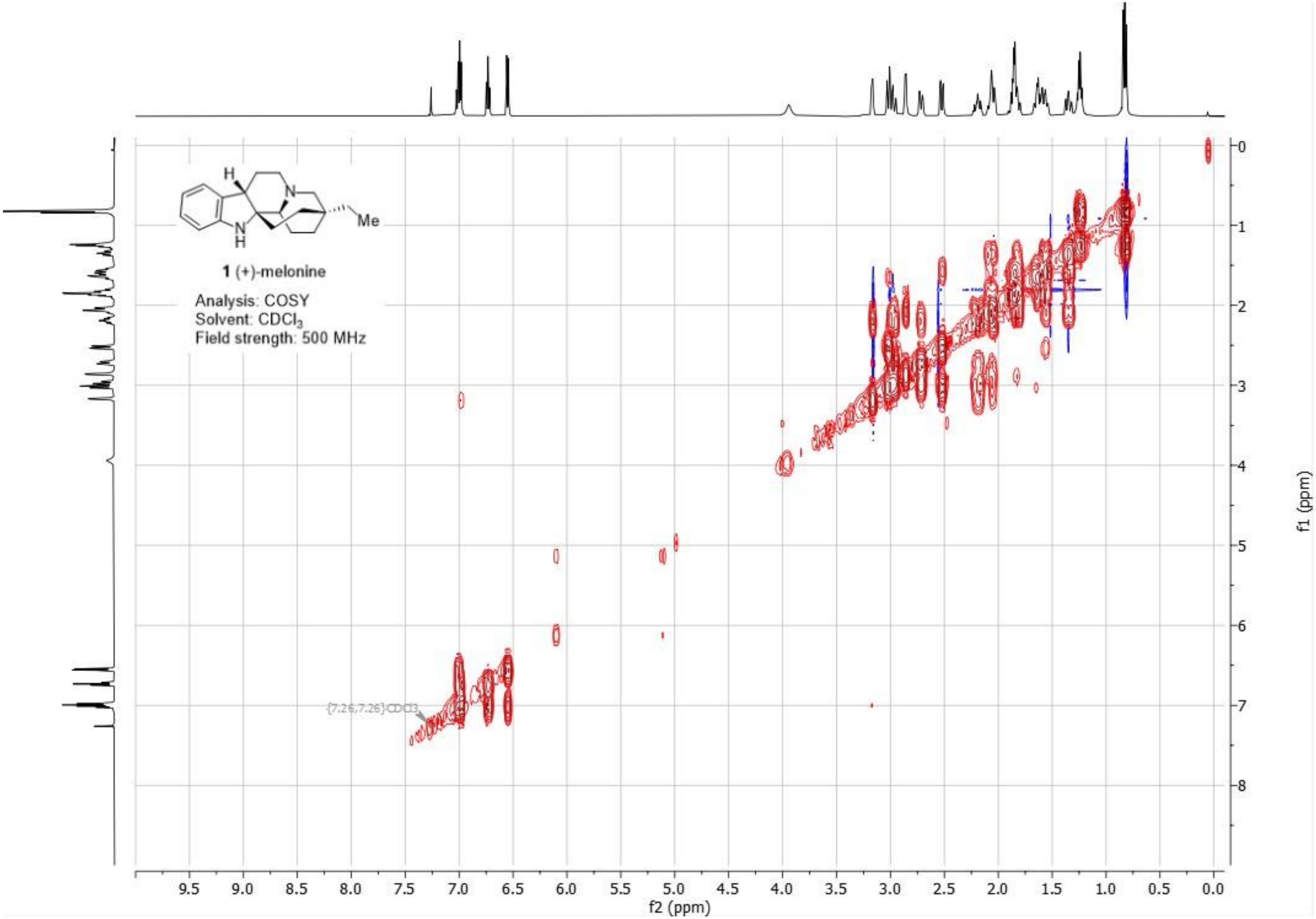

1 (+)-melonine
Analysis: COSY
Solvent: CDCl3
Field strength: 500 MHz
{7.26,7.26}CDCl3
f2 (ppm)
f1 (ppm)

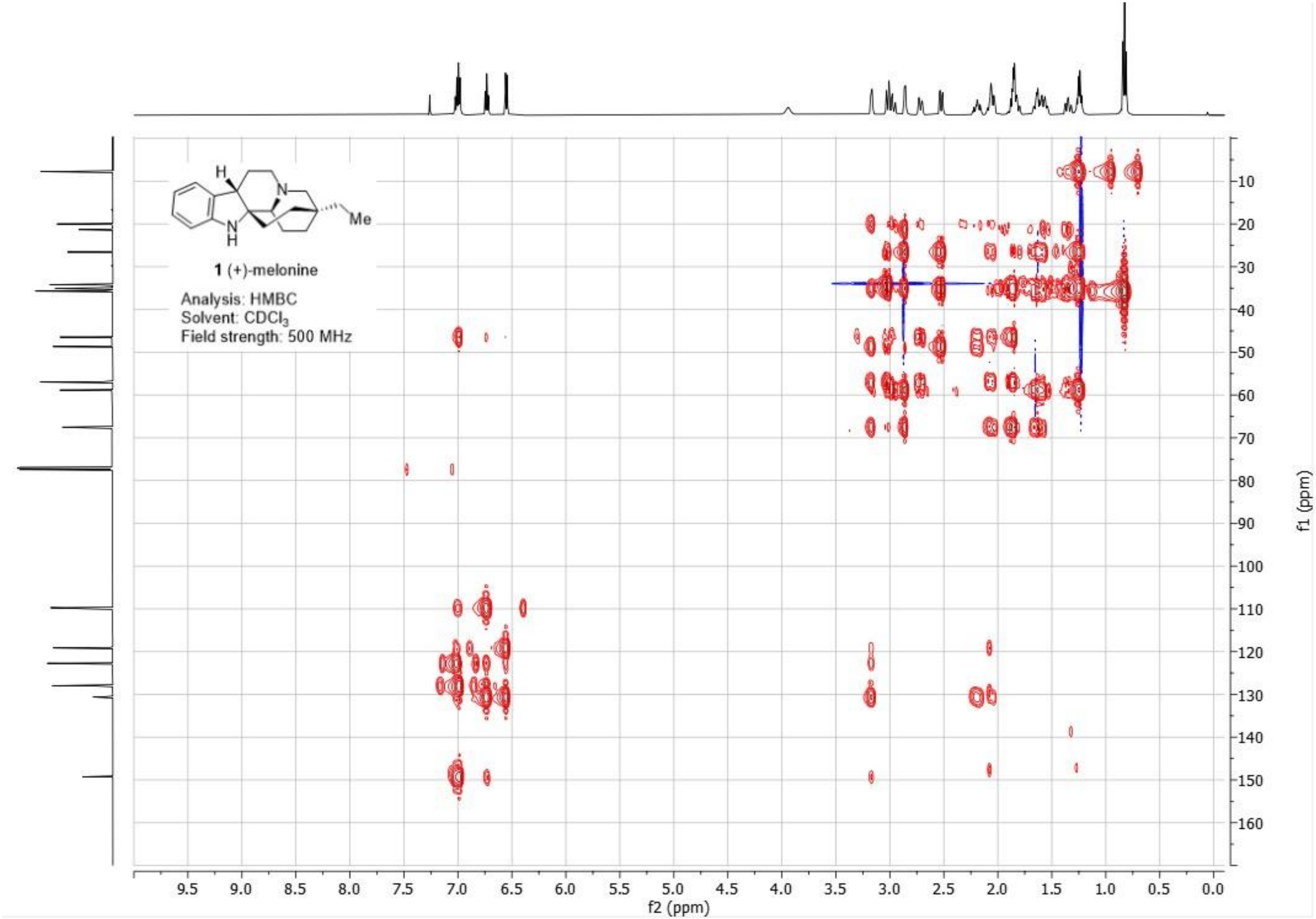

H
N
Me
N
H
1 (+)-melonine
Analysis: HMBC
Solvent: CDCl3
Field strength: 500 MHz
f1 (ppm)
f2 (ppm)

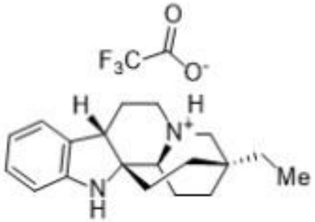

1·TFA (+)-melonine·TFA

Analysis: ^{1}H NMR
Solvent: CD_3CN
Field strength: 600 MHz

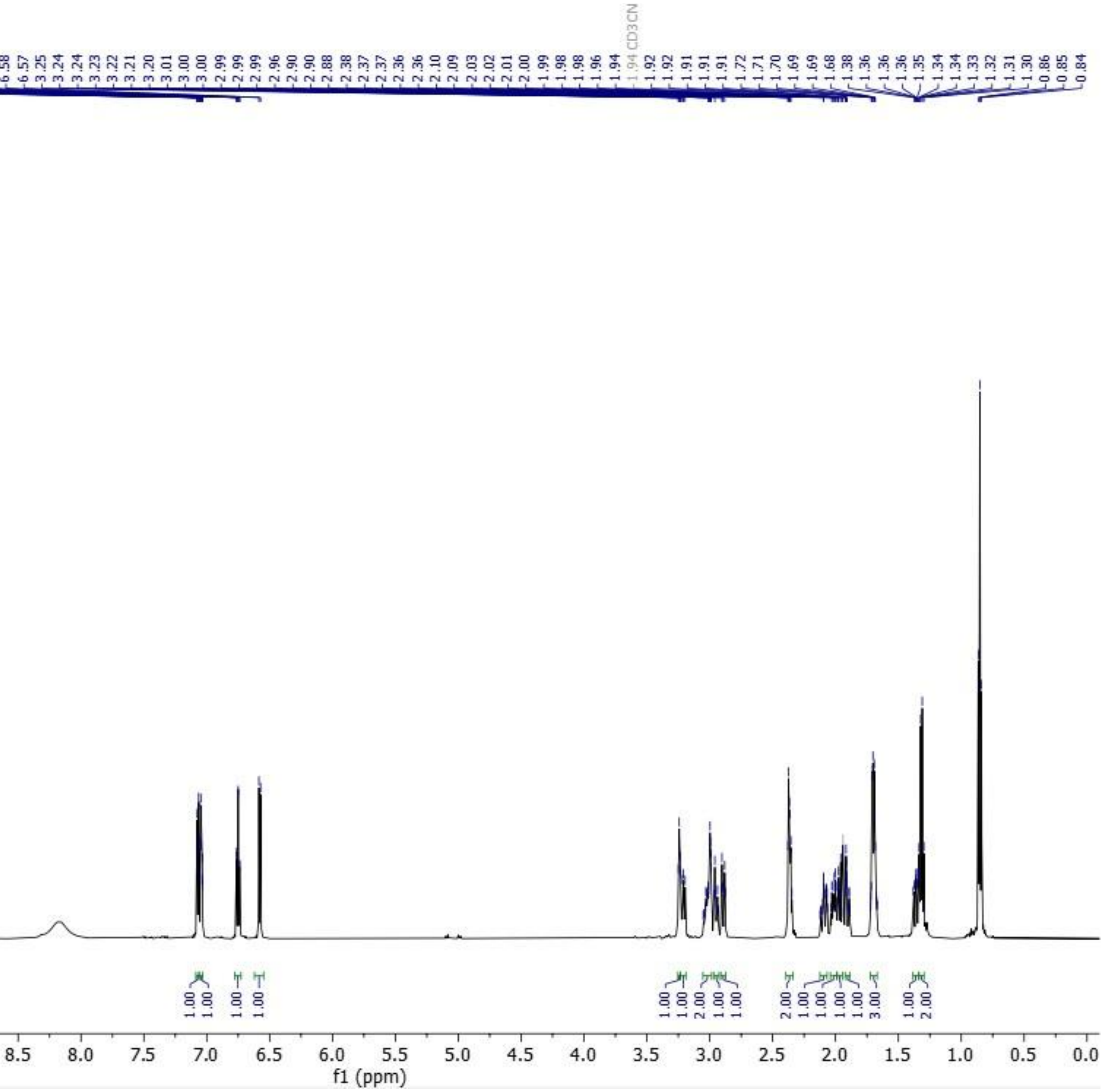

1·TFA (+)-melonine·TFA

Analysis: ^{13}C NMR
Solvent: CD_3CN
Field strength: 151 MHz

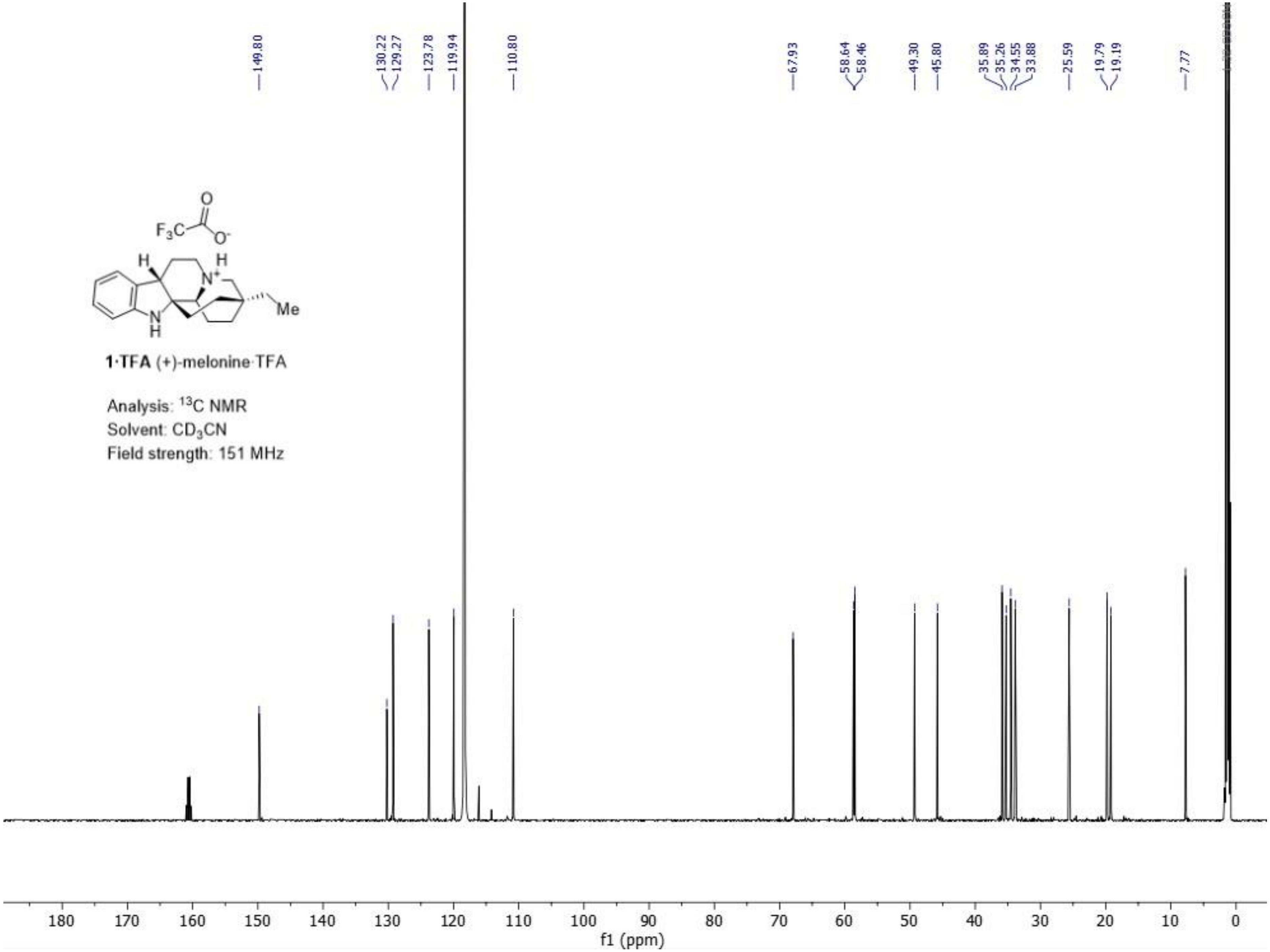

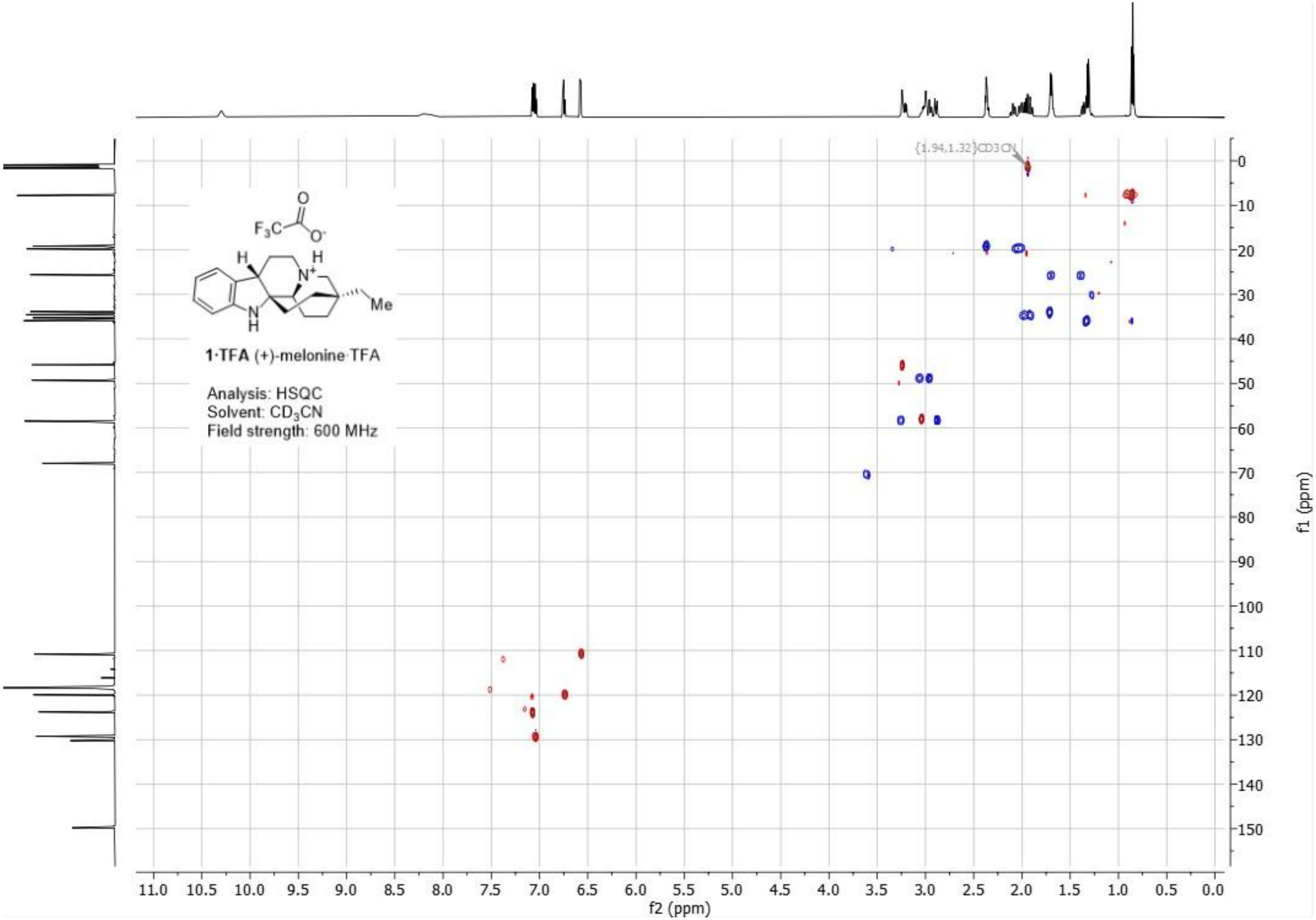
{1.94,1.32}CD3CN
1·TFA (+)-melonine·TFA
Analysis: HSQC
Solvent: CD3CN
Field strength: 600 MHz
f2 (ppm)
f1 (ppm)

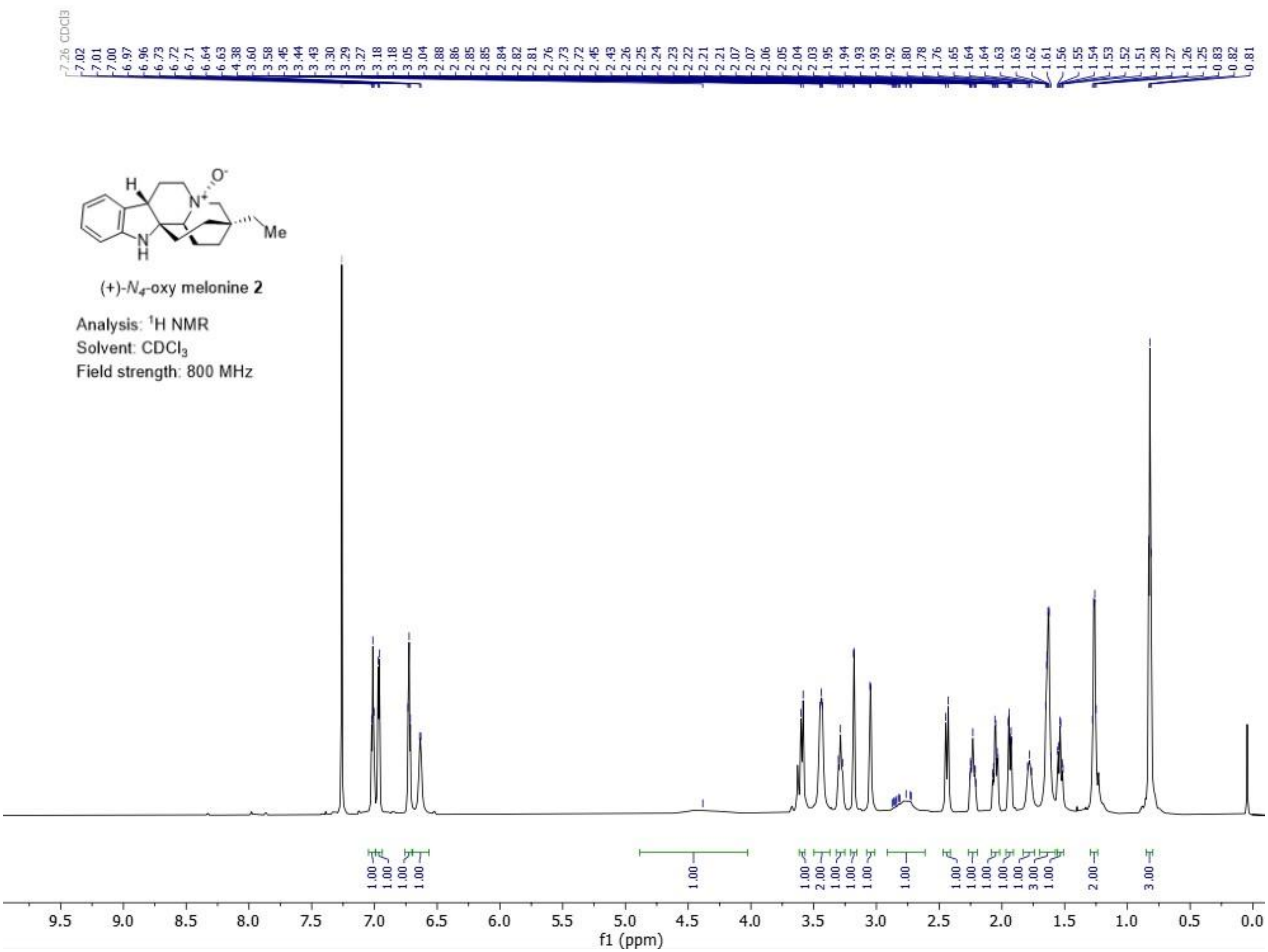
7.26 CDCl3
7.02 7.01 7.00 6.97 6.96 6.73 6.72 6.71 6.64 6.63 4.38 3.60 3.58 3.45 3.44 3.43 3.30 3.29 3.27 3.18 3.18 3.05 3.04 2.88 2.86 2.85 2.85 2.84 2.82 2.81 2.76 2.73 2.72 2.45 2.43 2.26 2.25 2.24 2.23 2.22 2.21 2.21 2.07 2.07 2.06 2.05 2.04 2.03 1.95 1.94 1.93 1.93 1.92 1.80 1.78 1.76 1.65 1.64 1.64 1.63 1.63 1.62 1.61 1.56 1.55 1.54 1.53 1.52 1.51 1.28 1.27 1.26 1.25 0.83 0.82 0.81
H
O⁻
N⁺
Me
N
H
(+)-N4-oxy melonine 2
Analysis: 1H NMR
Solvent: CDCl3
Field strength: 800 MHz
1.00 1.00 1.00 1.00 1.00 1.00 2.00 1.00 1.00 1.00 1.00 1.00 1.00 1.00 1.00 3.00 1.00 2.00 3.00
9.5 9.0 8.5 8.0 7.5 7.0 6.5 6.0 5.5 5.0 4.5 4.0 3.5 3.0 2.5 2.0 1.5 1.0 0.5 0.0
f1 (ppm)

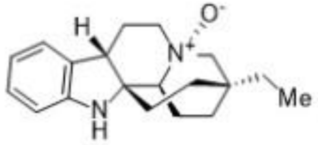

(+)-N_4-oxy melonine **2**

Analysis: ^{13}C NMR
Solvent: $CDCl_3$
Field strength: 201 MHz

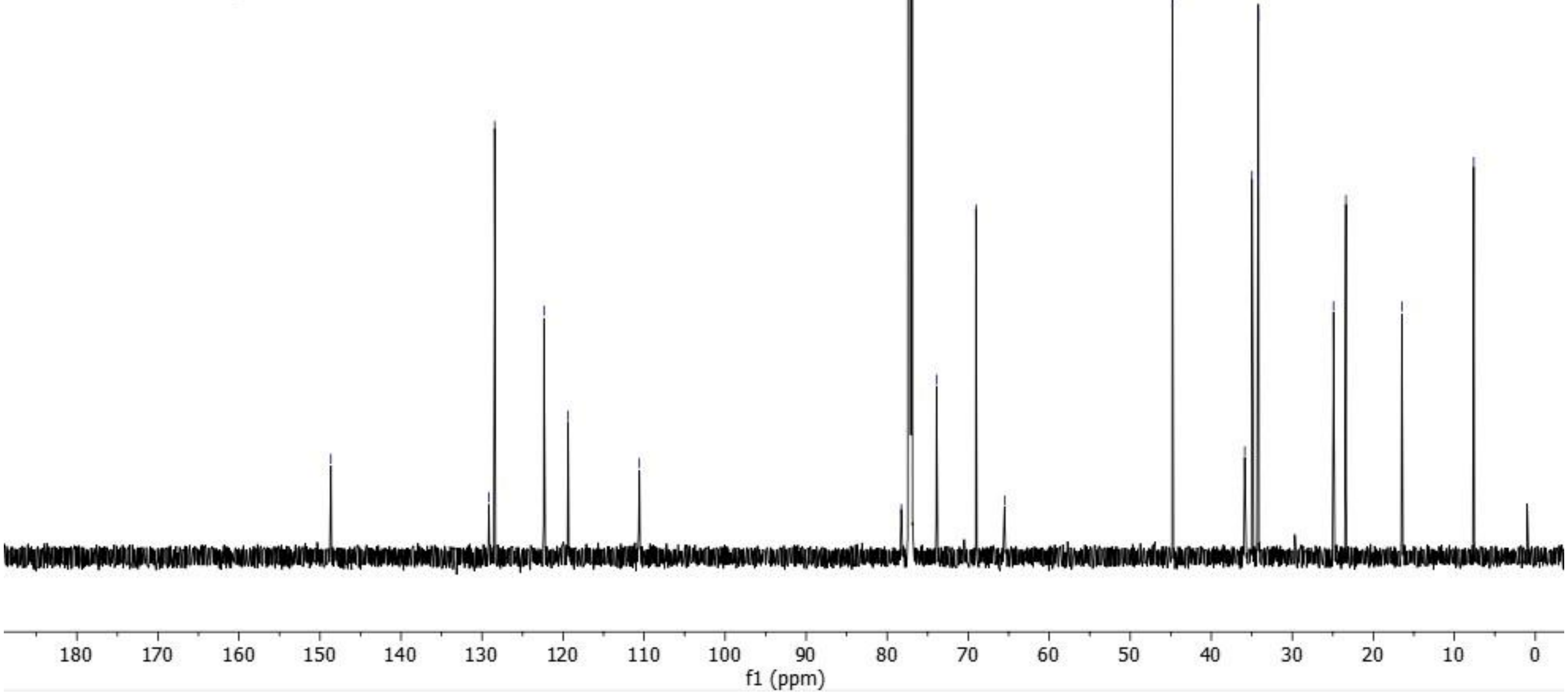

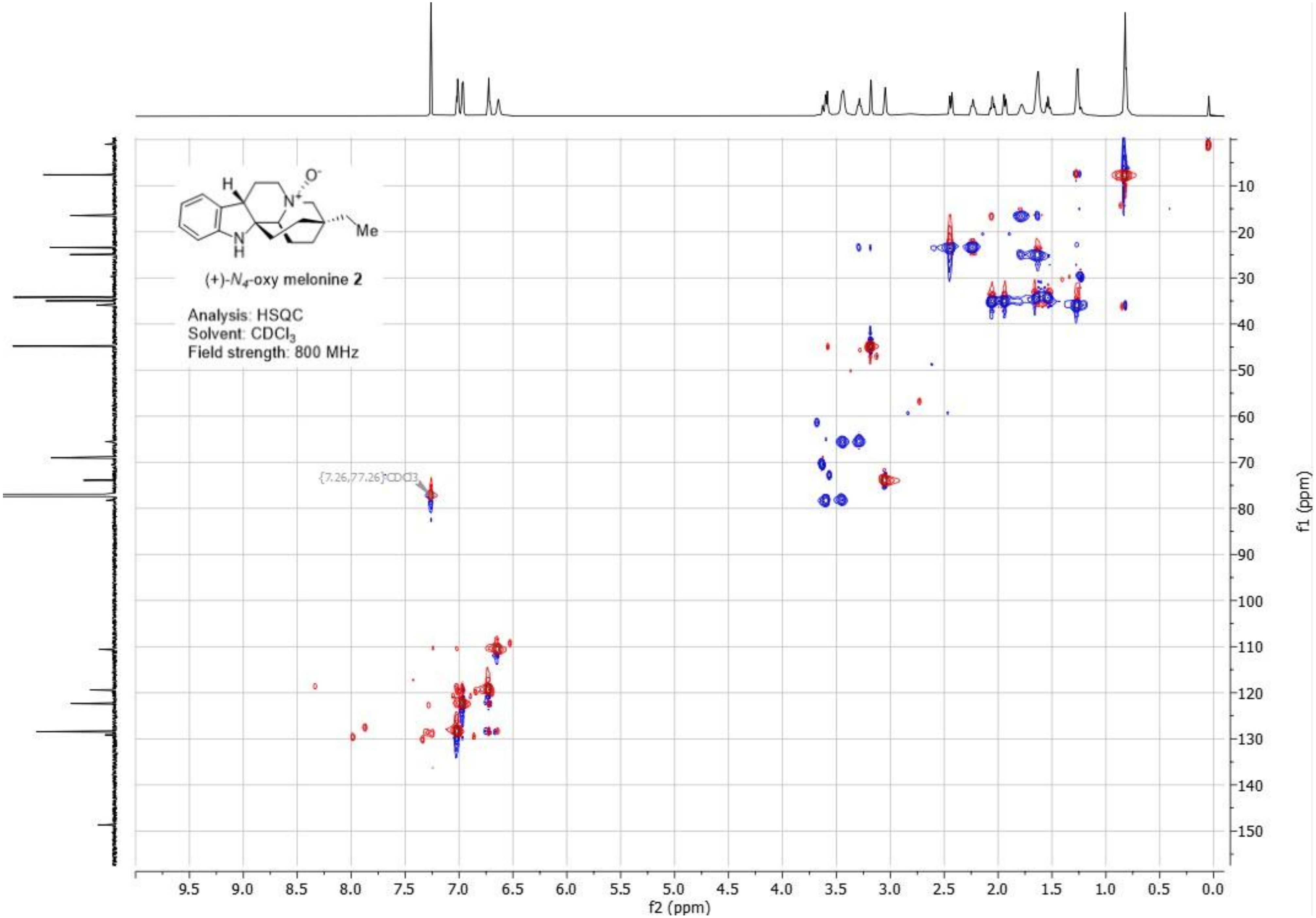
H
N+
O-
Me
N
H
(+)-N4-oxy melonine 2
Analysis: HSQC
Solvent: CDCl3
Field strength: 800 MHz
{7.26,77.26} CDCl3
9.5 9.0 8.5 8.0 7.5 7.0 6.5 6.0 5.5 5.0 4.5 4.0 3.5 3.0 2.5 2.0 1.5 1.0 0.5 0.0
f2 (ppm)
10 20 30 40 50 60 70 80 90 100 110 120 130 140 150
f1 (ppm)

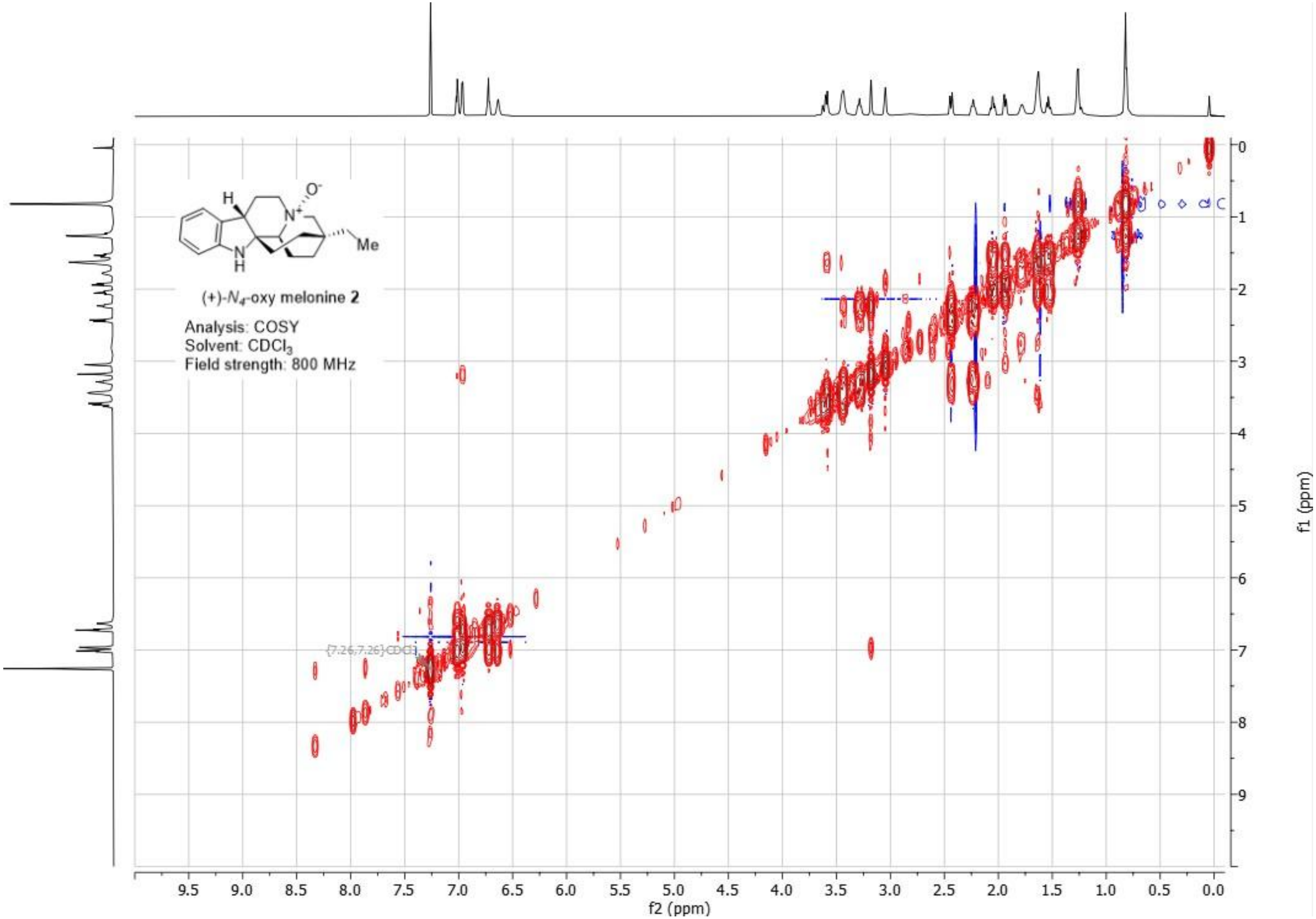

(+)-N_4-oxy melonine **2**
Analysis: COSY
Solvent: $CDCl_3$
Field strength: 800 MHz
f2 (ppm)
f1 (ppm)

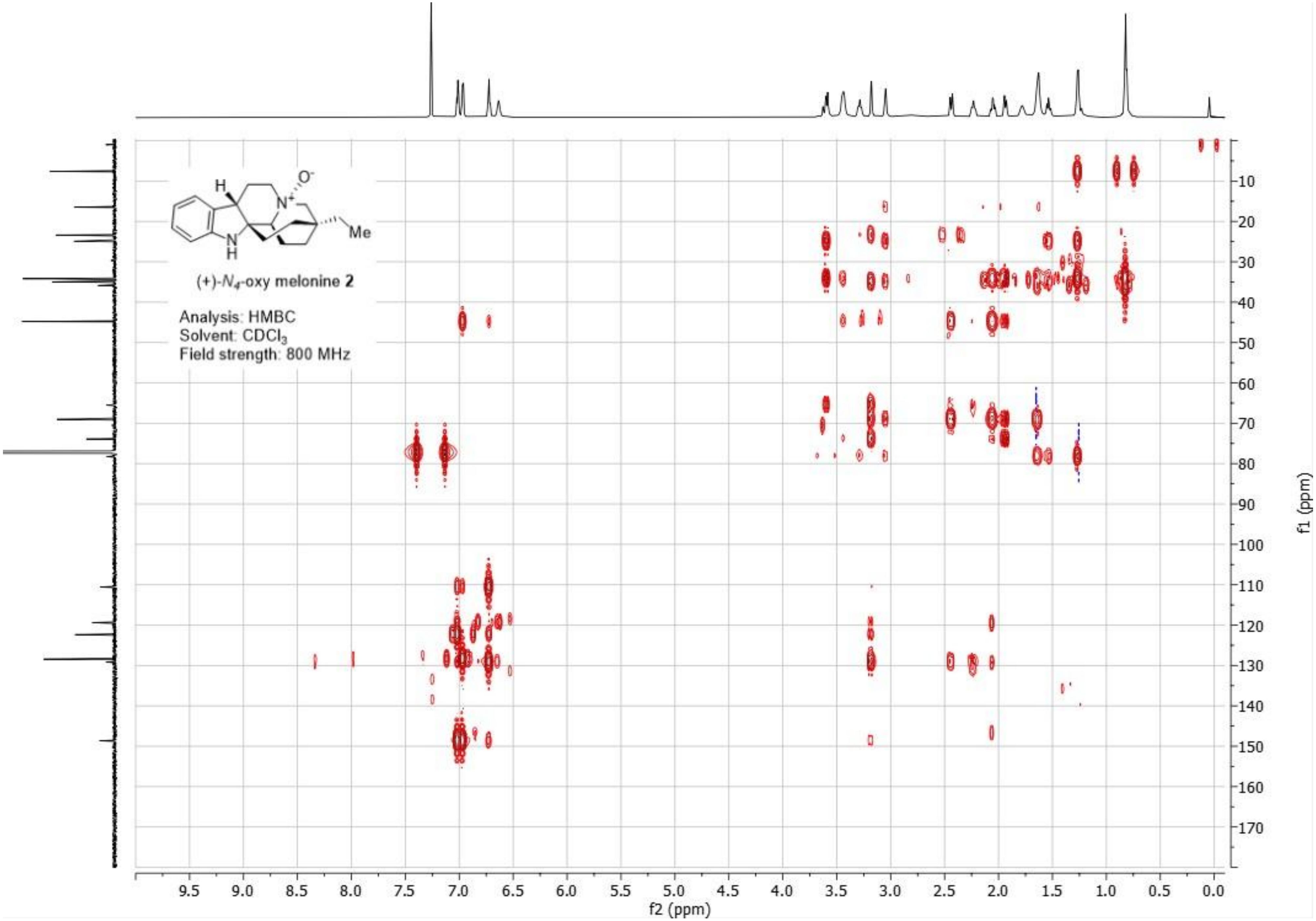

H
N+
O-
Me
N
H
(+)-N4-oxy melonine 2
Analysis: HMBC
Solvent: CDCl3
Field strength: 800 MHz
f1 (ppm)
f2 (ppm)

6) X-Ray structural analysis

X-Ray crystallographic data of **29** (CCDC 2483453)

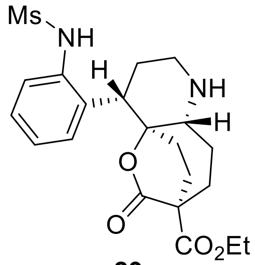

29

R_1=2.68%

Crystal Data and Experimental

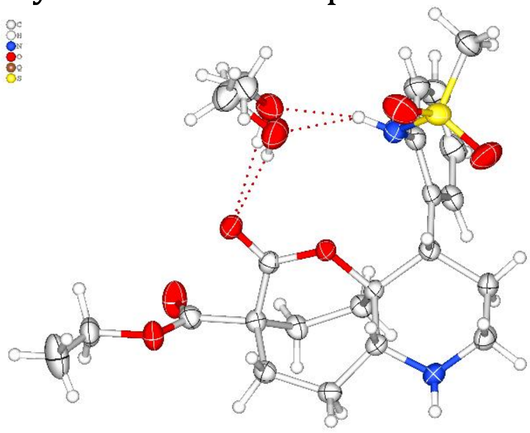

Experimental. Single clear pale colourless plate-shaped crystals of **VG-3-187-SP** were used as supplied. A suitable crystal with dimensions 0.55 × 0.09 × 0.06 mm^3 was selected and mounted on a SuperNova, Dual, Cu at home/near, Atlas diffractometer. The crystal was kept at a steady T = 200.00(10) K during data collection. The structure was solved with the ShelXT (Sheldrick, 2015) solution program using dual methods and by using Olex2 1.5 (Dolomanov et al., 2009) as the graphical interface. The model was refined with ShelXL 2019/3 (Sheldrick, 2015) using full matrix least squares minimisation on F^2.

Crystal Data. $C_{22}H_{32}N_2O_7S$, M_r = 468.55, monoclinic, $P2_1$ (No. 4), a = 10.7621(2) Å, b = 9.1222(2) Å, c = 12.1712(2) Å, β = 106.822(2)°, $\alpha = \gamma$ = 90°, V = 1143.76(4) Å^3, T = 200.00(10) K, Z = 2, Z' = 1, μ(Cu K_α) = 1.651, 10475 reflections measured, 4137 unique (R_{int} = 0.0256) which were used in all calculations. The final wR_2 was 0.0695 (all data) and R_1 was 0.0268 (I≥2 σ(I)).

**Compound**	**VG-3-187-SP**
Formula	$C_{22}H_{32}N_2O_7S$
$D_{calc.}$/ g cm^{-3}	1.361
μ/mm^{-1}	1.651
Formula Weight	468.55
Colour	clear pale colourless
Shape	plate
Size/mm^3	0.55×0.09×0.06
T/K	200.00(10)
Crystal System	monoclinic
Flack Parameter	0.011(9)
Hooft Parameter	0.011(9)
Space Group	$P2_1$
a/Å	10.7621(2)
b/Å	9.1222(2)
c/Å	12.1712(2)
α/°	90
β/°	106.822(2)
γ/°	90
V/Å^3	1143.76(4)
Z	2
Z'	1
Wavelength/Å	1.54184
Radiation type	Cu K_α
Θ_{min}/°	3.794
Θ_{max}/°	72.304
Measured Refl's.	10475
Indep't Refl's	4137
Refl's I≥2 σ(I)	3961
R_{int}	0.0256
Parameters	427
Restraints	38
Largest Peak	0.155
Deepest Hole	-0.212
GooF	1.025
wR_2 (all data)	0.0695
wR_2	0.0676
R_1 (all data)	0.0289
R_1	0.0268

Structure Quality Indicators

**Reflections:**	d min (CuKα) 2Θ=144.6° 0.81	I/σ(I) 35.8	Rint m=2.54 2.56%	Full 135.4° 99% to 144.6° 100	
**Refinement:**	Shift 0.000	Max Peak 0.2	Min Peak -0.2	GooF 1.025	Hooft .011(9)

A clear pale colourless plate-shaped crystal with dimensions 0.55 × 0.09 × 0.06 mm^3 was mounted. Data were collected using a SuperNova, Dual, Cu at home/near, Atlas diffractometer operating at T = 200.00(10) K.

Data were measured using ω scans with Cu K_α radiation. The diffraction pattern was indexed and the total number of runs and images was based on the strategy calculation from the program CrysAlisPro system (CCD 44.112a 64-bit (release 21-05-2025)). The maximum resolution that was achieved was Θ = 72.304° (0.81 Å).

The unit cell was refined using CrysAlisPro 1.171.44.112a (Rigaku OD, 2025) on 6792 reflections, 65% of the observed reflections.

Data reduction, scaling and absorption corrections were performed using CrysAlisPro 1.171.44.112a (Rigaku OD, 2025). The final completeness is 100.00 % out to 72.304° in Θ. A gaussian absorption correction was performed using CrysAlisPro 1.171.44.112a (Rigaku Oxford Diffraction, 2025). The numerical absorption correction was based on gaussian integration over a multifaceted crystal model. The empirical absorption correction was done using spherical harmonics, implemented in SCALE3 ABSPACK scaling algorithm. The absorption coefficient μ of this crystal is 1.651 mm^{-1} at this wavelength (λ = 1.54184Å) and the minimum and maximum transmissions are 0.584 and 1.000.

The structure was solved and the space group $P2_1$ (# 4) determined by the ShelXT (Sheldrick, 2015) structure solution program using dual methods and refined by full matrix least squares minimisation on $\boldsymbol{F^2}$ using version 2019/3 of ShelXL (Sheldrick, 2015). All non-hydrogen atoms were refined anisotropically. Most hydrogen atom positions were calculated geometrically and refined using the riding model, but some hydrogen atoms were refined freely.

There is a single formula unit in the asymmetric unit, which is represented by the reported sum formula. In other words: Z is 2 and Z' is 1. The moiety formula is C21 H28 N2 O6 S, C H4 O.

The Flack parameter was refined to 0.011(9). Determination of absolute structure using Bayesian statistics on Bijvoet differences using the Olex2 results in 0.011(9). The chiral atoms in this structure are: C1(S), C4(R), C5(S), C8(S). Note: The Flack parameter is used to determine chirality of the crystal studied, the value should be near 0, a value of 1 means that the stereochemistry is wrong and the model should be inverted. A value of 0.5 means that the crystal consists of a racemic mixture of the two enantiomers.

Data Plots: Diffraction Data

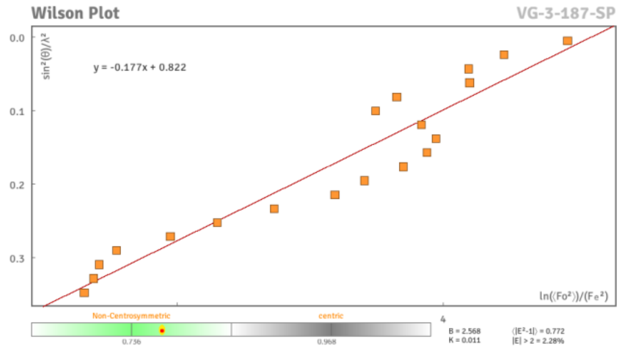

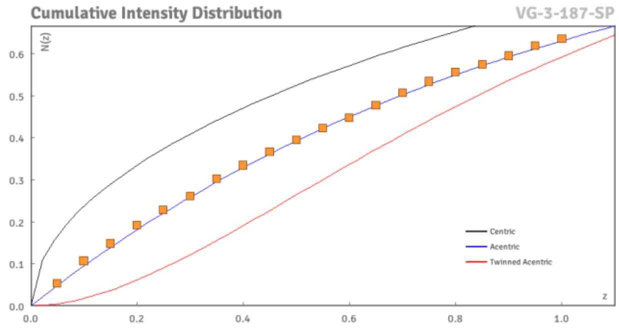

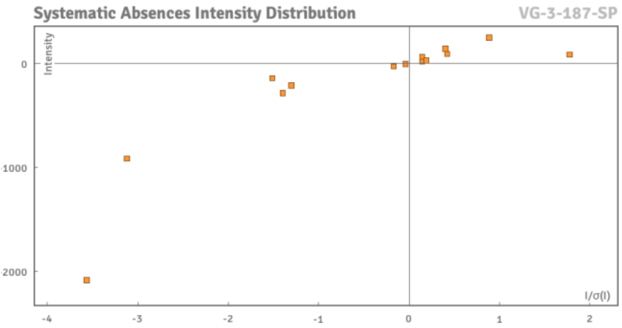

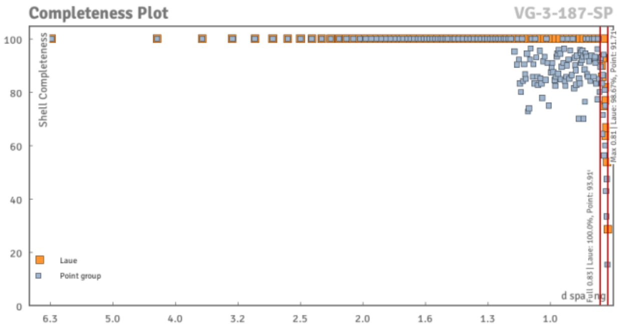

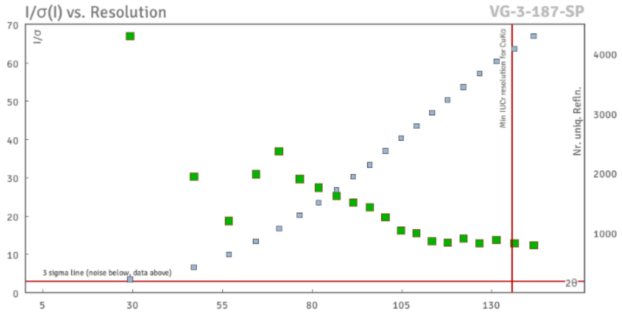

Data Plots: Refinement and Data

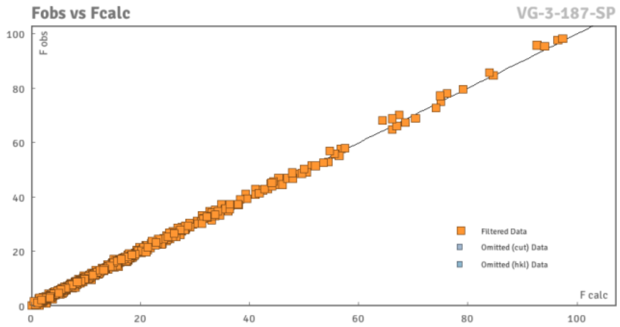

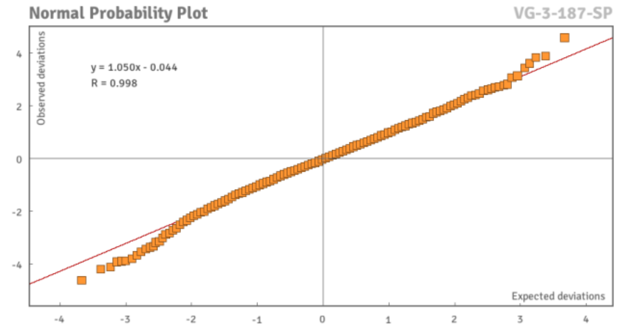

Reflection Statistics

Total reflections (after filtering)	10489	Unique reflections	4137
Completeness	0.917	Mean I/σ	23.49
hkl_{max} collected	(12, 10, 15)	hkl_{min} collected	(-13, -9, -14)
hkl_{max} used	(12, 10, 15)	hkl_{min} used	(-13, -9, 0)
Lim d_{max} collected	100.0	Lim d_{min} collected	0.77
d_{max} used	11.65	d_{min} used	0.81
Friedel pairs	2268	Friedel pairs merged	0
Inconsistent equivalents	0	R_{int}	0.0256
R_{sigma}	0.0279	Intensity transformed	0
Omitted reflections	0	Omitted by user (OMIT hkl)	0
Multiplicity	(3111, 2101, 726, 213, 22, 6)	Maximum multiplicity	10
Removed systematic absences	14	Filtered off (Shel/OMIT)	0

Table S12: Fractional Atomic Coordinates (×10^4) and Equivalent Isotropic Displacement Parameters (Å^2×10^3) for **VG-3-187-SP**. U_{eq} is defined as 1/3 of the trace of the orthogonalised U_{ij}.

Atom	x	y	z	U_{eq}
S1	6248.6(5)	6747.5(7)	1582.7(5)	34.45(15)
O1	7757.9(14)	4255.7(18)	4818.1(12)	26.5(3)
O2	7354.0(17)	5363.8(19)	6273.2(14)	33.3(4)

Atom	x	y	z	U_{eq}
O3	7406(2)	2686(3)	8083.2(16)	48.5(5)
O4	9095.8(17)	4211(2)	8544.0(13)	35.9(4)
O5	6735(2)	8132(2)	2085.9(18)	53.5(5)
O6	7031(2)	5866(3)	1078.4(19)	55.5(6)
N1	10221.3(19)	2142(2)	3892.8(16)	30.6(4)
N2	5937(2)	5807(2)	2600.9(16)	32.7(4)
C1	7656(2)	3230(3)	3030.7(17)	27.3(4)
C2	8287(2)	2278(3)	2303.3(19)	32.8(5)
C3	9744(2)	2517(3)	2676(2)	34.0(5)
C4	9758(2)	3121(3)	4642.8(17)	26.4(4)
C5	8262(2)	2961(2)	4344.6(16)	24.7(4)
C6	7824(2)	1591(3)	4832.6(17)	27.9(4)
C7	8074(2)	1619(3)	6150.4(17)	29.9(4)
C8	8580(2)	3109(3)	6692.5(16)	27.0(4)
C9	7841.8(19)	4324(3)	5929.3(17)	26.4(4)
C10	10497(2)	2677(3)	5864.9(18)	29.7(5)
C11	10044(2)	3338(3)	6835.5(18)	29.3(5)
C12	8278(2)	3292(3)	7842.4(18)	30.7(5)
C13	8863(3)	4496(3)	9647.5(19)	39.8(6)
C14	9897(5)	5486(5)	10302(3)	68.2(11)
C15	6189(2)	3119(3)	2631.4(16)	27.9(4)
C16	5584(2)	1744(3)	2440.8(17)	33.0(4)
C17	4242(2)	1597(4)	2109(2)	40.8(6)
C18	3473(3)	2842(4)	1930(2)	45.6(6)
C19	4040(2)	4220(4)	2071(2)	40.0(6)
C20	5394(2)	4363(3)	2423.3(17)	30.9(5)
C21	4779(3)	7082(4)	518(2)	45.3(7)
O7	5391(2)	6955(3)	4595.4(18)	43.5(7)
C22	5607(7)	8447(7)	4773(4)	51.9(13)
O8	6360(20)	7430(20)	4621(15)	57(6)
C23	5790(60)	8500(50)	5120(40)	54(8)

Table S13: Anisotropic Displacement Parameters (×10[4]) for **VG-3-187-SP**. The anisotropic displacement factor exponent takes the form: $-2\pi^2[h^2a^{*2} \times U_{11} + ... + 2hka^* \times b^* \times U_{12}]$

Atom	U_{11}	U_{22}	U_{33}	U_{23}	U_{13}	U_{12}
S1	32.2(3)	36.1(3)	35.3(3)	6.3(2)	10.13(19)	5.6(2)
O1	31.1(7)	23.9(8)	24.5(6)	0.0(6)	8.3(5)	3.7(6)
O2	37.6(9)	32.2(9)	32.4(7)	-0.6(6)	13.6(7)	5.5(7)
O3	53.7(11)	58.2(14)	41.2(9)	-10.8(9)	25.9(8)	-21.2(10)
O4	40.6(9)	42.1(10)	26.9(7)	-6.2(7)	12.6(6)	-7.3(7)
O5	54.8(11)	39.9(12)	55.6(11)	9.0(9)	0.0(9)	-5.0(9)
O6	58.6(12)	60.4(15)	60.2(12)	18.6(11)	37.2(10)	25.5(11)
N1	29.5(10)	33.0(12)	30.9(9)	-1.9(7)	11.2(7)	4.6(7)
N2	38.3(10)	31.4(11)	28.2(9)	0.0(8)	9.7(8)	6.3(8)
C1	30.2(10)	25.9(12)	25.6(9)	1.9(8)	7.9(8)	1.6(8)
C2	36.8(12)	36.3(14)	26.2(10)	-2.2(9)	10.5(9)	4.3(9)
C3	37.8(12)	36.0(15)	31.9(11)	1.1(9)	16.1(9)	2.8(10)
C4	26.2(10)	24.2(12)	29.7(10)	-1.0(8)	9.4(8)	1.2(8)
C5	26.7(10)	22.3(11)	25.5(9)	-1.9(8)	8.1(7)	1.9(8)
C6	31.1(10)	24.5(12)	27.3(9)	1.1(9)	7.1(8)	-1.4(9)
C7	35.8(11)	26.1(12)	27.6(9)	1.8(9)	9.1(8)	-3.1(10)
C8	28.9(10)	27.0(12)	24.8(9)	-1.3(8)	7.6(7)	-1.6(8)
C9	26.0(10)	26.9(12)	27.4(9)	-2.3(8)	9.3(8)	-1.9(8)
C10	26.4(11)	30.2(14)	31.3(10)	0.8(9)	6.5(8)	1.9(8)
C11	26.0(10)	31.6(13)	28.9(10)	-1.3(9)	5.7(8)	-1.3(9)
C12	32.1(11)	32.3(13)	27.6(10)	0.8(9)	8.4(8)	0.2(9)
C13	50.3(15)	45.1(17)	25.5(10)	-4.3(10)	13.6(10)	0.7(12)
C14	87(3)	81(3)	37.4(15)	-20.9(16)	19.3(16)	-33(2)
C15	32.1(11)	31.7(13)	19.6(8)	0.5(8)	7.1(7)	0.6(9)

Atom	U_{11}	U_{22}	U_{33}	U_{23}	U_{13}	U_{12}
C16	36.9(11)	33.8(13)	26.8(9)	0.5(10)	7.0(8)	-3.7(11)
C17	39.5(12)	47.1(17)	33.7(10)	0.0(11)	7.3(9)	-11.2(12)
C18	31.9(13)	58.3(19)	45.2(13)	-0.1(13)	8.8(10)	-7.4(12)
C19	32.4(12)	50.2(17)	37.8(12)	2.9(11)	10.9(9)	4.8(11)
C20	32.5(11)	35.8(13)	24.7(9)	0.5(9)	8.5(8)	0.8(9)
C21	42.2(14)	61(2)	31.3(11)	8.7(12)	8.2(10)	7.7(13)
O7	50.0(14)	37.8(14)	38.2(10)	-0.5(9)	5.8(9)	4.5(10)
C22	68(3)	37(2)	47(3)	-1.9(19)	11(2)	5.9(19)
O8	76(12)	57(10)	46(8)	8(7)	29(8)	22(9)
C23	65(14)	44(13)	57(15)	-1(13)	25(13)	9(12)

Table S14: Bond Lengths in Å for **VG-3-187-SP**.

Atom	Atom	Length/Å
S1	O5	1.435(2)
S1	O6	1.425(2)
S1	N2	1.620(2)
S1	C21	1.757(3)
O1	C5	1.484(3)
O1	C9	1.331(3)
O2	C9	1.216(3)
O3	C12	1.196(3)
O4	C12	1.331(3)
O4	C13	1.459(3)
N1	C3	1.461(3)
N1	C4	1.464(3)
N2	C20	1.432(3)
C1	C2	1.532(3)
C1	C5	1.562(3)
C1	C15	1.515(3)
C2	C3	1.516(3)
C4	C5	1.552(3)
C4	C10	1.525(3)
C5	C6	1.516(3)
C6	C7	1.548(3)
C7	C8	1.540(3)
C8	C9	1.515(3)
C8	C11	1.549(3)
C8	C12	1.535(3)
C10	C11	1.526(3)
C13	C14	1.475(4)
C15	C16	1.401(4)
C15	C20	1.399(3)
C16	C17	1.389(3)
C17	C18	1.385(5)
C18	C19	1.386(5)
C19	C20	1.401(3)
O7	C22	1.387(7)
O8	C23	1.39(3)

Table S15: Bond Angles in ° for **VG-3-187-SP**.

Atom	Atom	Atom	Angle/°
O5	S1	N2	105.69(12)
O5	S1	C21	107.55(14)
O6	S1	O5	119.49(15)
O6	S1	N2	108.01(12)
O6	S1	C21	107.32(14)
N2	S1	C21	108.37(13)
C9	O1	C5	120.46(17)

Atom	Atom	Atom	Angle/°
C12	O4	C13	116.42(19)
C3	N1	C4	113.96(18)
C20	N2	S1	122.51(15)
C2	C1	C5	112.27(18)
C15	C1	C2	111.82(19)
C15	C1	C5	113.91(17)
C3	C2	C1	109.8(2)
N1	C3	C2	107.42(19)
N1	C4	C5	108.34(17)
N1	C4	C10	105.71(17)
C10	C4	C5	114.59(17)
O1	C5	C1	100.45(16)
O1	C5	C4	107.78(16)
O1	C5	C6	108.32(16)
C4	C5	C1	108.78(16)
C6	C5	C1	116.48(18)
C6	C5	C4	113.88(18)
C5	C6	C7	114.30(19)
C8	C7	C6	113.13(19)
C7	C8	C11	112.94(19)
C9	C8	C7	108.98(17)
C9	C8	C11	107.82(18)
C9	C8	C12	105.52(18)
C12	C8	C7	110.17(18)
C12	C8	C11	111.10(17)
O1	C9	C8	117.28(19)
O2	C9	O1	118.5(2)
O2	C9	C8	124.19(19)
C4	C10	C11	117.19(18)
C10	C11	C8	113.68(17)
O3	C12	O4	123.6(2)
O3	C12	C8	124.2(2)
O4	C12	C8	112.22(19)
O4	C13	C14	107.6(2)
C16	C15	C1	120.2(2)
C20	C15	C1	122.0(2)
C20	C15	C16	117.8(2)
C17	C16	C15	121.9(3)
C18	C17	C16	119.4(3)
C17	C18	C19	120.2(2)
C18	C19	C20	120.3(3)
C15	C20	N2	121.1(2)
C15	C20	C19	120.5(2)
C19	C20	N2	118.4(2)

Table S16: Torsion Angles in ° for **VG-3-187-SP**.

Atom	Atom	Atom	Atom	Angle/°
S1	N2	C20	C15	93.1(2)
S1	N2	C20	C19	-89.0(2)
O1	C5	C6	C7	-48.7(2)
O5	S1	N2	C20	178.14(19)
O6	S1	N2	C20	-52.9(2)
N1	C4	C5	O1	-162.14(16)
N1	C4	C5	C1	-54.1(2)
N1	C4	C5	C6	77.7(2)
N1	C4	C10	C11	-169.76(19)
C1	C2	C3	N1	58.5(3)
C1	C5	C6	C7	-160.89(18)
C1	C15	C16	C17	177.98(19)

Atom	Atom	Atom	Atom	Angle/˚
C1	C15	C20	N2	-1.2(3)
C1	C15	C20	C19	-179.03(19)
C2	C1	C5	O1	165.19(18)
C2	C1	C5	C4	52.2(3)
C2	C1	C5	C6	-78.1(2)
C2	C1	C15	C16	48.8(3)
C2	C1	C15	C20	-130.2(2)
C3	N1	C4	C5	64.0(2)
C3	N1	C4	C10	-172.7(2)
C4	N1	C3	C2	-65.9(3)
C4	C5	C6	C7	71.2(2)
C4	C10	C11	C8	53.6(3)
C5	O1	C9	O2	-175.97(18)
C5	O1	C9	C8	5.8(3)
C5	C1	C2	C3	-54.9(3)
C5	C1	C15	C16	-79.8(2)
C5	C1	C15	C20	101.2(2)
C5	C4	C10	C11	-50.5(3)
C5	C6	C7	C8	6.5(3)
C6	C7	C8	C9	41.2(2)
C6	C7	C8	C11	-78.6(2)
C6	C7	C8	C12	156.56(19)
C7	C8	C9	O1	-50.3(2)
C7	C8	C9	O2	131.6(2)
C7	C8	C11	C10	35.7(3)
C7	C8	C12	O3	-25.4(3)
C7	C8	C12	O4	154.9(2)
C9	O1	C5	C1	167.29(18)
C9	O1	C5	C4	-78.9(2)
C9	O1	C5	C6	44.7(2)
C9	C8	C11	C10	-84.7(2)
C9	C8	C12	O3	92.0(3)
C9	C8	C12	O4	-87.6(2)
C10	C4	C5	O1	80.1(2)
C10	C4	C5	C1	-171.80(19)
C10	C4	C5	C6	-40.1(2)
C11	C8	C9	O1	72.7(2)
C11	C8	C9	O2	-105.5(2)
C11	C8	C12	O3	-151.4(3)
C11	C8	C12	O4	28.9(3)
C12	O4	C13	C14	177.9(3)
C12	C8	C9	O1	-168.55(18)
C12	C8	C9	O2	13.3(3)
C12	C8	C11	C10	160.1(2)
C13	O4	C12	O3	-1.6(4)
C13	O4	C12	C8	178.1(2)
C15	C1	C2	C3	175.6(2)
C15	C1	C5	O1	-66.4(2)
C15	C1	C5	C4	-179.42(19)
C15	C1	C5	C6	50.3(3)
C15	C16	C17	C18	1.7(3)
C16	C15	C20	N2	179.81(18)
C16	C15	C20	C19	2.0(3)
C16	C17	C18	C19	0.6(4)
C17	C18	C19	C20	-1.6(4)
C18	C19	C20	N2	-177.6(2)
C18	C19	C20	C15	0.3(3)
C20	C15	C16	C17	-3.0(3)
C21	S1	N2	C20	63.1(2)

Table S17: Hydrogen Fractional Atomic Coordinates (×10[4]) and Equivalent Isotropic Displacement Parameters (Å^2×10^3) for **VG-3-187-SP**. U_{eq} is defined as 1/3 of the trace of the orthogonalised U_{ij}.

Atom	x	y	z	U_{eq}
H1	11150(40)	2100(40)	4130(30)	51(9)
H2	5740(30)	6390(40)	3100(30)	44(9)
H1A	7910(30)	4200(40)	2890(20)	36(7)
H2A	8100(30)	1210(40)	2420(30)	32(7)
H2B	7900(30)	2580(40)	1500(20)	35(7)
H3A	9940(30)	3480(40)	2510(30)	49(9)
H3B	10240(30)	1920(40)	2280(30)	40(7)
H4	9910(30)	4190(30)	4510(20)	28(6)
H6A	6930(30)	1490(30)	4490(20)	33(7)
H6B	8230(30)	770(40)	4580(30)	47(9)
H7A	7290(30)	1410(30)	6330(30)	39(8)
H7B	8670(40)	890(50)	6500(30)	59(10)
H10A	10460(30)	1600(40)	5910(20)	38(7)
H10B	11400(30)	2960(40)	6000(20)	35(7)
H11A	10560(30)	2890(30)	7600(20)	31(7)
H11B	10220(30)	4370(40)	6870(30)	46(8)
H13A	8880(40)	3430(40)	10040(30)	58(10)
H13B	7980(40)	4960(50)	9510(40)	73(12)
H14A	10700(50)	4980(50)	10340(40)	69(13)
H14B	9760(40)	5670(50)	11040(40)	69(12)
H14C	10070(110)	6490(130)	9900(90)	230(40)
H16	6110(30)	850(40)	2520(30)	37(8)
H17	3810(40)	560(50)	2010(30)	54(10)
H18	2490(40)	2790(50)	1700(30)	69(11)
H19	3560(30)	5050(40)	1970(30)	47(9)
H21A	4220(30)	7700(40)	870(30)	55(9)
H21B	4960(30)	7670(40)	-70(30)	47(8)
H21C	4400(40)	6160(50)	200(40)	69(12)
H7	5920(40)	6490(50)	5070(40)	51(11)
H22A	5141.79	8981.99	4077.47	78
H22B	6538.86	8647.98	4952.6	78
H22C	5293.19	8764.51	5414.62	78
H8	6880.29	6933.66	5136.16	86
H23A	5782.98	9430.53	4725.29	81
H23B	6279.04	8602.49	5932.69	81
H23C	4892.87	8207.13	5063.64	81

Table S18: Atomic Occupancies for all atoms that are not fully occupied in **VG-3-187-SP**.

Atom	Occupancy
O7	0.883(7)
H7	0.883(7)
C22	0.883(7)
H22A	0.883(7)
H22B	0.883(7)
H22C	0.883(7)
O8	0.117(7)
H8	0.117(7)
C23	0.117(7)
H23A	0.117(7)
H23B	0.117(7)
H23C	0.117(7)

Citations

CrysAlisPro Software System, Rigaku Oxford Diffraction, (2025).
O.V. Dolomanov and L.J. Bourhis and R.J. Gildea and J.A.K. Howard and H. Puschmann, Olex2: A complete structure solution, refinement and analysis program, *J. Appl. Cryst.*, (2009), **42**, 339-341.
Sheldrick, G.M., Crystal structure refinement with ShelXL, *Acta Cryst.*, (2015), **C71**, 3-8.
Sheldrick, G.M., ShelXT-Integrated space-group and crystal-structure determination, *Acta Cryst.*, (2015), **A71**, 3-8.

X-Ray crystallographic data of **32** (CCDC 2483452)

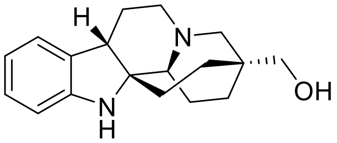

32

R_1=4.20%

**Compound**	**VG-3-194-A**
Formula	$C_{19}H_{28}N_2O_2$
$D_{calc.}$/ g cm^{-3}	1.270
μ/mm^{-1}	0.648
Formula Weight	316.43
Colour	clear light yellow
Shape	irregular
Size/mm^3	0.25×0.17×0.07
T/K	200.00(10)
Crystal System	orthorhombic
Flack Parameter	0.4(2)
Hooft Parameter	0.3(3)
Space Group	$P2_12_12_1$
a/Å	8.4224(2)
b/Å	9.8335(3)
c/Å	19.9861(5)
α/°	90
β/°	90
γ/°	90
V/Å^3	1655.28(8)
Z	4
Z'	1
Wavelength/Å	1.54184
Radiation type	Cu K_α
Θ_{min}/°	4.425
Θ_{max}/°	72.528
Measured Refl's.	3411
Indep't Refl's	3411
Refl's I≥2 σ(I)	2828
R_{int}	.
Parameters	224
Restraints	0
Largest Peak	0.244
Deepest Hole	-0.169
GooF	0.968
wR_2 (all data)	0.1042
wR_2	0.1009
R_1 (all data)	0.0517
R_1	0.0420

Crystal Data and Experimental

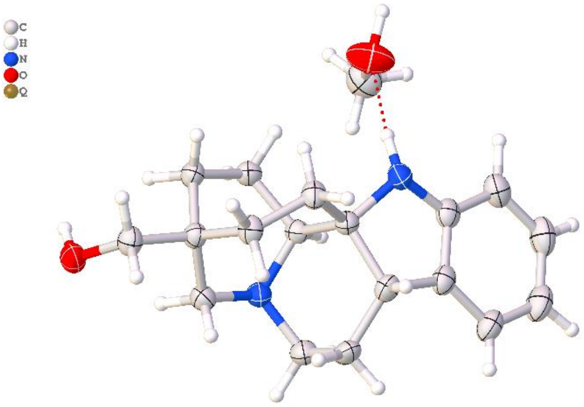

Experimental. Single clear light yellow irregular-shaped crystals of **VG-3-194-A** were used as supplied. A suitable crystal with dimensions 0.25 × 0.17 × 0.07 mm^3 was selected and mounted on a SuperNova, Dual, Cu at home/near, Atlas diffractometer. The crystal was kept at a steady T = 200.00(10) K during data collection. The structure was solved with the ShelXT (Sheldrick, 2015) solution program using dual methods and by using Olex2 1.5 (Dolomanov et al., 2009) as the graphical interface. The model was refined with ShelXL 2019/3 (Sheldrick, 2015) using full matrix least squares minimisation on F^2.

Crystal Data. $C_{19}H_{28}N_2O_2$, M_r = 316.43, orthorhombic, $P2_12_12_1$ (No. 19), a = 8.4224(2) Å, b = 9.8335(3) Å, c = 19.9861(5) Å, $\alpha = \beta = \gamma$ = 90°, V = 1655.28(8) Å^3, T = 200.00(10) K, Z = 4, Z' = 1, μ(Cu K_α) = 0.648, 3411 reflections measured, 3411 unique (R_{int} = .) which were used in all calculations. The final wR_2 was 0.1042 (all data) and R_1 was 0.0420 (I≥2 σ(I)).

Structure Quality Indicators

**Reflections:**	d min (CuKα) 2Θ=145.1° 0.81	I/σ(I) 13.6	Rint m=1.16 18.63%	Full 135.4° 98% to 145.1° 99.9
**Refinement:**	Shift 0.000	Max Peak 0.2	Min Peak -0.2	GooF 0.968
				Hooft .3(3)

A clear light yellow irregular-shaped crystal with dimensions 0.25 × 0.17 × 0.07 mm^3 was mounted. Data were collected using a SuperNova, Dual, Cu at home/near, Atlas diffractometer operating at T = 200.00(10) K.

Data were measured using ω scans with Cu K_α radiation. The diffraction pattern was indexed and the total number of runs and images was based on the strategy calculation from the program CrysAlisPro system (CCD 44.113a 64-bit (release 02-06-2025)). The maximum resolution that was achieved was Θ = 72.528° (0.81 Å).

The unit cell was refined using CrysAlisPro 1.171.44.113a (Rigaku OD, 2025) on 3157 reflections, 93% of the observed reflections.

Data reduction, scaling and absorption corrections were performed using CrysAlisPro 1.171.44.113a (Rigaku OD, 2025). The final completeness is 99.70 % out to 72.528° in Θ. A gaussian absorption correction was performed using CrysAlisPro 1.171.44.113a (Rigaku Oxford Diffraction, 2025). The numerical absorption correction was based on gaussian integration over a multifaceted crystal model. The empirical absorption correction was done using spherical harmonics, implemented in SCALE3 ABSPACK scaling algorithm. The absorption coefficient μ of this crystal is 0.648 mm^{-1} at this wavelength (λ = 1.54184Å) and the minimum and maximum transmissions are 0.707 and 1.000.

The structure was solved and the space group $P2_12_12_1$ (# 19) determined by the ShelXT (Sheldrick, 2015) structure solution program using dual methods and refined by full matrix least squares minimisation on $\boldsymbol{F^2}$ using version 2019/3 of ShelXL (Sheldrick, 2015). All non-hydrogen atoms were refined anisotropically. Most hydrogen atom positions were calculated geometrically and refined using the riding model, but some hydrogen atoms were refined freely.

_refine_special_details: Refined as a 3-component twin.

_twin_special_details: Component 2 rotated by 16.7017° around [-0.89 -0.26 0.37] (reciprocal) or [-0.98 -0.21 0.07] (direct)Component 3 rotated by -178.3109° around [0.00 0.01 1.00] (reciprocal) or [0.01 0.05 1.00] (direct)

There is a single formula unit in the asymmetric unit, which is represented by the reported sum formula. In other words: Z is 4 and Z' is 1. The moiety formula is C18 H24 N2 O, C H4 O.

The Flack parameter was refined to 0.4(2). Determination of absolute structure using Bayesian statistics on Bijvoet differences using the Olex2 results in 0.3(3). The chiral atoms in this structure are: C1(S), C3(S), C8(S), C15(R). Note: The Flack parameter is used to determine chirality of the crystal studied, the value should be near 0, a value of 1 means that the stereochemistry is wrong and the model should be inverted. A value of 0.5 means that the crystal consists of a racemic mixture of the two enantiomers.

Data Plots: Diffraction Data

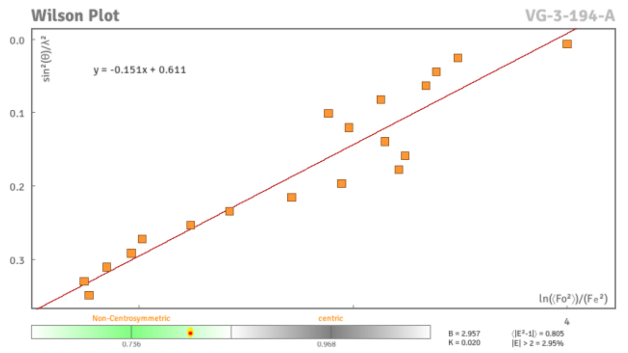

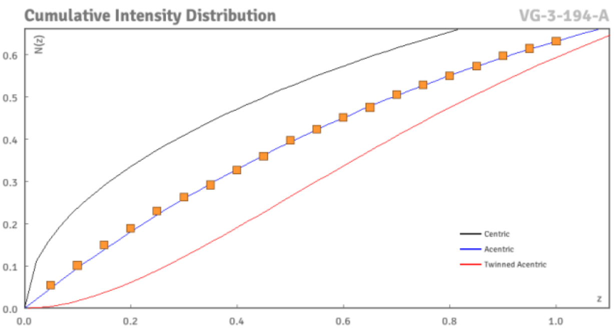

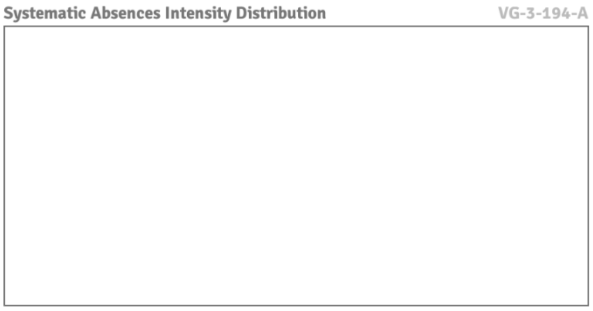

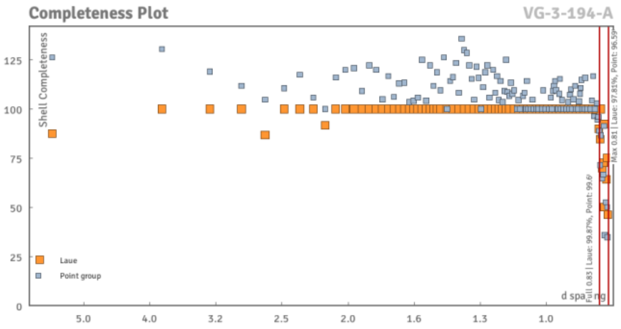

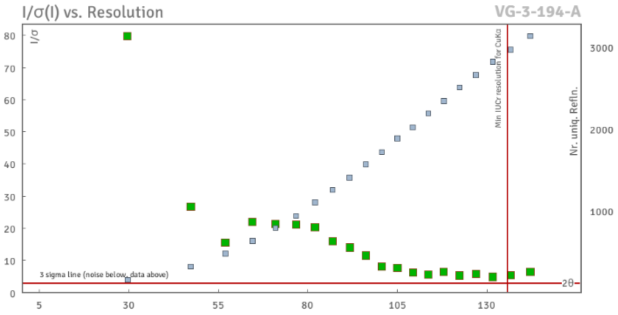

Data Plots: Refinement and Data

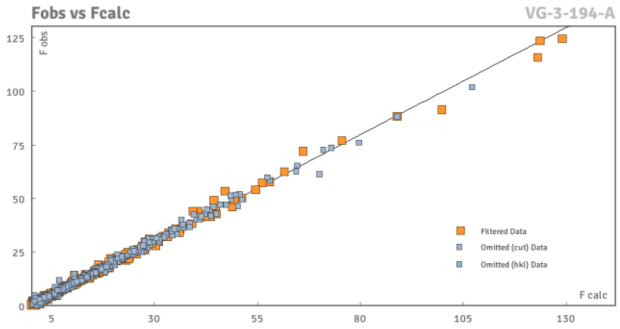

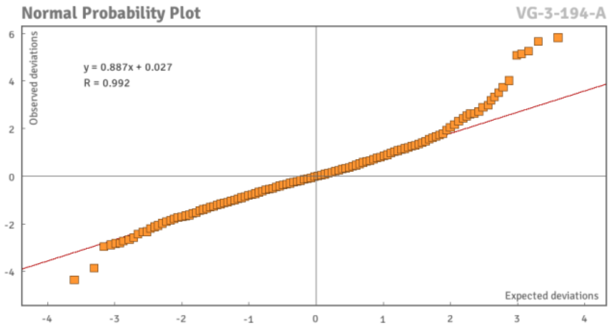

Reflection Statistics

Total reflections (after filtering)	3682	Unique reflections	3176
Completeness	0.966	Mean I/σ	14.48
hkl$_{max}$ collected	(10, 11, 24)	hkl$_{min}$ collected	(-8, -12, -17)
hkl$_{max}$ used	(10, 12, 24)	hkl$_{min}$ used	(-10, 0, 0)
Lim d$_{max}$ collected	100.0	Lim d$_{min}$ collected	0.77
d$_{max}$ used	9.99	d$_{min}$ used	0.81
Friedel pairs	90	Friedel pairs merged	0
Inconsistent equivalents	95	R$_{int}$	0.1863
R$_{sigma}$	0.0735	Intensity transformed	0
Omitted reflections	0	Omitted by user (OMIT hkl)	0
Multiplicity	(3373, 19)	Maximum multiplicity	4
Removed systematic absences	0	Filtered off (Shel/OMIT)	0

Table S19: Fractional Atomic Coordinates (×10^4) and Equivalent Isotropic Displacement Parameters (Å^2×10^3) for **VG-3-194-A.** U_{eq} is defined as 1/3 of the trace of the orthogonalised U_{ij}.

Atom	x	y	z	U_{eq}
O1	1808(3)	9205(2)	4439.6(12)	38.5(5)
O2	3569(4)	1499(3)	4389.0(14)	65.9(8)
N1	5802(3)	6568(3)	4284.4(12)	29.6(5)
N2	5223(3)	3204(2)	3402.1(13)	32.0(5)
C1	3046(3)	7185(3)	3886.6(14)	27.9(6)
C2	4657(4)	7676(3)	4142.5(14)	31.5(6)

Atom	x	y	z	U_{eq}
C3	5205(3)	5173(3)	4168.7(13)	26.9(6)
C4	3626(3)	5002(3)	4540.5(14)	30.4(6)
C5	2392(3)	6106(3)	4363.1(14)	30.9(6)
C6	7361(3)	6767(3)	3965.1(15)	34.9(7)
C7	7321(4)	6532(3)	3214.5(16)	36.5(7)
C8	6667(3)	5123(3)	3043.2(14)	31.2(6)
C9	7751(4)	3934(3)	3194.1(14)	32.9(6)
C10	9380(4)	3781(4)	3114.8(16)	39.5(8)
C11	10064(4)	2522(4)	3275.2(18)	46.8(8)
C12	9131(4)	1454(4)	3494.4(18)	46.6(8)
C13	7500(4)	1594(4)	3564.4(15)	40.6(7)
C14	6822(4)	2848(3)	3407.2(14)	32.7(6)
C15	5123(3)	4709(3)	3421.8(14)	28.1(6)
C16	3591(3)	5151(3)	3085.5(15)	32.4(6)
C17	3151(4)	6647(3)	3163.1(14)	31.8(6)
C18	1890(4)	8403(3)	3856.5(15)	34.8(6)
C19	4524(5)	1501(4)	4961(2)	54.3(9)

Table S20: Anisotropic Displacement Parameters (×10[4]) for **VG-3-194-A**. The anisotropic displacement factor exponent takes the form: $-2\pi^2[h^2a^{*2} \times U_{11} + ... + 2hka^* \times b^* \times U_{12}]$

Atom	U_{11}	U_{22}	U_{33}	U_{23}	U_{13}	U_{12}
O1	46.9(13)	29.6(11)	39.1(12)	0.7(9)	10.5(11)	3.2(9)
O2	77(2)	55.6(17)	64.8(17)	19.5(14)	-12.9(15)	-25.5(15)
N1	25.7(11)	29.5(12)	33.5(12)	-3.7(10)	-0.6(10)	-1.0(10)
N2	31.2(12)	28.5(12)	36.2(13)	-4.7(10)	0.1(11)	0.3(10)
C1	26.9(15)	27.0(13)	29.7(13)	0.2(11)	1.3(12)	3.0(11)
C2	33.7(15)	27.4(14)	33.4(14)	-0.7(11)	0.2(13)	0.3(12)
C3	24.9(13)	24.2(13)	31.6(14)	1.2(10)	-1.2(11)	1.0(11)
C4	29.6(15)	29.1(15)	32.5(14)	1.1(12)	1.0(11)	-1.8(11)
C5	26.9(14)	31.0(15)	34.7(15)	-1.2(11)	4.0(12)	2.4(11)
C6	27.4(14)	35.1(15)	42.3(17)	-4.6(13)	0.9(13)	-3.5(12)
C7	30.9(15)	34.5(16)	43.9(16)	3.4(14)	9.5(13)	-3.5(12)
C8	28.0(14)	37.0(16)	28.5(14)	-0.6(12)	3.0(12)	1.9(12)
C9	31.5(15)	40.3(17)	27.1(13)	-6.1(12)	0.5(12)	3.7(12)
C10	28.7(16)	52(2)	37.7(16)	-6.2(14)	2.3(12)	5.2(13)
C11	30.9(17)	62(2)	47.4(18)	-12.0(16)	-3.2(15)	12.8(15)
C12	48.6(19)	46(2)	45.1(18)	-7.9(16)	-6.0(15)	20.0(16)
C13	49.1(19)	35.5(16)	37.0(16)	-3.7(13)	-1.1(14)	7.7(14)
C14	33.7(15)	37.3(15)	26.9(13)	-6.9(11)	-2.1(13)	4.2(12)
C15	26.4(14)	27.8(13)	30.0(13)	-2.1(11)	-0.2(11)	-0.1(11)
C16	29.2(14)	35.7(16)	32.3(15)	-6.9(12)	-5.0(12)	3.4(12)
C17	31.9(14)	34.2(15)	29.4(13)	0.8(12)	-2.9(12)	5.8(12)
C18	35.3(16)	34.3(15)	34.7(15)	0.0(12)	1.6(13)	6.2(13)
C19	60(2)	49(2)	54(2)	7.1(17)	-1.8(18)	2.0(18)

Table S21: Bond Lengths in Å for **VG-3-194-A**.

Atom	Atom	Length/Å
O1	C18	1.409(4)
O2	C19	1.398(5)
N1	C2	1.483(4)
N1	C3	1.479(4)
N1	C6	1.473(4)
N2	C14	1.391(4)
N2	C15	1.483(4)
C1	C2	1.528(4)
C1	C5	1.529(4)
C1	C17	1.543(4)
C1	C18	1.544(4)
C3	C4	1.532(4)
C3	C15	1.563(4)

Atom	Atom	Length/Å
C4	C5	1.544(4)
C6	C7	1.518(4)
C7	C8	1.529(4)
C8	C9	1.514(4)
C8	C15	1.559(4)
C9	C10	1.390(4)
C9	C14	1.390(4)
C10	C11	1.403(5)
C11	C12	1.383(6)
C12	C13	1.388(5)
C13	C14	1.395(4)
C15	C16	1.519(4)
C16	C17	1.525(4)

Table S22: Bond Angles in ° for **VG-3-194-A**.

Atom	Atom	Atom	Angle/°
C3	N1	C2	115.5(2)
C6	N1	C2	113.5(2)
C6	N1	C3	111.0(2)
C14	N2	C15	107.8(2)
C2	C1	C5	109.3(2)
C2	C1	C17	111.8(2)
C2	C1	C18	109.2(2)
C5	C1	C17	111.5(2)
C5	C1	C18	109.6(2)
C17	C1	C18	105.4(2)
N1	C2	C1	114.2(2)
N1	C3	C4	108.7(2)
N1	C3	C15	115.8(2)
C4	C3	C15	113.1(2)
C3	C4	C5	113.3(2)
C1	C5	C4	112.9(2)
N1	C6	C7	112.8(2)
C6	C7	C8	111.5(2)
C7	C8	C15	115.4(2)
C9	C8	C7	116.0(3)
C9	C8	C15	101.8(2)
C10	C9	C8	131.0(3)
C10	C9	C14	120.5(3)
C14	C9	C8	108.3(2)
C9	C10	C11	118.4(3)
C12	C11	C10	120.7(3)
C11	C12	C13	121.2(3)
C12	C13	C14	118.1(3)
N2	C14	C13	128.3(3)
C9	C14	N2	110.5(3)
C9	C14	C13	121.1(3)
N2	C15	C3	108.3(2)
N2	C15	C8	101.6(2)
N2	C15	C16	108.8(2)
C8	C15	C3	110.5(2)
C16	C15	C3	112.1(2)
C16	C15	C8	114.8(2)
C15	C16	C17	115.9(2)
C16	C17	C1	116.1(2)
O1	C18	C1	115.6(2)

Table S23: Torsion Angles in ° for **VG-3-194-A**.

Atom	Atom	Atom	Atom	Angle/°
N1	C3	C4	C5	53.8(3)

Atom	Atom	Atom	Atom	Angle/˚
N1	C3	C15	N2	154.8(2)
N1	C3	C15	C8	44.4(3)
N1	C3	C15	C16	-85.1(3)
N1	C6	C7	C8	-55.4(3)
N2	C15	C16	C17	170.0(2)
C2	N1	C3	C4	-51.8(3)
C2	N1	C3	C15	76.9(3)
C2	N1	C6	C7	-73.1(3)
C2	C1	C5	C4	-48.1(3)
C2	C1	C17	C16	87.2(3)
C2	C1	C18	O1	-49.6(3)
C3	N1	C2	C1	-1.0(3)
C3	N1	C6	C7	59.0(3)
C3	C4	C5	C1	-3.6(4)
C3	C15	C16	C17	50.2(3)
C4	C3	C15	N2	-78.7(3)
C4	C3	C15	C8	170.8(2)
C4	C3	C15	C16	41.4(3)
C5	C1	C2	N1	51.9(3)
C5	C1	C17	C16	-35.4(3)
C5	C1	C18	O1	70.1(3)
C6	N1	C2	C1	128.9(2)
C6	N1	C3	C4	177.2(2)
C6	N1	C3	C15	-54.1(3)
C6	C7	C8	C9	-72.4(3)
C6	C7	C8	C15	46.5(3)
C7	C8	C9	C10	-40.0(4)
C7	C8	C9	C14	144.1(3)
C7	C8	C15	N2	-155.2(3)
C7	C8	C15	C3	-40.4(3)
C7	C8	C15	C16	87.6(3)
C8	C9	C10	C11	-177.4(3)
C8	C9	C14	N2	0.8(3)
C8	C9	C14	C13	178.2(3)
C8	C15	C16	C17	-77.0(3)
C9	C8	C15	N2	-28.7(3)
C9	C8	C15	C3	86.1(3)
C9	C8	C15	C16	-145.9(2)
C9	C10	C11	C12	1.2(5)
C10	C9	C14	N2	-175.5(3)
C10	C9	C14	C13	1.8(4)
C10	C11	C12	C13	-0.2(5)
C11	C12	C13	C14	0.0(5)
C12	C13	C14	N2	176.0(3)
C12	C13	C14	C9	-0.7(4)
C14	N2	C15	C3	-85.4(3)
C14	N2	C15	C8	31.0(3)
C14	N2	C15	C16	152.5(2)
C14	C9	C10	C11	-2.0(5)
C15	N2	C14	C9	-21.0(3)
C15	N2	C14	C13	161.9(3)
C15	C3	C4	C5	-76.4(3)
C15	C8	C9	C10	-166.1(3)
C15	C8	C9	C14	18.1(3)
C15	C16	C17	C1	-55.2(4)
C17	C1	C2	N1	-72.0(3)
C17	C1	C5	C4	76.0(3)
C17	C1	C18	O1	-169.8(2)
C18	C1	C2	N1	171.8(2)
C18	C1	C5	C4	-167.7(2)
C18	C1	C17	C16	-154.3(3)

Table S24: Hydrogen Fractional Atomic Coordinates (×10[4]) and Equivalent Isotropic Displacement Parameters (Å^2×10^3) for **VG-3-194-A**. U_{eq} is defined as 1/3 of the trace of the orthogonalised U_{ij}.

Atom	x	y	z	U_{eq}
H1	1510(50)	8740(50)	4770(20)	54(12)
H2C	2970(50)	640(50)	4440(20)	57(12)
H2	4530(50)	2700(40)	3682(18)	42(10)
H2A	5127.07	8293.43	3804.98	38
H2B	4490.07	8208.46	4556.99	38
H3	5975.34	4548.64	4393.36	32
H4A	3830.2	5028.71	5028.14	36
H4B	3177.51	4097.92	4432.45	36
H5A	1458.15	5666.92	4154	37
H5B	2032.21	6553.36	4780.04	37
H6A	8136.75	6134.24	4169.12	42
H6B	7729.51	7705.9	4053.69	42
H7A	8408.03	6620.94	3031.46	44
H7B	6648.3	7234.96	3001.67	44
H8	6429.26	5107.9	2553.08	37
H10	10015.23	4510.96	2955.77	47
H11	11178.48	2401.83	3232.88	56
H12	9616.67	607.96	3599.05	56
H13	6864.02	857.15	3714.91	49
H16A	2712.06	4594.73	3268.17	39
H16B	3672.93	4944.61	2601.92	39
H17A	2111.65	6796.81	2943.73	38
H17B	3946.35	7197.73	2918.93	38
H18A	2206.7	8994.21	3478.7	42
H18B	813.11	8051.65	3757.72	42
H19A	5642.71	1443.77	4829.37	65
H19B	4252.13	718.72	5242.27	65
H19C	4346.68	2343.04	5212.91	65
H19D	3851.64	1559.91	5360.33	65
H19E	5242.22	2284.97	4947.43	65
H19F	5147.66	660.65	4976.79	65

Table S25: Hydrogen Bond information for **VG-3-194-A**.

D	H	A	d(D-H)/Å	d(H-A)/Å	d(D-A)/Å	D-H-A/deg
O1	H1	N1[1]	0.84(5)	2.01(4)	2.792(3)	155(4)
O2	H2C	O1[2]	0.99(5)	1.72(5)	2.702(4)	173(4)
N2	H2	O2	0.95(4)	2.01(4)	2.939(4)	166(3)
C4	H4B	O2	0.99	2.58	3.458(4)	148.1

----

[1]-1/2+x,3/2-y,1-z; [2]+x,-1+y,+z

Table S26: Atomic Occupancies for all atoms that are not fully occupied in **VG-3-194-A**.

Atom	Occupancy
H19A	0.26(4)
H19B	0.26(4)
H19C	0.26(4)
H19D	0.74(4)
H19E	0.74(4)
H19F	0.74(4)

Citations

CrysAlisPro Software System, Rigaku Oxford Diffraction, (2025).

O.V. Dolomanov and L.J. Bourhis and R.J. Gildea and J.A.K. Howard and H. Puschmann, Olex2: A complete structure solution, refinement and analysis program, *J. Appl. Cryst.*, (2009), **42**, 339-341.

Sheldrick, G.M., Crystal structure refinement with ShelXL, *Acta Cryst.*, (2015), **C71**, 3-8.

Sheldrick, G.M., ShelXT-Integrated space-group and crystal-structure determination, *Acta Cryst.*, (2015), **A71**, 3-8.

X-Ray crystallographic data of **1·TFA** (CCDC 2488639)

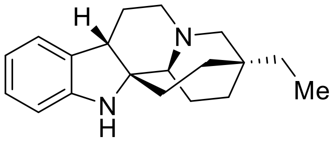

1·TFA

Crystal Data and Experimental

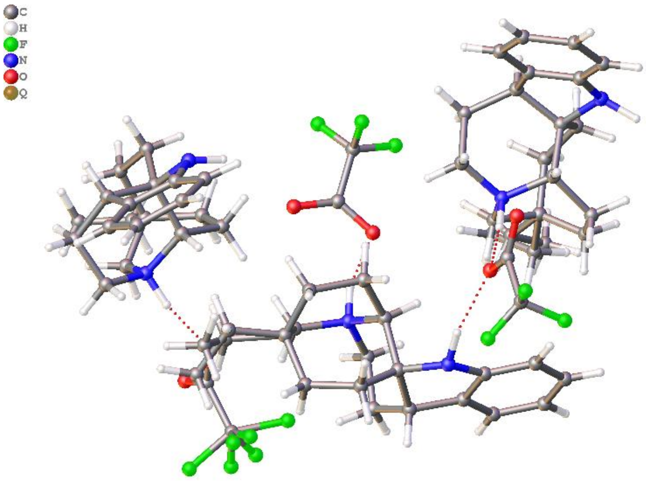

Experimental. Single clear pale colourless plate-shaped crystals of **VG-3-214-2-A2** were used as supplied. A suitable crystal with dimensions 0.20 × 0.16 × 0.13 mm³ was selected and mounted on a XtaLAB Synergy R, DW system, HyPix-Arc 150 diffractometer. The crystal was kept at a steady T = 139.99(10) K during data collection. The structure was solved with the ShelXT (Sheldrick, 2015) solution program using dual methods and by using Olex2 1.5 (Dolomanov et al., 2009) as the graphical interface. The model was refined with ShelXL 2019/3 (Sheldrick, 2015) using full matrix least squares minimisation on $\boldsymbol{F}^2$.

Crystal Data. $C_{21}H_{27}F_3N_2O_2$, M_r = 396.44, orthorhombic, $P2_12_12_1$ (No. 19), a = 8.9214(2) Å, b = 20.3439(3) Å, c = 32.7071(5) Å, $\alpha = \beta = \gamma = 90°$, V = 5936.21(18) Å³, T = 139.99(10) K, Z = 12, Z' = 3, μ(Cu K$_\alpha$) = 0.880, 115407 reflections measured, 11869 unique (R_{int} = 0.0662) which were used in all calculations. The final wR_2 was 0.1753 (all data) and R_1 was 0.0589 (I≥2σ(I)).

R_1=5.89%

**Compound**	**VG-3-214-2-A2**
Formula	$C_{21}H_{27}F_3N_2O_2$
$D_{calc.}$/ g cm^{-3}	1.331
μ/mm^{-1}	0.880
Formula Weight	396.44
Colour	clear pale colourless
Shape	plate-shaped
Size/mm³	0.20×0.16×0.13
T/K	139.99(10)
Crystal System	orthorhombic
Flack Parameter	0.1(2)
Hooft Parameter	0.03(4)
Space Group	$P2_12_12_1$
a/Å	8.9214(2)
b/Å	20.3439(3)
c/Å	32.7071(5)
α/°	90
β/°	90
γ/°	90
V/Å³	5936.21(18)
Z	12
Z'	3
Wavelength/Å	1.54184
Radiation type	Cu K$_\alpha$
Θ_{min}/°	2.558
Θ_{max}/°	74.278
Measured Refl's.	115407
Indep't Refl's	11869
Refl's I≥2σ(I)	7793
R_{int}	0.0662
Parameters	850
Restraints	166
Largest Peak/eÅ³	0.408
Deepest Hole/eÅ³	-0.282
GooF	1.026
wR_2 (all data)	0.1753
wR_2	0.1478
R_1 (all data)	0.1004
R_1	0.0589

Structure Quality Indicators

**Reflections:**	d min (CuKα) 2Θ=148.6° 0.80	I/σ(I) 26.0	Rint m=9.75 6.62%	Full 135.4° 99% to 148.6° 100
**Refinement:**	Shift 0.000	Max Peak 0.4	Min Peak -0.3	GooF 1.026

A clear pale colourless plate-shaped crystal with dimensions 0.20 × 0.16 × 0.13 mm^3 was mounted. Data were collected using a XtaLAB Synergy R, DW system, HyPix-Arc 150 diffractometer operating at T = 139.99(10) K.

Data were measured using ω scans with Cu K_α radiation. The diffraction pattern was indexed and the total number of runs and images was based on the strategy calculation from the program CrysAlis^{Pro} system (CCD 44.120a 64-bit (release 22-08-2025)). The maximum resolution achieved was Θ = 74.278° (0.80 Å).

The unit cell was refined using CrysAlis^{Pro} 1.171.44.121a (Rigaku OD, 2025) on 25215 reflections, 22% of the observed reflections.

Data reduction, scaling and absorption corrections were performed using CrysAlis^{Pro} 1.171.44.121a (Rigaku OD, 2025). The final completeness is 100.00 % out to 74.278° in Θ. An analytical absorption correction was performed using CrysAlis^{Pro} 1.171.44.121a (Rigaku Oxford Diffraction, 2025). The analytical numeric absorption correction was done using a multifaceted crystal model based on expressions derived by R.C. Clark & J.S. Reid. (Clark, R. C. & Reid, J. S. (1995). Acta Cryst. A51, 887-897). The empirical absorption correction was done using spherical harmonics, implemented in SCALE3 ABSPACK scaling algorithm. The absorption coefficient μ of this crystal is 0.880 mm^{-1} at this wavelength (λ = 1.54184Å) and the minimum and maximum transmissions are 0.879 and 0.925.

The structure was solved in the space group $P2_12_12_1$ (# 19) by ShelXT (Sheldrick, 2015) using dual methods. It was refined by full matrix least squares minimisation on $\boldsymbol{F}^2$ using version 2019/3 of ShelXL (Sheldrick, 2015). All non-hydrogen atoms were refined anisotropically.

Most hydrogen atom positions were calculated geometrically and refined using the riding model, but some hydrogen atoms were refined freely.

_refine_special_details: Refined as a 2-component inversion twin.

The value of Z' is 3. The moiety formula is C2 F3 O2, C19 H27 N2.

The Flack parameter was refined to 0.1(2). Determination of absolute structure using Bayesian statistics on Bijvoet differences using the Olex2 results in 0.03(4). The chiral atoms in this structure are: N2(S), N4(S), N6(S), C7(S), C11(R), C14(S), C15(R), C26(S), C27(R), C28(S), C47(S), C50(S), C51(R). Note: The Flack parameter is used to determine chirality of the crystal studied, the value should be near 0, a value of 1 means that the stereochemistry is wrong and the model should be inverted. A value of 0.5 means that the crystal consists of a racemic mixture of the two enantiomers.

Data Plots: Diffraction Data

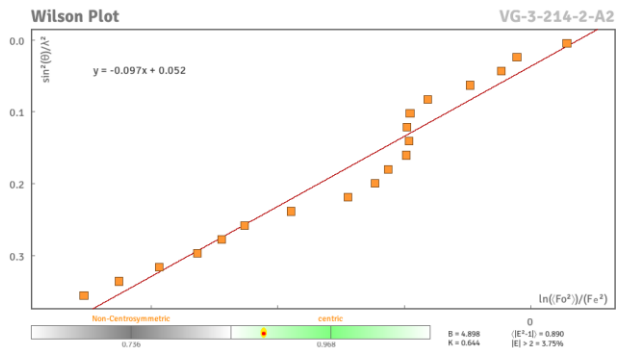

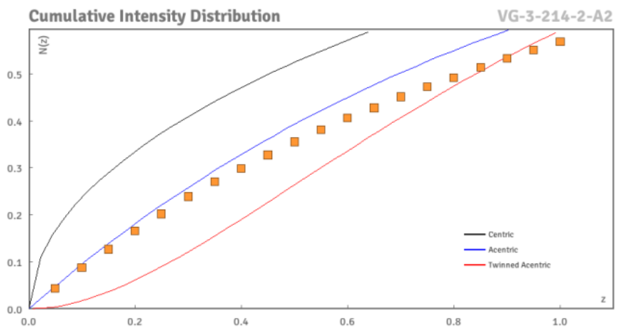

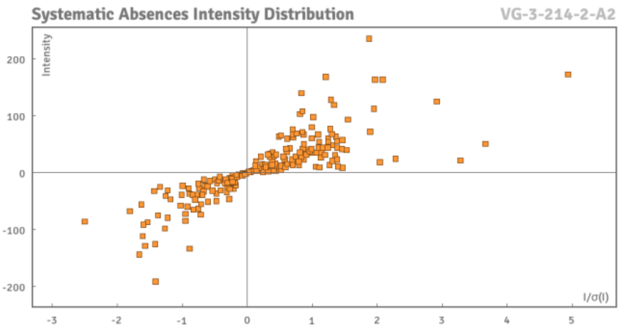

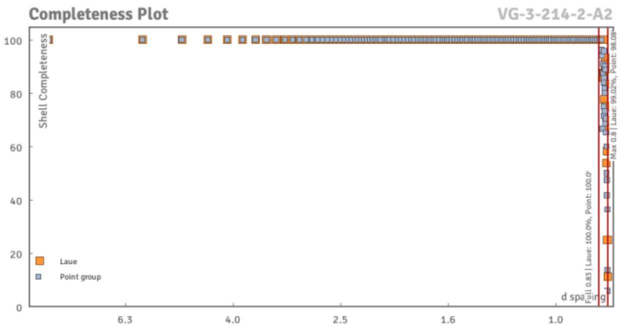

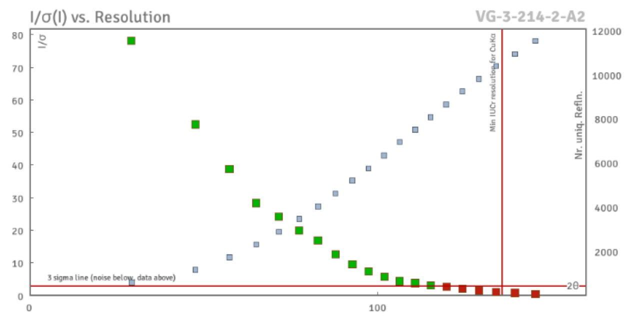

Data Plots: Refinement and Data

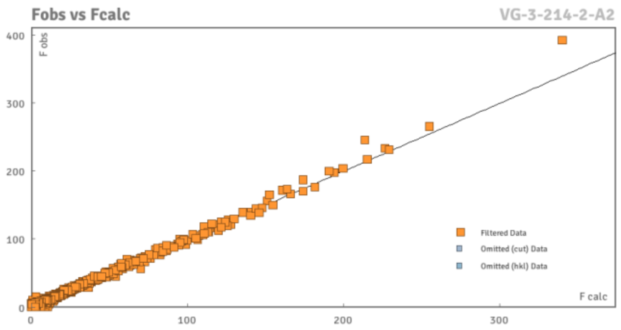

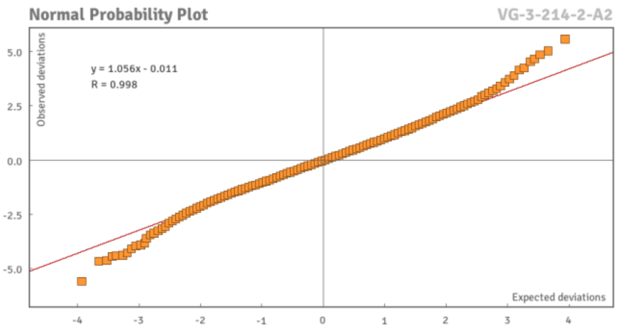

Reflection Statistics

Total reflections (after filtering)	115720	Unique reflections	11869
Completeness	0.981	Mean I/σ	15.94
hkl_{max} collected	(11, 25, 40)	hkl_{min} collected	(-10, -23, -40)
hkl_{max} used	(11, 25, 40)	hkl_{min} used	(-11, 0, 0)
Lim d_{max} collected	100.0	Lim d_{min} collected	0.77
d_{max} used	20.34	d_{min} used	0.8
Friedel pairs	13183	Friedel pairs merged	0
Inconsistent equivalents	2	R_{int}	0.0662

R_{sigma}	0.0385	Intensity transformed	0
Omitted reflections	0	Omitted by user (OMIT hkl)	0
Multiplicity	(9072, 8404, 7200, 3737, 2196, 1264, 878, 716, 530, 452, 326, 273, 176, 107, 59, 55, 34, 10, 10, 7, 3)	Maximum multiplicity	37
Removed systematic absences	313	Filtered off (Shel/OMIT)	0

Table S27: Fractional Atomic Coordinates (×10^4) and Equivalent Isotropic Displacement Parameters (Å^2×10^3) for **VG-3-214-2-A2.** U_{eq} is defined as 1/3 of the trace of the orthogonalised U_{IJ}.

Atom	x	y	z	U_{eq}
F1	9145(15)	3292(8)	10348(4)	94(4)
F2	7129(11)	3717(4)	10109(2)	106(3)
F3	7124(13)	3386(5)	10707(3)	87(4)
F4	8848(14)	3332(8)	10207(3)	92(4)
F5	6828(11)	3481(5)	10505(3)	99(3)
F6	8702(9)	3241(3)	10853.0(19)	82(2)
O1	9548(5)	4575.6(16)	10360.0(11)	70.9(11)
O2	7967(5)	4607.2(16)	10881.2(10)	69.1(11)
C62	8579(7)	4343(2)	10589.1(14)	54.8(13)
C63	8251(16)	3608(6)	10537(4)	63(3)
C68	7993(18)	3675(7)	10442(5)	71(4)
F7	9533(5)	7085.8(14)	9048.0(10)	86.0(11)
F8	10428(6)	6708.3(18)	8499.2(10)	101.4(13)
F9	8115(6)	6694.3(18)	8581.0(16)	123.8(17)
O3	10576(7)	5875.6(19)	9224.1(12)	93.0(15)
O4	8801(5)	5486.3(16)	8802.6(11)	64.4(10)
C64	9576(7)	5928(2)	8963.1(14)	58.3(14)
C65	9360(8)	6600(2)	8786.7(17)	65.1(15)
F10	12946(5)	3066.6(19)	6825.7(10)	91.7(12)
F11	10612(5)	2944(2)	6862.7(17)	131.9(18)
F12	11991(6)	2807.2(16)	7384.7(11)	101.2(14)
O5	12085(6)	4302(2)	6935.3(12)	91.3(15)
O6	10697(4)	3987.7(15)	7472.6(9)	56.4(9)
C66	11532(7)	3895(2)	7163.9(13)	55.6(12)
C67	11783(7)	3182(3)	7062.6(15)	59.1(13)
N1	12508(6)	6641.8(19)	9835.5(12)	58.2(11)
N2	10611(5)	5722.2(17)	10685.0(11)	51.2(10)
C1	13703(6)	6183(2)	9802.3(17)	61.2(14)
C2	14315(8)	5939(3)	9430.3(19)	77.3(18)
C3	15528(8)	5512(3)	9479(2)	77.4(18)
C4	16047(7)	5301(3)	9854(2)	87(2)
C5	15418(7)	5550(3)	10209(2)	75.8(17)
C6	14250(6)	5995(2)	10175.0(17)	61.2(13)
C7	13406(6)	6381(2)	10500.0(16)	57.7(13)
C8	13171(7)	6023(3)	10898.1(16)	65.2(14)
C9	12071(7)	5476(3)	10844.8(17)	67.9(15)
C10	9708(7)	6106(2)	10998.5(15)	61.8(14)
C11	9170(6)	6774(2)	10841.7(15)	56.9(12)
C12	8372(7)	6681(3)	10432.4(17)	66.1(14)
C13	9223(6)	6254(3)	10128.9(17)	61.8(13)
C14	10768(5)	6048(2)	10273.2(13)	44.1(10)
C15	11972(6)	6595(2)	10266.0(13)	46.5(11)
C16	11310(6)	7257(2)	10395.0(13)	52.1(12)
C17	10484(6)	7269(2)	10800.8(14)	52.1(12)
C18	7994(8)	7049(3)	11145.5(17)	73.0(16)
C19	8517(9)	7193(4)	11562.3(18)	91(2)
N3	9671(5)	3138.1(17)	8151.4(10)	43.2(9)
N4	9423(5)	4281.8(18)	9054.7(10)	53.4(10)
C20	8133(6)	3139(2)	8067.4(13)	47.0(11)
C21	7439(6)	3165(3)	7687.4(15)	57.2(13)
C22	5877(7)	3124(3)	7678.8(18)	67.3(15)
C23	5049(7)	3065(3)	8033(2)	72.0(16)

Atom	x	y	z	U_{eq}
C24	5764(7)	3037(2)	8410.3(17)	61.5(13)
C25	7311(6)	3071(2)	8425.7(14)	49.9(12)
C26	8396(6)	2989(2)	8772.3(12)	47.3(11)
C27	9862(5)	3280(2)	8593.8(12)	43.2(10)
C28	9922(6)	4037.3(19)	8637.9(12)	43.5(10)
C29	11429(6)	4351(2)	8544.7(13)	50.7(12)
C30	12616(7)	4245(2)	8879.0(15)	59.6(13)
C31	12052(7)	3882(2)	9257.9(15)	63.9(15)
C32	11834(8)	3150(2)	9169.7(16)	66.7(16)
C33	11313(6)	2957(2)	8739.8(14)	53.6(13)
C34	7923(7)	3279(2)	9181.3(14)	62.8(15)
C35	7915(7)	4016(2)	9167.1(16)	64.7(15)
C36	10581(8)	4219(2)	9385.6(13)	66.3(16)
C37	13374(19)	3965(8)	9568(4)	68(4)
C38	13093(12)	3585(5)	9970(2)	74(3)
C39	12770(30)	3981(13)	9695(6)	56(6)
C40	14300(20)	3691(9)	9670(5)	66(5)
N5	8908(6)	6111(2)	6482.2(12)	58.9(11)
N6	9414(6)	5171.4(19)	7462.4(12)	69.9(14)
C41	10426(7)	6184(2)	6413.2(16)	61.7(14)
C42	11232(7)	6101(2)	6053.3(19)	68.0(15)
C43	12748(8)	6215(3)	6053(2)	82.2(19)
C44	13470(9)	6411(3)	6412(3)	98(3)
C45	12666(9)	6497(3)	6771(3)	86(2)
C46	11130(8)	6383(2)	6773.7(18)	69.4(18)
C47	9940(8)	6462(2)	7094.9(16)	71.7(18)
C48	10406(10)	6305(2)	7533.9(16)	87(2)
C49	10728(9)	5582(2)	7583.1(17)	83(2)
C50	8974(7)	5292(2)	7022.5(14)	59.8(15)
C51	8679(7)	6021(2)	6926.2(14)	60.7(14)
C52	7100(8)	6237(3)	7029.7(17)	77.4(18)
C53	6534(10)	6074(3)	7459.0(18)	95(2)
C54	6576(10)	5342(3)	7578.9(19)	91(2)
C55	6339(8)	4892(3)	7215.6(19)	80.6(18)
C56	7679(7)	4838(3)	6924.7(16)	67.2(16)
C57	8136(9)	5172(3)	7764.7(16)	88(2)
C58	5230(20)	5141(8)	7890(4)	76(4)
C59	5170(16)	5596(6)	8268(4)	95(5)
C60	5950(40)	5328(14)	7972(9)	84(9)
C61	4350(30)	5373(16)	7914(11)	112(13)

Table S28: Anisotropic Displacement Parameters (×10[4]) for **VG-3-214-2-A2.** The anisotropic displacement factor exponent takes the form: $-2\pi^2[h^2a^{*2} \times U_{11} + ... + 2hka^* \times b^* \times U_{12}]$

Atom	U_{11}	U_{22}	U_{33}	U_{23}	U_{13}	U_{12}
F1	115(7)	51(5)	118(10)	-25(7)	-6(7)	-5(5)
F2	151(7)	110(6)	58(4)	-7(4)	-28(5)	-58(6)
F3	138(9)	61(5)	62(5)	-19(4)	58(6)	-43(6)
F4	142(8)	76(5)	59(5)	-23(4)	51(5)	-36(6)
F5	88(5)	78(5)	132(8)	-4(6)	-32(6)	-16(4)
F6	117(6)	44(3)	85(4)	15(3)	-19(4)	-12(3)
O1	93(3)	49.4(19)	70(2)	1.8(16)	25(2)	-18(2)
O2	106(3)	52.1(19)	49.3(19)	-7.6(16)	17(2)	-10(2)
C62	75(4)	49(3)	40(2)	6(2)	3(2)	-13(3)
C63	91(7)	57(6)	42(5)	-18(5)	23(5)	-24(6)
C68	100(8)	60(6)	54(7)	-7(6)	32(6)	-24(6)
F7	132(3)	45.2(15)	81(2)	-18.3(15)	15(2)	-0.2(18)
F8	167(4)	79(2)	58.0(19)	8.8(17)	23(2)	-3(3)
F9	132(4)	55(2)	185(4)	13(2)	-67(4)	4(2)
O3	153(5)	63(2)	62(2)	-4.7(19)	-39(3)	3(3)
O4	82(3)	43.3(18)	68(2)	-5.7(16)	-6(2)	1.9(18)
C64	90(4)	43(2)	41(2)	-9(2)	5(3)	2(3)

Atom	U_{11}	U_{22}	U_{33}	U_{23}	U_{13}	U_{12}
C65	93(4)	43(3)	59(3)	-6(2)	-9(3)	1(3)
F10	108(3)	94(2)	73(2)	4.9(18)	37(2)	32(2)
F11	101(3)	104(3)	191(4)	-87(3)	-32(3)	6(3)
F12	164(4)	58.9(19)	81(2)	19.4(17)	41(3)	35(2)
O5	144(4)	61(2)	68(2)	14(2)	36(3)	-11(3)
O6	85(2)	45.8(17)	38.0(16)	4.1(13)	10.0(17)	1.0(17)
C66	78(4)	52(3)	38(2)	5(2)	2(2)	-2(3)
C67	65(4)	60(3)	51(3)	-4(2)	8(3)	5(3)
N1	84(3)	43(2)	47(2)	-3.3(17)	13(2)	-5(2)
N2	68(3)	34.5(17)	51(2)	6.8(16)	-1(2)	-3.8(19)
C1	63(3)	42(2)	78(4)	-15(2)	27(3)	-9(2)
C2	96(5)	59(3)	77(4)	-18(3)	24(4)	-20(3)
C3	71(4)	71(4)	90(5)	-32(3)	23(4)	-1(3)
C4	66(4)	87(4)	107(5)	-45(4)	-4(4)	-2(3)
C5	62(4)	71(3)	94(4)	-35(3)	-9(3)	5(3)
C6	58(3)	53(3)	73(3)	-21(3)	-2(3)	-1(3)
C7	54(3)	48(3)	71(3)	-18(2)	-7(3)	-1(2)
C8	72(4)	61(3)	63(3)	-5(3)	-20(3)	14(3)
C9	90(4)	52(3)	63(3)	8(2)	-10(3)	19(3)
C10	79(4)	53(3)	54(3)	11(2)	14(3)	-1(3)
C11	63(3)	47(3)	61(3)	2(2)	9(3)	1(2)
C12	55(3)	64(3)	80(4)	-3(3)	-7(3)	8(3)
C13	59(3)	58(3)	69(3)	-5(2)	-11(3)	0(3)
C14	53(3)	35(2)	44(2)	3.8(17)	-4(2)	-6(2)
C15	67(3)	35(2)	37(2)	-3.3(17)	4(2)	-4(2)
C16	78(4)	34(2)	45(2)	-0.3(18)	2(2)	-5(2)
C17	68(3)	41(2)	47(2)	-2.2(19)	5(2)	4(2)
C18	75(4)	73(3)	71(4)	-1(3)	15(3)	8(3)
C19	94(5)	114(5)	64(4)	3(4)	12(4)	16(4)
N3	54(2)	43.6(19)	32.0(17)	-2.3(15)	0.1(17)	-1.5(18)
N4	84(3)	39.4(18)	36.4(19)	-5.4(15)	8(2)	1(2)
C20	60(3)	41(2)	39(2)	-0.7(18)	1(2)	-2(2)
C21	67(4)	58(3)	46(3)	2(2)	-3(2)	-5(3)
C22	64(4)	71(3)	67(3)	5(3)	-10(3)	-8(3)
C23	57(3)	68(4)	92(4)	6(3)	-1(3)	2(3)
C24	64(4)	53(3)	67(3)	0(2)	15(3)	1(3)
C25	63(3)	42(2)	45(2)	0(2)	9(2)	-3(2)
C26	72(3)	37(2)	33(2)	1.0(17)	7(2)	-4(2)
C27	64(3)	37(2)	29.0(19)	-1.2(16)	-1(2)	0(2)
C28	63(3)	33.3(19)	34(2)	-0.8(16)	6(2)	0(2)
C29	69(3)	43(2)	40(2)	0.2(18)	4(2)	-3(2)
C30	76(4)	49(3)	54(3)	-6(2)	-7(3)	-6(3)
C31	96(4)	43(2)	53(3)	-3(2)	-22(3)	-1(3)
C32	101(5)	41(2)	58(3)	1(2)	-32(3)	2(3)
C33	77(4)	36(2)	47(2)	-2.4(19)	-14(2)	8(2)
C34	93(4)	51(3)	44(3)	-7(2)	23(3)	-15(3)
C35	83(4)	54(3)	56(3)	-14(2)	32(3)	-10(3)
C36	117(5)	50(3)	33(2)	-1(2)	-2(3)	-9(3)
C37	98(14)	68(6)	36(7)	-7(6)	-8(6)	-11(8)
C38	86(7)	96(7)	42(5)	6(4)	-15(5)	-4(6)
C39	80(18)	60(10)	28(11)	-6(9)	-3(8)	-5(11)
C40	74(13)	78(11)	47(9)	-6(8)	-15(9)	4(10)
N5	81(4)	52(2)	44(2)	7.5(18)	-20(2)	1(2)
N6	124(4)	39(2)	46(2)	0.4(17)	-26(3)	14(3)
C41	88(4)	37(2)	60(3)	1(2)	-19(3)	10(3)
C42	78(4)	43(3)	83(4)	-8(3)	-16(3)	7(3)
C43	77(5)	48(3)	122(5)	-17(3)	-7(4)	12(3)
C44	80(5)	50(3)	164(8)	-5(4)	-49(5)	11(3)
C45	102(6)	48(3)	108(5)	-7(3)	-44(5)	12(3)
C46	102(5)	34(2)	73(4)	-1(2)	-44(4)	7(3)
C47	123(5)	42(2)	50(3)	-5(2)	-28(3)	11(3)
C48	163(7)	43(3)	56(3)	-4(2)	-47(4)	5(4)

Atom	U_{11}	U_{22}	U_{33}	U_{23}	U_{13}	U_{12}
C49	149(6)	43(3)	57(3)	1(2)	-60(4)	6(3)
C50	103(4)	39(2)	38(2)	1.6(18)	-23(3)	2(3)
C51	95(4)	45(3)	42(2)	1(2)	-16(3)	10(3)
C52	115(5)	58(3)	59(3)	13(3)	-2(3)	24(3)
C53	147(6)	75(4)	62(3)	17(3)	13(4)	41(4)
C54	142(6)	72(4)	60(3)	19(3)	13(4)	30(4)
C55	92(5)	75(4)	75(4)	23(3)	2(4)	6(4)
C56	99(5)	54(3)	49(3)	5(2)	-18(3)	-1(3)
C57	156(7)	67(4)	40(3)	2(2)	-6(4)	31(4)
C58	88(12)	73(8)	66(7)	9(6)	3(8)	-2(8)
C59	105(9)	116(9)	64(7)	8(6)	25(7)	4(7)
C60	90(20)	90(18)	73(17)	26(13)	2(14)	-26(14)
C61	76(19)	110(20)	150(30)	41(19)	40(20)	9(15)

Table S29: Bond Lengths in Å for **VG-3-214-2-A2**.

Atom	Atom	Length/Å
F1	C68	1.325(15)
F2	C68	1.337(15)
F3	C68	1.302(15)
F4	C63	1.328(13)
F5	C63	1.300(14)
F6	C63	1.338(13)
O1	C62	1.238(6)
O2	C62	1.225(6)
C62	C63	1.532(13)
C62	C68	1.534(15)
F7	C65	1.315(6)
F8	C65	1.357(7)
F9	C65	1.312(7)
O3	C64	1.239(7)
O4	C64	1.250(6)
C64	C65	1.497(7)
F10	C67	1.316(6)
F11	C67	1.324(7)
F12	C67	1.313(6)
O5	C66	1.219(6)
O6	C66	1.269(6)
C66	C67	1.506(7)
N1	C1	1.420(7)
N1	C15	1.490(6)
N2	C9	1.490(7)
N2	C10	1.520(6)
N2	C14	1.507(5)
C1	C2	1.423(7)
C1	C6	1.368(8)
C2	C3	1.397(9)
C3	C4	1.380(10)
C4	C5	1.384(8)
C5	C6	1.385(8)
C6	C7	1.521(7)
C7	C8	1.507(7)
C7	C15	1.554(7)
C8	C9	1.494(8)
C10	C11	1.530(7)
C11	C12	1.528(7)
C11	C17	1.551(7)
C11	C18	1.550(7)
C12	C13	1.522(8)
C13	C14	1.516(7)
C14	C15	1.548(6)
C15	C16	1.528(6)

Atom	Atom	Length/Å
C16	C17	1.519(6)
C18	C19	1.470(8)
N3	C20	1.399(7)
N3	C27	1.486(5)
N4	C28	1.518(5)
N4	C35	1.496(7)
N4	C36	1.501(7)
C20	C21	1.389(6)
C20	C25	1.390(6)
C21	C22	1.396(8)
C22	C23	1.380(8)
C23	C24	1.390(8)
C24	C25	1.383(8)
C25	C26	1.500(7)
C26	C27	1.550(7)
C26	C34	1.522(6)
C27	C28	1.548(6)
C27	C33	1.529(7)
C28	C29	1.520(7)
C29	C30	1.538(7)
C30	C31	1.528(7)
C31	C32	1.530(7)
C31	C36	1.539(9)
C31	C37	1.563(17)
C31	C39	1.58(2)
C32	C33	1.532(6)
C34	C35	1.500(7)
C37	C38	1.547(16)
C39	C40	1.49(3)
N5	C41	1.381(8)
N5	C51	1.478(6)
N6	C49	1.493(8)
N6	C50	1.511(6)
N6	C57	1.509(9)
C41	C42	1.389(8)
C41	C46	1.395(7)
C42	C43	1.373(9)
C43	C44	1.399(10)
C44	C45	1.386(11)
C45	C46	1.390(10)
C46	C47	1.503(9)
C47	C48	1.529(7)
C47	C51	1.541(8)
C48	C49	1.507(7)
C50	C51	1.540(6)
C50	C56	1.513(8)
C51	C52	1.514(9)
C52	C53	1.529(8)
C53	C54	1.540(9)
C54	C55	1.515(9)
C54	C57	1.558(11)
C54	C58	1.625(19)
C54	C60	1.40(3)
C55	C56	1.532(9)
C58	C59	1.54(2)
C60	C61	1.44(5)

Table S30: Bond Angles in ° for **VG-3-214-2-A2**.

Atom	Atom	Atom	Angle/°
O1	C62	C63	116.0(6)
O1	C62	C68	112.8(7)

Atom	Atom	Atom	Angle/°
O2	C62	O1	128.0(5)
O2	C62	C63	115.5(6)
O2	C62	C68	118.8(7)
F4	C63	F6	105.7(11)
F4	C63	C62	115.3(11)
F5	C63	F4	104.0(11)
F5	C63	F6	104.1(10)
F5	C63	C62	113.0(11)
F6	C63	C62	113.6(9)
F1	C68	F2	107.2(12)
F1	C68	C62	109.2(13)
F2	C68	C62	113.3(11)
F3	C68	F1	110.6(14)
F3	C68	F2	103.1(12)
F3	C68	C62	113.3(11)
O3	C64	O4	128.7(5)
O3	C64	C65	115.9(5)
O4	C64	C65	115.1(5)
F7	C65	F8	104.3(5)
F7	C65	C64	114.9(4)
F8	C65	C64	108.9(5)
F9	C65	F7	108.8(5)
F9	C65	F8	102.4(5)
F9	C65	C64	116.1(5)
O5	C66	O6	128.7(5)
O5	C66	C67	117.3(5)
O6	C66	C67	113.9(4)
F10	C67	F11	105.5(4)
F10	C67	C66	114.7(5)
F11	C67	C66	110.1(5)
F12	C67	F10	104.9(5)
F12	C67	F11	107.2(5)
F12	C67	C66	113.8(4)
C1	N1	C15	105.8(4)
C9	N2	C10	113.5(4)
C9	N2	C14	112.3(4)
C14	N2	C10	115.2(3)
N1	C1	C2	125.6(6)
C6	C1	N1	112.6(4)
C6	C1	C2	121.8(6)
C3	C2	C1	114.7(6)
C4	C3	C2	123.7(6)
C3	C4	C5	119.7(6)
C4	C5	C6	118.5(6)
C1	C6	C5	121.5(5)
C1	C6	C7	107.6(5)
C5	C6	C7	130.9(5)
C6	C7	C15	102.0(4)
C8	C7	C6	115.0(4)
C8	C7	C15	116.5(4)
C9	C8	C7	110.5(4)
N2	C9	C8	111.4(4)
N2	C10	C11	113.4(4)
C10	C11	C17	111.6(5)
C10	C11	C18	108.6(4)
C12	C11	C10	109.3(4)
C12	C11	C17	110.9(4)
C12	C11	C18	106.9(5)
C18	C11	C17	109.4(4)
C13	C12	C11	114.2(4)
C14	C13	C12	114.1(4)
N2	C14	C13	108.4(4)

Atom	Atom	Atom	Angle/°
N2	C14	C15	113.2(3)
C13	C14	C15	115.2(4)
N1	C15	C7	102.6(4)
N1	C15	C14	106.4(3)
N1	C15	C16	109.2(3)
C14	C15	C7	111.2(4)
C16	C15	C7	115.4(4)
C16	C15	C14	111.2(4)
C17	C16	C15	116.3(4)
C16	C17	C11	115.6(4)
C19	C18	C11	116.8(6)
C20	N3	C27	107.7(4)
C35	N4	C28	111.5(4)
C35	N4	C36	114.3(4)
C36	N4	C28	114.7(4)
C21	C20	N3	127.8(4)
C21	C20	C25	121.5(5)
C25	C20	N3	110.6(4)
C20	C21	C22	117.4(5)
C23	C22	C21	121.5(5)
C22	C23	C24	120.2(6)
C25	C24	C23	119.2(5)
C20	C25	C26	107.9(4)
C24	C25	C20	120.1(5)
C24	C25	C26	131.8(5)
C25	C26	C27	102.6(3)
C25	C26	C34	116.3(5)
C34	C26	C27	114.6(4)
N3	C27	C26	101.3(3)
N3	C27	C28	106.8(3)
N3	C27	C33	108.5(4)
C28	C27	C26	112.0(4)
C33	C27	C26	115.6(3)
C33	C27	C28	111.7(4)
N4	C28	C27	113.6(3)
N4	C28	C29	107.6(4)
C29	C28	C27	115.5(4)
C28	C29	C30	114.1(4)
C31	C30	C29	114.7(5)
C30	C31	C32	111.1(4)
C30	C31	C36	106.6(4)
C30	C31	C37	103.0(7)
C30	C31	C39	122.6(10)
C32	C31	C36	112.2(5)
C32	C31	C37	108.8(7)
C32	C31	C39	110.3(11)
C36	C31	C37	114.8(7)
C36	C31	C39	92.5(10)
C31	C32	C33	117.4(4)
C27	C33	C32	115.7(4)
C35	C34	C26	111.2(4)
N4	C35	C34	111.4(5)
N4	C36	C31	115.4(4)
C38	C37	C31	112.1(11)
C40	C39	C31	105.9(15)
C41	N5	C51	108.0(4)
C49	N6	C50	111.4(4)
C49	N6	C57	114.8(5)
C57	N6	C50	115.3(5)
N5	C41	C42	129.2(5)
N5	C41	C46	109.5(5)
C42	C41	C46	121.2(6)

Atom	Atom	Atom	Angle/˚
C43	C42	C41	119.4(6)
C42	C43	C44	120.0(8)
C45	C44	C43	120.6(7)
C44	C45	C46	119.7(7)
C41	C46	C47	107.7(6)
C45	C46	C41	119.1(7)
C45	C46	C47	133.1(6)
C46	C47	C48	116.2(6)
C46	C47	C51	101.7(4)
C48	C47	C51	114.4(5)
C49	C48	C47	110.9(4)
N6	C49	C48	111.6(5)
N6	C50	C51	113.3(4)
N6	C50	C56	107.5(4)
C56	C50	C51	114.5(5)
N5	C51	C47	100.3(5)
N5	C51	C50	107.2(4)
N5	C51	C52	108.2(4)
C50	C51	C47	111.3(4)
C52	C51	C47	115.5(5)
C52	C51	C50	113.1(5)
C51	C52	C53	116.7(5)
C52	C53	C54	115.8(5)
C53	C54	C57	109.6(7)
C53	C54	C58	112.7(7)
C55	C54	C53	112.4(5)
C55	C54	C57	107.2(6)
C55	C54	C58	103.7(8)
C57	C54	C58	111.0(7)
C60	C54	C53	104.1(13)
C60	C54	C55	130.6(14)
C60	C54	C57	89.7(14)
C54	C55	C56	114.9(6)
C50	C56	C55	114.9(5)
N6	C57	C54	114.8(4)
C59	C58	C54	112.1(13)
C54	C60	C61	106(3)

Table S31: Torsion Angles in ˚ for **VG-3-214-2-A2**.

Atom	Atom	Atom	Atom	Angle/˚
O1	C62	C63	F4	-11.2(14)
O1	C62	C63	F5	-130.6(10)
O1	C62	C63	F6	111.1(9)
O1	C62	C68	F1	47.8(14)
O1	C62	C68	F2	-71.5(13)
O1	C62	C68	F3	171.5(11)
O2	C62	C63	F4	176.3(10)
O2	C62	C63	F5	56.9(12)
O2	C62	C63	F6	-61.4(12)
O2	C62	C68	F1	-139.2(10)
O2	C62	C68	F2	101.5(11)
O2	C62	C68	F3	-15.5(16)
O3	C64	C65	F7	-37.1(8)
O3	C64	C65	F8	79.4(6)
O3	C64	C65	F9	-165.7(5)
O4	C64	C65	F7	148.4(5)
O4	C64	C65	F8	-95.1(6)
O4	C64	C65	F9	19.8(8)
O5	C66	C67	F10	21.4(8)
O5	C66	C67	F11	-97.3(7)
O5	C66	C67	F12	142.3(6)

Atom	Atom	Atom	Atom	Angle/˚
O6	C66	C67	F10	-161.7(5)
O6	C66	C67	F11	79.6(6)
O6	C66	C67	F12	-40.8(7)
N1	C1	C2	C3	177.5(5)
N1	C1	C6	C5	180.0(5)
N1	C1	C6	C7	-2.8(6)
N1	C15	C16	C17	170.5(4)
N2	C10	C11	C12	51.5(6)
N2	C10	C11	C17	-71.5(6)
N2	C10	C11	C18	167.8(5)
N2	C14	C15	N1	153.7(4)
N2	C14	C15	C7	42.7(5)
N2	C14	C15	C16	-87.4(5)
C1	N1	C15	C7	28.7(4)
C1	N1	C15	C14	-88.3(5)
C1	N1	C15	C16	151.6(4)
C1	C2	C3	C4	4.2(9)
C1	C6	C7	C8	147.5(5)
C1	C6	C7	C15	20.4(5)
C2	C1	C6	C5	-0.8(8)
C2	C1	C6	C7	176.4(5)
C2	C3	C4	C5	-4.3(10)
C3	C4	C5	C6	1.6(9)
C4	C5	C6	C1	0.8(8)
C4	C5	C6	C7	-175.7(6)
C5	C6	C7	C8	-35.7(8)
C5	C6	C7	C15	-162.8(6)
C6	C1	C2	C3	-1.6(8)
C6	C7	C8	C9	-70.0(6)
C6	C7	C15	N1	-29.3(4)
C6	C7	C15	C14	84.1(5)
C6	C7	C15	C16	-148.0(4)
C7	C8	C9	N2	-57.3(6)
C7	C15	C16	C17	-74.5(6)
C8	C7	C15	N1	-155.5(4)
C8	C7	C15	C14	-42.0(5)
C8	C7	C15	C16	85.9(5)
C9	N2	C10	C11	129.3(5)
C9	N2	C14	C13	177.6(4)
C9	N2	C14	C15	-53.2(5)
C10	N2	C9	C8	-72.3(5)
C10	N2	C14	C13	-50.3(5)
C10	N2	C14	C15	78.9(5)
C10	C11	C12	C13	-47.6(6)
C10	C11	C17	C16	87.7(5)
C10	C11	C18	C19	63.5(7)
C11	C12	C13	C14	-5.1(7)
C12	C11	C17	C16	-34.3(6)
C12	C11	C18	C19	-178.7(5)
C12	C13	C14	N2	54.2(5)
C12	C13	C14	C15	-73.9(6)
C13	C14	C15	N1	-80.7(5)
C13	C14	C15	C7	168.3(4)
C13	C14	C15	C16	38.1(5)
C14	N2	C9	C8	60.6(6)
C14	N2	C10	C11	-2.3(6)
C14	C15	C16	C17	53.4(6)
C15	N1	C1	C2	163.7(5)
C15	N1	C1	C6	-17.1(6)
C15	C7	C8	C9	49.3(6)
C15	C16	C17	C11	-56.4(6)
C17	C11	C12	C13	75.8(6)

Atom	Atom	Atom	Atom	Angle/˚
C17	C11	C18	C19	-58.5(7)
C18	C11	C12	C13	-165.0(5)
C18	C11	C17	C16	-152.1(4)
N3	C20	C21	C22	176.6(5)
N3	C20	C25	C24	-177.7(4)
N3	C20	C25	C26	-2.6(5)
N3	C27	C28	N4	154.1(4)
N3	C27	C28	C29	-80.9(5)
N3	C27	C33	C32	169.9(4)
N4	C28	C29	C30	53.8(5)
C20	N3	C27	C26	29.7(4)
C20	N3	C27	C28	-87.7(4)
C20	N3	C27	C33	151.8(4)
C20	C21	C22	C23	0.3(9)
C20	C25	C26	C27	20.8(4)
C20	C25	C26	C34	146.6(4)
C21	C20	C25	C24	-1.0(7)
C21	C20	C25	C26	174.1(4)
C21	C22	C23	C24	-0.7(9)
C22	C23	C24	C25	0.3(8)
C23	C24	C25	C20	0.6(7)
C23	C24	C25	C26	-173.2(5)
C24	C25	C26	C27	-164.9(5)
C24	C25	C26	C34	-39.0(7)
C25	C20	C21	C22	0.6(8)
C25	C26	C27	N3	-29.7(4)
C25	C26	C27	C28	83.7(4)
C25	C26	C27	C33	-146.8(4)
C25	C26	C34	C35	-69.2(6)
C26	C27	C28	N4	44.1(5)
C26	C27	C28	C29	169.0(4)
C26	C27	C33	C32	-77.2(5)
C26	C34	C35	N4	-58.2(7)
C27	N3	C20	C21	165.5(4)
C27	N3	C20	C25	-18.0(5)
C27	C26	C34	C35	50.3(7)
C27	C28	C29	C30	-74.2(5)
C28	N4	C35	C34	59.9(5)
C28	N4	C36	C31	-3.4(6)
C28	C27	C33	C32	52.4(5)
C28	C29	C30	C31	-3.7(6)
C29	C30	C31	C32	73.9(6)
C29	C30	C31	C36	-48.6(5)
C29	C30	C31	C37	-169.7(7)
C29	C30	C31	C39	-152.7(12)
C30	C31	C32	C33	-34.0(8)
C30	C31	C36	N4	53.0(5)
C30	C31	C37	C38	-175.7(9)
C30	C31	C39	C40	-68.2(19)
C31	C32	C33	C27	-55.6(7)
C32	C31	C36	N4	-68.8(5)
C32	C31	C37	C38	-57.8(12)
C32	C31	C39	C40	65.6(17)
C33	C27	C28	N4	-87.3(5)
C33	C27	C28	C29	37.6(5)
C34	C26	C27	N3	-156.7(4)
C34	C26	C27	C28	-43.2(5)
C34	C26	C27	C33	86.3(5)
C35	N4	C28	C27	-53.0(5)
C35	N4	C28	C29	178.0(4)
C35	N4	C36	C31	127.1(4)
C36	N4	C28	C27	78.9(5)

Atom	Atom	Atom	Atom	Angle/˚
C36	N4	C28	C29	-50.2(5)
C36	N4	C35	C34	-72.2(5)
C36	C31	C32	C33	85.1(6)
C36	C31	C37	C38	68.8(12)
C36	C31	C39	C40	-179.7(15)
C37	C31	C32	C33	-146.7(7)
C37	C31	C36	N4	166.3(7)
C39	C31	C32	C33	-173.3(11)
C39	C31	C36	N4	178.1(10)
N5	C41	C42	C43	178.5(5)
N5	C41	C46	C45	-178.9(5)
N5	C41	C46	C47	-1.9(5)
N5	C51	C52	C53	169.7(5)
N6	C50	C51	N5	156.1(5)
N6	C50	C51	C47	47.4(7)
N6	C50	C51	C52	-84.6(6)
N6	C50	C56	C55	54.4(6)
C41	N5	C51	C47	34.0(5)
C41	N5	C51	C50	-82.3(5)
C41	N5	C51	C52	155.4(4)
C41	C42	C43	C44	0.3(8)
C41	C46	C47	C48	147.6(4)
C41	C46	C47	C51	22.6(5)
C42	C41	C46	C45	-0.2(7)
C42	C41	C46	C47	176.8(4)
C42	C43	C44	C45	-0.5(9)
C43	C44	C45	C46	0.4(9)
C44	C45	C46	C41	-0.1(8)
C44	C45	C46	C47	-176.1(5)
C45	C46	C47	C48	-36.1(8)
C45	C46	C47	C51	-161.0(6)
C46	C41	C42	C43	0.1(8)
C46	C47	C48	C49	-67.4(8)
C46	C47	C51	N5	-33.0(5)
C46	C47	C51	C50	80.2(5)
C46	C47	C51	C52	-149.0(4)
C47	C48	C49	N6	-56.8(8)
C47	C51	C52	C53	-78.9(7)
C48	C47	C51	N5	-159.1(5)
C48	C47	C51	C50	-45.9(7)
C48	C47	C51	C52	84.8(6)
C49	N6	C50	C51	-54.6(7)
C49	N6	C50	C56	177.9(4)
C49	N6	C57	C54	127.8(5)
C50	N6	C49	C48	59.2(7)
C50	N6	C57	C54	-3.8(7)
C50	C51	C52	C53	51.0(7)
C51	N5	C41	C42	160.2(5)
C51	N5	C41	C46	-21.2(5)
C51	C47	C48	C49	50.8(9)
C51	C50	C56	C55	-72.4(6)
C51	C52	C53	C54	-56.7(9)
C52	C53	C54	C55	-31.5(10)
C52	C53	C54	C57	87.7(8)
C52	C53	C54	C58	-148.2(9)
C52	C53	C54	C60	-177.5(16)
C53	C54	C55	C56	74.0(8)
C53	C54	C57	N6	-70.3(7)
C53	C54	C58	C59	-52.4(14)
C53	C54	C60	C61	79(2)
C54	C55	C56	C50	-5.6(7)
C55	C54	C57	N6	52.0(7)

Atom	Atom	Atom	Atom	Angle/°
C55	C54	C58	C59	-174.2(10)
C55	C54	C60	C61	-59(3)
C56	C50	C51	N5	-80.1(6)
C56	C50	C51	C47	171.1(5)
C56	C50	C51	C52	39.2(6)
C57	N6	C49	C48	-74.2(6)
C57	N6	C50	C51	78.5(6)
C57	N6	C50	C56	-49.0(6)
C57	C54	C55	C56	-46.5(7)
C57	C54	C58	C59	70.9(12)
C57	C54	C60	C61	-171(2)
C58	C54	C55	C56	-164.1(7)
C58	C54	C57	N6	164.6(7)
C60	C54	C55	C56	-151.5(18)
C60	C54	C57	N6	-175.2(13)

Table S32: Hydrogen Fractional Atomic Coordinates (×10^4) and Equivalent Isotropic Displacement Parameters (Å^2×10^3) for **VG-3-214-2-A2.** U_{eq} is defined as 1/3 of the trace of the orthogonalised U_{ij}.

Atom	x	y	z	U_{eq}
H1	11630(60)	6660(30)	9644(16)	85
H2	10006.66	5317.25	10631.52	61
H2A	13930.36	6057.71	9169.7	93
H3A	16022.65	5358.02	9240.11	93
H4A	16832.73	4986.56	9868.63	104
H5A	15780.25	5418.06	10469.19	91
H7	14000.1	6786.38	10560.38	69
H8A	14138.24	5842.74	10995.2	78
H8B	12794.89	6334.04	11107.01	78
H9A	12486.22	5147.18	10652.47	82
H9B	11906.74	5255.76	11111.03	82
H10A	10333.27	6175.02	11244.94	74
H10B	8826.79	5841.37	11080.27	74
H12A	7378.62	6480.69	10483.2	79
H12B	8203.1	7118.48	10307.8	79
H13A	8624.09	5854.53	10072.58	74
H13B	9327.6	6498.32	9868.69	74
H14	11118.29	5699.32	10079.71	53
H16A	12135.29	7581.38	10407.91	63
H16B	10609.56	7403.51	10178.86	63
H17A	10086.45	7717.84	10844.54	63
H17B	11214.45	7179.58	11021.82	63
H18A	7576.73	7459.17	11028.35	88
H18B	7161.33	6729.04	11164.79	88
H19A	9347.25	7508.39	11550.37	136
H19B	8860.81	6786.45	11692.35	136
H19C	7692.53	7381.48	11722	136
H3	10210(60)	3400(20)	7934(14)	67(15)
H4	9269.45	4765.5	9019.83	64
H21	8005.45	3207.46	7442.92	69
H22	5373.08	3138.15	7423.07	81
H23	3986.17	3044.01	8019.31	86
H24	5196.34	2993.99	8654.67	74
H26	8558.51	2507.17	8813.21	57
H28	9194.43	4219.75	8434.83	52
H29A	11281.6	4829.47	8505.7	61
H29B	11812.69	4169.05	8284.26	61
H30A	13461.72	3993.96	8760.48	72
H30B	13008.45	4679.18	8964.4	72
H32A	11095.39	2976.09	9367.97	80
H32B	12797.44	2923.78	9223.48	80
H33A	12120.02	3068.82	8543.92	64

Atom	x	y	z	U_{eq}
H33B	11177.4	2474.43	8731.88	64
H34A	6908.25	3119.12	9252.21	75
H34B	8623.55	3129.87	9396.87	75
H35A	7166.03	4165.91	8964.2	78
H35B	7618.14	4190.33	9437.9	78
H36A	10138.39	3968.51	9615.19	80
H36B	10820.88	4664.72	9488.44	80
H37A	14310.72	3803.24	9440.29	81
H37B	13506.81	4437.16	9630.01	81
H38A	13037.92	3112.86	9912.15	112
H38B	12146.65	3731.46	10091.75	112
H38C	13916.82	3669.78	10161.03	112
H39A	12825.07	4453.95	9764.02	67
H39B	12166.97	3753.74	9905.9	67
H40A	14859.28	3900.71	9447.46	100
H40B	14224.64	3218.09	9618.23	100
H40C	14828.49	3764.52	9929.29	100
H5	8250(60)	5830(30)	6297(16)	82(18)
H6	9798.38	4709.81	7465.94	84
H42	10737.68	5966.05	5810.1	82
H43	13308.19	6161.67	5807.87	99
H44	14520.89	6486.51	6410.78	118
H45	13161.8	6632.67	7013.57	103
H47	9582.05	6927.75	7087.05	86
H48A	11312.32	6561.31	7605.21	105
H48B	9593.47	6434.8	7723.59	105
H49A	11601.59	5461.85	7411.96	100
H49B	10988.09	5490.08	7871.78	100
H50	9838.25	5150.32	6849.02	72
H52A	6408.54	6033.76	6829.54	93
H52B	7038.71	6719.24	6992.04	93
H53A	5487.61	6231.09	7483.05	114
H53B	7142.84	6323.61	7658.93	114
H55A	5462.99	5051.45	7058.8	97
H55B	6094.15	4446.79	7318.29	97
H56A	8049.02	4379.21	6929.14	81
H56B	7330.55	4932.41	6643.41	81
H57A	8364.06	5493.89	7983.13	105
H57B	8076.45	4732.51	7893.42	105
H58A	4263.07	5163.72	7743.53	91
H58B	5380.33	4681.58	7981.67	91
H59A	4867.92	6038.1	8183.14	143
H59B	6162.28	5614.61	8396.14	143
H59C	4440.99	5422.45	8464.57	143
H60A	6316.79	5702.5	8136.88	101
H60B	6212.7	4913.8	8113.5	101
H61A	4066.16	5135.97	7665.21	168
H61B	4060.29	5835.7	7888.76	168
H61C	3837.57	5177.59	8149.82	168

Table S33: Hydrogen Bond information for **VG-3-214-2-A2**.

D	H	A	d(D-H)/Å	d(H-A)/Å	d(D-A)/Å	D-H-A/deg
N1	H1	O3	1.01(4)	2.30(5)	3.066(6)	132(5)
N2	H2	O1	1.00	1.80	2.733(5)	154.2
N2	H2	O2	1.00	2.46	3.335(6)	145.5
N3	H3	O6	1.01(4)	1.97(4)	2.959(5)	164(5)
N4	H4	O3	1.00	2.63	3.446(6)	139.1
N4	H4	O4	1.00	1.68	2.644(5)	160.1
C35	H35B	F2	0.99	2.44	3.217(9)	135.5
C36	H36A	F4	0.99	2.60	3.586(12)	177.2
C36	H36B	O3	0.99	2.62	3.411(6)	136.9

D	H	A	d(D-H)/Å	d(H-A)/Å	d(D-A)/Å	D-H-A/deg
N5	H5	O2[1]	1.02(4)	1.96(4)	2.966(6)	171(5)
N6	H6	O6	1.00	1.67	2.667(5)	171.5
C47	H47	N3[2]	1.00	2.67	3.521(6)	143.4
C50	H50	O5	1.00	2.66	3.441(8)	135.1

––––

[1]3/2-x,1-y,-1/2+z; [2]2-x,1/2+y,3/2-z

Table S34: Atomic Occupancies for all atoms that are not fully occupied in **VG-3-214-2-A2**.

Atom	Occupancy
F1	0.463(8)
F2	0.463(8)
F3	0.463(8)
F4	0.537(8)
F5	0.537(8)
F6	0.537(8)
C63	0.537(8)
C68	0.463(8)
C37	0.649(14)
H37A	0.649(14)
H37B	0.649(14)
C38	0.649(14)
H38A	0.649(14)
H38B	0.649(14)
H38C	0.649(14)
C39	0.351(14)
H39A	0.351(14)
H39B	0.351(14)
C40	0.351(14)
H40A	0.351(14)
H40B	0.351(14)
H40C	0.351(14)
C58	0.662(19)
H58A	0.662(19)
H58B	0.662(19)
C59	0.662(19)
H59A	0.662(19)
H59B	0.662(19)
H59C	0.662(19)
C60	0.338(19)
H60A	0.338(19)
H60B	0.338(19)
C61	0.338(19)
H61A	0.338(19)
H61B	0.338(19)
H61C	0.338(19)

Citations

CrysAlis^{Pro} Software System, Rigaku Oxford Diffraction, (2025).
O.V. Dolomanov and L.J. Bourhis and R.J. Gildea and J.A.K. Howard and H. Puschmann, Olex2: A complete structure solution, refinement and analysis program, *J. Appl. Cryst.*, (2009), **42**, 339-341.
Sheldrick, G.M., Crystal structure refinement with ShelXL, *Acta Cryst.*, (2015), **C71**, 3-8.
Sheldrick, G.M., ShelXT-Integrated space-group and crystal-structure determination, *Acta Cryst.*, (2015), **A71**, 3-8.
